# Polypharmacological Cell-Penetrating Peptides from Venomous Marine Animals Based on Immunomodulating, Antimicrobial, and Anticancer Properties

**DOI:** 10.3390/md20120763

**Published:** 2022-12-04

**Authors:** Shiva Hemmati, Haniyeh Rasekhi Kazerooni

**Affiliations:** 1 Department of Pharmaceutical Biotechnology, School of Pharmacy, Shiraz University of Medical Sciences, Shiraz 71345-1583, Iran; 2 Department of Pharmaceutical Biology, Faculty of Pharmaceutical Sciences, UCSI University, Cheras, Kuala Lumpur 56000, Malaysia; 3 Biotechnology Research Center, Shiraz University of Medical Sciences, Shiraz 71345-1583, Iran; 4 Student Research Committee, Shiraz University of Medical Sciences, Shiraz 71345-1583, Iran

**Keywords:** adjuvant, anticancer peptide, antigen-presenting cell, antimicrobial peptide, artificial intelligence, biofilm, immunotherapy, immune checkpoint, PDL-1 inhibitor, vaccination

## Abstract

Complex pathological diseases, such as cancer, infection, and Alzheimer’s, need to be targeted by multipronged curative. Various omics technologies, with a high rate of data generation, demand artificial intelligence to translate these data into druggable targets. In this study, 82 marine venomous animal species were retrieved, and 3505 cryptic cell-penetrating peptides (CPPs) were identified in their toxins. A total of 279 safe peptides were further analyzed for antimicrobial, anticancer, and immunomodulatory characteristics. Protease-resistant CPPs with endosomal-escape ability in *Hydrophis hardwickii,* nuclear-localizing peptides in *Scorpaena plumieri*, and mitochondrial-targeting peptides from *Synanceia horrida* were suitable for compartmental drug delivery. A broad-spectrum *S. horrida-*derived antimicrobial peptide with a high binding-affinity to bacterial membranes was an antigen-presenting cell (APC) stimulator that primes cytokine release and naïve T-cell maturation simultaneously. While antibiofilm and wound-healing peptides were detected in *Synanceia verrucosa*, APC epitopes as universal adjuvants for antiviral vaccination were in *Pterois volitans* and *Conus monile*. *Conus pennaceus*-derived anticancer peptides showed antiangiogenic and IL-2-inducing properties with moderate BBB-permeation and were defined to be a tumor-homing peptide (THP) with the ability to inhibit programmed death ligand-1 (PDL-1). Isoforms of RGD-containing peptides with innate antiangiogenic characteristics were in *Conus tessulatus* for tumor targeting. Inhibitors of neuropilin-1 in *C. pennaceus* are proposed for imaging probes or therapeutic delivery. A *Conus betulinus* cryptic peptide, with BBB-permeation, mitochondrial-targeting, and antioxidant capacity, was a stimulator of anti-inflammatory cytokines and non-inducer of proinflammation proposed for Alzheimer’s. Conclusively, we have considered the dynamic interaction of cells, their microenvironment, and proportional-orchestrating-host- immune pathways by multi-target-directed CPPs resembling single-molecule polypharmacology. This strategy might fill the therapeutic gap in complex resistant disorders and increase the candidates’ clinical-translation chance.

## 1. Introduction

Peptides are relatively safe and biocompatible with low toxicity. They have simple production, high selectivity, and specificity with tunable bioactivity. According to the definition of FDA and EMA, peptides contain less than 40 amino acid residues and originate from synthetic and natural resources [1]. Cell-penetrating peptides (CPPs) act as the central point in peptide-based drug delivery systems for macromolecular cargo internalization due to the hurdles on the way of transportation through the cell membrane [2]. These peptides are attached to the cargo molecule by electrostatic or covalent interactions as complexes or conjugates, respectively [3]. It has been pointed out in the definition of CPPs that they are not only cargo-delivery carriers but can potentially conduct intracellular reactions by themselves [4]. 

Naturally derived or rationally designed synthetic CPPs might have intrinsic biological functions, such as antimicrobial, anticancer, anti-inflammatory, and immunomodulatory properties. CPPs with innate immunomodulatory properties can be used to harmonize various pathological conditions. For example, they can penetrate and interact with intracellular Toll-Like Receptors (TLRs); hence, they are promising to trigger immunomodulatory responses applicable in vaccine adjuvants [5]. The other example is antimicrobial peptides (AMPs). Since AMPs can access intracellular microbial targets, they were considered potential delivery substances before the era of the CPP definition [4]. Introducing novel CPPs with AMP function is urgent due to the emergence of drug-resistant microbial strains called superbugs. Out of ten global health warnings, antibiotic resistance stands first, according to the WHO ultimatum [6]. However, in devising antimicrobial-drug-design criteria, the pathogen should be targeted by various mechanisms, including the attraction of the host-immune-system assistance. In this context, host defense peptides (HDPs) can target and clear pathogens directly, although their dominant antimicrobial function arises from their immunomodulatory properties [7]. Several AMPs might act as anticancer peptides (ACPs) with the ability to target the membrane or mitochondria of tumor cells. Despite the zwitterionic nature of normal mammalian cells, the negative charge of cancerous-cell outer membrane and bacterial membranes are similar. ACPs with cell-penetrating potential cross the tumor cell membrane passively or through endocytosis and might induce apoptosis via targeting mitochondrial membranes, inhibiting DNA synthesis, localizing in the nucleus, and initiating nuclear disruption. Therefore, cell-penetrating ACPs with immunomodulation properties are a newborn solution to multi-drug-resistant tumors [8]. To identify such inclusive peptides, novel plans for discovering therapeutics by big data analysis are required. This becomes possible by screening natural sources via accelerated growth in the next-generation high throughput technologies, such as venomics, peptidomics, and bioinformatics.

The main elements of venom are peptides and proteinaceous toxins, and have been translated into therapeutics on a number of occasions [9,10]. Toxins are usually resistant to proteases due to their disulfide-rich structure with high specificity and potency for particular targets. Melittin, anoplin, latarcin-1, bombesin, and crotamin are some examples of toxin-derived CPPs [11]. Due to the high abundance of venom, most toxin-derived therapeutics originate from snakes [12]. However, the primacy of life in the marine ecosystem over terrestrial land and harsh environmental conditions, such as deep variation in temperature, pressure, and nutrition, resulted in an extensive evolutionary diversity and myriad biological functions [13]. For example, invertebrate organisms, such as the *Conus* species, do not have adaptive immunity; thus, they are considered an intriguing origin of native-immunity-effector molecules, such as AMPs. FDA and EMA have approved Ziconotide as a cysteine-rich 25mer peptide derived from *Conus magus* conotoxin. This peptide is used as a non-opioid anti-hyperalgesic medication. However, it is injected intrathecally due to its poor penetration [14]. CSF delivery of Ziconotide is proposed as nose-to-brain delivery that bypasses the BBB [15].

Venomous animals distributed in the waters of some geographical areas, such as the Persian Gulf, are underexploited. The specific focus of the current study is the CPP-centered drug discovery with multifunctional-intrinsic-biological activity from Persian-Gulf venomous animals. This investigation is due to various reasons, such as the emergence of newborn pathogens and the not-yet-fulfilled requirements in cancer therapy. On the other hand, combination therapy in complex diseases, such as cancer, infection, and Alzheimer’s, has several drawbacks. These limitations are drug-drug interaction, distinct pharmacokinetic and pharmacodynamic profiles of combined medications, and toxicity, resulting in treatment failure or drug resistance [16]. Single-target monotherapy might also fail in complex conditions for various reasons, such as compensatory and neutralizing actions. Multi-target drug design for multifactorial disorders, such as anticancer tyrosine kinase inhibitors, increases treatment outcomes, reduces side effects, and lowers drug resistance [17]. Identification of CPPs with innate biological activity is like a one-pot generation of therapeutics instead of multistep conjugation of peptides with different biological properties. In other words, these peptides can eradicate pathogens or tumor cells and simultaneously modulate the immune system. We investigate how understanding the interface of peptide membrane interaction, tumor microenvironment, immunology, and microbiology results in a profound understanding of polypharmacological peptide therapeutic discovery and development.

## 2. Results and Discussion

Since the uptake efficiency of CPPs is not 100%, they can affect both extra- and intracellular compartments. This is only desirable when multi-target effects are required. Therefore, besides introducing CPPs suitable for drug delivery, we will emphasize CPPs that can target intracellular molecules and their microenvironment with the ability to interact with the immune-system components.

### 2.1. CPPs as Drug Delivery Carriers

Drug delivery by CPPs has a myriad of superiority over conventional methods, such as biocompatibility, and promising biophysical and biochemical properties. However, one of the most critical limitations hindering the translation of peptides into drugs is toxicity. Peptide toxicity is extensively classified into cytotoxicity, hemotoxicity, and immunotoxicity/allergenicity [18]. Therefore, safety for normal tissues should be considered in parallel to efficacy. In addition, proper physiochemical characteristics, such as lipid-binding potential, interaction with the target membrane, solubility, and propensity of forming aggregates, are critical aspects before devising a CPP for delivery purposes. Other factors like biodegradation and clearance that affect the half-life are also important [19]. In the next section, we emphasize the abovementioned issues to introduce the optimal CPP candidates for drug delivery.

### 2.2. Identification of CPPs and Analysis of Their Safety and Physiochemical Properties

**Figure 1:**
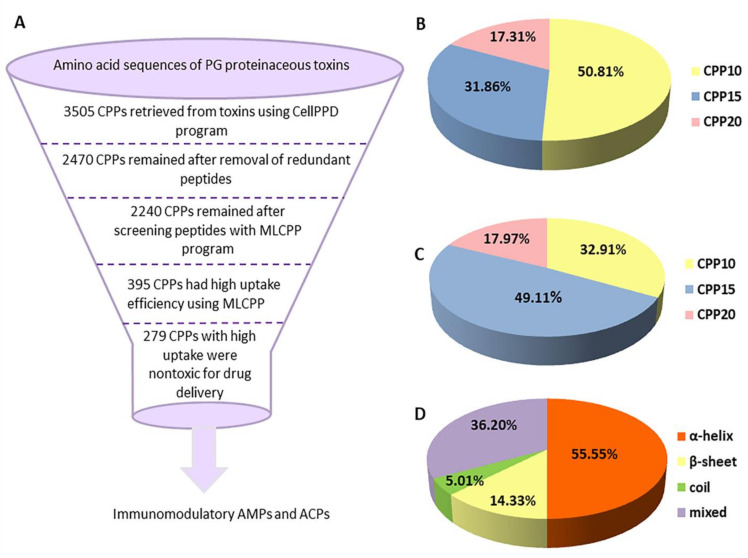
Flow chart and pie charts represent the screening of toxins and identification of cell-penetrating peptides (CPPs): (**A**) A flowchart that represents Persian Gulf-derived toxins, decoding toxins for CPPs, and screening for the safest peptides with high uptake efficiency; (**B**) Distribution of 3505 CPPs with a length of 10, 15, and 20 amino-acid residues according to CellPPD program; (**C**) Distribution of 395 non-redundant CPPs with a length of 10, 15, and 20 amino acid residues and high uptake efficiency according to MLCPP program; (**D**) The percentage of abundance distribution of 395 CPPs’ secondary structure.

A total of 82 venomous animal species of the Persian Gulf were retrieved by bibliography as classified in Table 1. Species were distributed in various phyla, including Cnidaria, Echinoermata, Chordata, Porifera, and Mollusca. Sequences of toxins were retrieved from the protein data bank and collected in Table S1. Despite the evolutionary trajectories of venoms, we could identify toxins with high similarities. This can be attributed to the clusters of synergistic peptides in venom that are critical for predation purposes in some species, such as cone snails. The evolution of these defense peptides guarantees the survival and diversification of more than 750 *Conus* species [20]. Using the CellPPD program, venom protein and peptide sequences were scanned to detect putative- encrypted CPPs with a fragment length of 10, 15, and 20 residues. A total of 1781, 1117, and 607 fragments were retrieved with a length of 10, 15, and 20 amino-acid residues, respectively (Figure 1). CPPs were annotated using the prefix “CPP”, a number indicating the length of the peptide, followed by a serial digit as CPP10-1 to CPP20-607 (Table S2). Then, redundant CPPs were verified, and peptides with high uptake efficiency were determined using MLCPP. A total number of 395 CPPs with high uptake efficiency were attained, 279 of which were non-toxic, according to the ToxinPred program, and submitted for further in-depth analyses (Figure 1) (Table S3). In investigations of membrane-active compounds, the hemolytic assay is a universal method for rapid toxicity estimation due to the ease of isolation of erythrocytes. Using HemoPI analysis, none of the 279 final CPP candidates were hemolytic (HemoPI scores: 0.41–0.55), compared to a hemolytic peptide like melittin, as the positive control (HemoPI score: 0.83). A total of 196 CPPs were predicted to be non-allergic, according to the AllerTOP program. Peptide aggregation lowers the peptide’s physical stability and might result in defective activity or side effects like toxicity and immunogenicity [21]. Except for the 19 peptides which showed a propensity to in-vitro aggregation with a score higher than 30, other CPPs were not prone to form aggregates. Hereafter, non-toxic, non-hemolytic, non-allergic, and non-aggregate forming peptides are proposed for selecting the optimal biologically active candidates.

All safe CPPs with high uptake efficiency can be used for drug-delivery goals (Table S3); however, the most prominent ones are introduced here. CPP10-1389 (RRRRSITRRG) displayed the highest probability of being a CPP, according to the first layer of the MLCPP program. This peptide is derived from a conotoxin (Uniprot ID: Q9BP75) expressed by the venom duct of *Conus tessulatus*. With a total net charge of +6 and a coil containing secondary structure, CPP10-1389 is classified as a cationic arginine-rich CPP similar to poly-Arg peptides (R9) and is totally unstructured. Non-amphipathic CPPs, such as R9 and TAT, remain unfolded even in the membrane environment and are translocated as random coil structures. Both electrostatic contact and membrane potential are impelling powers for CPP translocation. CPPs are internalized through direct membrane translocation at higher concentrations. They enter the cells via endocytotic energy-dependent mechanisms, such as phagocytosis or clathrin/caveolin-mediated routs, at lower concentrations [47]. CPP15-134 (KRQCRGVRVTRRSLR) is the second peptide with the highest probability of being a CPP derived from verrucotoxin (Q98993) in *Synanceia verrucosa* with a net charge of +7. CPP15-134 has a mixed coil and extended β-strand structure (CCCCCCCEECEEECC); however, a short peptide segment (residues 9–11) forms helix conformation after interaction with the membrane. CPP10-946 (KKGRKNLWRR) was a high-probability-predicted cationic CPP, rich in Lys and Arg residues, and is derived from *Conus betulinus* conotoxin (A0A142C1C7). This peptide has a hydropathicity of −2.62, indicating high stability in the aqueous medium. Notably, Arg and Lys residues interact distinctively with a membrane. Although both are basic residues and have a high amphipathic index, Arg is able to bind with higher affinity to the phosphate group of lipids than Lys’s amino group due to the guanidinium group of Arg. Besides, the guanidinium group’s polarity decreases in the binding state, forming a stacking contact with another guanidinium group to internalize the membrane. Hence, R9 is considered a CPP, while K9 is not able to permeate the membrane [48].

**Figure 2:**
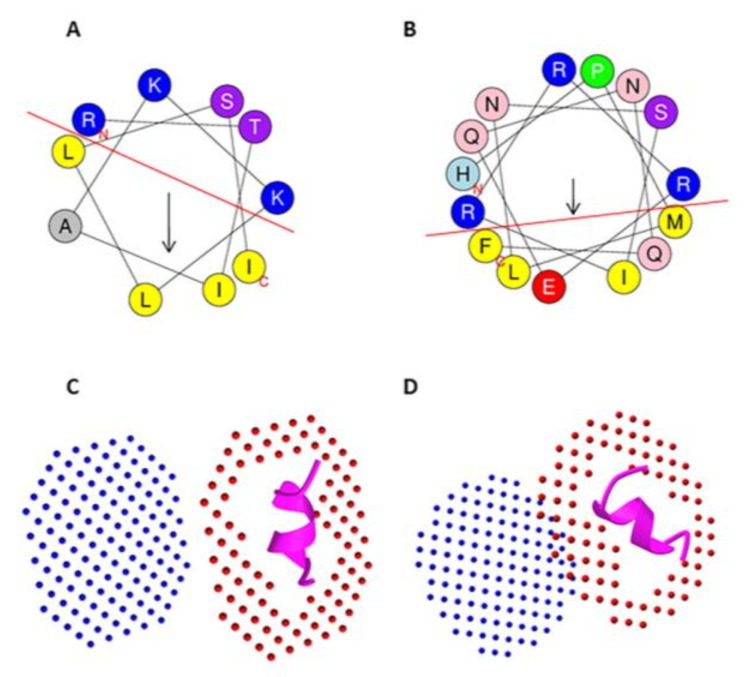
Helical-wheel projection and peptide-membrane interaction using Heliquest and FMAP programs, respectively: (**A**) Helical-wheel representation of CPP10-298 as an amphipathic CPP with segregated cationic and hydrophobic faces; (**B**) Presence of Glu (E) residue in the hydrophobic face of CPP15-382 facilitates endosomal escape; (**C**) Interaction of CPP10-421 as a mitochondrial targeting CPP with the mitochondrial-membrane model at 300 °K and pH = 7; (**D**) Interaction of CPP10-298 with the DOPC membrane.

Peptides often do not have a distinctive conformation but have flexible folding depending on the environment. While numerous peptides are unstructured in an aqueous solution, they fold into a defined conformation upon interacting with membranes [3]. Cell penetration occurs more efficiently when a CPP’s folding shifts to a helix or sheet conformation. The FMAP program models α-helical peptides in lipid membranes or membrane-mimetic environments and analyzes binding-free energy. CPP15-560 (RRSLKDFWKRHFYLR) and CPP20-156 (RAKINLLSKRKPPAERWWRW) with binding energies of −6.3 and −5.0 Kcal/mol are safe CPPs with strong binding potential to the zwitterionic DOPC membrane. The underlined residues represent regions that adopt helical conformation after interaction with the membrane. Although some CPPs have a more negative binding energy upon interaction with the membrane, they are predicted as probable allergens, which might be due to the mast-cell-membrane instability by these peptides and degranulation. Lipid-binding discrimination factors higher than 1.34, which is calculated according to the equation D = 0.944(<µH>) + 0.33(z), indicate a firm lipid-binding potential of the CPP candidates [49]. CPP15-560 derived from *C. betulinus* conotoxin (A0A142C1P9) and CPP20-156 from *Conus textile* conotoxin (Q9BHA0D) show D factors of about 2.03 and 2.26, respectively. CPPs that contain membrane-binding amphipathic helix parts, such as CPP10-298 (RTIAKKLLSI) from *Dendrochirus zebra*, adopt helical conformation via interaction with anionic head-groups of lipids and display segregation of the lipophilic and hydrophilic regions of an α-helix (Figure 2) [50]. This amphipathic-helix motif can show effectual interactions with membrane phospholipids, making the peptide a promising CPP candidate for drug delivery. CPP15-151 (LPILRRVRNTLEAMR) from *D. zebra* toxin (A0A068BFX2) is an amphipathic peptide showing a high percentage (86.66%) of helical content (underlined) upon interaction with the membrane with separated hydrophobic and polar faces, according to the helical-wheel projection. CPP15-151 has a hydrophobic moment of about 0.444 with a binding energy of −5.3 Kcal/mol to the DOPC membrane.

The bottleneck in the endocytosis-dependent CPP delivery is endosomal entrapment. One of the resolutions to overcome this obstacle on the roadmap of CPP delivery is mimicking viral-fusion proteins that can evade the endosome. The inclusion of synthetic- endosomal-escape domains that abolishes the entrapment in the endosome can also be applied to improve the activity [51]. Incorporating negatively charged residues, such as Glu, at the hydrophobic face of amphipathic peptides results in the protonation of the lysosome’s acidic environment, which in turn increases the hydrophobicity of the peptide, lysosomal-membrane fusion, and disruption [52]. This endosomal-escape potential has been predicted intrinsically for CPP15-382 (HPMLNSIRRREQNQF) from *C. textile* conotoxin (Q9BPJ4) as an amphipathic peptide with a hydrophobic moment of 0.315 (Figure 2). The other adopted strategy to circumvent endosomal entrapment is the incorporation of disulfide moieties [53]. In the oxidative condition of endosomes, Cys residues are able to form intramolecular-disulfide bonds to escape the endosome. This bond can then be reduced due to the high glutathione content in the intracellular compartment [54]. It should be mentioned that conotoxins are known as natural disulfide-rich peptides and are categorized based on their conserved cysteine framework [20]. Although most Cys-rich derived CPPs were predicted to be toxic in this study, we tried to identify non-toxic Cys-containing CPPs that are able to form intramolecular-disulfide bonds toward endosomal escape and higher stability against proteases. A total of 17 Cys-containing CPPs were predicted to have at least one disulfide bond, but the most optimal candidates are displayed in Table 2. Three candidates, including CPP10-352 from Cys-rich venom of *Hydrophis hardwickii*, and CPP10-1032 and CPP10-1344 from conotoxin in *Conus* species, were predicted to be resistant to proteases. It should be mentioned that the predicted disulfide bonds in Table 2 might not be native pairing, and the advantage of endosomal escape due to intracellular-disulfide bond formation should be further validated by in vitro and in vivo assays.

A high peptide’s chemical and physical stability increases the half-life of the candidate. There is a correlation between in vivo half-life of peptides and their bioavailability, distribution, clearance, and dosing. Although parenteral administration of peptides is a way to bypass gastrointestinal proteases, there are numerous systemic proteases/peptidases to cleave peptides and result in rapid hepatic and renal clearance. CPP20-551 (Q**A**KINFLSKRKPS**AE**R**W**RRD), CPP15-45 (KQLIRRHY**WE**IK**W**SG), and CPP20-173 (QR**A**KINLLSKRKPP**AE**R**WWE**) were cell-penetrating with the longest predicted half-lives of about 3643.51, 3246.11, and 3139.71 s, respectively. These three peptides are cryptic in *Conus pennaceus* γ-conotoxin (P56711), *Pterois volitans* Pvtoxin (F2ZAF0), and *C. textile* γ-conotoxin (Q9BPB0). A high frequency of negatively charged and small residues is observed in peptides having long half-lives. The presence of Ala, Glu, and, Trp displayed the highest correlation with the longer half-life bolded in the above sequences [55]. Conclusively, all the optimal candidates, achieved through safety and efficacy screening, could putatively enhance the preclinical validation success in drug delivery. Resolutions, such as terminal capping, stereochemistry modification, conformational freezing, cyclization, fatty acid conjugation, and physical shielding with nanoparticles, liposomes, or hydrogel are suggested to overcome shortcomings, such as protease susceptibility [56].

### 2.3. Blood-brain Barrier Penetrating Peptides

**Figure 3:**
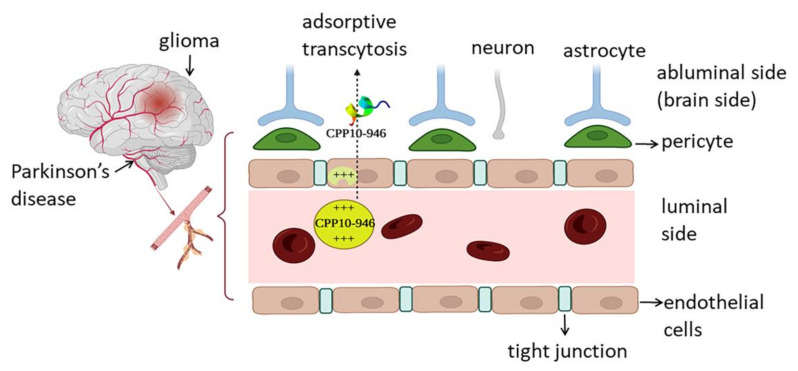
A schematic representation of blood-brain barrier and transportation of a conotoxin derived 10-mer CPP with BBB permeation ability from the luminal side of the brain microvasculature to the abluminal side.

The blood-brain barrier (BBB) is the most important physiological barrier in the brain cell microenvironment, separating the blood and the brain fluid. BBB hinders undesirable molecules from brain transition. According to the rule of CNS likeness, only passive penetration of a few lipophilic or low Mw medications is conducted through BBB [57]. Joined endothelial cells with sealed-paracellular-tight junctions surrounded by the basement membrane, pericytes, and the end-feet of astrocytes are the main components of BBB [58] (Figure 3). Intranasal delivery through olfactory and trigeminal nerves, lipidation, prodrug formation, and conjugation to the CPPs are known as non-invasive strategies to circumvent BBB. BBB-shuttle peptides are more favorable than the conventional Trojan-horse antibodies due to lower immunogenicity and facile synthesis [58]. BBB penetrating peptides might hijack endogenous receptor-, adsorptive-, and carrier-mediated transcytosis. CPP10-946 (KKGRKNLWRR) and CPP10-963 (KNFWKRNLYL), both derived from *C. betulinus* conotoxin (ID: A0A142C1C7 and A0A142C1Q1) and CPP10-757 (VRWYRNGTCR) from *Chanos chanos* stonustoxin (A0A6J2VWA1), were defined as the most optimal BBB permeable CPPs with a permeation score in between 0.34–0.43. The cationic peptide CPP10-946, with a total net charge of +6, a µH of about 0.438, and a D value of 2.39, is the most potent BBB-permeating peptide. The Tat_47–57_ peptide from HIV-1 transactivator of transcription and has been validated experimentally as a BBB permeating peptide that was used as the positive control with a predicted permeation score of 0.98 by B3Pred [57]. Altogether, the moderate potential of our candidates to penetrate CSF needs to be further evaluated in comparison to the positive controls experimentally. Drug delivery to the brain is mandatory for many CNS disorders, including Parkinson’s and Alzheimer’s disease, convulsion, depression, psychosis, and migraine, as well as CNS infections, such as meningitis, encephalitis, and glioma. Hence, introducing BBB-permeating peptides has been shown to be an effective minimally invasive treatment in future drug developments.

### 2.4. CPPs Containing Nuclear Localization Signals

Organelle-targeted design of therapeutics enhances the effect, lowers the side effect, and reduces drug resistance. Various nuclear-related disorders associated with gene expression and DNA impairment, such as cancer, requires nuclear drug delivery. The nuclear pore complex (NPC) acts as a gatekeeper for the transportation of macromolecules, such as proteins, across the double-membrane envelope of the nucleus [59]. Nuclear transporters recognize and navigate cytoplasmic proteins into the nucleus identifying sequences in proteins known as nuclear localization signal (NLS). TAT and polyarginine are examples of CPPs that are able to localize inside the nucleus [60]. A substantial cationic-charge distribution over a particular surface potentiates nuclear accumulation. Classical NLS (cNLS) are grouped as monopartite and bipartite depending on the distribution of positively charged residues, such as simian virus T antigen, derived NLS (PKKKRKV) and T53 binding protein 1 (GKRKLITSEEERSPAKRGRKS), respectively [61]. A total of 22 CPPs were predicted to have NLS characteristics using the cNLS Mapper in this study. A total of 17 peptides showed the “KRK” conserved motif in their sequences (Table S4). In sum, five detected NLS-containing peptides showed a score higher than 8.0, designating potential complete-nuclear localization, while 17 CPPs with a score of 7–8 showed partial-nuclear localization. We could identify CPP15-29 (KPKKLRPSTKDYWYI) from *P. volitans* pvtoxin (F2ZAF1) as an exclusive NLS candidate. CPP15-377 (KRPRENIRFLSKRKS) from *C. textile* conotoxin (Q9BHB7) was predicted to be a bipartite NLS peptide with a total net charge of +6 (Table 3). Accordingly, some targeting signals have been conjugated to CPPs to address desired cargo to the subcellular organelle [62]. For example, conjugating NLS to doxorubicin increased the cytotoxicity and anticancer selectivity of the medication [63]. Therefore, a CPP with intrinsic NLS characteristics is encouraging in drug design and development as it abrogates the requirement of an extra conjugation of peptides for nuclear delivery.

### 2.5. CPPs as Mitochondrial Penetrating Peptides

Mitochondria are the main energy producer of the body, and a wide variety of afflictions, such as muscular dystrophy, Lou Gehrig’s disease, diabetes, cancer, and dementia, are attributed to mitochondrial disorders [64]. The mitochondrial-phospholipid bilayer and its negative charge restrict drug delivery to this organelle. The inner membrane of mitochondria is greatly impenetrable with a substantial negative internal potential of about −180 mV that is needed for the electron-transport chain. Mitochondrial targeting CPPs (mtCPPs) or mitochondrial penetrating peptides (MPPs) target and penetrate this organelle. Within this study, 14 safe mtCPPs were defined using TargetP 2.0 program (Table S5). The most potent mtCPPs were CPP15-81 (KLQATIAKKLFAIRS), CPP20-272 (LLTRRSLKNFWKRNLYLRDE), and CPP15-144 (KLNLQRTIAKKLLSI), displaying −5.5, −6.0, and −4.6 Kcal/mol binding energies to the mitochondrial membrane-like environment, respectively (Table 3). CPP15-81 is derived from *Synanceia horrida* stonustoxin (Q91453), CPP20-272 originates from *C. betulinus* conotoxin (A0A142C1Q1), and CPP15-144 encrypted in *D. zebra* toxin (A0A068BD83). Experimentally validated mitochondrial-targeting peptides, such as MTP (MLSLRQSIRFFK) and MTS (MLRAALSTARRGPRLSRLL) [62] were used as the positive control and showed a likelihood of about 0.753 and 0.578 as mitochondrial-targeting peptides by targetP-2.0. Conjugation of the MTS + CPP and MTP + CPP successfully mediated mitochondrial localization of a dye and plasmid DNA delivery to the mitochondria, respectively [62]. Overall, CPPs with innate-mitochondrial-targeting characteristics, namely mtCPPs introduced in this study, provide a noteworthy outlook to bypass the extra-conjugation of a CPP and an MPP in constructing mitochondrial- targeting therapeutics.

### 2.6. Cytokine Inducing Peptides

The immune system constitutes cells and molecules, each with a particular defending contribution. The system has two different arms: innate immunity (it works the same in repeated exposure to a pathogen) and acquired immunity (it improves in repeated encounters due to memory). Natural or innate immunity is defined as physical, chemical, and microbiological barriers. It includes neutrophils, monocytes, macrophages, complement systems, cytokines, and acute-phase proteins that provide a rapid and primary defense. Acquired or adaptive immunity involves specific responses to antigens by T cells and B cells (cellular and humoral) [65]. Acquired immunity is more accurate, but it takes longer for initiation. The development of cytokine-based immunotherapy by activating or suppressing the immune system is of great interest in drug development. The effect of CPPs on the immune system has been less investigated to describe their off-target effects or their beneficial potential for therapeutic purposes [66]. Usually, CPPs have been used to conjugate inducers or inhibitors of the immune system [67]. For example, a peptide called CPPecp is a 10-mer peptide derived from human-eosinophil- cationic protein involved in immunomodulation for the treatment of asthma [68]. In the next subsections, we will investigate the effect of derived peptides in this study on the immune-system components.

#### 2.6.1. IL-2 Inducing Peptides

IL-2, a T cell-growth factor, is dominantly generated by activated CD4^+^ T cells in response to antigen triggers [69]. CD8^+^ T cells, dendritic cells (DC), and natural killer (NK) cells can produce a small amount of this cytokine. IL-2 (at low and medium quantities) can differentiate a class of CD8^+^ T cells into memory T cells [70]. IL-2 also differentiates CD4^+^ T cells into helper T cells, such as Th1, Th2, and Th17 [71]. Lack of IL-2 diminishes Treg-cell numbers and elevates the amount of conventional effector T cells that, in turn, results in an incremental vulnerability to autoimmune, inflammatory, and metabolic disorders [72,73]. In other words, IL-2 acts as an immunosuppressant at low doses applicable to control autoimmune disorders and an immunostimulant at high doses beneficial for cancer immunotherapy [74]. High-dose IL-2 therapy has FDA approval for metastatic melanoma and metastatic-renal-cell carcinoma known as aldesleukin (Proleukin^®^) [75]. 

Constitutive activation of IL-2 harms effector T cells in TME and promotes cytotoxic T lymphocyte (CTL) exhaustion and tumor relapse. Therefore, the dose and duration of IL-2 therapy should be controlled as it poses pleiotropic functions by employing protumor and anti-tumor activities. High doses of recombinant aldesleukin that is administrated of about 600,000–720,000 IU/Kg can exert toxic side effects, such as vascular leak syndrome (VLS), which leads to life-threatening edema [69]. Therefore, other strategies, such as the production of engineered IL-2 mutants or IL-2 inducing epitopes/antigens are adopted [76]. IL-2- inducing peptides have MHC-binding affinity and activate IL-2 secretion. In this study, the strongest putative inducers of IL-2 secretion had a length of about 10 aa residues, while the weakest inducers had a length of 15–20 residues (Table S3). A cysteine-rich peptide known as CPP10-1537 (CSKICWRPRC) derived from a conotoxin fragment (Q9UA85) in *Conus abbreviates* was defined potentially as the strongest IL-2 inducing peptide.

Angiogenesis results in endothelial anergy in tumor cells and hampers endothelial- adhesion-molecule overexpression and infiltration of leukocytes. Therefore, a combination therapy containing antiangiogenics and immunotherapeutics overcome immune evasion [77]. Combination therapy of high-dose IL-2 and VEGF inhibitors was promising in metastatic-melanoma therapy [78]. This observation can be extrapolated to a strong IL-2-inducing peptide having antiangiogenic characteristics simultaneously to decrease TME resistance toward IL2 therapy. A total of 77 candidates were proper IL-2 inducers with antiangiogenic properties; still, CPP10-1537 was the strongest optimal candidate for this purpose. IL-2 therapy might result in VLS and patients with extravascular-fluid retention are more susceptible to infection [79]. We suggest IL-2 inducing candidates with intrinsic AMP properties to prevent such infections. Totally, 14 peptides were both IL-2 inducers with AMP characteristics. CPP10-299 (TIAKKLLSIR), derived from *D. zebra* toxin (A0A068BD83), is a helical-amphipathic peptide amongst strong IL-2 inducing candidates with AMP characteristics, according to the CAMP_R3_ program. It is active against Gram-positive bacteria, such as *S. aureus* with a MIC lower than 25 µg/mL. CPP10-946 (KKGRKNLWRR) and CPP20-2 (RCTAFQCKHSMKYRLSFCRK) are the most optimal IL-2 inducers with AMP and antiangiogenic properties simultaneously. CPP20-2 is a cryptic peptide in *Stichodactyla haddoni* κ-stichotoxin (E2S062). Taken together, introducing peptides with the intrinsic ability to induce IL-2 secretion with antiangiogenic and tumor-homing characteristics provides a perspective of an all-inclusive therapeutic targeting TME instead of combinatorial therapy.

#### 2.6.2. IL-4 Inducing Peptides

**Figure 4:**
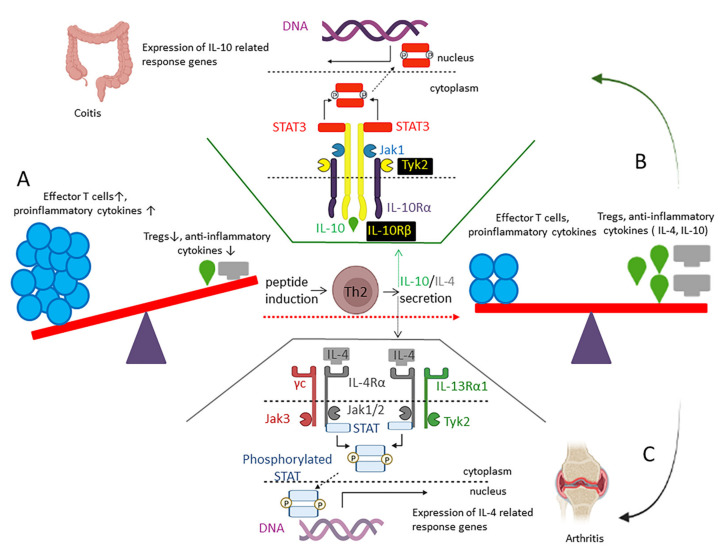
Immunotherapy of autoimmune disorder mediated by peptides as inducers of cytokines: (**A**) While the dominance of effector T cells and proinflammatory cytokines prone the patients to autoimmune disorders, such as colitis and rheumatoid arthritis, peptides that are inducers of anti-inflammatory IL-4 and IL-10 cytokines restore disturbed immune hemostasis in autoimmune disorders; (**B**) IL-10 exerts its anti-inflammatory response through its receptors and the JAK/STAT signaling pathway; (**C**) IL-4 forces its anti-inflammatory effect via its receptors and the JAK/STAT signaling pathway.

Interleukin 4 (IL-4), a main immunomodulatory cytokine, is produced predominantly by Th2 cells, eosinophils, mast cells, and basophils (Figure 4) [80]. IL-4 triggers the differentiation of naïve Th cells to Th2 and stimulates associated Th2-type reactions. IL-4 enhances the secretion of IgE by antibody-class switching, develops basophils, eosinophils, and mast cells, and polarizes macrophages into the anti-inflammatory M2 subtype. It inhibits the secretion of IL-1, IL-6, and tumor necrosis factor (TNF) as pro-inflammatory cytokines [81]. IL-4 protects cartilage in rheumatoid arthritis, restrains matrix metalloproteinases, and inhibits IL-1 as a pro-inflammatory cytokine in the synovium [82]. Transfection of mesenchymal-stem cells with IL-4 enhances anti-inflammatory and chondroprotective impacts in an osteoarthritis-chondrocyte model after intra-articular implantation [83]. IL-4 enhances insulin sensitivity in type 2 diabetes, interferes with lipid storage by improving lipase activity, and promotes adipocytes’ lipolysis, resulting in reduced weight and fat mass [84,85]. There is a hypothesis that assumes that β amyloid peptide (Aβ42) aggregates into toxic β-sheet assemblies, triggering a cascade of neurotoxic events, including neuroinflammation, neurodegeneration, and cognitive impairment in Alzheimer’s disease. IL-4 restores progenitor plasticity, neurogenic capacity, and network-formation ability of primary- human-cortical astrocytes and neurons [86]. The immediate safe signal related to IL-4 is the modulation of responses to infection with helminthic parasites [87]. However, the therapeutic significance of systemic IL4 might be curtailed due to side effects. For example, uncontrolled signaling of IL-4 confers allergic reactions, such as asthma and dermatitis [88]. Therefore, a rapid channeling of IL4 from the systemic circulation into the target tissue has been considered in some studies [82].

This study defined a total of 101 IL-4 inducing peptides. The strongest IL-4 inducing peptides had a length of 15–20 aa residues (Table S3). An anionic IL-4 inducing peptide (ASDVETAEGGEIHELLRLQ) derived from the measles virus was used as the positive control, which showed an SVM score equal to 1.0 using IL4Pred [89]. Therefore, IL-4 inducing candidates within this study with predicted scores between 0.20 and 0.67 were considered moderate IL-4 inducers. CPP20-70 (RKAEINFSETRKLARNKQKR) derived from γ-conoxin like peptide (P24160) in *C. textile* as a cationic-helical peptide was the most optimal/potent IL-4 inducing peptide. CPP20-70 was non-allergic and was proposed for the amelioration of autoimmune disorder, such as arthritis. CPP15-134 (KRQCRGVRVTRRSLR) is a cationic IL-4 inducing peptide with BBB permeation ability and would be offered in Alzheimer’s disease.

It has been shown that combination therapy of IL-4 and IL-10 is potentially superior to stand-alone therapy because of synergistic anti-inflammatory properties. IL-4 + IL-10 fusion protein displayed favorable therapeutic potential in rheumatoid arthritis [90]. A total of 55 peptides with proper physiochemical properties had putative IL-4 and IL-10 inducing properties simultaneously; yet, CPP20-70 with intrinsic dual IL-4 and IL-10 inducing ability was the best candidate. Therefore, CPP20-70 is potentially an extraordinary candidate for targeted delivery to the synovial fluid for cartilage protection. To avoid systemic side effects, intrasynovial injection and preferably topical tapes loaded with these penetrating peptides for transdermal delivery are suggested. Ultimately, due to the systemic side effects, investing in topical formulations for the delivery of IL-4 inducing peptides seems to be safer than systemic administration.

#### 2.6.3. IL-10 Inducing Peptides

A prolonged or extreme immune-response results in auto-immune disorders. This condition needs to be overcome by immunosuppression moderated by anti-inflammatory cytokines, such as IL-10. IL-10 secretes from various myeloid and lymphoid lineage cells [91]. The induction of pattern recognition receptor (PRR) signaling pathways, such as TLR-activated DCs through various mediators, induces IL-10 transcription [92]. Other cytokines are also able to induce IL-10 production. IL-10 receptor (IL-10R) has two subunits known as high-affinity IL-10Rα, mainly expressed on leukocytes, and a ubiquitously expressed IL-10Rβ. IL-10 induction exerts its anti-inflammatory response via receptor oligomerization and recruitment of the JAK1/STAT3 signaling pathway (Figure 4). Other cascades, such as PI3K/AKT/GSK3 pathways, have also been reported for IL-10 signaling in macrophages [93].

IL-10 limits the efficient expansion of T cell response by inhibiting antigen presentation through transcriptional inhibition of cytokines, MHCII, and adhesion molecules. It might also directly inhibit memory and effector T cells [92]. The most in-depth investigation on the role of IL-10 is devoted to its role in ameliorating inflammatory bowel diseases (IBD), such as colitis and Crohn’s disease, and intestinal-wound healing [94,95]. In addition to its effect on immune cells, IL-10 is shown to play hemostatic roles in non-hematopoietic cells. For example, the STAT3-Nfkb pathway involves axon regeneration and restricts neural damage during infection, trauma, or ischemia [96].

A total of 145 peptides were predicted to be IL-10 inducers according to the IL-10Pred program, and 101 peptides had proper physiochemical properties (Table S3). A helical- cationic peptide derived from *C. textile* conotoxin (Q9BHB7) named CPP15-376 (EKRPRENIRFLSKRK) was the optimal IL-10 inducing peptide. Other candidates are CPP20-24 (ERHKKREYNVRAPNPGFKRL), which originated from *S. horrida* stonustoxin (Q98989) and CPP15-611 (YKRLLQRPARRMDRG) from *Conus caracteristicus* conotoxin (V5V9Y7). CPP15-560 (RRSLKDFWKRHFYLR) with a µH of 0.407, a total net charge of +6, a lipid-binding discrimination factor of about 2.03, and binding energy of about −6.3 Kcal/mol to the DOPC membrane, is a moderate IL-10 inducing peptide with acceptable BBB permeability and antioxidant activity. This candidate might be potentially beneficial in Alzheimer’s disease [97]. However, it should be noted that IL-10 is now known as a pleiotropic cytokine that may have a hitherto-dual function, which boosts or represses the immune response depending on the cell and context [92].

#### 2.6.4. IFN-γ Inducing Peptides

While type I interferons (IFN-α/β) predominantly act as antiviral agents, type II interferon (IFN-γ) is implicated in immunoregulation with anti-bacterial, anti-parasitic, and anti-tumor properties. IFN-γ is not induced by PRRs but through cytokine-stimulated immune cells [98]. Native IFN-γ is secreted by CD4^+^ Th1 cells and CD8^+^ cytotoxic T cells, which is important in adaptive immunity. Production of IFN-γ by NK cells and APCs are vital for early host reaction towards infection. IFN-γ has two receptor subunits, namely high-affinity IFNGR-α (IFNGR1) and low-affinity IFNGR-β (IFNGR2) receptors, that are responsible for binding and signal transduction, respectively [99]. 

INF-γ is well known to trigger cell-autonomous immunity to eradicate intracellular parasites, such as *Toxoplasma gondii*, *Trypanosoma cruzi*, *Leishmania donovani*, *Chlamydia trachomatus*, and *Mycobacterium tuberculosis*, responsible for toxoplasmosis, Chagas disease, kala-azar, chlamydial infections, and tuberculosis, respectively [100,101,102]. One of the dominant IFN-γ dependent antimicrobial responses is mediated through the production of superoxide anion or nitric oxide, and finally destroys phagolysosome-containing pathogens [101]. Besides, IFN-γ facilitates microbial clearance by inducing antimicrobial peptides, such as α/β-defensins and cathelicidins [100]. Human INF-γ is produced recombinantly in *E. coli* and is branded as Actimmune^®^ with FDA approval to reduce chronic granulomatous disease-related infections (CGD) with recurrent infections and to delay the progression of severe malignant osteoporosis [103]. Cytokine intervention using recombinant IFN-γ is a high-dose invasive therapy with side effects such as, lethargy, cough, atherosclerosis, and suppressed nervous system. IFN-γ inducing peptides can elevate the cytokine’s titer in a much less aggressive form with a smart control on immunotherapy [100].

A total of 167 IFN-γ inducing peptides have been detected by IFNepitope program, 121 of which had proper physiochemical characteristics (Table S3). CPP20-404 (RQKHRALRSTDKNIKLTRRC) was a helical-cationic peptide derived from *Conus striatus* conotoxin (P0C833) defined as the most potent and safe IFN-γ inducing peptide. CPP20-156 has a dual mechanism against tuberculosis, simultaneously with strong anti-tubercular and IFN-γ induction. IFN-γ inducing peptides are incorporated as an adjuvant in constructing multi-epitope vaccines for the vaccination against a plethora of pathogens [104,105]. Therefore, not only the candidate mentioned above, but also CPP20-564 (QAMQRDAINVRRRRSITRRV) from *C. pennaceus* conotoxin (Q9BP64) and CPP10-1389 (RRRRSITRRG) are significant IFN-γ inducers with immunoadjuvant properties suitable as vaccine adjuvants, according to IFNepitope and VaxinPAD, respectively. Altogether, IFN inducing peptides show promising perspectives for modulating TME and higher responsiveness to anti-tumor therapy and moderating the antimicrobial capacity upon pathogen attack. A total of 167 IFN-γ inducing peptides have been detected by IFNepitope, 121 of which had proper physiochemical characteristics (Table S3). CPP20-404 (RQKHRALRSTDKNIKLTRRC), as a helical-cationic peptide derived from *Conus striatus* conotoxin (P0C833) was defined as the most potent and safe IFN-γ inducing peptide. CPP20-156 has a dual mechanism against tuberculosis, simultaneously with strong anti-tubercular and IFN-γ induction. IFN-γ inducing peptides are incorporated as an adjuvant in the construction of multi-epitope vaccines for the vaccination against a plethora of pathogens [104,105]. Therefore, not only the candidate mentioned above, but also CPP20-564 (QAMQRDAINVRRRRSITRRV) from *C. pennaceus* conotoxin (Q9BP64) and CPP10-1389 (RRRRSITRRG) are significant IFN-γ inducers with immunoadjuvant properties suitable as vaccine adjuvants, according to IFNepitope and VaxinPAD, respectively. Altogether, IFN-γ inducing peptides show promising perspectives for modulating TME and the antimicrobial capacity upon pathogen attack, and higher responsiveness to anti-tumor therapy.

#### 2.6.5. TNF-α Inducing Peptides

TNF-α is mainly a proinflammatory cytokine and is produced by circulating monocytes, macrophages, activated T cells, NK cells, and non-immune cells, such as fibroblast and endothelial cells. This cytokine is a central intermediary in the immunological reactions to manage infectious diseases, autoimmune disorders, and neoplasm. The TNF superfamily has 19 members with more than 40 receptors. TNF-α, which exists as a transmembrane or soluble form, has two receptors called TNFR1 and TNFR2 [106]. TNFR1 is critical in promoting cytotoxic and proinflammatory reactions, while TNFR2 is mainly a mediator in cell migration and proliferation [107].

TNF induces apoptotic cell death through TNFR1 in cancer cells, a minor reaction usually covered by the dominant antiapoptotic mechanism of TNF-α via NfkB signaling activation that results in the expression of proinflammatory cytokines, such as IL-6 [108]. TNFα inhibitors are considered future strategies in cancer treatment. Passive immunization by anti-TNF drugs, such as adalimumab or infliximab, is highly appreciated for rheumatoid arthritis and IBD. However, as these medications bind to both soluble forms and membrane-bound TNF, TNF’s function should be privileged in normal physiology. Anti-TNF drugs have an immunosuppression effect, and increase the risk of infection and malignancies [109].

TNF-α has a highly potent antiviral activity through both receptors. TNF-α can display an antimicrobial effect through TNFR1-mediated apoptosis by killing the viral-containing cell. It recruits inflammatory cells, induces inflammatory cytokines to attract immune cells, matures APCs to stimulate naive T cells to initiate antigen-specific cells, and differentiates macrophages to promote the M2 phenotype. TNF-α also has a dominant role in the inflammatory and proliferation phases of wound healing. A total of 170 TNF-α inducing peptides were predicted using the TNFepitope program, 112 of which were non-allergic without forming aggregates. The most potent TNF inducing candidate to combat pathogens in the acute phase of infectious diseases were CPP20-272 (LLTRRSLKNFWKRNLYLRDE) from *C. betulinus* and CPP20-45 (KRQCRGVRVTRRSLREFSHF) from *S. verrucosa* verrucotoxin (Q98993). Altogether, TNF-α inducing peptides show optimistic expectations in combating specific bacteria, fungi, and viruses, with wound-healing characteristics, which will be further analyzed in Section 2.8

### 2.7. Immunoadjuvant Peptides

Various strategies in developing vaccine platforms should be adopted to prepare against future pandemics and provide more efficient active immunization to prevent cancer and infectious diseases. The administration of antigens alone will not always be adequately immunogenic to provide prolonged protection. Incorporating adjuvants is considered in all vaccine types, including inactivated, live-attenuated toxoid, subunit, recombinant, and mRNA-based platforms [110]. Adjuvants enhance vaccine efficacy by enriching antigen immunogenicity, expanding the duration, type of provoked response, and vaccine stability. They are beneficial for enhancing immunogenicity in populations with an inadequate response and sparing antigen dose. Despite myriad progress in immunology, fewer than ten adjuvants have received FDA approval until now [111]. Current adjuvants have some limitations, such as inadequacy in granting full immunity, the requirement of supplementary doses, and a narrow coverage in the elderly and children population [112]. In addition, the inflexible global-vaccine reservoir and healthcare sources are critical logistic concerns. Therefore, developing novel adjuvants with new targets is inevitable.

It has been shown that a series of short-immunomodulatory peptides derived from host-defense peptides, namely A-cell epitopes, can trigger PRRs on APCs, such as macrophages and dendritic cells [113]. These A-cell epitope antigens (named immunoadjuvant peptides in this study) affect the breadth and amplitude of immune response to fine-tune the efficacy of subunit vaccines. These peptides act as ligands for innate immune receptors that stimulate cytokine production via signaling processes. The cytokines direct the development of naïve immune cells into mature adaptive cells, such as diverse classes of T-lymphocytes.

A total of 183 peptides were predicted to have immunoadjuvant properties, and 120 were non-allergen without forming aggregates (Table S3). The strongest candidates were CPP10-583 (NRFVRIGRRD) originating from *C. textile* conorfamide (P0DM27), CPP10-859 (KPLKRRVKQY) from *C. betulinus* conotoxin (B0KZ78), and CPP15-1042 (DAINVRRRRSITRRV) from *C. pennaceus* conotoxin (Q9BP64), containing 40%, 20%, and 40% Arg content, respectively. These results were similar to the report of Nagpal et al., 2018 that the most potent candidates were Arg-rich [113]. Interestingly, CPP10-859 and CPP15-1042 were strong immunoadjuvants predicted to have antimicrobial properties. Hence, they might boost the immune system and simultaneously target the pathogen, which will be discussed in Section 2.8.1. LL-37 is a human cathelicidin that is accumulated intracellularly as hCAP18. The hCAP18 is cleaved after secretion to produce mature active LL-37. Therefore, LL-37, an immunomodulatory peptide, was used as the positive control and showed an SVM score of 0.02 using the VaxinPAD program. This means that the most optimal candidates found in this study, with SVM scores higher than 1.00, are at least 50 times more potent than LL-37. These peptides are safe and efficient off-the-shelf adjuvants with novel mechanisms promising for incorporation into the vaccine formulation.

### 2.8. Antimicrobial Peptides

While the current mortality of antibiotic-resistant complications is more than a million, this rate is expected to reach at least ten times higher by 2050, far greater than diabetes and cancer, costing economically 66 trillion pounds sterling [114]. Therefore, discovering alternative therapeutics with a multi-mechanistic approach to target the pathogen and simultaneously boost the host-immune system is an unavoidable demand. On the other hand, detecting and remedying intracellular pathogens is challenging as they might stay inactive and survive in therapy, leading to recurrent and chronic infections. As the concentration of antibiotics in the host-intracellular compartment is lower than their MIC, numerous treatments for internalized microorganisms are inefficient at therapeutic doses, which can result in additional drug resistance [115]. This makes cell penetrating AMPs a fantastic strategy to clear the intracellular compartment.

AMPs are usually known as broad-spectrum antibiotics with evolutionary diversity that target fungal, viral, bacterial, and parasitic diseases with a much lower probability of microbial resistance than conventional antibiotics [116]. AMPs have 10–50 aa residues and are an essential player in the innate immunity of living organisms. With a total net charge of +2 to +9, AMPs are usually cationic and act as the first defense layer against microorganisms. AMPs interact with the anionic-bacterial cell wall by electrostatic forces; then, hydrophobic interactions of AMPs and the acyl chains of the bacteria enable non-receptor-mediated peptide permeation [117]. This means that they might act through membranolytic or non-membranolytic mechanisms by affecting intra-microbial targets. Due to relatively high net charge, which results in electrostatic repulsion among peptides and non-high mean hydrophobicity in short AMPs, they are usually unstructured in an aqueous solution without aggregation and adopt a unique conformational-secondary structure in the membrane environment [118]. Membrane potential, which is correlated with the translocation capability of AMPs, is markedly higher in the case of the prokaryotic cell than eukaryotic [118]. Therefore, AMPs mainly target microorganisms than the host cell as the membrane potential of the former is about −130 to −150 mV, while mammalian cells have a membrane potential of −90 to −110 mV [119]. CPP-membrane interactions disrupt lipid arrangement. Therefore, in microbial membranes where anionic lipids are more elevated, the peptide-membrane interaction is fortified with higher perturbations and higher local concentrations on their surface.

To define AMPs within this study, CAMP_R3_ program was used. Because of the usage of a large training dataset, this program showed the highest performance in predicting AMPs, compared to other freely available web-based portals [120]. A total of 52 peptides showed AMP properties according to all performed models in CAMPR3, including SVM, RF, DA, and ANN (Table S3). A perfect therapeutic peptide should have a high therapeutic index (TI), which means high therapeutic activity with the lowest RBC lysis. The TI of AMPs was between 5 and 75 in this study. All defined AMPs had a total net charge between +2 and +7. According to the RF method that outperforms in prediction among other models, CPP15-81 (KLQATIAKKLFAIRS) was putatively the most potent broad-spectrum AMP. CPP15-81 has a binding affinity of −6.1 and −5.5 Kcal/mol towards Gram-positive and Gram-negative bacterial membranes using the FMAP program. Gram-negative bacteria have a thin peptidoglycan coating and an additional outer membrane, compared to the thick peptidoglycan surface observed in Gram-positive bacteria. Analysis with DBAASP shows that CPP15-81 is active against *Staphylococcus aureus*, *Pseudomonas aeruginosa*, *Klebsiella pneumonia*, and *Escherichia coli* with a MIC lower than 25 µg/mL. While defining the exact mechanism of AMPs is complicated, the effect is highly dependent on peptide-membrane interaction. The peptide-membrane interaction mode depends on the membrane composition, the peptide-physiochemical features, and peptide concentration. For example, CPPs at a particular concentration attain AMP characteristics [118]. CPP15-81 is a broad-spectrum amphipathic peptide with a µH of 0.349 and a D factor of 1.64, reconfirming its lipid-binding potential. CPP15-81 shows a Boman index of 4.62 Kcal/mol. Boman indices above 2.48 indicate an AMP with protein-binding potential [121]. Hence, CPP15-81 might target proteinaceous targets in the pathogen, in addition to membrane targeting (Table 4).

Although, according to the secondary structure, AMPs can be classified into four distinct groups, α-helical and β-strands are more common. In an aqueous solution, α-helical AMPs are linear and adopt amphipathic-helical conformation when they interact with bacterial membranes or organic solvents. β-stranded AMPs are more structured in aqueous media with less conformational variations upon interaction with the membrane and usually contain disulfide bridges to stabilize their structure, such as CPP15-245 (ARRTPRMLSNKRICC) in *C. textile* conotoxin (Q3YEH7). Out of the 52 predicted AMPs, 86.53% had a helical segment in their structure after interaction with the membrane (Table S3).

Pharmacodynamically, AMPs differ from traditional antibiotics with a narrower therapeutic window. They destroy target pathogens within minutes rather than hours for antibiotics [116]. Conventional antibiotics take days to extinguish *Mycobacterium tuberculosis*, a slow-growing bacterium; however, a defensin called HNP-1 shows antitubercular properties within hours [122]. Out of the 52 AMPs predicted by CAMPR3, CPP20-155 (QRAKINLLSKRKPPA**E**RWW**R**) originated from *C. textile* γ-conotoxin (Q9BHA0) is a potential AMP with the longest half-life (48 min), is effective against *E. coli*, and shows antitubercular activity. The long half-life of this peptide can be attributed to the stabilizing effect of salt-bridge formation between the acidic residues Glu and cationic residue, Arg, distributing in i and i + 4 distances. This peptide has a higher binding affinity to the bacterial membrane (−5.5 Kcal/mol) than the host membrane (−5.2 Kcal/mol). CPP20-155, with a µH of about 0.311 and a total net charge of +6, shows a high lipid-binding potential.

Some motifs in AMPs, such as the heptapeptide known as Rana box, resemble antibiotics, such as colistin, and boost the effectiveness against antibiotic-resistant *S. aureus* and *P. aeruginosa*. Rana box is the C-terminal motif, including “CXXXXXC” or “CXXXXC”, wherein two Cys residues are separated by four or five amino acids and are able to form a cyclic-disulfide bond [123]. CPP15-596 (VRRCCSRDCRVCIPC), a *C. betulinus* conotoxin-derived peptide (A0A142C1G6), contains a C-terminal Rana box motif and is predicted to be antimicrobial using CAMP_R3_ program. However, this peptide is toxic due to the presence of a CC dipeptide composition, and a C^5^F mutation is necessary to abrogate the toxicity. This peptide retains AMP activity even after mutation with good water solubility without aggregation. The MIC value of CPP15-596 decreased from 43.20 to 37.58 µM after mutation, according to the MICpredictor (http://splitbioinf.pmfst.hr/micpredictor/) (accessed on November 2022).

To translate newly introduced defense peptides to clinical applications, the synergistic potential of these peptides in combination with approved antibiotics lowers the quantity of each therapeutic in the administered cocktail, which in turn declines the evolution rate of drug resistance and lowers the side effects. This has been observed in the combination therapy of LL-37 and azithromycin via increasing antibiotic-cell permeation [124]. Interestingly, AMPs can be combined for the observation of synergistic effects. For example, a combination of three AMPs has superior synergistic effects than a cocktail of two in the case of Temporins A, B, and, C against Gram-positive or Gram-negative bacteria. The synergy might be due to the formation of heterodimeric or heterotrimeric complexes between peptides in the membrane-lipid bilayer, improving membrane binding [118]. Therefore, the trait of combinatorial therapy with peptides could be adopted for the development of future anti-infective therapeutics.

#### 2.8.1. Immunomodulatory AMPs

In order to move to the post-antibiotic time, novel antimicrobial modalities, such as host-directed treatments, should be adopted. Multifunctional AMPs that target the microorganism directly and modulate the immune system and the infection microenvironment are becoming a new paradigm in antimicrobial therapy. Immunomodulation might be exerted through immunostimulation, immunosuppression, or immunoadjuvants. Immunotherapy ameliorates a disorder by harmonizing the immune system. Immune hyperactivation and immunosuppression are the main observed responses of the immune system in the early acute and late chronic phases of infection, respectively [125].

Currently available immunomodulatory drugs have severe side effects for the patient. Natural peptide immunomodulators have no or few side effects [65]. The association of damage-associated molecular patterns (DAMPs) and pathogen-associated molecular patterns (PAMPs) with host pattern recognition receptors (PRRs) triggers NF-κB and a low-level HDP expression which intensifies after colonization, inflammation, and cell injury. As a primary responder, macrophages detect and eradicate pathogens and tumor cells by phagocytosis. Macrophages also act as immunoregulatory agents by secretion of cytokines, such as IFN-γ, TNF-α, IL-1β, IL-6, IL-10, and IL-12. HDPs, such as cathelicidins and defensins, as the ancient evolutionarily elements of the innate immune system, either straightly destroy microorganisms or perform through immunomodulatory mechanisms by stimulating innate immunity or connecting innate and adaptive immunity. LL-37 has a variety of immunomodulatory functions, some of which are boosting pro-inflammatory cytokines, such as IL-6, and its receptor or anti-inflammatory cytokines, such as IL-10 and IL-4 [7]. Cathelicidins, which act as TLR agonists, are added as vaccine adjuvants [126]. Relatively, similar production of pro- and anti-inflammatory cytokines has been reported for some human α- and β-defensin HDPs [127]. However, proteinases secreted from pathogens, such as *S. aureus*, cleavehuman defensin, resulting in bacterial resistance [127]. Therefore, introducing novel AMPs with immunomodulatory properties is in great demand.

We have defined the intersection of IL-6, TNF-α, INF-γ, and IL-10 inducing peptides with 52 AMPs to introduce putative-immunomodulatory-antimicrobial peptides. A total of 11 peptides, including CPP20-48, CPP20-2, CPP20-156, CPP20-155, CPP15-1050, CPP15-387, CPP15-195, CPP20-184, CPP20-557, CPP20-45, and CPP20-51 were achieved. The most optimal candidate was CPP20-48 (KKICNDYKLNLQRTIAKKLL) from *D. zebra* toxin (A0A068BD83), which stimulates APC and acts as the inducer of IL-6, TNF-α, IL-10, and IFN-γ. CPP20-48 can straightly attack the pathogen having a high affinity to the inner membrane of Gram-negative bacteria (Binding energy of −5.3 Kcal/mol) than the host membrane (−5.0 Kcal/mol). This peptide has a mixed α-helix, β-strand, and coil structure with a hydropathicity of −0.57, indicating stability in the aqueous medium. A total of two peptides derived from tuna fish protein hydrolysates (*Thunnus albacares*) with the consensus sequences HIAEEADRK and AEQAESDKK were used as the positive control. Both peptides have strong immune activity by inducing phagocytosis and promoting the secretion of NO, IL-1β, IL-6, and TNF-α [128]. We showed that both peptides are IL-6 inducing using the IL-6pred program with RF-based scores of 0.76 and 0.74, respectively. In addition, both peptides were TNF-α inducers, according to the TNFepitope program, with hybrid scores of about 0.51 and 0.66, respectively. A modified antimicrobial peptide derived from Clavanin observed in the marine organism *Styela clava* (FLPIIVFQFLGKIIHHVGNFVHGFSHVF) was shown to boost the anti-inflammatory cytokine IL-10. Analysis of this peptide using the IL-10Pred program also confirmed the IL-10-inducing property of this peptide [129].

Although IL-6 and IL-10 are functionally opposed, both are required for a satisfactory prognosis. When IL-6 or STAT3 are deleted genetically, antimicrobial defense in the host is hindered. For example, IL-6 and Stat3 phosphorylation are involved in the transcriptional activation of AMPs in epithelial cells during UTIs originating from uropathogenic *E. coli* (UPEC) through TLR-4 signaling. Thus, IL-6 secretion activates immunity and inhibits intracellular bacterial community (IBC) formation [130]. On the other hand, upon urinary-tract colonization of UPEC, survival, invasion, and biofilm formation, flagellin is synthesized and recognized by TLR-5, which contributes to IL-10 production in the bladder during UTI [131]. Altogether, the contribution of IL-6 and IL-10 in controlling infection should be tightly regulated for the success of treatment outcomes. Likewise, the role of IFN-γ in survival rate, lowering microbial burden, and declining renal pathology is investigated [6]. IFN-γ is involved in moderating the antimicrobial capacity of the host via the STAT1/STAT3 signaling pathway and acts as a protective cytokine towards bacterial and viral infections by triggering AMP release [132]. In addition, some peptides, such as CPP10-859 (KPLKRRVKQY), CPP15-1042 (DAINVRRRRSITRRV), and CPP15-1043 (AINVRRRRSITRRVS), are predicted AMPs with a high potency of APC induction. Some AMPs, such as CPP15-1029 (RDAINFRWRRSLIRR), cryptic in *C. pennaceus conotoxin* (*Q9BP63*), are wide-spectrum immunomodulatory AMPs. CPP15-1029 can simultaneously target Gram-positive, and Gram-negative bacteria, viral pathogens, and *Mycobacterium tuberculosis*, as well as APC induction. CPP10-946 (KKGRKNLWRR) is an AMP active against viral and tubercular infections with a high BBB permeability. This peptide is also an APC stimulant suitable for encephalitis-immunoadjuvant design. Therefore, these candidates are optimistic peptides for the development of universal adjuvants.

Taken together, the pleiotropic function of cytokines should be considered carefully to raise the effectiveness of therapy and deter related complications. It is noteworthy that the actual function of an AMP relies on the whole regulatory impact on the immune system and not only on a single factor. If stimulating the pro-inflammatory cytokine induction was realized to be disfavored, AMPs with anti-inflammatory characteristics should be consumed.

#### 2.8.2. Antibacterial Peptides

Currently, the “ESKAPE” class of pathogens is of special concern as they can evade standard treatments via antibiotic-resistance strategies [133]. Within this study, we have determined AMPs against three members (*S. aureus*, *K. pneumonia*, and *P. aeruginosa*) of the ESKAPE family with a MIC lower than 25 µg/mL. In addition, specific AMPs against *E. coli* were defined. WLBU2 (RRWVRRVRRWVRRVVRVVRRWVRR) and LL37 (LLGDFFRKSKEKIGKEFKRIVQRIKDFLRNLVPRTES) that are experimentally validated to be active against the ESKAPE family were used as the positive control [134]. The DBAASP program predicted that both peptides are active against *P. aeruginosa* and *E. coli*, and WLBU2 was also predicted to be active against *S. aureus*.

**Figure 5:**
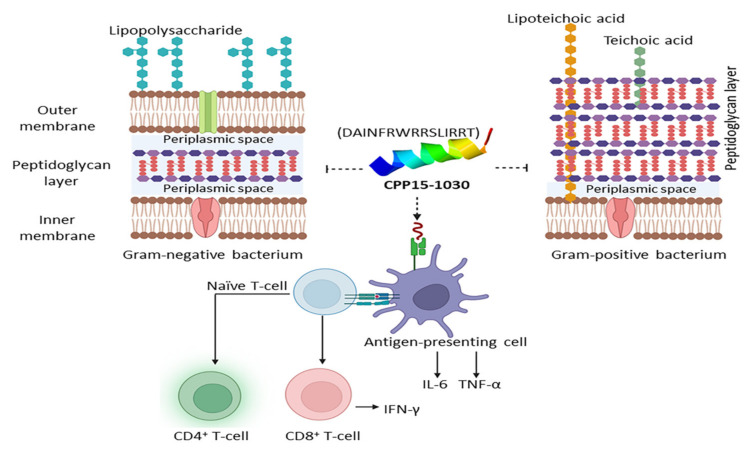
A schematic representation of a multi-target peptide to cover the pitfall of conventional antibiotics. CPP15-1030 is a broad-spectrum AMP with binding energies of −4.9 and −5.2 Kcal/mol to the Gram-positive and Gram-negative bacterial membranes. It exerts its effects both by targeting the bacterial membrane and stimulating antigen-presenting cells to secrete cytokines and chemokines and activating naïve T-cells to recruit adaptive immunity.

*S. aureus*, a normal microbiota of skin, is a Gram-positive bacterium that can provoke infections in wounds or soft tissues with a challenging therapy due to antibiotic resistance. Out of 279 CPPs, 41 peptides were predicted to be effective against *S. aureus* with a MIC lower than 25 µg/mL (Table S3). A total of 16 peptides were predicted to be AMP with antistaphylococcal properties using CAMP_R3_ and DBAASP, respectively (Table S3). Seven peptides, including CPP10-297, CPP10-299, CPP15-81, CPP15-1029, CPP15-1030, CPP15-1032, and CPP15-1043, were safe and showed proper physiochemical properties to be used against *S. aureus*. CPP15-81 (KLQATIAKKLFAIRS) seems to be an appropriate antistaphylococcal candidate as it had the highest affinity to the Gram-positive membrane with a binding energy of −6.1 Kcal/mol using FMAP. Except for CPP10-299 and CPP15-1032, the other five candidates had a higher affinity to the plasma membrane of the Gram-positive bacteria than the human membrane (DOPC), which lowers their side effects on the host cells. CPP10-297 (LQRTIAKKLL) derived from *D. zebra* toxin (A0A068BD83), with a binding affinity of −4.6 Kcal/mol to the Gram-positive bacterial membrane, shows to be a proinflammatory peptide by induction of IL-6, TNF-α and is appropriate to be applied in the acute phase of infection. Due to the low affinity of CPP15-1043 (AINVRRRRSITRRVS) derived from *C. pennaceus* conotoxin (Q9BP64) to the bacterial membrane (−0.6 Kcal/mol), it potentially recruits other mechanisms to combat bacterial infection. It has a Boman index of 5.72 Kcal/mol and is a moderate inducer of IL-4, IL-6, TNF-α, and IFN-γ with a strong ability to activate APC (VaxinPAD score: 1.13). Hence, CPP15-1043 can stimulate both innate and active immunity and is applicable as an immunoadjuvant in vaccine design against *S. aureus*. CPP15-1029 (RDAINFRWRRSLIRR) and CPP15-1030 (DAINFRWRRSLIRRT), isomers derived from *C. pennaceus* conotoxin (Q9BP63), show a high affinity with binding energies of about −4.4 and −4.9 Kcal/mol to the bacterial membrane with moderate immunostimulatory properties. CPP15-1030 also is an immunoadjuvant triggering APCs, which results in T cell maturation and expanding adaptive immunity (Figure 5).

*K. pneumonia* is a non-motile Gram-negative encapsulated bacillus colonizing on the mucosa. It is known as the etiology of dominant cases of hospital-acquired pneumonia, especially in immunocompromised patients. A synergistic effect of an antibiofilm peptide called DJK-6 and meropenem has been observed against *Klebsiella* species [135]. A total of 160 peptides were predicted to be active against *K. pneumonia* using DBAASP, 35 of which were also predicted to be AMPs, according to the CAMPR3 program (Table S3). Computing the intersection of cytokine-inducing peptides and 35 anti-Klebsiella peptides resulted in eight candidates, seven of which had proper physiochemical properties. CPP15-195, CPP15-1050, CPP20-48, and CPP20-557 were agonists of APC receptors. CPP15-1050 showed the highest potential to trigger APCs. Other optimal candidates were CPP20-45, CPP20-51, and CPP20-156, active against *K. pneumonia*. CPP20-156 (RAKINLLSKRKPPAERWWRW) showed the highest affinity to the inner membrane of this bacterium with a binding energy of about −6.9 Kcal/mol. Trp appears to be the aromatic activator of Arg-rich AMPs by ion pair-π interactions to facilitate connection with the membrane [136]. A similar observation has been reported for indolicidin and triptrpticin as Trp- and Arg-containing peptides [137]. As this peptide is predicted to be a potent IFN-γ inducing peptide, it acts by targeting the pathogen with simultaneous immunomodulation.

*P. aeruginosa*, with antibiotic-resistance mechanisms, is a rod-shaped non-spore forming Gram-negative bacteria. It is a major opportunistic pathogen in severe burn wounds and nosocomial infections, especially in immunocompromised patients with cancer, diabetes, organ transplant, and ICU admissions [117]. A total of 23 peptides were active against *P. aeruginosa* according to DBAASP, eight of which were also in common with the prediction of CAMPR3. Four peptides, including CPP10-297, CPP15-81, CPP15-144, and CPP15-1032 had proper physiochemical properties. CPP10-297, CPP15-81, and CPP15-144 had a higher affinity to the Gram-negative bacterial membrane than the host mammalian membrane, with binding affinities of −4.3, −5.5, and −5.0 Kcal/mol, respectively. All three peptides are moderate immunostimulators by induction of IL-6, TNF-α, and IFN-γ to be applied in the acute phase infection. CPP15-81 as an inducer of APCs can also drive adaptive immunity activation.

Although it is considered part of the microbiome, *E. coli* is one of the etiologies of recurrent UTI. Approximately half of the universal antibiotic resistance is attributed to *E. coli* infections. It secretes an exopolysaccharide known as Colanic acid promoting biofilm formation under harsh conditions exposure. A total of 100 peptides were predicted to be active against *E. coli*, 37 of which were in common according to the prediction of CAMPR3. The intersection of these 37 Anti *E. coli* peptides with cytokine-inducing peptides resulted in the final 10 candidates. In sum, eight peptides, including CPP15-195, CPP15-387, CPP15-1050, CPP20-45, CPP20-51, CPP20-155, CPP20-156, and CPP20-557 had proper physiochemical properties. Although all these candidates are immunomodulatory, CPP20-156 (RAKINLLSKRKPPAERWWRW) had a high affinity to the Gram-negative bacterial membrane with a binding affinity of −6.9 Kcal/mol and seemed to be an appropriate candidate against *E. coli* that targeted the microorganism and modulates the immune system simultaneously. In addition, amongst inducers of APCs, CPP10-859, CPP15-825, and CPP15-1050 were also active against *E. coli* and suitable to trigger adaptive immunity.

#### 2.8.3. Antitubercular Peptides

Tuberculosis (TB), an airborne spreading infectious disease, AIDS, and malaria are termed the “Big Three” infectious disorders [138,139]. TB mainly arises from *Mycobacterium tuberculosis*, with a high mortality rate in low/middle-income countries, especially in immunocompromised patients. However, the emergence of multidrug-resistant *M. tuberculosis* towards rifampicin and isoniazid as the first line antitubercular medications is a great concern of healthcare authorities. *M. tuberculosis* enters into alveolar macrophages by phagocytosis and attacks the interstitial lung tissue and dendritic cells, stimulating the secretion of various chemokines, cytokines, and the recruitment of immune cells. The intracellular (intra-macrophagic) lifestyle of Mycobacteria necessitates shifting drug-discovery agendas towards more accurate *in cellulo* investigations on infected host cells using cell-penetrating antitubercular peptides [140]. Mycobacteria have a waxy and highly hydrophobic complex cell wall. Peptidoglycan layers formed by N-glycolylmuramic acid are fused to a layer of arabinogalactan through a phosphodiester bridge. The outer wall layer contains long-chain fatty acids known as mycolic acids that are covalently conjugated to arabinogalactan, responsible for high hydrophobicity and decreased drug permeability [141]. The presence of inflammatory cytokines, such as TNF-α and IFN-γ, in the early stages of tubercular infection is vital. It has been verified that in the prolonged presence of IL-10 and Th1 responses toward mycobacterium bacilli are reduced and extend the latent tuberculosis infection [142].

A total of 150 antitubercular peptides were identified in this study, and 90 of them showed proper physiochemical properties. The most potent peptides were CPP20-156 (RAKINLLSKRKPPAERWWRW), CPP10-1657 (RDAINFRWRR), and CPP10-757 (VRWYRNGTCR). We also tried to define antitubercular candidates that are inducers of TNF-α and IFN-γ and non-inducers of IL-10. Out of the 32 candidates, 26 had proper physiochemical properties (Table S3). CPP20-563 (KQAMQRDAINVRRRRSITRR) in *C. pennaceus* conotoxin (Q9BP64), CPP15-1027 (TQRDAINFRWRRSLI) derived from *C. pennaceus* conotoxin (Q9BP63), and CPP10-1383 (RDAINVRRRR) from *C. tessulatus* were the most potent immunomodulatory-antitubercular candidates. Interestingly, CPP10-1383 and CPP20-563 showed strong immunoadjuvant properties and were proposed to be applied as an adjuvant in the construct of antitubercular vaccines. These candidates can be formulated as aerosolized peptides for intrapulmonary delivery. A highly hydrophobic peptide called VpAmp1.0, derived from the scorpion venom, which is experimentally validated to be active against an MDR strain of *M. tuberculosis* [143], shows antitubercular activity with an SVM score of 0.99 using AntiTbPred program. LL-37, which shows both pro-inflammatory and immunosuppressing effects in a time-dependent manner against Mycobacteria, is predicted to have antitubercular activity with an SVM score of 0.781. This reveals that the introduced AntiTB candidates in this study have a higher potential than a human HDP.

A poorly prognosed type of meningitis known as tuberculous/ tubercular meningitis is an extrapulmonary *M. tuberculosis* infection. Therefore, AntiTB peptides that can pass the BBB are highly valuable in targeting tuberculous meningitis [144]. CPP10-757 (VRWYRNGTCR) derived from *C. chanos* stonustoxin (A0A6J2VWA1) is a strong antitubercular non-allergic peptide with the ability to pass the BBB suitable for application in tubercular meningitis. AntiTB peptides show a high content of R, W, and L residues. The presence of high hydrophobic residues seems to be useful for interaction with the highly hydrophobic barrier around Mycobacteria. Human β-defensins (hBDs) are upregulated during *M. tuberculosis* infection. A synergistic effect has been observed during the coadministration of HNPs (α-defensins) and hBDs with current antitubercular medications. Taken together, to lower the dose, resistance, and side effects of available drugs, AntiTB peptides are recommended as co-adjuvants in antitubercular therapy [141].

#### 2.8.4. Antifungal Peptides

The increased universality of invasive-fungal infections, such as Candida, Aspergillus, and Cryptococcus, and their high mortality rate is the outcome of growing immunocompromised patients. Long-term hospitalized patients, permanent catheterization, abdominal surgeries, deep burns, diabetes, dialysis, mechanical ventilation, and premature infants are prone to fungal contamination [145]. Excessive or incomplete consumption of antifungal drugs has developed drug resistance for commercially known antifungal medications, especially the candidiasis drug of choice, fluconazole [146]. On the other hand, few mechanisms diversity and high side effects of current antifungal drugs necessitate developing novel, with safe antifungal agents with higher efficacy acting through multiple mechanisms.

Antifungal peptides (AFPs) might interfere with chitin or 1,3-β-glucan synthesis or induce cell lysis by targeting the negatively charged fungal membrane. They might also interfere with DNA, RNA, and protein synthesis or induce apoptosis. Many AFPs interfere with fungal components, such as phosphorylceramide, mannosyldiinositol, glucosylceramide, β-glucan synthesis, or ergosterol biosynthesis-related enzymes. Hence, AFPs display higher selectivity toward fungi with diminished side effects for the host [147]. Echinocandins, such as Caspofungin, are examples of FDA-approved lipopeptides that exert their function by inhibiting fungal cell-wall glucan synthesis. Amphotericin B and azoles target the fungal membrane.

Analysis of the toxin-derived peptides to define AFPs using the Antifp program showed that among the 168 predicted AFPs, CPP10-858 (EKPLKRRVKQ), a conotoxin-derived peptide from *C. betulinus* (B0KZ78) with a hydropathicity of −2.13, has the strongest antifungal activity. Interestingly, this peptide has oppositely charged pairs at i (GLu^1^) and i + 4 (Lys^4^) positions on the same face of the helix to construct a salt bridge. VaxinPAD analysis shows that CPP10-858 has a strong immunoadjuvant property. CPP15-5 (HSMKYRLSFCRKTCG), CPP10-1032 (DCRFNRGRCR), and CPP10-352 (CKTEWIKSKC), all with the potential to form disulfide bonds, are the next potent antifungal peptides, according to Antifp program. All three candidates contain at least one disulfide bond. Disulfide-containing peptides are more structured than linear ones and display higher stability. AFPs are usually rich in residues, such as Cys, Arg, Lys, and His. A chitin-binding motif at inter-cysteine loops and aromatic residues is required for chitin binding and antifungal activity in hevein-like peptides [148]. Human β-defensins (hBD2 and 3) are examples of Cys-rich peptides with three disulfide bonds that eradicate *C. albicans* by membrane disruption [149]. CPP15-5 and CPP10-1032 are potent antifungal peptide with BBB permeability applicable for fungal meningitis and meningo-encephalitis. A total of 10 peptides were predicted to be active against *C. albicans* with a MIC lower than 25 µg/mL according to DBAASP, two of which (CPP10-11 and CPP10-963) had proper physiochemical properties. CPP10-11 (MKYRLSFCRK) derived from *S. haddoni* κ-stichotoxin (E2S062) was also predicted to be AFP, according to the AntiFP program. To dominate fungal-antibiotic resistance, the vision should turn towards compounds that are effective not only on the fungus itself but also on the immune system. HBD and cathelicidin in the skin and histatins in the oral cavity are examples of HDPs for such antifungal activity [149]. CPP10-11 is also able to trigger APCs and recruit adaptive immune cells. A synergistic effect has been reported in the combination therapy with fluconazole and a disulfide-cyclized cationic lipopeptide against Candidia [150]. Therefore, similar to the synergistic effect observed in combination therapy with AMPs mentioned earlier, combinatorial therapy of currently available antifungal antibiotics with AFPs can decrease the concentration of each component in the therapeutic regimen, which in turn decreases the drug side effects and resistance.

#### 2.8.5. Antiviral Peptides

While HIV and influenza are examples of longstanding threats to humans, emerging viral infections, such as coronavirus, compelled huge life losses. AMPs can destabilize the membrane of enveloped RNA or DNA viruses by various mechanisms. Antiviral peptides (AVPs) might inhibit viral proteases, integrase, reverse transcriptase, DNA transport to the host nucleus, or viral prefusion [151]. Since viruses are intracellular pathogens, AVPs with cell permeation ability are highly valued to target the intracellular parasite [152,153]. The meta-iAVP program that outperforms several other AVP predictors regarding accuracy was used to discriminate between viral and non-viral peptides [154]. According to the meta-iAVP, a total of 108 peptides were predicted to have antiviral properties 61 with proper physiochemical characteristics. CPP20-156 (RAKINLLSKRKPPAERWWRW) was the most potent antiviral peptide defined by meta-iAVP and shows an IC_50_ value of about 7.39 µM against the respiratory-syncytial virus (RSV), influenza virus (INFV), and herpes simplex virus (HSV). This candidate targets the virus itself and shows immunomodulatory properties by triggering the production of IL-6, INF-γ, IL-10, and TNF-α. Like other AMPs, the effect of AVPs on the immune system might seem paradoxical; however, the final effect should favor pathogen eradication and host-tissue protection from inflammatory damage. TLR4 and G-protein coupled receptors typically detect bacterial peptidoglycan, lipopeptides, and viral envelop glycoproteins, resulting in IL-1β, IL-6, INF-γ, IL-10, and TNF-α secretion [155]. LL-37, which is known to be active against various viral infections, such as HSV, DENV, RSV, and IAV (H1N1) [155], showed to be AVP using Meta-iAVP with a score of about 0.742. Because patients suffering from viral contamination frequently acquire a secondary bacterial infection later, peptides with dual antibacterial and antiviral characteristics are beneficial to target both pathogens simultaneously [156]. Therefore, CPP20-156, which is shown to have a high affinity toward bacterial membranes with antibiofilm properties, is of great value. CPP20-51 (RRFERYQQVLCNKGLSRRHY) is another strong immunomodulator antiviral candidate, which also displays an IC_50_ of about 5.52 µM toward HIV. CPP20-184 (VERAGENRSKENIKFLLKRK) with an IC_50_ of about 10.08 µM against HCV shows immunomodulatory properties and is verified as an antiviral peptide, according to meta-iAVP. HNP-1, which is known to inhibit HSV and influenza virus [127], shows IC_50_ values of about 110.14 µM according to the RF model of AVP-IC_50_Pred. This confirms that the introduced antiviral peptides in this study are much more potent than the HDPs. According to the intersection of AVPs determined by MetaiAVP and APC-inducing epitopes CPP15-44 (KKQLIRRHYWEIKWS) from *P. volitans* Pvtoxin, CPP20-332 (ARRKLMKVLRESECPWKPWC) from *Conus monile* contryphan and CPP20-426 (KQATQRDAINVRRRRSITRR) from *C. tessulatus* conotoxin are proper universal adjuvants in the formulation of vaccines designed for viral pathogens. CPP10-582 (RNRFVRIGRR) from *C. textile* conorfamide and CPP10-936 (LQILLNKRPC) from *C. betulinus* conotoxin are antiHIV and antiHCV peptides, respectively, with immunoadjuvant properties. Taken together, these candidates are not only able to target the virus but also induce APCs stimulating cytokine secretion and maturation of T cells.

#### 2.8.6. Antibiofilm Peptides

The concept of a unicellular, freely swimming bacterial lifestyle known as the planktonic phase is mainly in axenic-suspension-culture media. The predominant phase of bacterial growth in nature is observed as heterogeneous-adhesive communities of matrix-encased polymicrobial cells collectively known as biofilms as a survival strategy [157]. The extracellular matrix (ECM) of biofilms is a mixture of exopolysaccharides, such as glycolipids, glycoproteins, lipopolysaccharides, and nucleic acids. The scattered microcolonies in a biofilm receive varied nutrients and oxygen in distinct biofilm zones. The exterior layer with metabolically active cells and the deeper slow-growing layers show phenotypic variations and different antibiotic sensitivity [158]. Communication of microbial cells in a biofilm is adopted through pili or quorum-sensing signaling molecules. Biofilms are depicted as an adaptively recalcitrant growth condition of bacteria towards stressors, such as the host immune system and antibiotics. Populations within a biofilm phase might be a thousand times more unsusceptible to antibiotics than their planktonic growing state, creating huge limitations in surface sterilization of industrial settings, medical devices, and biofilm-born infections. About 65% of chronic infections, such as cystic fibrosis pneumonia, endocarditis, and dental caries, are correlated with biofilm formation [159].

**Figure 6:**
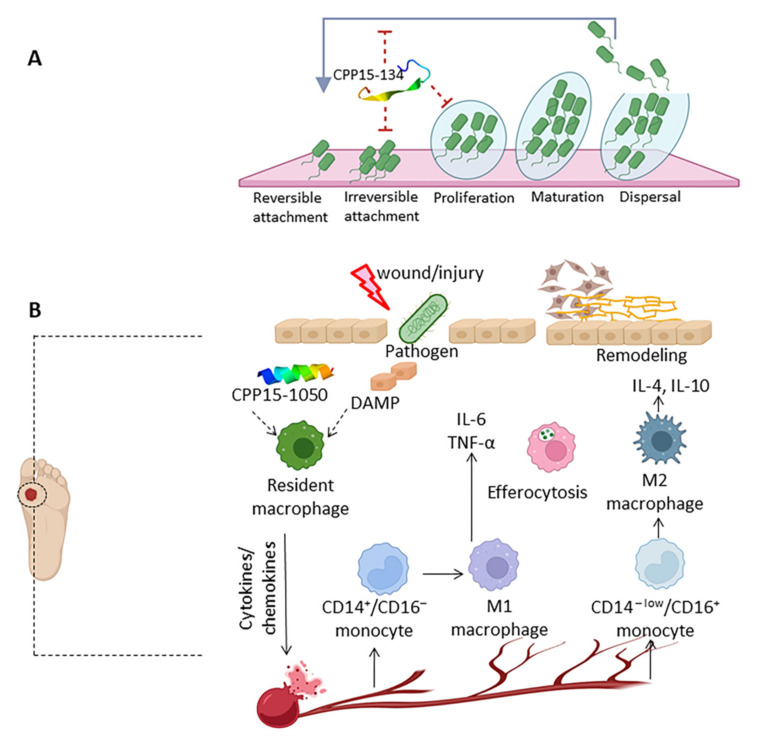
Schematic representations of biofilm formation and wound healing: (**A**) CPP15-134 is an antibiofilm-penetrating peptide able to disrupt or interfere at any stages of biofilm formation; (**B**) Recruitment of monocytes and macrophages in the pro-inflammation phase of wound healing is derived from de-granulated platelets in blood clots at injury site as the first source of cytokines and growth factors. Receptors on the surface of resident macrophages in the tissue detect DAMPs and peptides, such as CPP15-1050, resulting in cytokine secretion and recruitment of monocytes and neutrophils to phagocyte the pathogen and cell debris for wound cleansing. Infiltrated CD14^+^/CD16^−^ pro-inflammatory monocytes are polarized to M1 macrophages as a source of IL-6 and TNF-α. Anti-inflammatory CD14^−low^/CD16^+^ monocytes are polarized to M2 macrophages as a source of IL-10 and IL-4 for epithelialization, remodeling, wound repair, and neovascularization.

Antibiofilm peptides might interfere in any stage of biofilm developmental phases, including planktonic-cell attachment to a substratum, matrix secretion, biofilm expansion and development, and bacterial-cell release through desorption, dispersion, or detachment [160]. Therefore, despite being AMPs, antibiofilm peptides target biofilm formation and structure rather than bacteria (Figure 6A). About 56% of non-toxic CPPs were identified as potentially biofilm-active peptides. Since ESKAPE microbial pathogens are involved in nosocomial infections, we tried to select antibiofilm candidates with a broader spectrum. The most potent candidate was defined to be CPP15-134 (KRQCRGVRVTRRSLR), with an antibiofilm score of about 1.5. This Arg-rich cationic antibiofilm peptide with a total net charge of +7 and good water solubility (GRAVY: −1.44) is predicted to be active against *S. aureus*, *K. pneumonia*, and *E. coli*. About 95% of the extracellular polymer substance (EPS) in the biofilm is composed of water. The negatively charged matrix covering the biofilm is able to interact with cationic-antibiofilm peptides and distribute them across the biofilm [161]. CPP10-297 (LQRTIAKKLL), derived from *D. zebra* toxin (A0A068BD83), seems to be an amphipathic-biofilm-active peptide against *S. aureus*, *P. aeruginosa*, *K. pneumonia*, *E. coli*, and *M. tuberculosis.* Positive controls, including synthetic peptides 1037 and 1018, piscidin 3, and LL-37, were predicted as antibiofilm peptides by the dPABBS program. A synergistic effect of antibiofilm peptides and conventional antibiotics, includingciprofloxacin, with mechanisms, such as increasing cell penetration in abscess infections, has been reported [162]. Cell-penetration ability of a peptide might be more promising for biofilm eradication. A cationic CPP (PEP-NKSM) with antibiofilm activity has been reported to successfully eradicate 55% *Staphylococcus epidermis* biofilm [163]. Notably, a minimum biofilm inhibitory concentration (MBIC) lower than MIC is more appropriate to decrease the probability of antibiotic resistance and dysbiosis [164].

Conclusively, skin and soft tissue infections that are developed by *S. aureus* or *P. aeruginosa* biofilm-forming species are considered high-priority pathogens to be treated with antibiofilm peptides. Similarly, ventilator-born lower-respiratory-system-biofilm-forming pathogens, catheter-originated biofilms in the urinary tract or vascular tissue, dentures, contact lens, biofilm-infected implants, stents, pacemakers, shunts, and orthopedic hardware are candidates for antibiofilm-peptide coating [165].

#### 2.8.7. Wound Healing Peptides

Chronic-wound infections observed in diabetes, severe burns, and venous/arterial disorders are death-threatening infections usually contaminated, with *S. aureus* and *P. aeruginosa* as known pathogens colonizing the wound and forming biofilms. Wound healing is a complicated procedure with overlapping phases. In the inflammation phase, the wound is coated by a matrix and is invaded by circulating immune cells to clear the destroyed tissue and control infection. Then in the proliferation stage, fibroblasts are recruited, and angiogenesis occurs. Finally, epithelialization and tissue remodeling are observed [166].

Proinflammatory cytokines, such as IL-6 and TNF-α produced by M1 macrophages, are involved in the regulation of immune cells in the inflammation phase and cell proliferation in epithelialization [167]. TNF-α, as a paramount element in wound healing, triggers the synthesis of cell-surface-adhesion molecules. This is effective in the adhesion of neutrophils to the endothelium and promotes keratinocyte proliferation. IL-6 is required for neutrophil, macrophage infiltration, and keratinocyte proliferation in wound healing [168]. Only moderate levels of proinflammatory cytokines are required to control the infection. Anti-inflammatory cytokines, such as IL-4 and IL-10 produced by M2 macrophages, are involved in the wound proliferation phase and remodeling. IL-4 induces neovascularization required for wound healing. This cytokine has a direct influence on endothelial cells and is involved in hypoxia-induced angiogenesis. For example, IL-4 can promote choroidal neovascularization to ameliorate age-related macular degeneration. IL-4 polarizes macrophages to the M2a phenotype that are able to express angiogenic elements like VEGF-A, TGFα, and PDGF-β to facilitate neovascularization and wound healing. Similarly, some evidence argues that because IL-10 can polarize the macrophage into an M2c phenotype, it might be beneficial in the angiogenic process of wound healing [169].

Wound contamination with pathogens can result in afflictions, such as limb loss. AMPs have a prominent role in wound healing through various mechanisms. They promote the polarization of M0 macrophages to either M1 or M2 phenotypes, activate receptor signaling mechanisms to elevate cell proliferation, promote re-epithelization, upregulate angiogenic proteins, and contract fibroblasts (Figure 6B) [170]. Conjugation of wound-healing peptides to CPPs has been demonstrated to improve keratinocyte-drug delivery and wound healing [171]. In this study, to define peptide candidates for wound healing, the intersection of IL-6, TNF-α, IL-4, and IL-10 inducers with 52 AMPs and biofilm-active peptides showed six candidates, four of which were the most optimal. These wound healing peptides were CPP20-45 (KRQCRGVRVTRRSLREFSHF), CPP20-184 (VERAGENRSKENIKFLLKRK), CPP20-51 (RRFERYQQVLCNKGLSRRHY), and CPP15-1050 (NRLKENIKFLLKRKT) that are derived from *S. verrucosa*, *C. textile*, *D. zebra*, and *C. pennaceus*. Taken together, penetrating AMPs that are able to disrupt biofilms with the simultaneous ability to recruit immune cells are introduced as promising wound- healing products. Finally, a proper formulation is required to sustain the stability and proper delivery of the healing peptide to the tissue, such as wound dressings, topical hydrogels, ointments, or creams.

### 2.9. Anticancer Peptides

Cancer, with almost 10.0 million worldwide deaths in 2020, is the untuned proliferation of abnormal cells that overspreads circumambient tissue [172]. Cancerous cells amplify proliferative signaling and escape tumor suppressors through genetic (mutation or deletion) and epigenetic mechanisms. Immortal-cancerous cells diffuse through blood or lymphatic circulation, known as metastasis [173]. Expanding drug resistance, unfavorable side effects, and low efficacy are disadvantages of chemotherapy, radiotherapy, and immunotherapy, respectively. There are some advantages for therapeutic peptides over antibodies in cancer therapy. Peptides are less immunogenic, and lower doses of peptides are required for solid tumors. Expressing antibodies in eukaryotic systems is more costly, and due to their large size and high molecular weight, they cannot penetrate the target tissue [174]. Therefore, developing novel anticancer agents, such as ACPs, is in great demand due to low side effects and high selectivity and penetration [1]. Combinatorial therapy of conventional anticancer agents with ACPs might be an effective approach due to the heterogeneous characteristics of tumor cells. Several peptide-based FDA or EMA-approved anticancer drugs are available on the market, including goserelin, histrelin, and leuprolide applicable to treating prostate cancer. Other examples are ixazomib, bortezomib, and carfilzomib in multiple myeloma and mifamortide in osteosarcoma [175].

**Figure 7:**
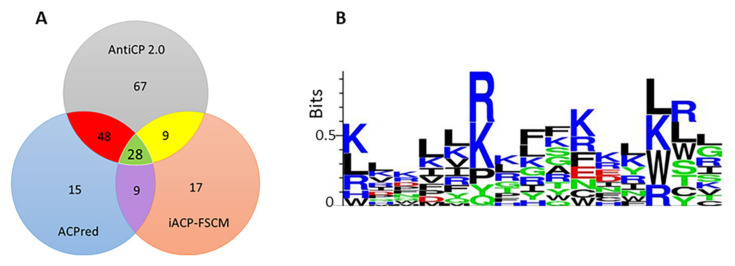
Anticancer peptides and their targets: (**A**) Intersection of predicted ACPs with three programs shows 28 ACPs in common; (**B**) Preferred positions of amino-acid residues represented by Seq2Logo after multiple-sequence alignment for 15-mer ACPs. Lysine, as a cationic residue, is distributed throughout ACPs.

ACPs collected in this study were selected out of candidates that are non-toxic to normal cells and toxic to tumor cells. According to the ACPred, AntiCP 2.0 (model 2 with an SVM threshold higher than 0.45), and iACP-FSCM programs, a total of 99, 152, and 62 peptides were predicted as ACPs, respectively (Table S6). The intersection of all three programs showed that 28 ACPs were in common (Figure 7). A total of 18 peptides that were non-allergic without forming aggregates are collected in Table 5. Positive controls, such as Aurein1.2 (GLFDIIKKIAESF) and Aurein3.1 (GLFDIVKKIAGHIAGSI) isolated from *Litoria aureus* (frog) as well-known ACPs, were designated as strong anticancer peptides by all three applied programs in this study [176]. Knowledge of the characteristics of the TME is guidance to the smart ACP design and delivery. Phosphatidylserine, heparan sulfates, gangliosides, sialic acid, O-glycosylated mucins, and a high abundance of microvilli are the origins of the higher negative charge of the tumor cell surface and enhanced surface area [177]. Various mechanisms, titled barrel-stave, toroidal pore, and carpet models, have been ascribed to the ACPs upon interaction with the membrane. The anionic charge on the tumor-cell surface is appealing to specific targeting and penetration of cationic ACPs. Some ACP candidates are classified as Arg-rich peptides promoting membrane penetration of CPP [178]. ACPs usually have a charge of +2 to +9, which is in line with our observation regarding cryptic ACPs in this study.

Proton secretion through transporters and higher glycolysis result in lactate secretion and extracellular acidification. Hypoxic conditions in tumors due to restricted blood supply exacerbate the acidic status of TME as low as 6.2 in the extracellular milieu compared to the normal tissue with a pH of about 7.4 [179]. CPPs with ACP characteristics found in this study have a pI higher than physiological pH, resulting in a total positive net charge and promoting ACP-tumor cell interaction. It should be noted that other factors, such as peptide concentration, peptide-lipid ratio, and environmental factors, affect the conformation of peptides and membrane permeability, which alters the response. Amino-acid-composition analysis of 28 ACPs shows that the cationic residue Lys is dominantly distributed in various peptide regions. According to the generated logo, hydrophobic residues, such as Leu and aromatic residues, Phe and Trp, are also dominant in the ACP candidates (Figure 7).

About 28% of our ACPs displayed either primary or secondary cationic-amphipathic characteristics with both polar and nonpolar faces suitable for interaction with the hydrophobic regions of the membrane. The hydrophobicity of these candidates varied between 10–70%; however, increasing hydrophobicity decreases the solubility of the peptide [1]. The conformation of a peptide is also critical for its biological function. Although peptides can adopt β-sheet or coil structure, the dominant conformation of ACPs is recognized as α-helix. This conformation is especially critical upon interaction with the membrane. We have defined the interaction of candidate ACPs and a DOPC bilayer with zwitterionic characteristics of the mammalian membranes using the FMAP program. CPP10-421 was an ACP with the highest affinity to both cell and mitochondrial membranes (Table 5). Most ACPs showed a helical segment in their conformation after interaction with the membrane, wherein CPP10-421 showed the highest helical content (Table 5). ACPs with mitochondrial targeting showed helical regions in their structure upon association with the mitochondrial membrane.

Complex pathological diseases, such as cancer, must be targeted by multipronged curative. Sometimes, single-molecule polypharmacology has been translated to drug conjugates, fused, or merged hybrids in one formulation [180]. However, polypharmacology is defined as a mixture of multiple drugs or a single therapeutic modulating numerous targets. Conjugation might not always be favorable due to the necessity for activated functional groups participating in the reaction and requires a cleavable linker at the target site [181]. Perturbing tumor-membrane integrity via necrosis or apoptosis, interfering protein-protein interaction, inhibiting angiogenesis, activating immune cells, and inducing cytokine secretion are some ACP mechanisms to eradicate cancer cells. For example, lenalidomide, a chemotherapeutic agent used to treat multiple myeloma, is one of the best examples of single-molecule polypharmacology. Lenalidomide, as a multi-target drug, has multiple modes of action, such as immunomodulation, anti-angiogenesis, and apoptosis induction. Therefore, we tried to shed light on the potential mechanisms of the identified ACPs within this study in the subsequent subsections.

#### 2.9.1. Apoptosis and Necrosis Inducing Peptides

Differences between mitochondria in normal and tumor cells regarding structure and function make this organelle an appropriate target for anti-tumor drug design and delivery [182]. ACPs preferentially disrupt the integrity of mitochondrial membranes at concentrations 100 times lower than the concentrations needed to disrupt the integrity of tumor cells’ membranes. The priority originates from the difference in membrane potential provoked by proton pumps during mitochondrial-inner-membrane-oxidative phosphorylation [183]. The membrane potential forces cationic peptides into the mitochondria, especially in cancerous cells, as the potential persistently ratchets up and supplies a greater killing capability in TME [184]. For example, TLSGAFELSRDK peptide is an mtCPP both targeting intrinsic-apoptotic pathway and delivering chemotherapeutics ligands to the mitochondria [185]. Peptides containing mitochondrial-targeting domain can trigger mitochondrial Ca leakage and cell death [186]. Notably, some ligands might interact with cell surface death receptors, such as TNF-receptor type 1 and Fas-associated death domain, to induce caspase 8 and activate the extrinsic-apoptotic pathway [182]. ACPs might target the open and close conformation of voltage-dependent anion channels (VDACs) in the outer membrane of mitochondria as a pore-forming protein which leads to the mitochondrial-solute flux and apoptosis [187]. The conjugation of mitochondrial targeting signals probably is insufficient and their conjugation to a CPP is more effective. Conjugation of a poly-Arg CPP known as R7 to (KLAKLAK)2 as a mitochondrial-disrupting peptide triggers tumor apoptosis [188]. To define if the identified ACPs in this study are acting through mitochondria, mtCPPs were characterized using the TargetP-2.0 program. (KLAKLAK)_2_, a distinguished cytotoxic mtCPP disrupting the mitochondrial membrane as the positive control and verified as a strong ACP, and a mitochondrial-transfer peptide using AntiCP 2.0 and TragetP-2.0, respectively. As shown in Table 5, four ACPs were able to target the mitochondrial membrane, including CPP 10-421 (ILLPALRKFC), CPP10-186 (KAYLFRNLAK), CPP10-299 (TIAKKLLSIR), and CPP15-144 (KLNLQRTIAKKLLSI). Some ACPs, such as CPP10-421 with a helical structure, were mtCPP and showed a higher binding affinity to the mitochondrial membrane than the cell membrane.

Notably, cell death can be extensively classified into non-inflammatory and proinflammatory types. Apoptosis is normally recognized to be non-inflammatory without rendering collateral injury to adjacent cells. Necrosis leads to inflammatory responses, vicinal cell destruction, and removal of neoplastic-cell populations, which prevent metastasis and are beneficial to eradicating aberrant surrounding cells which have not been triggered for apoptosis [189]. Some ACPs, such as CPP20-48 (KKICNDYKLNLQRTIAKKLL), is a proinflammatory-inducing peptide that triggers IL-6 and INF-γ and potentially exacerbate necrosis by a feedback mechanism. chMAP28 (GRFKRFRKKLKRLWHKVGPFVGPILHY) is known as a broad-spectrum necrotic factor for cancerous cells. We have used this peptide to evaluate its proinflmmatory characteristics [190]. It showed IL6 and INF-γ inducing characteristics similar to our CPP20-48. It was neither targeting mitochondria nor an antiangiogenic peptide, according to our analysis. However, some peptides exert their effect in a concentration-dependent manner. For example, hepcidin from tilapia fish is necrotic or apoptotic at high and low concentrations, respectively [191].

**Figure 8:**
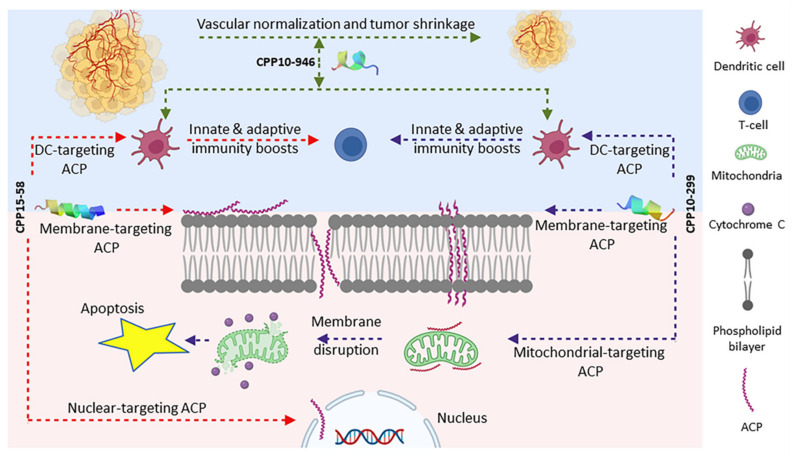
Representation of anticancer peptides’ (ACPs) interaction with their targets. **CPP15-58** can interact with the tumor-cell membrane (binding energy −3.0 Kcal/mol), partially targets the nucleus, and induces antigen-presenting cells (APCs), such as dendritic cells (DCs) to recruit immune cells to the tumor site. **CPP10-299** targets the tumor-cell membrane (binding energy −3.7 Kcal/mol) and stimulates APC. It targets the mitochondrial membrane (binding energy −3.4 Kcal/mol) to induce apoptosis. **CPP10-946** has a lower binding affinity to the membrane (−1.9 Kcal/mol), but it has antiangiogenic characteristics and induces DCs to retrieve adaptive immunity.

ACPs that are able to localize inside the nucleus might exert their anticancer effect by interfering with DNA synthesis or proteins involved in cell division. A few ACPs in this study showed sole-nuclear accumulation. The helical-cationic peptide CPP15-58 (LKKKLKTFQKHYERL) derived from *Scorpaena plumieri* is an ACP with a partial-nuclear- targeting property, according to the cNLSMapper prediction score. CPP15-29 (KPKKLRPSTKDYWYI) achieved from Pvtoxin of *P. volitans* exclusively localizes in the nucleus with moderate ACP activity. Altogether, CPPs with ACP predicted function, which have innate potency of targeting mitochondria, nucleus, or inducing proinflammation, provide an exciting perspective of apoptosis, necrosis, or anoikis-inducing mechanisms that require experimental investigations (Figure 8).

#### 2.9.2. Antiangiogenic Peptides

The construction of blood vessels from endothelial cells and the development of new blood vessels are called vasculogenesis and angiogenesis, respectively. This process is controlled by proangiogenic (VEGF, PDGF, FGF) and antiangiogenic (endostatin, angiostatin, PF4, TSP-1) regulators. Vascular endothelial growth factor (VEGF), especially VEGF-A_165_ isoform, as a proangiogenic signal is indispensable for vascular expansion and homeostasis [192]. VEGF-A ligand mainly interacts with the VEGF receptor 2 (R2). It triggers intrinsic kinase activity by phosphorylation of the intracellular tyrosine kinase domain of the receptor, which in turn recruits downstream-signaling pathways and angiogenesis promotion [193].

In cancer, diabetic/hypertensive retinopathy, age-related macular degeneration (AMD), and increased amounts of VEGF can shape pathological vessels via angiogenesis [192]. Despite the role of the vasculature in wound healing, angiogenesis in TME promotes solid-tumor progression and metastasis. VEGF confers immune escape by persuading an immunosuppressive TME. This effect is manifested by elevating intra-tumoral Treg and MDSC, hindering DC maturation and up-regulation of immune checkpoints. Hypoxic conditions, low pH, and proinflammatory cytokines (IL6) are some of the factors involved in the fibrotic and extravasation (leakage of fluid and blood cells) environment that leads to the enhanced permeability and retention (EPR) effect [77]. The consequence is the entrance of therapeutics into the interstitial-tumor space and drug-delivery failure. Due to the role of diverse proangiogenic factors, screening for novel antiangiogenic peptides is required. Inhibitors of the VEGF-VEGFR angiogenesis signaling pathway with FDA approval have been developed in recent years. An anti-VEGF monoclonal antibody called bevacizumab inhibits VEGF-A binding to its receptor. Sorafenib and sunitinib are VEGFR tyrosine kinase inhibitors [194].

In our study, a total of 211 peptides showed putative antiangiogenic properties, 145 of which were non-allergen without forming aggregates. CPP10-1743 (SCWQRYPERC) from *Conus coronatus* conotoxin, CPP10-1389 (RRRRSITRRG) in *C. tessulatus* conotoxin, and CPP15-1020 (SKRKPSAERWRRDCT) from *C. pennaceus* γ-conotoxin (P56711) are some of the most potent antiangiogenic candidates. Cysteine, which forms disulfide bonds upon oxidation, stabilizes the 3D structure and is the residue of choice in antiangiogenic peptides. Antiangiogenic cysteine-containing peptides play a vital function in restricting vessel proliferation. They act through the activation of angiostatin, improving anti-angiogenesis through lessening receptors for proangiogenic factors, rendering apoptosis, and balancing conflicting signals in the TME [195]. For example, endostatin, an internal antiangiogenic protein, has two disulfide bonds. It has been shown that secreted protein, which is acidic and rich in cysteine (SPARK), is able to inhibit both ovarian metastasis and the progression of breast cancer [196]. FSEC, as a positive control with the consensus sequence “CELDENNTPMC”, is shown to be antiangiogenic using the TargetAntiAngio program. FSEC is a member of the SPARK family with a disulfide bond that inhibits angiogenesis and tumor growth experimentally [196]. Interestingly, the most potent antiangiogenic peptides, such as CPP10-1344 (SKWCRDHSRC) from *Conus kinoshitai* conotoxin and CPP10-1743, harbor at least one disulfide bond with high stability. CPP10-1537 (CSKICWRPRC) and CPP10-352 (CKTEWIKSKC) are other antiangiogenic peptides that can be completely cyclic due to the presence of Cys residues at the N and C terminal of the peptide. Arg is another residue of choice in antiangiogenic peptides [195]. Hence, arg-rich peptides, such as CPP10-1389, are strong antiangiogenic candidates. Table 5 shows nine antiangiogenic ACPs wherein CPP10-1389 had the highest antiangiogenic potential. This peptide showed a moderate BBB permeation ability. CPP10-946 and CPP10-1725 are antiangiogenic ACPs with a significant potential to penetrate CNS. Therefore, they can be considered for developing formulations applicable to CNS tumors.

The retina is a neurovascular tissue consisting of glial cells, neurons, and a network of endothelial cells padded in capillaries. Retinal/neural-derived VEGF is involved in retinal-vascular development and neovascularization [197]. Ocular angiogenesis is observed in retinopathy, AMD, corneal neovascularization, neovascular glaucoma, and some typical blindness reasons. Retinal neovascularization results in hemorrhage and retinal detachment. Currently, ranibizumab, aflibercept, and bevacizumab are administrated for neovessel formation in AMD [198]. Intravitreal injection of antiangiogenic agents impedes serious side effects [199]. Hence, CPPs with antiangiogenic characteristics can be postulated as non-invasive therapy for various intraocular disturbance vascularization. Combinatorial administration of CPPs with bevacizumab has been adopted for targeted macular-drug delivery in AMD [200]. 

Taken together, tumors employ parallel mechanisms to escape immune demolition, such as activating additional angiogenic pathways. A combination therapy containing antiangiogenic agents and other chemotherapy and immunotherapy approaches is more promising than single administration to ameliorate anti-tumor therapy [193]. According to Table 5, antiangiogenic ACPs had parallel mechanisms by targeting tumor-cell membrane or mitochondrial membrane or exerting an immunomodulatory property providing a bright perspective of the success of these candidates in clinical translation (Figure 8).

#### 2.9.3. Immunomodulatory ACPs

Cancer immunotherapy is a multidimensional matrix consisting of tumor cells, TME, and the host-immune system. Devising any therapeutic regimen should consider the dynamic interaction of tumor cells, their niche, and immune pathways. Since sole administration of antibodies targeting immune checkpoints might have harsh immune-related adverse events (irAEs), peptide therapeutics with a shorter pharmacokinetic profile are getting great attention [201] Therefore, the investigation of immunomodulatory ACPs that are not only able to target tumors but also affect the tumor immune microenvironment (TIME) can be a new platform for drug discovery and development. As mentioned earlier, since constitutive activation of cytokines, such as IL-2, impairs effector T cells in TME and tumor relapse, IL-2-inducing peptides are preferred for cancer therapy instead of the direct use of recombinant IL-2. As shown in Table 5, several ACPs with IL-2 inducing potential have been found, such as CPP10-946, CPP10-299, CPP10-1725, and CPP10-1389.

IFN-γ has modulatory roles in TME. Hot tumors with lymphocyte infiltration capacity are the main source of IFN-γ in TME. However, low pH, a hallmark of cancerous tissue, downregulates IFN-γ production by NK cells [202]. Although it depends on the tumor stage and type, it has been demonstrated that immune-checkpoint-blockade inhibitors can increase IFN-γ expression with lower progression and higher survival in non-small cell lung cancer and melanoma [203]. IFN-γ is entailed in the anti-tumor outcomes of cancer radiotherapy and decreases tumor cell growth by various mechanisms, such as cell cycle arrest, necroptosis in solid and liquid apoptotic-resistant tumors, and tumor ischemia. IFN-γ inhibits immunosuppressive cells, such as Tregs, which promotes programmed death-1 (PD-1) targeting immunotherapy. IFN-γ switches tumor-associated macrophages into the M1-phenotype macrophages to suppress VEGF secretion and restrict angiogenesis [69]. On the contrary, IFN-γ might induce tumor progression by downregulatingTNFSF15 in the vascular endothelium and angiogenesis promotion. This interferon might suppress monocytic myeloid-derived suppressor cells (MDSC) and induce programmed death ligand-1 (PD-L1) expression as a tumor protector against immune cells [69]. Therefore, IFN-γ has both protumor and anti-tumor activities and is an important linker in tumor-immunotherapy responsiveness. Since all nucleated cells can react to IFN-γ, the practical impacts of IFN-γ production must be carefully scrutinized depending on the target cell [99]. CPP10-1389 and CPP20-48 are ACPs with significant IFN-γ induction, according to Table 5. Using the PDL1Binder program [204], some ACPs were defined as PDL-1 inhibitors (Table 5). The most potent PDL-1 inhibitor was CPP10-1725 (HIFWSKRNCC) from *C. pennaceus*. PDL-1 is expressed on various malignant tumor cells and APCs. Blocking PDL-1 in the immune-checkpoint pathway restores tumor-T-cell infiltration. Therefore, PDL-1 binding peptides can be considered as neo-adjuvants in cancer immunotherapy. CPP10-1725 has a mixture of coil and extended B-strands; however, after interaction with the DOPC membrane model, helical segments are observed in the structure. 

TNF-α has sometimes been proposed to have anti-tumor effects at high concentrations by promoting tumor infiltration by CTL, which can increase the outcome of immune checkpoint inhibitors. However, a plethora of collected preclinical evidence shows that TNF-α expression, especially at lower amounts, should be assumed as a pro-tumoral cytokine which causes immunotherapy resistance [205].

**Figure 9:**
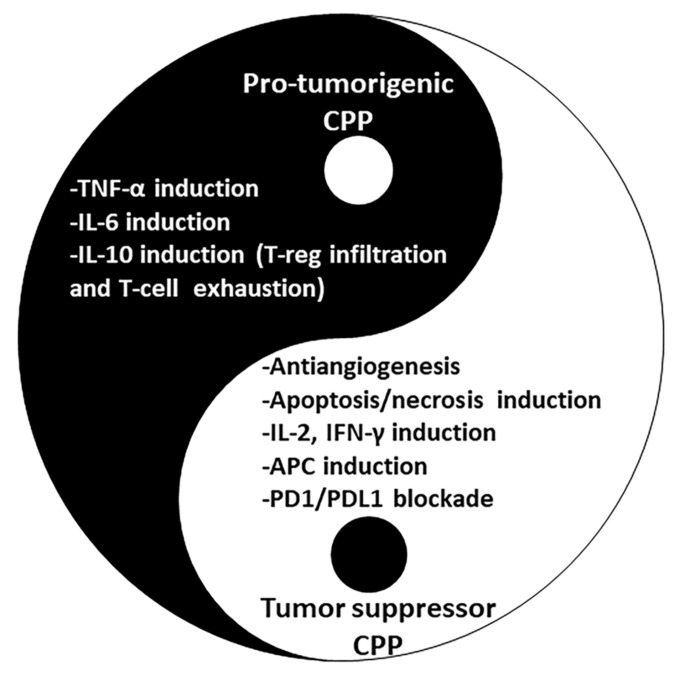
The yin and the yang of CPPs in tumor progression and regression. Peptides that are intrinsically antiangiogenic or induce apoptosis or necrosis, inducers of IL-2 or IFN-γ stimulators of antigen-presenting cells (APCs) to generate mature T-cells, and immune-checkpoint blockers are tumor suppressors. Meanwhile, peptides that are inducers of TNF-α, IL-6, and IL-10 can reduce the outcome of anticancer therapy.

IL-10, as an anti-inflammatory cytokine, might be considered an anti-tumor agent. This cytokine benefits brain tumors, melanoma, and ovarian cancer via MHC-1 down-regulation and tumor-cell-lysis induction by NK cells [206]. Despite its immunosuppressive role, the prevailing opinion rotates around tumor evasion in the presence of IL-10. Infiltration of Tregs in TME and IL-10 production results in the exhaustion of effector T cells [207]. According to the abovementioned roles, CPP10-1389 is one of the optimal immunomodulator ACPs, a strong inducer of IL-2 and IFN-γ, and a non-inducer of TNF-α and IL-10. As observed in Table 5, 12 ACPs display various degrees of APC stimulating effects defined as immunoadjuvants. Epitopes-inducing APCs, such as dendritic cells, prime cytokine secretion, and maturation of naïve T cells to effector T cells, provide the assistance of adaptive immunity in addition to native immunity to eradicate tumors. In this regard, CPP10-1389, CPP15-44, CPP15-58, CPP15-423, and CPP10-946 are ACPs with significant ability to affect tumor- niche-immune system (Figure 8). Altogether, since immunomodulation with anticancer peptides has lower side effects enhancing the therapeutic outcome of currently available chemotherapeutics, they are promising subjects for further in-depth investigation (Figure 9).

#### 2.9.4. Tumor Targeting Peptides

Poor drug penetration to extravascular tissue is a major hindrance in solid-tumor- drug delivery. CPPs enhance the delivery of payloads nonspecifically. Hence, it is invaluable to identify peptides that target tumors and their microenvironments selectively, such as tumor-homing peptides (THPs), [208]. Besides CPPs, other peptide-mediated-cargo delivery are homing peptides (HPs) and cell-penetrating homing peptides (CPHPs) [209]. THPs navigate the cargo to tumor cells or tumor vasculature. Constructing a THP-conjugated payload is a step toward precise medicine for cancer diagnosis and therapy. The permeability of CPPs is highly dependent on their physiochemical properties, such as electric charge and the penetration mechanism. THP endocytosis is more specific via the tumor-cell-surface receptors or receptor-associated proteins [210]. To harmonize a peptide-drug conjugate with minimal off-target side effects, THPs are linked to a protease-cleavable linker and an anticancer molecule. RGD and NGR-containing peptides are the first generations of THPs as ligands for alpha integrin and aminopeptidase N, respectively. However, not all THPs are classified in RGD or NGR-containing triad ligands. For example, miscellaneous-peptide sequences have been reported to target tenascin-C or fibronectin-overexpressed glycoproteins in the ECM of tumors or interact with human-epidermal-growth-factor receptors [211].

Interestingly, we could identify a series of multifunctional CPTHP using predictors of THP. A total of 20 CPPs were predicted as THP, according to the THpep, SCMTHP, and TumorHPD programs (Table S7, and Table 3). tLyP-1 (CGNKRTR), a linear experimentally validated peptide, known as a THP with the ability to penetrate the anionic membrane of tumor cells, was used as the positive control [212]. tLyP-1 was predicted to be THP, according to the THpep, SCMTHP (score: 391.85), TumorHPD (SVM score:1.05), and NEPTUNE (score: 0.93) programs. CPP10-1537 (CSKICWRPRC) is predicted to be THP according to our analyses, and it contains “WRP” motif, which is able to bind VEGF-C as an antiangiogenic peptide. CPP10-1537 can pass the BBB and has been defined as the most potent THP, making it the candidate of choice for drug delivery in glioma. Kapoor et al. developed a database of experimentally validated THPs and concluded that THPs have a high frequency of some residues, such as Cys, Trp, and Arg, with a higher frequency of RR and especially Cys composition than normal peptides [213,214] This has been correlated to the positive charge of Arg to afford higher interaction with negatively charged cancerous tissue, disulfide bond formation, and higher stability of peptide due to the role of Cys and hydrophobic interaction of Trp with the target transmembrane receptor [209]. Besides, the presence of RC and GR are informative dipeptide compositions. Interestingly, five THPs found in this study, including CPP15-5, CPP15-4, CPP10-1725, CPP10-1389, and CPP10-757, were predicted to be ACPs as well (Table 5). This is similar to a CD13-binding peptide with both THP and ACP characteristics known as CNGRCGGKLAKLAKKLAKLAK [215]. Although the exact type of tumor that each THP might target is the bottleneck of investigations, we propose the conjugation of CPPs with ACP function identified in this study to THPs through a tumor-activatable protease linker. Carrier molecules, such as albumin, can be coupled to the construct to decrease renal-clearance rate in favor of a longer half-life if required.

#### 2.9.5. RGD-Containing Peptides

Integrins as cell-matrix adhesion molecules and growth factor receptors are distinguishable markers of tumor neo-vasculature. The integrin-protein family has 24 members, and as a heterodimeric-transmembrane glycoprotein contains one alpha and one beta subunit with subtype-selective substrates. The α_v_β_3_ integrin subtype is significantly overexpressed on angiogenic endothelial cells and interacts with VEGF receptors to develop tumor invasion. Up-regulation of α_v_β_3_ is observed in breast, gastric, prostate, pancreatic, ovarian cancers, glioma, oral squamous cell carcinoma, and non-small cell lung cancer [216]. The αvβ5 integrin subtype is mainly involved in tumor metastases. Ligands containing arginine-glycine-aspartic acid sequence known as RGD triad are preferential modules recognized by eight subtypes of integrins (αvβ1, αvβ3, αvβ5, αvβ6, αvβ8, αIIbβ3, α5β1 and α8β1), out of which α_v_β_3_, and α_v_β_5_ RGD-binding integrins are highly overexpressed in malignant tumor vessels [217].

Interestingly, out of the seven RGD-containing peptides identified in this study, SCMTHP predicted five RGD-containing peptides, including CPP10-1390 (RRRSITRRGD), CPP15-830 (VRRRRSITRRGDEEC), CPP15-829 (NVRRRRSITRRGDEE), CPP15-828 (INVRRRRSITRRGDE), and CPP15-827 (AINVRRRRSITRRGD) as THPs. They are different isoforms of *C. tessulatus* conotoxin (Q9BP75), all with strong antiangiogenic characteristics and moderate IFN-γ inducing potential. However, TumorHPD only assigned CPP10-1390 as a THP. Due to the overexpression of αvβ3 integrin, particularly in the brain tumors’ neovasculature, RGD peptide conjugated payloads for brain delivery are beneficial in glioblastoma therapy [218]. CPP10-1390 and CPP15-827 are RGD-containing THPs with BBB permeability. Radiolabeled RGD-containing substrates are valued for diagnostic delivery in positron emission tomography (PET) and single photon emission computed tomography (SPECT) [219]. RGD-containing nanoparticles have been used for the targeted delivery of cytotoxic chemotherapeutics, such as doxorubicin [220]. RGD-based peptides are also used in the construction of smart materials in vascular, ocular, and bone-tissue engineering [221,222]. Conclusively, RGD containing antiangiogenic peptides, such as CPP10-1390, are valuable to localize to the target tumor and inhibit tumor progression with higher efficacy in combination with bevacizumab similar to tumstatin [223].

Increased vascular penetrance and leakage are the consequences of sepsis progression. This permits the extravasation of pathogens into the blood circulation and distribution into organs resulting in organ failure and septic-shock induction. Various pathogens with RGD motifs in their surface proteins can interact with inner vessel endothelial integrins (mainly α_v_β_3_) to disturb cellular adhesion and vascular stability [224]. CPP20-429 (TQRDAINVRRRRSITRRGDE) and CPP20-428 (ATQRDAINVRRRRSITRRGD), both from *C. tessulatus* conotoxin (Q9BP75) andCPP10-1390 (RRRSITRRGD), are RGD-containing antibacterial peptides active against *E. coli*. These candidates can target the microorganism and interfere with pathogen adherence to endothelial cells and sepsis dissemination. Therefore, novel RGD-incorporated antibiotics can be designed to antagonize pathogen-endothelium attachment in the new era of antibiotic development [225].

#### 2.9.6. NRP-Targeting Peptides

**Figure 10:**
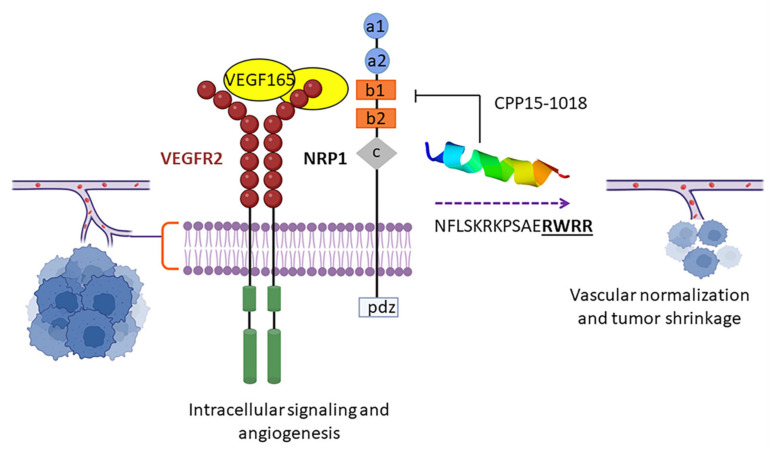
Tumor neovascularization is mediated by the interaction of VEGF-A with its receptor VEGFR2 and the co-receptor neuropilin 1 (NRP1). An NRP1 ligand containing the “RXXR” sequence at the C-terminal (C-endR) antagonizes the interaction of VEGF to NRP1 and signaling, which results in vascular normalization and tumor shrinkage.

Neuropilins (NRPs) are dominantly non-tyrosine kinase-transmembrane- glycoprotein-surface receptors with two isoforms, namely NRP1 and NRP2. NRPs have pleiotropic functions in the neural, cardiovascular, and immune systems. They act as coreceptors for VEGFs and are involved in angiogenesis and vascular leakage in normal embryonic conditions and pathological states, such as cancer. Knock out or overexpression of NRP1 results in vascular defect and hypervascularization, respectively. It was clarified that NRP1 and NRP2 signaling are involved in angiogenesis and lymphangiogenesis, respectively. NRP1-VEGF interaction is crucial for the recruitment and subsequent immunosuppressive functions of Tregs and tumor-immune tolerance [226]. For example, in glioma as well as skin and breast cancer, VEFGA-NRP1 is the main signaling pathway. Therefore, targeting the VEFGA-NRP1 and VEFGC-NRP2 axes seems reasonable to become a main target for anti-tumor strategies. NRP binds to the C-terminal motif R/KXXR/K, mediating the uptake of C-terminal Arg-containing peptides [227]. Growth factors, such as VEGF-A_165_ and TGF-β, use the aforementioned motif to bind to NRP. Tumor cells overexpress NRP to support tumor vasculature, growth, and progression. Other ligands following the exposed C-terminal R/KXXR/K motif known as the C-end rule (C-endR) might act as competitive inhibitors of VEGF to inhibit angiogenesis and metastasis (Figure 10). These receptors are becoming valuable therapeutic targets to modulate vascular leakage and endocytic internalization for smart drug delivery to extravascular tissue. Although basic residues are essential at positions 1 and 4, Arg is critical as the last residue for efficient interaction with NRP binding pocket. Notably, the cryptic R/KXXR/K consensus sequence can target NRP after protease cleavage and C-terminal exposure of the motif [228]. CPP10-631, CPP10-768, CPP10-771, CPP10-1383, CPP10-1636, CPP10-1657, CPP15-343, CPP15-429, CPP15-459, CPP15-1018, and CPP20-155 had C-endR penetration motif for extravasation of cargo loading. CPP10-1636 is predicted as an antiangiogenic peptide and contains a C-endR sequence. This peptide was docked against NRP1b1 (PDB ID: 5dQ0) and NRP2b1 (PDB ID: 3i97) receptors to verify if it can be applied as an antagonist for neuropilin receptor to suppress tumor growth and invasion. CPP10-1636 (RKPSAERWRR), originated from *C. pennaceus* conotoxin (P56711), showed a higher affinity toward NRP1 with a binding energy of about −195.837 Kcal/mol than NRP2 (−178.288 Kcal/mol). Similarly, CPP10-1383 (RDAINVRRRR) from *C. tessulatus* conotoxin (Q9BP75) as an antiangiogenic peptide displayed a much firmer interaction with NRP1 (−202.194 Kcal/mol) than NRP2 (−160.103 Kcal/mol). The most potent NRP antiangiogenic antagonist was CPP15-1018 (NFLSKRKPSAERWRR) derived from *C. pennaceus* conotoxin (P56711) with a stronger binding affinity against NRP1 (−218.903 Kcal/mol) than NRP2 (−189.903 Kcal/mol) (Figure 10). A7R (ATWLPPR) was used as the positive control in docking studies [226]. Altogether, NRP-targeting ligands can be optimized to deliver imaging probes or therapeutics to solid tumors [229].

### 2.10. Antioxidant Peptides

The production of free radicals with unpaired electrons as highly reactive agents originates from oxidation, a fundamental reaction in various biological processes at low concentrations. Free radicals are unstable and accept or donate electrons; therefore, they might damage various cellular components at high concentrations, namely as oxidative stress. Oxidative stress is involved in the etiology of various ischemia/reperfusion injuries, such as the brain, heart, and kidneys. Mitochondria are the primary organelle in producing reactive oxygen species (ROS) due to the e-leakage from the electron transport chain and oxygen presence. Mitochondrial deterioration mediated by ROS is highly associated with aging. In addition, peroxisome, CYP450s, and NADPH oxidase enzymes are extra-mitochondrial generators of ROS [230]. Antioxidants contradict the linked responses provoked by radicals through various mechanisms, including scavenging free radicals, scavenging oxygen, regenerating antioxidants, or chelating metal ions. While natural-derived peptides are generally considered safe, currently available synthetic antioxidants have hazardous side effects [231]. To prevail over oxidants, both well-timed administration and penetration ability to the damaged tissue is vital.

According to the AnOXPred program (Table S8), the most potent and safe free radical scavenger (FRS) peptides were CPP15-423 (VPFFGKRLHHTCPCL) derived from *C. chanos* toxin (A0A6J2VNJ3), CPP15-45 (KQLIRRHYWEIKWSG) from *Pterois volitans* Pvtoxin, and CPP10-1743 (SCWQRYPERC) in *C. coronatus* conotoxin (S4UJD3). Although the required knowledge correlated to the exact characteristics of antioxidant peptides is insufficient, the role of sulfur and imidazole ring in Cys and His, N-terminal located Leu and Val, as well as the involvement of Trp, Phe, and Tyr, has been emphasized [232]. MPPs with antioxidant characteristics protect mitochondria from oxidative damage and result in the rejuvenation of bioenergetics in aging [233]. A cell-permeable mitochondrial targeting antioxidant with promising effects to alleviate atherosclerosis and acute kidney injury has been reported to lower intracellular oxidants and decrease lipid peroxidation, diminishing ischemic damage and post-stroke disability [234]. CPP10-421 (ILLPALRKFC), defined in *C. textile* conotoxin (P0C1N6), and CPP10-933 (NRIALTVRSA), a *C. betulinus* conopeptide (A0A075ILT2), are mitochondrial targeting CPPs and antioxidants with ion-chelating mechanism. CPP15-541 (SLKNFWKRNLYLRDE), derived from *C. betulinus* conotoxin (A0A142C1Q1), is an FRS antioxidant targeting the mitochondria.

Antioxidant administration is intensively recommended for neurodegenerative disorders, such as Huntington’s, Alzheimer’s, and Parkinson’s. However, existing antioxidants available in the market might not be effective in penetrating the target organelle through BBB. It has been shown that reducing β-amyloid formation and improving cognitive performance in AD is amenable with mtCPPs [235]. Interestingly, CPP10-186 (KAYLFRNLAK) from *S. horrida* stonustoxin (Q91453) is an ion-chelator antioxidant MTP with BBB permeation. CPP10-757 (VRWYRNGTCR) and CPP10-1743 (SCWQRYPERC) were defined as FRS antioxidants with significant BBB permeation ability suitable for scavenging ROS in the peripheral and central compartments. Investigation of peptides to ameliorate AD progression has been performed by various strategies [236]. To identify the most optimal and safe candidates for Alzheimer’s, the intersection of BBB permeating peptides, antioxidant peptides, IL-4 and IL-10 inducing peptides, with non-inducers of TNF-α, and non-inducers of IL-6 were calculated. A total of seven peptides, including CPP15-244, CPP15-557, CPP15-1018, CPP15-76, CPP15-380, CPP15-211, and CPP15-212, were achieved. In between, CPP15-557 (HGFLTRRSLKDFWKR), derived from *C. betulinus* conotoxin (A0A142C1P9), shows mitochondrial targeting, which makes it an optimal candidate for this complex disorder. Taken together, the utilization scope of antioxidant CPPs found in our study provides an outlook to be applied for broad purposes, from chronic-allograft nephropathy and kidney- transplantation rejection as a result of ischemia/reperfusion injury to oxidative stress-inducing neural damage in macular degeneration.

## 3. Materials and Methods

### 3.1. Retrieval of Cryptic CPPs from Toxins and Analysis of Safety and Physiochemical Characteristics

Aquatic venomous animals were explored in electronic databases, such as Scopus, Pubmed, ISI, google scholar, and printed books using a combination of the “Persian Gulf”, “Arabian Gulf”, “venom”, and “toxin”. Toxins of the related species were retrieved in FASTA format from the UniProt database. Protein sequences of toxins were scanned for encrypted CPPs with a peptide fragment length of 10, 15, and 20 amino acid residues using Cell-PPD, a support vector machine-based (SVM) program with a threshold value of zero. This program has a high performance with sensitivity, specificity, accuracy, and Matthew’s correlation coefficient (MCC) of about 92%, 88%, 90%, and 0.81, considering the following formula [237].

(1) $$Sensitivity=\frac{TP}{TP+FN}*100$$

(2) $$Specificity=\frac{TN}{TN+FP}*100$$

(3) $$Accuracy=\frac{TP+TN}{TP+TN+FP+FN}*100$$

(4) $$MCC=\frac{\left(TP*TN\right)-\left(FP*FN\right)}{\sqrt{\left(TP+FP\right)\left(TP+FN\right)\left(TN+FP\right)\left(TN+FN\right)}}$$

While *TP* and *TN* are true-predicted positive and negative results, respectively, *FP* and *FN* are false-predicted positive and negative outputs. Sensitivity is denoted by the rate of correctly predicted true positives, and specificity is related to the rate of excluding true-negative results. While accuracy is the power of a program to distinguish between *TP* and *TN*, *MCC* is the association of anticipated and observed data that varies in the range of −1 to +1. The closer the value of *MCC* is to one, the more accurate the prediction [238]. Physiochemical properties of extracted peptides, such as hydropathicity, isoelectric point (pI), and total net charge were also verified by CellPPD. Thereafter, a machine learning-based prediction of CPPs called MLCPP was applied to predict the permeation and uptake efficiency of CPPs with an accuracy of about 0.896 and 0.725 in the first and second layers, respectively [239].

Peptides with high penetration were submitted to the ToxinPred program using the SVM method with an accuracy of 94.50% and an *MCC* value of 0.88 to exclude toxic peptides for drug-delivery purposes [240]. HemoPI program was implemented to predict hemolytic potency [18]. AllerTOP v.2 program, which applies machine-learning approaches, such as *k* nearest neighbors (*k*NN) method with an accuracy of 85.3%, has been used to predict the allergenicity of peptides [241]. The propensity to in vitro aggregation and hydrophobic moment (µH) were determined using the DBAASP tool [242]. Peptide’s half-lives were determined using PlifePred by batch submission to the sequence-based natural module [55]. Water solubility and modeling of peptides were determined using the PepCalc and the I-TASSER programs (https://zhanggroup.org/I-TASSER/) [243,244]. The protease susceptibility of peptides was determined using Prosper webserver [245].

Seq2Logo-2.0 was used as a sequence-logo generator for visualization of sequence weighting according to the default settings. The formation of potential disulfide bonds was calculated by the DiANNA program [246]. Stable helical conformation of peptides in lipid bilayers that simulates experimental conditions at the physiological pH and temperature was defined by the FMAP 2.0 program using 1,2-Dioleoyl-sn-glycero-3-phosphocholine (DOPC) as membrane bilayer [247]. The targetP-2.0 program was used to define mitochondrial-targeting peptides [248]. Classic nuclear localization signals (cNLS) were distinguished using the cNLS Mapper by setting the cut-off score at 7.0. Detected NLS with a score higher than 8.0 represents exclusive nuclear localization, while a score between 7-8 indicates partial nuclear localization. HPEPDOCK server was used to verify the binding free energy of the modeled peptides following the C-endR rule towards neuropilin receptors on tumor cells [249]. B3Pred program that exerts a random-forest-based model (RF) with an accuracy of about 85.08% was adopted to define blood-brain barrier penetrating peptides with an adjusted threshold of 0.1 [250].

### 3.2. Identification of Cytokine-Inducing Peptides

IL-2 inducing peptides were defined by the IL2Pred program based on the RF method with hybrid features having an accuracy of about 73.25% [251]. The hybrid model that applies the SVM+ motif-based method in the IL4pred program with an accuracy of 75.76% was used to screen IL-4 inducing peptides [252]. Induction of IL-10 production was assessed by the IL-10Pred program that operates an SVM-based model using dipeptide composition with an accuracy of 78.42% [253]. IFN-γ inducing peptides were identified using the IFNepitope program using the hybrid approach that performs through motif-based methods and SVM predictions. The sensitivity, specificity, and accuracy of the program performance adjusted on the hybrid approach were about 86.97%, 68.38%, and 80.93%, respectively [254]. TNF-α inducing peptides were defined using the TNFepitope program adjusted to the main prediction model. IL-6Pred, with an accuracy of 77.39%, was used for the identification of interleukin 6, inducing peptides [255]. VaxinPAD program, an SVM-based hybrid model which applies dipeptide composition and motif occurrence with an accuracy of 95.71%, was used to define peptides with immunomodulatory properties targeting PRR on APCs as vaccine adjuvants [114].

### 3.3. Identification of Antimicrobial Peptides

CAMP_R3_ program, with a balanced accuracy of about 89% using RF, SVM, and discriminant analysis (DA) models, was used to predict AMPs [120,256]. AMPs that are able to target specific bacterial and fungal species with a MIC lower than 25 µg/mL were defined using the DBAASP prediction tool [242]. AntiTbPred was applied to predict antitubercular peptides using the AntiTB_MD SVM ensemble method with sensitivity, specificity, and accuracy of 80.20%, 72.89%, and 76.56%, respectively [257]. The dPABBs program—with sensitivity, specificity, and accuracy of about 92.5%, 97.73%, and 95.24%, respectively—was applied to verify antibiofilm properties of CPPs using SVM-based whole AAC model [161]. Antifungal peptides were retrieved by the Antifp program (https://webs.iiitd.edu.in/raghava/antifp/predict3.php) with an accuracy of 83.33% and according to the default settings [258]. Meta-iAVP program with an overall accuracy of 95.2% was used to distinguish antiviral peptides *versus* non-antivirals [259]. AVP-IC_50_Pred was used to calculate the IC_50_ values for peptides against HIV, HCV, RSV, INFV, and HSV. The concentration of an AMP rendering hemolysis of 50% of RBCs per MIC is called therapeutic index or TI, wherein higher values are considered safer for the host [260]. Boman-index, which is an indicator of the protein-binding potential of a peptide, was calculated using the APD3 program (https://aps.unmc.edu/prediction/predict) [261]. Although a value higher than 2.48 shows a protein target for the peptide, values lower than 1.0 do not abrogate AMP property and stand for fewer side effects to the host.

### 3.4. Identification of Anticancer and Tumor-Homing Peptides

AntiCP 2.0 with an accuracy of 75.43% and 92.01% on the main and alternate datasets [262], ACPred with an overall accuracy of 95.61% [263], and iACP_FSCM were used to predict ACPs [264]. The TargetAntiAngio program using various classes of peptide features and the RF classifier with an accuracy of 77.50% was used to define antiangiogenic peptides [195]. TumorHPD, a sequence-based predictor using SVM model with an accuracy of 86.56%, and THPep which uses an RF classifier with an accuracy of about 90.13%, were applied to define tumor homing peptides (THPs) [209,265]. A newly developed program known as SCMTHP that identifies THPs by the scoring card method (SCM), outperforming ML-based predictors, was also applied to define THPs with an accuracy of 82.7% [214]. Antioxidant peptides were predicted using the AnOxPePred-1.0 program [232]. All programs and webservers were accessible in November 2022. 

## 4. Conclusions

The plethora of modes of functions cements the peptide’s position as the subsequent "go-to" therapeutic agent, for challenging disease states. We have shown, by big-data analysis, that marine-derived toxins, such as conotoxins, stichotoxins, and verrucotoxin, are rich sources of therapeutic leads and molecular probes. Peptides are usually used in a cocktail of conjugated peptides and adaptors or several complexes to overcome a constraint. We have used state-of-the-art artificial intelligence to assess the hypothesis of one-drug-multiple-target that is beneficial for a pronounced additive or synergistic- therapeutic effect. We have introduced CPPs with innate biological activity; hence, instead of multistep conjugation of peptides, a one-pot generation of therapeutic CPPs can be proposed similar to single-molecule polypharmacology which is proved to have a bright perspective in drug discovery. For example, as the concentration of antibiotics in the host- intracellular compartment is lower than their MIC, numerous treatments for internalized microorganisms are inefficient at therapeutic doses, which can result in additional drug resistance. Therefore, CPPs with AMP characteristic introduced in this study is greatly valuable. On the other hand, peptides are evolved by nature in a way to combat various targets; hence, they do not rely on a single mode of action which is highly valuable for bypassing multidrug-resistance mechanisms in clinical applications. In order to move to the post-antibiotic time, novel antimicrobial modalities, such as host-directed treatments, should be adopted. Therefore, we have introduced multifunctional AMPs that target the microorganism directly and modulate the immune system as a new paradigm in antimicrobial therapy. CPPs with AMP properties are proposed as a component in antibiotic regimens to reduce pathogen resistance. We have also introduced tumor-homing immunomodulatory ACPs that are not only able to target tumor-cell membrane and intracellular apoptotic/necrotic cascades but also affect TME, such as angiogenesis, and TIME, such as APC induction. Since ACPs are derived from AMPs, we have found several peptides, such as CPP10-297, CPP10-299, and CPP10-757, with dual AMP/ACP characteristics, which should be optimized for the defined purposes by further experimental studies. This combinatorial platform for drug discovery and development provides a much more optimistic perspective in the era of drug resistance.

## Figures and Tables

**Table 1:** A collection of Persian Gulf venomous animals.

Phylum	Class	Order	Family	Species	Reference
**Cnidaria** ** 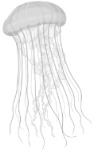 **	Cubozoa	Chirodropida	Chirodropidae	*Chironex fleckeri*	[22]
		Carybdeida	Carukiidae	*Carukia barnesi*	
	Hydrozoa	Siphonophorae	Physaliidae	*Physalia physalis* *Physalia utriculus*	
		Anthoathecata	Milleporidae	*Millepora alcicornis*	
	Scyphozoa	Semaeostomeae	Cyaneidae	*Cyanea capillata*	
			Pelagiidae	*Chrysaora quinquecirrha* *Pelagia noctiluca*	
		Coronatae	Linuchidae	*Linuche unguiculata*	
		Rhizostomeae	Cassiopeidae	*Cassiopea Andromeda*	[23]
			Rhizostomatidae	*Rhopilema esculentum* *Rhopilema nomadica*	[24,25]
	Anthozoa	Actiniaria	Stichodactylidae	*Stichodactyla haddoni*	[26]
**Echinodermata** ** 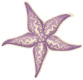 **	Ophiuroidae	Ophiacanthida	Ophiocomidae	*Ophiomastix annulosa*	[22]
	Asteroidea	Valvatida	Acanthasteridae	*Acanthaster planci*	
		Spinulosida	Echinasteridae	*Echinaster* sp.	
		Velatida	Solasteridae	*Solaster* sp.	
	Holothuroidea	Aspidochirotida	Holothuriidae	*Holothuria scabra*	[27]
		Dendrochirotida	Sclerodactylidae	*Ohshimella ehrenbergii*	
	Echinoidea	Diadematoida	Diadematidae	*Echinothrix* sp.	[22]
		Echinothurioida	Phormosomatidae	*Phormosoma* sp.	
			Echinothuriidae	*Araeosoma* sp. *Asthenosoma* sp.	
		Camarodonta	Toxopneustidae	*Toxopneustes pileolus* *Tripneustes* sp.	
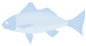 **Chordata**	Actinopterygii	Scorpaeniformes	Scorpaenidae	*Pterois volitans* *Pterois lunulata* *Pterois antennata* *Pterois russelli* *Dendrochirus zebra* *Scorpaenopsis oxycephala*	[22,28,29]
			Synanceiidae	*Pseudosynanceia melanostigma* *Synanceia trachyni* *Synanceia verrucosa* *Synanceia horrida*	[22,30]
			Scorpaenidae	*Scorpaena plumieri* *Scorpaena spotted*	
		Perciformes	Scatophagidae	*Scatophagus argus*	[31]
			Latidae	*Lates calcarifer*	[22,29]
			Lutjanidae	*Lutjanus lutjanus*	
			Serranidae	*Epinephelus coioides*	
			Siganidae	*Siganus canaliculatus*	
			Sparidae	*Sparus aurata*	
			Pomacentridae	*Amphiprion clarkii*	
		Gonorynchiformes	Chanidae	*Chanos chanos*	
		Blenniiformes	Blenniidae	*Salarias fasciatus*	
		Carangiformes	Carangidae	*Seriola dumerili*	
			Echeneidae	*Echeneis naucrates*	
		Mugiliformes	Mugilidae	*Mugil cephalus*	
		Pleuronectiformes	soleidae	*Pardachirus marmuratus*	
		Siluriformes	Plotosidae	*Plotosus lineatus*	
		Tetraodontiformes	Molidae	*Mola mola*	
			Tetraodontidae	*Lagocephalus lunaris* *Takifugu oblongus*	
** 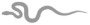 **	Reptilia	Squamata	Elapidae	*Hydrophis hardwickii* *Hydrophis ornatus* *Hydrophis cyanocinctus* *Enhydrina schistosa* *Toxicocalamus longissimus* *Laticauda* sp. *Pelamis platurus* *Hydrophis lapemoides*	[22,32,33,34,35]
	Chondrichthyes	Milyobatiformes	Dasyatidae	*Himantura gerrardi* *Hemitrygon bennetti* *Dasyatis kuhlii* *Himantura walga* *Pastinachus sephen*	[22,36,37,38]
			Myliobatidae	*Aetobatus flagellum*	[39]
			Gymnuridae	*Gymnura poecilura*	
**Porifera** 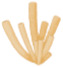	Demospongiae	Clionaida	Clionaidae	*Cliona celata* *Cliona vastifica*	[40,41]
**Mollusca** ** 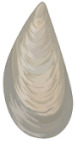 **	Gastropda	Neogastropoda	Conidae	*Conus textile* *Conus coronatus* *Conus betulinus* *Conus elegans* *Conus flavidus* *Conus monile* *Conus striatus* *Conus tessulatus* *Conus abbreviates* *Conus pennaceus* *Conus frigidus*	[42,43,44,45,46]

**Table 2:** Safe CPPs with the potential of disulfide-bond formation according to the DiANNA program proposed for endosomal escape.

Peptide ID	Pattern of Disulfide Bond Formation	* Protease Sensitivity	* BE DOPC (Kcal/mol)	Toxin ID	Species
**CPP10-352**	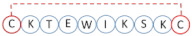	R	−2.4	Q8UW25 (cys-rich venome)	*Hydrophis hardwickii*
**CPP10-1032**	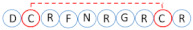	R	−1.2	A0A068B6R0 (conotoxin)	*Conus betulinus*
**CPP10-1344**	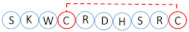	R	−1.4	P0C195 (µ-conotoxin)	*Conus kinoshitai*
**CPP10-1537**	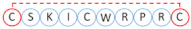	S	−1.6	Q9UA85 (conotoxin)	*Conus abbreviatus*
**CPP10-1743**	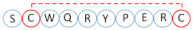	S	−1.7	S4UJD3 (conotoxin)	*Conus coronatus*
**CPP15-3**	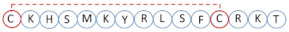	S	−1.3	B1B5I9 (δ-stichotoxin)	*Stichodactyla haddoni*
**CPP15-4**	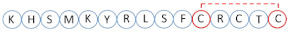	S	−1.7	B1B5I9 (δ-stichotoxin)	*S. haddoni*
**CPP20-2**	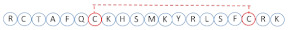	S	−3.2	E2S062 (κ-stichotoxin)	*S. haddoni*
**CPP20-209**	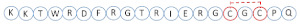	S	−3.0	P01437 (neurotoxin)	*Hydrophis lapemoides*

* R: Protease resistance, **S:** Protease sensitive, **BE:** Binding energy upon interaction with the zwitterionic DOPC membrane.

**Table 3:** CPPs with the potential to target special cells, tissues, or subcellular organelles.

Target	Peptide ID	Peptide Sequence	Charge	Hydrophobicity (%)	µH	Toxin ID	Species
BBB-pp *	CPP10-946	KKGRKNLWRR	6	20	0.438	A0A142C1C7	*Conus betulinus*
	CPP10-963	KNFWKRNLYL	3	40	0.371	A0A142C1Q1	*C. betulinus*
	CPP10-757	VRWYRNGTCR	3	20	0.241	A0A6J2VWA1	*Chanos chanos*
NLS *	CPP15-29	KPKKLRPSTKDYWYI	4	33.33	0.267	F2ZAF1	*Pterois volitans*
	CPP15-1019	FLSKRKPSAERWRRD	4	33.33	0.330	P56711	*Conus pennaceus*
	CPP15-377	KRPRENIRFLSKRKS	6	26.67	0.34	Q9BHB7	*Conus textile*
mtCPP *	CPP15-81	KLQATIAKKLFAIRS	4	53.33	0.349	Q91453	*Synanceia horrida*
	CPP15-144	KLNLQRTIAKKLLSI	4	46.67	0.361	A0A068BD83	*Dendrochirus zebra*
	CPP20-272	LLTRRSLKNFWKRNLYLRDE	4	35.00	0.215	A0A142C1Q1	*C. betulinus*
THP *	CPP10-1537	CSKICWRPRC	3	30.00	0.244	Q9UA85	*Conus abbreviatus*
	CPP10-1743	SCWQRYPERC	1	20	0.674	S4UJD3	*Conus coronatus*
	CPP10-1344	SKWCRDHSRC	2.5	10	0.621	P0C195	*Conus kinoshitai*

* BBB-pp: Blood-Brain-Barrier penetrating peptide; **mtCPP**: mitochondrial targeting cell-penetrating peptide, **NLS**: Nuclear-localizing signal; **THP**: Tumor-homing peptide; **µH**: hydrophobic moment.

**Table 4:** Characteristics of selected antimicrobial peptides and their target microorganisms.

Peptide Name	Peptide Sequence	Charge	µH	Boman Index (Kcal/mol)	BBB	AMP Targets	Toxin Uniprot ID	Species
**CPP10-11**	MKYRLSFCRK	4	0.363	3.29	√	AFP (Ca), AVP, APCE	E2S062	*Stichodactyla haddoni*
**CPP10-757**	VRWYRNGTCR	3	0.241	4.55	√	AVP, RSV, INFV, HSV, BA, TB	A0A6J2VWA1	*Chanos chanos*
**CPP10-858**	EKPLKRRVKQ	4	0.472	4.99	×	AFP, AVP, Kn, APCE	B0KZ78	*Conus betulinus*
**CPP15-81**	KLQATIAKKLFAIRS	4	0.349	4.62	×	BA, APCE, Sa, Ec, Pa, Kn	Q91453	*Synanceia horrida*
**CPP15-134**	KRQCRGVRVTRRSLR	7	0.390	6.09	√	BA, Sa, Ec, Kn, TB, AFP, APCE	Q98993	*Synanceia verrucosa*
*** CPP15-1029 ***	RDAINFRWRRSLIRR	5	0.15	5.76	√	Sa, Ec, Kn, TB, APCE	Q9BP63	*Conus pennaceus*
**CPP15-1030**	DAINFRWRRSLIRRT	4	0.226	4.94	√	Sa, Ec, Kn, TB, APCE	Q9BP63	*C. pennaceus*
**CPP15-1043**	AINVRRRRSITRRVS	6	0.189	5.72	√	Sa, BA, APCE	Q9BP64	*C. pennaceus*
**CPP15-1050**	NRLKENIKFLLKRKT	5	0.399	3.47	√	Ec, Kn, APCE, IL-4, IL-10, IFN-γ	Q9BPA6	*C. pennaceus*
**CPP20-45**	KRQCRGVRVTRRSLREFSHF	6.5	0.379	5.01	√	Ec, Kn, BA, IL-4, IL-10, IFN-γ	Q98993	*S. verrucosa*
**CPP20-48**	KKICNDYKLNLQRTIAKKLL	5	0.231	2.02	×	Kn, AVP, APCE, IL-6, IFN-γ	A0A068BD83	*Dendrochirus zebra*
**CPP20-51**	RRFERYQQVLCNKGLSRRHY	5.5	0.189	4.70	√	AVP, Ec, Kn, TB, BA, IL-10, IFN-γ	A0A068BFX2	*D. zebra*
**CPP20-156**	RAKINLLSKRKPPAERWWRW	6	0.304	3.39	√	Ec, Kn, TB, AFP, RSV, INFV, HSV, IFN-γ	Q9BHA0	*Conus textile*
**CPP20-184**	VERAGENRSKENIKFLLKRK	4	0.207	3.98	×	AVP, BA, IL-4, IL-10, IFN-γ, HCV	Q9BPB7	*C. textile*

**AFP:** Antifungal peptide, **APCE**: antigen-presenting cell epitope, **AVP:** Antiviral peptide, **BA**: Biofilm active, **Ca:** *Candida albicans*, **EC**: *Escherichia coli,* **HCV:** hepatitis C virus, **HSV:** herpes simplex virus, **INFV:** influenza virus, **Kn**: *Klebsiella pneumonia*, **Sa**: *Staphylococcus aureus*, **Pa:** *Pseudomonas aeruginosa,* **TB:** Tuberculosis, **µH**: Hydrophobic moment.

**Table 5:** Predicted anticancer peptides according to the calculated intersection of AntiCP 2.0, ACPred, and iACP-FSCM programs.

Peptide Name	Peptide Sequence	DOPC BE (Kcal/mol)	Mit BE (Kcal/mol)	Charge	BBB Score	APCE (ImmunoAdjuvant Score)	Potential ACP Mechanisms	Toxin Uniprot ID	Organism
**CPP10-186**	KAYLFRNLAK	−2.5	−2.5	3	0.23	-	MTP (0.57), PDL-1B	Q91453	*Synanceia horrida*
**CPP10-297**	LQRTIAKKLL	−4.0	-	3	-	0.13	mIL-2/low IFN	A0A068BD83	*Dendrochirus zebra*
**CPP10-298**	RTIAKKLLSI	−3.7	-	3	0.10	-	mIL-2/low IFN, PDL-1B	A0A068BD83	*D. zebra*
**CPP10-299**	TIAKKLLSIR	−3.7	−3.4	3	0.11	0.27	MTP (0.62) sIL-2/mIFN, PDL-1B	A0A068BD83	*D. zebra*
**CPP10-421**	ILLPALRKFC	−4.5	−4.9	2	0.13	-	MTP (0.64), sIL-2	P0C1N6	*Conus textile*
**CPP10-757**	VRWYRNGTCR	−1.8	-	3	0.34	0.02	THP, Anti-ang	A0A6J2VWA1	*Chanos chanos*
**CPP10-946**	KKGRKNLWRR	−1.9	-	6	0.43	0.5	Anti-ang sIL-2	A0A142C1C7	*Conus betulinus*
**CPP10-1389**	RRRRSITRRG	−0.2	-	6	0.29	0.85	THP, Anti-ang sIL-2/sIFN-γ	Q9BP75	*Conus tessulatus*
**CPP10-1725**	HIFWSKRNCC	−2.7	-	2.5	0.30	-	THP, Anti-ang sIL-2, PDL-1B	Q9BPH2	*Conus pennaceus*
**CPP15-4**	KHSMKYRLSFCRKTC	−1.7	-	5.5	0.24	0.29	THP, Anti-ang mIL-2/low IFN	E2S062	*Synanceia haddoni*
**CPP15-5**	HSMKYRLSFCRKTCG	−1.7		4.5	0.21	0.17	THP, Anti-ang, mIL-2	E2S062	*S. haddoni*
**CPP15-29**	KPKKLRPSTKDYWYI	−2.7	-	4	0.15	-	Anti-ang, NLS lowIFN	A0A068BFX2	*D. zebra*
**CPP15-44**	KKQLIRRHYWEIKWS	−2.7	-	4	-	0.71	Anti-ang	F2ZAF0	*Pterois volitans*
**CPP15-45**	KQLIRRHYWEIKWSG	−2.7	-	3.5	-	0.38	PDL-1B	F2ZAF0	*P. volitans*
**CPP15-58**	LKKKLKTFQKHYERL	−3.0	-	5.5	-	0.67	NLS mIL-2/low IFN	A0A2P1BRP3	*Scorpaena plumieri*
**CPP15-144**	KLNLQRTIAKKLLSI	−4.7	−4.6	4	-	-	MTP (0.69) lowIFN	A0A068BD83	*D. zebra*
**CPP15-423**	VPFFGKRLHHTCPCL	−3.6	-	3	0.09	0.63	Anti-ang, membrane	A0A6J2VNJ3	*C. chanos*
**CPP20-48**	KKICNDYKLNLQRTIAKKLL	−5.0	-	5	-	0.32	mIL-2/sIFN	A0A068BD83	*D. zebra*

**Anti-Ang**: Antiangiogeneis, **APCE**: Antigen presenting cell epitope, **BE**: Binding energy of peptide with membrane, **Mit**: Mitochondrial, **MTP**: Mitochondrial targeting peptide, **m/s**: moderate/strong inducing peptide, **NLS:** Nuclear localizing signal, **THP**: Tumor-homing peptide, **PDL-1B:** programmed death ligand-1 binding.

## Data Availability

Not applicable.

## Supplementary Materials

The following supporting information can be downloaded at: https://www.mdpi.com/article/10.3390/md20120763/s1, Table S1: Venomous species of Persian-Gulf, their toxin sequences, and the number of encrypted short fragment (10mer, 15mer, and 20mer) cell-penetrating peptides (CPPs) in each toxin; Table S2: The sequence of 3505 cell-penetrating peptides with a length of 10, 15, and 20 amino acids in each toxin; Table S3: Physiochemical analysis of CPPs and evaluation of their safety and efficacy; Table S4: CPPs with partial or exclusive potential of nuclear localization; Table S5: CPPs with intrinsic ability to target mitochondrial membrane; Table S6: Anticancer CPPs using three prediction programs and the intersection of prediction; Table S7: CPPs with innate tumor homing characteristics predicted by three programs; Table S8: CPPs with innate antioxidant potential through free radical scavenging or chelating metal ions.
