# Supplementary material for: Polypharmacological Cell-Penetrating Peptides from Venomous Marine Animals Based on Immunomodulating, Antimicrobial, and Anticancer Properties
Source: Mar Drugs. 2022 Dec 4;20(12):763. doi: 10.3390/md20120763 (PMC9787916; doi:10.3390/md20120763)
Supplement: Supplementary file 1 [file marinedrugs-20-00763-s001.zip › Table S1.pdf]

Table S1 Venomous species of Persian-Gulf, their toxin sequences, and the number of encrypted short fragment (10mer, 15mer, and 20mer) cell-penetrating peptides (CPPs) in each toxin

Species	Toxin name	Accession number	Protein sequence	CPP (10)	CPP (15)	CPP (20)	sum
Stichodactyla haddoni	KappaPI-stichotoxin-Shd2a	B1B5I8	MAKLYFLLCLALVACLTMATEEMPALCHLQPDVPKCRGYFPRYYYNPEVGKCEQFIYG GCGGNKNNFVSFEACRATCIIPL	2	0	0	2
	Delta-stichotoxin-Shd3a	B1B5I9	MAYLKIVLVALMLVLGVSAMRLSDQEDQDVSVVKRAACKCDDDGPDIRSATLTGTV DFWNCNEGWEKCTAVYTAVASCCRKKKG	4	0	1	5
	Kappa-stichotoxin-Shd5a	E2S062	MKFQVIAAVLLIAFCLCVVVTARMELQDVEDVENGFQKRRSCIDTIPQSRCTAFQCKH SMKYRLSFCRKTCGTC	7	5	1	13
	OMEGA-stichotoxin-Shd4a	B1B5J0	MASFRTLFACVVILCCVLWSSMARYGEDMEVETEMNKRDEGVRCTGQHASSFCLN GGTCRHIASLGEYYCICPGDYTGHRCDQKSG	0	0	0	0
Chironex fleckeri	Toxin CfTX-1	A7L035	MVKMLFFAFLPLLFMTGIAAESTISSGLNSLKTKIDAKMPSGKQLFDKVVEMQKQIDA KFSNDDERAKVMGAIGSLSTAVGKFQSGDPAKIASGCLDILVGISSVLKDFAKFSPIFSIL SLVVGLFSGTKAEESVGSVVKKAVQEQSDQELQEALYGVKREYAVSKAFLDGVRNETS DLSPTEVSALAANVPIYQGVRFIAMVVQRIKYIKPKTESEIKRMLTMLELFTDLCSLRDLI LLDLYQLVATPGHSPNIASGIKEVSNLGREEYKKVFEDLLKNDDKETYLFLSYLYPREKN EQSRKIFNFFDLMKVKYDDRLKQDLTGVKIFSNVHWPNYFMCSSNDYLALICTKPYGS LKLDKLNDGYYSIKTTQHDPKICHRYGNYILFTHKRNDDLEKFNFVPVKLEKREIYLLSSK ESPNKFAYVPQNADGALFFVDGIPSKVGYGNQGYFTLVE	8	2	1	11
	Toxin CfTX-2	A7L036	MILVSLLPLLFMTGIASESTISSGLASLKAKIDIKKPTGKQLFDKVKSMEQALENKFSDD DERAKVMGAIGSLGTAIGKFQSGDPASIASGCLDILVGISSVLKDFAKFSPVFSILSLVV GLFSGTKAEESVSSVVTKAIQEQSDQELQEALYGVKREFAVSKAFLDGVRNEESDLRPT EVSALAANIPVYQGVRFIAMVVQRIKYIKPKTESEIKRMLTMLELFTDLCSIRDLILLDLH QLIATPGHSPNIASGIKEVTSLGREEYQRVFEDLLKTDDEETFLFLSYLYPKEKNEQSRKI FKFFDLIEVKYDDRFKLDLSGGQALSTLQWPNYYLCPHNDYLANNCHDLRVGLKLEKL SDGFYTIKTYGRDPRTCYWTDDYVKISSTSNGELEKFSFVPVQVKGQKAYLLSTKKWP HNFAYSQKTANGLLSILKDVPSKLGYGNQGFFTISTYSNPKNRHA	14	9	2	25
Acanthaster planci	Plancitoxin-1	Q75WF2	MPSSVIMFTFLALTVLTAVMVGTSEAVSCMDNKNKPVDWFIVYKLPQDSASSKPVIR EGYGQMYMDVNNQALKFSSTSLKDDDHAIAYTVDDIYKNHGKGNLAHVMYNDQP PAGEEIQSGLVGHTKGVLAFDGTSGFWLVHSVPKFPLPASKSYNWPDNAKRNGQTL LCITFKYDQFEKIGQQLKYNYPGVYDSDLPSKLVGKTPSIVDLVKNVHVTSPPWNRQL NLQSKSGQTFVSFNKASKWGEDLYKNWLATHFKSGLYCETWQNGGRNLNSSCEAG LNVYNVKKVSLSGGSDFKGTKDHSKWAVTTKSGLKWTCIGGINRQTSQMYRGGGA VCLENANVHKAFYDSVAEYEPCT	1	1	0	2
Pterois volitans	Pvtoxin-b	F2ZAF1	MSSQILVVAALGRPFTLGTLYDARKVRLIPGFTLWEDDVLQANTVESRQPSSAFEIIAS DSIEDKSSLMDIEASLKASFLGGLVEVGGSAKYLDDRKKFKNQSRVTLQYKATTSFKQL MTNLETKHVEYSEYFQNLEATHVFIGILYGANAFFVFDSAKTESSNVQDIQGSMEAAI KKIPSVEISGKGEVKLTSEDSAITSGFSCKFHGDFFLPSNPTTFEDAVQTYQQLPQMM	22	14	1	37

			GKENAVPMTVWLVPLVNFYSEAPKLMADSSTPILRKVRNTLEAMRELEMRCNDALE DNTVKLFPLIEKKLNNFKNFFGDYMLNLRRTIAEKLQSIRSGSENESAMLQLFQENLKS PFNIDSLNMWMDCAEREINVLRSCIDIIEEARHKVVLSESEMFRELLDPNVKHAVCYV FTFVTDQDPFLNVLSDFLDSPTSKPKKLRPSTKDYWYISDDIPEMMRAKAHHFRNLAL DMDNRCVRFLVASIGNPKQEGAGIHYYREGIQIIDEFTKPWIPPVETIQDRRELLWCD CELTLDPDTAQPALTLSEGNKKAVSGTAKPFDNQQRRFQCYQQVLCNKGLSRRHYW EVEWSGYVRAGVTYKGISRRTWNQDSSLGHNKISWVFDYFPKSGYNHIFDAKKTKV NVSSPGFKKLGIYLDWPAGTLSFYMVDTIWVTHIHTFKARFQEAVFPAFLIGHGPDNG QIKLL				
	Pvtoxin-a	F2ZAF0	MSSEILIVAALGRPFTLGALYDSRKDRLIPGFTLWEDDVLQANTVESRQPSSAFEIIASD SIEDKSSLMDIEASLKASFLGGLVEVGGSAKYLDDRKKFKNQSRVTLQYKATTSFKQLM TNLETKHVEYSEYFQNLEATHVVIGILYGANAFFVFDSAKTESSNVQDIQGSMEAAIKK IPSVEISGQGSVKLTSEDSAITDRFSCKFHGDVLLPSNPTTFEDAVQTYQQLPQMMGK ENAVPMKVWLVPLVHFYSEAPKLMADSSISILRRVRNTLEAMRELEMRCNDALEDNT VKLFPLMVKKLNNFQNLFGVYMSNLRRSIAEKLQSIRSGSEDQSAMLQLFQENLKSPF NTDSLNMWMDCAEREINVLRSCIDIIEEARHKVVLSESEMFRELLDSNVKHAVCYVFT FVTDQDPFLKVLTDYLDSPDSRPKRTRPPATEYWYASDTIPEMMRDKAHIFYNLAKE MDNRRVRFLVASIVNPKQEGAGIHYYRENILIEQEFLQPKMNPVEKIVDRRDLLWCD CELTLDPVTAHPALTLSEGNKKAVSGKPKPSDNNPERFEHFQYVLCKKQLIRRHYWEIK WSGFVGAGVIRNSIHPKSSQVDSSLGGSDKSWVFEYNPNTGYRQIFKNTPENVNVSS TGFDRLGVYLDWLGGTLSYYMINNNFVTHLHTFHTLFKEAVYPAFMIGDKQNVHGEI ELL	29	14	1	44
Scorpaena plumieri	Cytolytic toxin-alpha	A0A2P1BRQ0	MSSDIIMAGLGRPFTLGFLYDARREKLIPGFSLFGDETLQKYATSTPQHSSDFQIVASDS VESKSNVMDIEASLGVSFLGGLVEVGGSAKYLNNTKKYQNQSRVTLQYKVTTTFKQFK APPGKVNVQQTAISDKNVATHVVTAILFGANAFFVFDSDKVEDSNLQDIQGKMEAVI KKIPSVSIEGSGSVQLTDEEKSLASNLSCKFHGDFLLESLPTTFEEAVMTYQKLPELVGEE ASDGVPMKVWLVPLTRFYSKADLLVRDISQGLVRKVHSILEDLHKLKRRANDSLEDDT VKLFPLLEKKLKNFQKNYSDYMTTFRRTISQKLQSIRKGDEDETAMLQLFEDRLRSPFN IDSLNMWMEITEREINVLRSCIDIIEETKHKAVLSQSQMVKDLLDSEVMHAVCYVFTY VTDKDHYLDALRDYLKSPNSRPARVRPVVTWVASNTVLETMREKAHLFRSLAKDME NRCVHFLVASIVNLKVEGAAIHYYRESVLIEPNFKHPIISAVEKIVDRRDLLWYDCELTLD PNTSHPSLYLSEGNKKAVTGTLRAFDNNPERFGLWQQVLCNKGLSRRHYWEVEWN GYVIVGVTYSSIGRKNIDIQSFIGFSETSWTLMFIPKNGVAVKGARRSVSYYFISDLAFPP LGLYHDCHASTLSFYKVSDNVLNHFHTIEIKPKLSEPVYAIIRIGDEDRPYHGTVRLL	18	11	7	36
	Cytolytic toxin-beta	A0A2P1BRP3	MPSDILVVAALGRPFTLGALYDARKDKLYPGFTLWEHEVLEESTVESDQPSSTFEITAS DSIDDKSSLMDIEASLKASFLGGLIEVGGSAKYLNDTKKFKNQSRVTLQYKATTSFKQL MTNLETKHVEYSEYFQNIEATHVVIGILYGANAFFVFDSDKVDSSNVQDIQGSMEAVI KKIPSVEISGQGSVQLTSEESDITNSFSCKFHGDFHLPSNPTTFEDAVKTYQQLPQMM GKETAVPMTVWLVPMTNFYSEAPQLMADSSTPILRKVRNTLEAMRQLDMRCNDSL ERRHSEAGFHCLKKKLKTFQKHYERLHVNPFRKNHFPETFSPSGKGTKMKLQCLPTFR NKLRSPSNINSLNMWMDCAEREINVLRSCIDIIEEAKHKVVLSKSQMARELDDSEVKH AVCYVFTYVTDYDPFLNALSDFSKSIKPKKYSPSKKDYWYTSDDVPEMMREKAHHFY	30	12	3	45

			NLAKDMENRCVRFLVASIVNPKEEGAAIHYYREGIQIINDFSNPRIPPVETIQDQESYS<br>GMTVSSPWKETAHPALHLSEGNKKAMSGKPQPSDNNPKRFDHYQQVLCNKGLSKR<br>HYWEVEWCGYVRAGITYKGIQRKTFASECSLGHTDMSWVFDYYPKSGYHHIYNNKK<br>VRVKVASPGFDRLGVYLDWPAGTLSFYMVTSTWVTHLHTFSIRFNEAVYPAFLIGHG<br>QKNANGQIKLKGE				
Synanceia horrida	Stonustoxin subunit alpha	Q98989	MSSDLVMPALGRPFTLGMLYDARREKLIPGFSLFGDETLQKYQSSNAQRSSEFKIVAS<br>DSTESKSSAMDIEASLGVSFLGGLVEVGGSAKYLNNTKKYQNQSRVTLKYKATTVYKQ<br>FTAPPGTVTVQETAITEKGLATHVVTSILYGANAFFVSDSDKVEDTNLQDIQGKMEAA<br>IKKIPTISIEGSASVQLTDEEKSLASNLSCKFHGDFLLESLPTTFEDAVKTYQTLPTLIGED<br>GANSVPMKVWLAPLKSYNSKAQQLIQEINVSKVRRIHTTLEELHKLKRRANEAMDVK<br>LVQRIPLIHDKISNFQQIFQDYMLTVQKKIAEKLPLVRAGTESEQSLQKIIDDRAQSPFS<br>NEKVSKWLDAVEREIAVLKSCAGMVEGTQAKFVSNQTELDREVLVGKVKHAVCFIFT<br>SVERNDPYLKVLSDYWESPPSNNAKDVAPSTEDKWCFSTEVVLKMQQRAQTFCDHV<br>NDFEKSRNVGFFITALENGKFQGASIYYYKEGSLATQDFTFPRMPFVQGYKKRSDLLW<br>YACDLTFDRNTINNWISLSDNDTFAASEHGKRQNYPKHPERFVSFNQVLCNEGLMG<br>KHYWEVEWNGYIDVGIAYISIPRKEIDFASAFGYNTYSWVLSYNPKIGYIERHKKREYN<br>VRAPNPGFKRLGLFLDWRYGSISFYAVSSDEVHHLHTFKTKFTEPVYPAFSIGPAGNH<br>GTLRLL	30	10	8	48
	Stonustoxin subunit beta	Q91453	MPSDILVVAALGRPFTLGMLYDARNDKLIPGFTLWEDEVIEESTLESSQPSSAFEIIASD<br>STDDKSSLMDIEASLKASFLGGLVEVGGSAKYLNNQKKFKNQSRVTLQYKATTSFKQL<br>MTNLGTKHVEYSELFENIQATHVVIGILYGANAFFVFDSNKVDSTNVQEIQGQMEAVI<br>KKIPSVEISGKASVQLTGEETDITNSFSCEFHGDFFLTTNPTTFEDAVKTYQQLPQMM<br>GKDNAVPMTVWLVPMVNFYSEAPQLMADSSTPILRKVRNTLEAIVQVQMRCNDAL<br>DDPTVNLFTEVQKKLSDFQKICDDHMSKLQATIAKKLFAIRSGDEDESALLNLFEENLQ<br>SPFNIESLNMWMEFEEREINVLRSCMDILTKAKPKVIFNQGVLFKGLYDSKVKHALCY<br>VFTNVTKNDVFLNVLNEFLDSPQSRPKKLRPSPKDYWYSYDDIPETMREKAYLFRNLA<br>KEMNNRCVHFFVTAIHNPKQEGAGIHYYRESIQIIDEFTKPYMPGVESIKDRRELQWY<br>DCELTLDPETAHQVLTLSEGNKKAVSGNTKSPTDHLEKFSHFQQVMCTKGLSGRHY<br>WELEWSGYVGAGVTYKGIGRKTSTSDSSLGKNEKSWLFEYSTKSGYQQIHNSKKTRV<br>TVSSTGFKLLGVYLDWPAGTLSFYMVNKAWVTHLHTFHTKFNEAVYPAFLIGDAQQK<br>VNGQIKLL	22	14	4	40
Synanceia verrucosa	Neoverrucotoxin subunit alpha	A0ZSK3	MSSDLVMPALGRPFTLGMLYDTRREKLIPGFSLFGDETLQQYQSSNTQRSSEFKIVAS<br>DSTESKSSAMDIEASLGVSFLGGLVEVGGSAKYLNNTKKYQNQSRVTLKYKATTIYKQF<br>TAPPGTVKVQETVITQRGLATHVVTGILYGANAFFVFDSDKVEDTNLQDIQGKMEAV<br>IKKIPTISIEGSASVQLTDEEKSLASNLSCKFHGDFLLESLPTTFEDAVTTYQTLPTLLGED<br>GASAVPMKVWLVPLKKFFSKAKLLTQEITVSKVRRIHTTLEELYKLKRRANEAMDDKL<br>VQQIPLIHDKISNFHQIFQDYMLTVQKKIAEKLPLVRAGTESEQSLQKIIDDRAKSPFSN<br>ENVSTWLEVIEREIAVLKSCAGMVEGTQAKFVSNQTELDREVLAEDVKHALCFVFTSV<br>ERNDPYLKVLSDYLESPDSKDGKEAVPSTEDKWCFSTRVVLKMKQRAQTFCDHVNDF<br>EKSRNVGFFVTALENGKFQGASIYHYKDGSLATQDFTFPRMPFVQGYKKRSDLLWYA<br>CDLTFDRNTINIWVSLSDNDTFAASEHGKRQNYPKHPERFLCYNQVLCNEGLTGKHY	25	11	3	39

			WEVEWNGYVDVGVAYISISRKEDNWVSAIGHNTCSWVFSSIPRAGYVERYNQRQYYVTVPTPGFKQLGVFLNWPDGSLSFYAVSSDEVHHLHTFKTKFTEPVYPAFCLGYRFDHGTVRLL				
	Neoverrucotoxin subunit beta	A0ZSK4	MPSDILVVAALGRPFTLGMLYDARNDKLIPGFTLWEDEVIEESTVESSQPSSAFEIIASDSIDDKSSLMDIEASLKASFLGGLVEVGGSAKYLNNQKKFKNQSRVTLQYKATTNFKQLMTNLGTKHVEYSELFENIQATHVVIGILYGANAFFVFDSNKVDSTNVQEIQGQMEAVIKKIPSVEISGKASVQLTSEETDITNSFSCEFHGDFFLTSNPTTFEDAVKTYQQLPQMMGKDNAVPMTVWLVPMVNFYSEAPQLMADSSTPILRKVRNTLEAIVQVQMRCNDALDDPTVNLFTEVQKKLSDFQIICDDHMSKLQATIAKKLFAIRSGDEDESALVNLFEENLQSPFNIESLNMWMEFEEREINVLKSCMDILTKAKPKVIFNQGVLFKELYDSKVKHGLCYVFTNVTKNDDFLTVLNDFLDSPQSRPKKLRPSPKDYWYSYDDIPEMMREKAHLFRNLAKEMNNRCVHFFVTAINNPKQEGAGIHYYRESIQIIHEFTKPHMPGVETIKDRRELQWYDCELTLDTETAHQVLTLSEGNKKAVSGSTKSPADHFEKFSHFQQVMCTKGLSGRHYWELEWSGHVSAGVTYKGISRKTSTPDSSLGKNQKSWVFEYTKKSGYQQIHNGKNARVTVSSIGFKQLGVYLDWPAGTLSFYMVNKAWVTHLHTFHTKFYEAVYPAFLIGDAQQKVNGQIKLL	25	14	5	44
	Verrucotoxin subunit beta	Q98993	MPSDILVVAALGRPFTLGMLYDARNDKLIPGFTLWEDEVIEESTVESSQPSSAFEIIASDSIDDKSSLMEIEASLKASFLGGLVEVGGSAKYLNNQKKFKNQSRVTLQYKATTNFKQLMTNLGTKHVEYSELFENIQATHVVIGILYGANAFFVFDSNKVDSTNVQEIQGQMEAVIKKIPSVEISGKASVQLTSEETDITNSFSCEFHGDFFLTSNPTTFEDAVKTYQQLPQMMGKDNAVPMTVWLVPMVNFYSEAPQLMADSSTPILRKVRNTLEAIVQVQMRCNDALDDPTVNLFTEVQKKLSDFQIICDDHMSKLQATIAKKLFAIRSGDEDESALVNLFEENLQSPFNTESLNMWMEFEEREINVLKSCMDILTKAKPKVIFNQGVLFKELYDSKVKHGLCYVFTNVTKNDDFLTVLNDFLDSPQSRPKKLRPSPKDYWYSYDDIPEMMREKAHLFRNLAKEMNNRCVHFFVTAINNPKQEGAGIHYYRESIQIIHEFTKPHMPGVETIKDRRELQWYDCELTLDTETAHQVLTLSEGNKRQCRGVRVTRRSLREFSHFQQVMCHQGAEWTPLLGVRVAGHVSAGVTYKGISRKTSTPDSSLGKNQKSWVFEYTKKSGYQQIHNGKNARVTVSSIGFKQLGVYLDWPAGTLSFYIGQQSLGDSSPHLPHQILRGCLSSLPDWGCTTESQWSN	37	22	8	67
Dendrochirus zebra	Tx alpha-subunit (Fragment)	A0A068BGX9	MSSDLVMPALGRPFTLGMLYDARKEKLIPGFSLFGDETLQQYESSKSQRSSAFEIVASDSIESKSSVMDIEASLGVSFLGGLVEVAGSAKYLNDSKKYQNQSRVTLKYNATTTYQQFTAPPGTVKVQQTAITEKRLATHVVTGILYGANAFFVFDSDKVEDSNLQDIQGKMEAAIRKIPTISIEGSGSVQLTDEEKSLASNLSCKFHGDFILEALPTTFQDAVKTYQKLPELLGEEGTDAVPMKVWLVPLKKFYSQADLLVRDISAGRVKKIHSIVEELFVLRRRCNEALDDKLVQQLPLIYDRVNYFQQLFQDYIHTVQQTIATKLPLIREGTEDESSLEKIAKDRAKSPFSNEKVSMWLDAIEREIGVLKTCARVMEKTQTKFVSNQTELDREVLAENAKHALCFVFTSMESSDPYLRVLSEYLESPTAEISGNVVPSTTDMWYYSNGVILKMKQKAQAFTDFADTVKNRRYVRFLAAAITNGKYQGASIYYYKEGSLETQDYTFPSLAPLETIKDRSDLLWYACDLTFDPNTAQENVVLSDDNKFALTGRSQNYSNHPERFTGIRQVMCKEGLTGRHYWEVEW	16	2	2	20
	Tx beta-subunit (Fragment)	A0A068BD83	MSSEILMVAALGRPFTLGTLYDARQDKLIPGFTLWEDDVLQANTVESRQPSSAFEIIASDSIEDKSSLMDIEASLKASFLGGLVEVGGSAKYLNDSKKFKNQSRVTLQYKATTSFKQL	16	4	2	22

			MTNLETKHVEYSEYFQNLEATHVVIGILYGANAFFVFDSAKTESSNVQDIQGSMEAAIKKIPSVEISGKGEVKLTSEDSAISSGFSCKFHGDFFLASNPTTFEDAVKAYQQLPQMMGKENAVPMTVWLVPLVHFYPKATKLMADSSLPILRRVHNTLEEMRQVEMRCNDALEDNIVKRFPQIRKVLNNFKKICNDYKLNLQRTIAKKLLSIRSGEEDESAMLKVSEENRRSPFNIDSLNKWMECKEREINVLRACIDIIEEAKHQNVLSESEMFRELLDPKVKHAVCYVFTSVEDYDPFLNVLKDFSESPKMTVNIKKLQPPPNYYWYISDDIPEIMREKAHHFRNLAKDMNNRCVRFFVASILNPNQEGAGIHYYREGIQIIDAFTKPQIPPVETIQDRRALLWYDCELTLDPDTAHPALTLSEGNKKAVSGKPKPFDNHPERFEHYQQVLCNKGLSGRHYWELEW				
	Tx gamma-subunit (Fragment)	A0A068BFX2	MSSQILMVAALGRPFTLGTLYDARKDRLIPGFTLWEDDVLQANTVESRQPSSAFELIASDSIEDKSSLMDIEASLKASFLGGLVEVGGSAKYLDDRKKFKNQSRVTLQYKATTSFKQLMTNLETKHVEYSEYFQNLEATHVVIGILYGANAFFVFDSAKTESSNVQDIQGSMEAAIKKIPSVEISGKGEVKLTSEDSAITDRFSCKFHGDFFLPSNPTTFEDAVKTYQQLPQMMGKENAVPMTVWLVPLVHFYSEAPKLMADSSLPILRRVRNTLEAMRELEMRCNDALEDNTVKLFPLMIKKLNNFQNLFGDYMLNLRRSVAEKLQSIRSGSEDQTAMLQLFQENLKSPFNTDSLNMWMDCAEREINVLRSCIDIIEEAKHKVVLSESEMFRELLDPNVKHAVCYVFTYVTDQDPFLNVLSDFLDSPTSKPKKLRPSTKDYWYISDDIPEMMREKAHHFRNLALDMNNRCVRFLVASIGNPKQESAGIHYYREGIQIIDVFNKPRIPPVETIQDRRELLWYDCELTLDPDTAQPALTLSEGNKKAVSGTAKPFDNQQRRFERYQQVLCNKGLSRRHYWELEW	26	16	2	44
Hydrophis hardwickii	Short neurotoxin 1	P68416	MKTLLLTLVVVTIVCLDLGYTMTCCNQQSSQPKTTTNCAESSCYKKTWSDHRGTRIERGCGCPQVKPGIKLECCHTNECNN	2	1	0	3
	Cysteine-rich venom protein 1	Q8UW25	MIAFIVLLSLAAVLQQSSGTVDFASESSNKKDYRREIVDKHNALRRSVKPTARNMLQMKWNSRAAQNAKRSADRCTFAHSPEHTRTVGKFRCGENIFMSSQPFAWSGVVQDWYDEIKNVVDGIGAKPPGSVIGHYTQIVWYKSHLLGCASAKCSSTKYLYVCQYCPAGNISSSIATPYKSGPSCGDCPSACVNGLCTNPCEYEDTFSNCKALAKKTKCKTEWIKSKCPATCFCHNKII	18	13	8	39
	Cysteine-rich venom protein 2	Q8UW11	MIAFIVLLSLAAVLQQSSGTVDFASESSNKKDYQREIVDKHNALRRSVKPTARNMLQMKWNSHAAQNAKRSADRCTFAHSPEHTRTVGKFRCGENIFMSSQPFAWSGVVQAWYDEIKNFVYGIGAKPPGSVIGHYTQIVWYKSHLLGCASAKCSSTKYLYVCQYCPAGNIRSSIATPYKSGPSCGDCPSACVNGLCTNPCEYEDAYTNCNDLVKERKCQTEWIKSQCPATCFCHNKII	9	5	2	16
	Alpha-elapitoxin-Lh2a	Q8UW28	MKTLLLTLVVVTIVCLDLGDSLSCYLGYKRSQTCPPGEKVCFVKSWCDAFCGSRGKRIEMGCAATCPTVKDGIDITCCATDNCNTYANWGSG	0	0	0	0
	Long neurotoxin 1	Q8UW29	MKILLLTLVVVTIVCLDLAYTRTCFRTPYKPETCPPGQNLCYKKSWCDAFCSSRGKVIELGCTAKCPTVKDGKDITCCATDNCNTVANWKSR	5	3	0	8
	Long neurotoxin 2	A3FM53	MKILLLTLVVVTIVCLDLAYTRTCYRTHPYKPETCPPGQNLCYKKSWCDAFCSSRGKVIELGCTAKCPTVKHGKDINCCATDNCNTVANWKSR	5	3	1	9
Hydrophis ornatus	Short neurotoxin 1	P68413	MTCCNQQSSQPKTTTNCAGNSCYKKTWSDHRGTIIERGCGCPQVKSGIKLECCHTNECNN	0	0	0	0
Hydrophis cyanocinctus	Short neurotoxin 2 (Fragment)	P62376	PLLLTLVVVTIVCLDLGYTMTCCNQQSSQPKTTTNCAESSCYKKTWSDHRGTRIERGCGCPQVKSGIKLECCHTNECNN	2	1	0	3

	Short neurotoxin 1	P25494	MTCCNQQSSQPKTTTNCAESSCYKKTWSDHRGTRIERGCGCPQVKKGIKLECCHTNECNN	3	1	0	4
Hydrophis schistosus	Short neurotoxin 1	P68415	MTCCNQQSSQPKTTTNCAESSCYKKTWSDHRGTRIERGCGCPQVKPGIKLECCHTNECNN	2	1	1	4
Hydrophis platurus	Pelamitoxin a	P62388	MTCCNQQSSQPKTTTNCAESSCYKKTWSDHRGTRIERGCGCPQVKSGIKLECCHTNECNN	2	1	1	4
Conus textile	Conotoxin tx3c	P58846	MFKLGVLLTICLLLFSLNAVPLDGDQPADQPAERLLDDISFENNPFYDPAKRCCRTCFGCTPCCG	1	0	0	1
	Alpha-conotoxin TxID (Fragment)	K8DWB5	FDGRNAAGNDKMSALMALTTRGCCSHPVCSAMSPICG	0	0	0	0
	Epsilon-conotoxin TxVA	P81755	MRCFPVFIILLLLIASAPCFDARTKTDDDVPLSSLRDNLKRTIRTRLNIRECCEDGWCCTAAPLTGR	9	9	2	20
	Delta-conotoxin TxVIA	Q9U655	MKLTCMMIVAVLFLTAWTFATADDSGNGLENLFSNAHHQMKNPEASKLNKRWCKQSGEMCNLLDQNCCDGYCIVLVCT	5	1	1	7
	Alpha-conotoxin TxIA	P0DM21	MFTVFLLVVLATAVVSFTSDRASDDGKAAASDLITLTIKGCCSRPPCIANNPDLCG	1	0	0	1
	Conotoxin elongated-tx3a-a	Q9BH73	MLKMGVVLFIFLVLFPLATLQLDADQPVERYAENKQLLSPDERREIILHALGTRCCSWDVCDHPSCTCCG	0	0	0	0
	Conotoxin Tx3.5-a	P0C1N7	MSKLGVLLTICLLLFPLTALPLDGDQPADQAAERMQAEQHPLFDQKRRCCKFPCPDSCRYLCCG	8	6	1	15
	Conotoxin tx9a	Q9GU58	MHLSLARSAVLMLLLLFALGNFVVVQSGQITRDVDNGQLTDNRRNLQSKWKPVSLYMSRRGCNNSCQEHSDCESHCICTFRGCGAVNG	6	3	1	10
	Gamma-conotoxin-like TxVIIA	P24160	MEKLTILLLVAAVLMSTQAMFQGDGEKSRKAEINFSETRKLARNKQKRCGGYSTYCEVDSECCSDNCVRSYCTLFG	8	7	3	18
	Conotoxin tx3h	P0C1N6	MLKMGVVLFIFLVLFPLATLQLDADQPVERYAENKQLLNPDERREILLPALRKFCCDSNWCHISDCECCYG	4	1	1	6
	TxMEKL-P2	Q9BPA9	MEKLTILLLVAAVLTSTQALIQGGGDERQKAKINFLSRSDRDCRGYDAPCSSGAPCCDWWTCSARTNRCF	3	0	0	3
	TxMMSK-03	Q9BPJ7	MSKLGALLIICLLLFPLTAVPMDGDQPADRPAERMQDDISFEQHPMFDATRRCCNAGFCRFGCTPCCY	3	0	0	3
	Alpha-conotoxin-like Tx1.2	P0DPL9	MFTVFLLVVLATTVVSFTSGRSTFRGRNAAAKASGLVSLTDRRPQCCSHPACNVDHPEICR	7	5	0	12
	Alpha-conotoxin TxIB (Fragment)	K4RNX9	FDGRNTSANNKATDLMALPVRGCCSDPPCRNKHPDLCGGRR	1	0	0	1
	Conotoxin tx3b	P0C1N8	MSKLGALLTICLLLFSLTAVPLDGDQHADQPAQRLQDRIPTEDHPLFDPNKRCCPPVACNMGCKPCCG	2	0	0	2
	Textile convulsant peptide	P58845	NCPYCVVYCCPPAYCEASGCRPP	0	0	0	0
	Cysteine-rich venom protein	Q7YT83	MLSTMQTVGAVLMLSIVLVAGRKRHHCDSKYYELTPAHTMCLTDKPNAVAVPLTQETEHEILEMHNKIRADVTDAANMLKMEWDERLATVAQKWAMQCILGHDSGRRGEPDLPGSVGQNVAWSSGDLTFLGAVQMWADEIVDFQYGVWTDGTGHYIQQVFAGAS	11	4	0	15

			RIGCGQSACGNNKYFVCNYYKGTMGDEPYQLGRPCSQCRSSCQHIRGSQGRWGSL CDCTNGPDACFNGGIFNINTCQCECSGIWGGADCQEKHCPNEDFDDMCRYPDALR RPQHWCQYDNFQSDCPILCGYCPNPN				
	Conotoxin tx5c	P86259	KPCCSIHDNSCCGI	0	0	0	0
	Conotoxin Gla(1)-TxVI	P58922	MEKLTILLLVAAVLMSTQALVERAGENHSKENINFLLKRKRAADRGMWGECKDGLTT CLAPSECCSEDCEGSCTMW	11	8	3	22
	Omega-conotoxin TxVII	P56714	MKLTCMMIVAVLFLTAWTFATADDSGNGLENLFPKAHHEMKNPEASKLNKRCKQA DEPCDVFSLDCCTGICLGVCMW	5	1	1	7
	Conotoxin Gla(2)-TxVI/A	Q9BPB1	MQKLIILLLVAAVLMSTQALFQEKRPMKKIDFLSKGKTDAEKQQKRSCSDDWQYCES PTDCCSWDCDVVCSG	8	4	1	13
	Conotoxin 3	Q3YEH7	MRCLPVFVILLLLIASVPSDAVQLKTKDDMPLPSFNGNARRTPRMLSNKRICCYPNVW CCD	8	6	4	18
	Conotoxin Gla(2)-TxVI/B	P0CJ21	MEKLIILLLVAAVLMSTQALFQEKRTMKKIDFLSKGKADAEKQRKRNCSDDWQYCES PSDCCSWDCDVVCSG	11	4	4	19
	Omega-conotoxin-like TxO4	Q9XZL1	MKLTCVVIVAVLFLTAWTFVTAVPHSSNALENLYLKARHEMENPEASKLNTRYDCEPP GNFCGMIKIGPPCCSGWCFFACA	1	0	0	1
	Conotoxin TeAr151	Q3YEE9	MRCLPVFVVLLLLIASAPSVDAQPKTKDDVPLAPLHDNIQNTLQTLRKKVCCRPMQD CCSGK	9	3	0	12
	Conotoxin Gla(3)-TxVI	Q9BPB4	MQKLIILLLVAAVLMSAQAVLQEKRPKEKIKFLSKRKTDAEKQQKRLCPDYTEPCSHAH ECCSWNCYNGHCTG	13	11	12	36
	Conotoxin TeAr154	Q3YEE8	MHCLPVFVILLLLTASGLSVDARPKTEDDVPLSSFRDNTKSTLQRLLKRVNCCPIDESCC S	6	7	1	14
	Omega-conotoxin	P28880	MKLTCVMIVAVLLLTACQLITAEDSRGTQKHRTLRSTARRSKSESTTRCRSSGSPCGVT SICCGRCYRGKCT	12	12	9	33
	Omega-conotoxin	Q9XZK2	MKLTCMVIVAVLLLTACQLITADDSRGTQKHRTLRSKTKLSMSTRCKAAGKPCSRIAY NCCTGSCRSGKCG	13	13	12	38
	Delta-conotoxin	Q9XZK5	MKLTCVMIVAVLFLTTWTFVTADDSRYGLKNLFPKARHEMKNPEASKLNKRDGCSSG GTFCGIHPGLCCSEFCFLWCITFID	4	1	2	7
	Omega-conotoxin	P28881	MKLTCVVIVAVLLLTACQLITADDSRGTQKHRALRSDTKLPMSTRCKLKGQSCRKTSY DCCSGSCGRSGKCG	12	8	5	25
	Conotoxin Gla-TxX	Q5I4E6	MSGHTSVSFLLLSIVALGMVATVICSCDSEFSSEFCERPEESCSCSTHTCCHWARRDQC MKPQRCISAQKGNGRRRLIHMQK	8	3	2	13
	Contryphan-Tx	Q9NDA7	MGKLTILVLVAVALLSTQVMVQGDGDQPADRDAVPRDDNPGGMSEKFLNALQRR GCPWQPYCG	3	0	0	3
	Conotoxin Tx6.6	P0DPM4	MKLTCVMIVAVLFLTAWTLVMADDSNNGLANLFSKLRDEMEDPEGSKLEKKDCQEK WDYCPVPFLGSRYCCDGFICPSFFCA	0	0	0	0
	Conotoxin King-Kong 2	P18513	MKLTCMMIVAVLFLTAWTFVTADDSGNGLENLFSKAHHEMKNPEASNLNKRCAPFL HPCTFFFPNCCNSYCVQFICL	1	1	0	2
	Alpha-conotoxin S2	P28879	MGMRMMFTVFLLVVLATTVVSFPSDRASDGRDDEAKDERSDMHESDRNGRGCCC NPACGPNYGCGTSCSRTL	0	0	0	0

	Alpha-conotoxin S1	P15471	MGMRMMFTVFLLVVLATTVVSFPSDRASDGRDDEAKDERSDMHESDRKEICCNPACGPKYSCGR	0	0	0	0
	Conotoxin King-Kong 1	P18512	MKLTCMMIVAVLFLTAWTFATADDSSNGLENLFSKAHHEMKNPEASKLNKRCIEQFDPCDMIRHTCCVGVCFLMACI	1	1	0	2
	Leu-contryphan-Tx	Q9NDA6	MGKLTILVLVAAVLLSAQVMVQGDGDQPADRKAVPREDNPGGASGKLMDVLRPKKCVLYPWCG	3	1	0	4
	Conorfamide-Tx2	P0DM27	MSGRGFLLLALLLLVTVEATRVEKKHSGILLAWSGPRNRFVRIGRRDMQSPLLSERLRFRALGFRQPSSQKQ	8	6	8	22
	Delta-conotoxin TxVIB	P24159	WCKQSGEMCNVLDQNCCDGYCIVFVCT	0	0	0	0
	Alpha-conotoxin-like Tx1	Q9XZK6	MGMRMMFVVFLLVVLASTVVSSTSGRRAFHGRNAAAKASGLVSLTDRRPECCSDPRCNSSHPELCGGRR	6	4	0	10
	Omega-conotoxin-like TxO1	Q9XZK8	MKLTCVVIVAVLFLTVWTFATADDSGNGLEKLFSNAHHEMKNPEASKLNERCLDAGEVCDIFFPTCCGYCILLFCA	0	0	0	0
	Omega-conotoxin-like TxO6	Q9XZL3	MKLTCVVIVAVLFLTAWTLVMADDSNNGLANLFSKSRDEMEDPEAAKLEKNYCQEKWDYCPVPFLGSRYCCDGLFCTLFFCA	1	0	0	1
	Conotoxin Tx5.1	Q9U700	MCCLPVFVILLLLIASAPSVDAQPKTKDDVPLAPLHDNAKSALQHLNQRCCQTFYWCCVQGK	0	0	0	0
	Con-Ins Tx1	A0A0B5A7N8	MTTSSYFLLVALGLLLYVFQSSFGGEHVCWLGDPNHPQGICGPQVADIVEIRCEEKEAEQGGANNARANTGRTSSLMKRRGFLSLLKKRGKRDEGSPLQRSGRGIVCECCKHHCTKEEFTEYCH	14	12	13	39
	Omega-conotoxin-like TxO5	Q9XZL2	MKLTCMVIVAVLFLTAWTFVTAITSNGLENLFPNAHHEMKNPEASKLNKRCVPYEGPCNWLTQNCCDATCVVFWCL	1	0	0	1
	Omega-conotoxin-like TxO3	Q9XZL0	MKLTCVVIVAVLFLTAWTFVTAVPHSSNALENLYLKAHHEMNNPEASELNKRCYDGGTSCDSGIQCCSGWCIFVCF	0	0	0	0
	Alpha-conotoxin-like Tx2	Q9XZK7	MGMRMMFTVFLLVVLATTVVSFTSGRRTFHGRNAAAKASGLVSLTDRRPECCSHPACNVDHPEICR	5	2	0	7
	Conorfamide-Tx1	P0DM26	MSGRGFLLLALLLLVTVEATKVEKNKPGVLDIPVKSNSDDDSIFRYGRRDMQSPLLSERLRF	3	0	0	3
	Omega-conotoxin-like TxO5	Q9U654	MKLTCMMIVAVLFLTAWTFVTAITSNGLENLFPKAHHEMKNPEASKLNKRCVPYEGPCNWLTQNCCDATCVVFWCL	2	1	0	3
	Gamma-conotoxin-like TxMEKL-0511	Q9BHA0	MEKLTILLLVAAVLLSIQALNQEKHQRAKINLLSKRKPPAERWWRWGGCMAWFGLCSKDSECCSNSCDVTRCELMPFPPDW	15	13	9	37
	Contryphan-R/Tx	Q9NDA5	MGKLTILVLVAAVLLSTQAMAQGDGDQPAARNAVPRDDNPDGPSAKFMNVQRRSGCPWEPWCG	1	0	0	1
	Conotoxin TxXIIIA	Q9BPH1	MRCLPVFVILLLLIASVPSVDAELKAKDDMPQASFHDNAERDQQKKTSDCCFYHNCCC	0	0	0	0
	Conopressin-Tx	P86255	CFIRNCPP	0	0	0	0
	Alpha-conotoxin Tx1C	P86261	GCCSRPPCIANNPDIC	0	0	0	0

	Conotoxin tx3e	P86262	SCCNAGFCRFGCTPCCY	0	0	0	0
	Conotoxin Gla-TxXI	Q5I4E5	MVRVTSVGCFLLVIVSLNLVVLTNACIPEGSSCSSSGSCCHKSCCRWTCNQPCLIPGKRAKLLEFFRQR	2	1	1	4
	Omega-conotoxin-like TeA61	Q3YEF3	MKLTCMMIVAVLFLTAWTFATADDSSNGLGNLFLKAHHEMKNPEASKLNERCLDAGEVCDIFFPTCCGYCILLFCA	0	0	0	0
	Gamma-conotoxin-like TeA53	Q3YEG0	MQKLTILLLVAAVLMSTQALNQEQHQRAKINLLSKRKPPAERWWRWGGCMLWFGRCTKDSECCSNSCDRTYCELARFPSDW	16	14	9	39
	Omega-conotoxin-like TxMKLT1-0211	Q9U648	MKLTCMMIVAVLFLTAWTFVTAVPHSSNALENLYLKAHHEMNNPEDSELNKRCYDGGTSCDSGIQCCSGWCIFVCL	0	0	0	0
	Omega-conotoxin-like TxMKLT1-0223	Q9U645	MKLTCMMIVAVLFLTAWTFVTAVPHSSNALENLYLKARHEMENPEASKLNTRDDCEPPGNFCGMIKIGPPCCSGWCFFACA	1	0	0	1
	Delta-conotoxin-like TxMKLT1-0111	Q9U656	MKLTCMMIVAVLFLTAWTFATADDSGNGLENLFSNAHHQMKNPEASKLNKRWCKQSGEMCNLLDQNCCDGYCIVFVCT	5	1	1	7
	Gamma-conotoxin-like TxMEKL-053	Q9BPB0	MEKLTILLLVAAVLMSTQALNQEQHQRAKINLLSKRKPPAERWWECGIWFSRCTKDSECCSNSCDQTYCELMPFPPDW	14	9	5	28
	Omega-conotoxin-like TxMKLT1-0141	Q9U651	MKLTCMMIVAVLFLTAWTFATADDSSNGLENLFPKAHHEMKNPEASKLNERCLDAGEICDFFFPTCCGYCILLFCA	0	0	0	0
	Omega-conotoxin-like TxMKLT1-0142	Q9U650	MKLTCMMIVAVLFLTAWTFATADDSSNGLENLFLKAHHEMNPEASKLNERCLDAGEICDFFFPTCCGYCILLFCA	0	0	0	0
	Omega-conotoxin-like TxMKLT1-031	Q9U644	MKLTCMMIVAVLFLTAWTLVMADDSNNGLANLFSKSRDEMEDPEASKLEKKDCQEKWDFCPAPFFGSRYCCFGLFCTLFFCA	1	0	0	1
	Conotoxin TxMEKL-021	Q9BHB7	MEKLTILLLVAVVLMSTQALPQGGGEKRPRENIRFLSKRKSNAERWREGSCTSWLATCTDASQCCTGVCYKRAYCALWE	10	8	7	25
	Conotoxin TxMMSK-02	Q9BPJ6	MMSKLGALLTICLLLFSLTAVPLDGDQHADQPAQRLQDRIPTEDHPLFDPNKRCCDDSECSYSCWPCCYG	2	0	0	2
	Conotoxin TxMMSK-01	Q9BPJ1	MMSKLGVLLITCLLLFPLTAVPLDGDQPADQPAERLQDDISSENHPFFDPVKRCCRLLCLSCNPCCG	2	0	0	2
	Conotoxin TxMMSK-06	Q9BPJ4	MMSKLGVLLTICLLLFPHTAVPLDGDQHADQPAERLQDDISSEHHPMLNSIRRREQNQFMSFTSVKLRDSRGERCCGPTACMAGCRPCCG	6	2	0	8
	Conotoxin TxMMSK-04	Q9BPJ5	MMSKLGVLLTICLLLFPLTAVPLDGDQPADRPAERMQDGISSEHHPFFDSVKKKQQCCPPVACNMGCEPCCG	2	0	0	2
	Conotoxin TeAr193	Q3YEH2	MRCLPVFVILLLLIASAPSVDAQPKTKDDIPQASFLDNAKRYLQVLESKRNCCRRQICCGRTK	4	2	2	8
	Conotoxin TxMEKL-011	Q9BPB7	MEKLTILLLVAAVLMSTQALVERAGENRSKENIKFLLKRKRAADRGMWGKCKDGLTTCLAPSECCSGNCEQNCKMW	11	13	9	33
	Conotoxin Tx-D021	Q9BH21	MRCLPVFVILLLLIASTPSVDARAKTRDDMSLASFHDDAKRILQILQDRSGCCVIDSNCCG	1	0	0	1
	Conotoxin Tx11.3	S4UKA9	MKLCVTFLLVLVILPSVTGVKSSERTLSGAALRGDRGTCSGRGQECKHDSDCCGHLCCAGITCQFTYIPCK	0	0	0	0
	Conotoxin Tx-D0111	Q9BPG7	MRCLPVFVILLLLIASTPSDTVPLKTKDDMPQASFHGNARRTLQMLSKKQCCWYFDIS	4	3	1	8

			CCITV				
	Conotoxin Tx6.5	P0DPM3	NCPYCVVYCCPPAYCQASGCRPP	0	0	0	0
	Omega-conotoxin-like SVIA mutant 2	Q9XZL5	MKLTCMVIVAVLLLTACQLITAEDSRGTQKHRTLRSTARRSKSELTTRCRPSGSPCGVTSICCGRCSRGKCT	14	14	10	38
	Conotoxin Tx8.1 (Fragment)	B2CI27	LKMGAMFVLLLLFTLASSHREGDIQARKTHLKSDFYRTLPRFARGCTISCGYEDNRCQGECHCPGKTNCYCTSGHHNKGCGCAC	7	4	4	15
	Omega-conotoxin-like SO-5	Q9XZK4	MKLTCVMIVAVLLLTACQLITADDSRGTQKHRSLRSTTKVSKSTSCMEAGSYCGSTTRICCGYCAYFGKKCIDYPSN	7	7	1	15
	Short neurotoxin 1	P68413	MTCCNQQSSQPKTTTNCAGNSCYKKTWSDHRGTIIERGCGCPQVKSGIKLECCHTNECNN	0	0	0	0
Chanos chanos	toxin MIT1-like	A0A6J2VNJ3	MKSSFLILCCVLMLSSGSSAVITGACERDSQCGGAMCCAVSLWIRSLRMCTPMGQEGEECHPMSHKVPFFGKRLHHTCPCLPNLACIATGEGKYKCLSPYKFQDYYL	6	4	0	10
	stonustoxin subunit beta-like	A0A6J2VWL8	MDSSNCLVLEALGRSLYLGTLYNACSDTLVPGMSLWSNEVIQKETKVNPQPNTSFQVAASESISEKIKLLDVSASVKASFFAGLVEVGGSAHYLNEKTSSKLQCRVTMQHQVNTVFKELMFSELTVQYPDVFKAKEATHVVTGVLYGANAILEFQDTASDASEKQTIQGSLNVMIKKIPLIEISAEGKVDLDDADKEKVKHFSCKFYGDYRLKQNPTTYEEAVLLYKDLPNLLGKDGELAVPLKVWLYPLKNLNDTALQLKHVISESLISQVEKMMDDLHQAEMRANDLLEISKTIKASDICDKLELFNSRLKDFTTVFSQKLAELLPTIREGTAEETALTDLLKSQHASGFNRSEIDQWLDGKETEIGIIKSYIADLKLEIKTPGPELDTFLMQPDVTDVFMFSFTSLKYEEPYLNKITKTTEDLRRGINISTCVQNLSEKTLWYSTPEVRGVLLSSLKIIQGFTFGLSIVTYVSDPEHPGASVRWYHNGICRDPHVTDIPFRKDAIVLTLEPNTAHNRLLLSEGNRKVTHGEDQSYPDNPERFDYWQQVLCKESLTEHCYWEAERSGEGLTIAVTYKGIGRKGESNAYRYELTLDPNTAHKSLCLSDANKTATRGDTQRYRDNSKRFQYWEQVLCKEKLTGRCYWEVEWSGMGAAVGVAYKGITRSDTKIGHNDKSWCVNCGDGSFGGNSFTAWHNYKFVSAPAPAFYSRRVGVFLDYQAGTLSFYSVCPDTHTLTHVHTFHAKFKEPLYAGFWVLETVSLV	8	0	0	8
	stonustoxin subunit beta-like	A0A6J2VWA1	MSLWSDEVIQKETKVNPQPYTNFQVAASESIGEKTKLLDVSASLKASFFAGLVEVGGSAHYLNEKTSSKLQCRVSMQHQVTTVFKELMFSGLEVQYPDVFNMKEATHVVTGVLYGANAILEFENTASDASEKQTVQGTLNVMIKKIPSMEISAEGKVDLSDTDKEKVKNFSCKFYGDYRLKQNPTTYEEAVLLYKDLPNLLGKDGELAVPLKVWLYPLKNLNDIAAQLKHMISESLISQVEKMMEDLHHAEMRTNDLLEISKTIKAKDICDKLELFNCRLKDFTTVFSQKLTELLPTIRDGTAEEKSLTDLLMSQHASGFTRSEMDDWLDGKETEIGTIKSYVTELKLEIKTPGPELDIFLIQPDVVHAFMFTFTSLKYEEPYLNKITKTTEDLRRGINIRLPDQNTPIETPWYLKPGIKETLDFSLTLIQCFPSHSKIISYISDPEHPGASVRWYRNGTCRDPYLMSVPFLKGMSADLTLDPNTAHQFLGLAEGNKKVTRLGPPSGITDSIFGTPQVLSEETLTGLCYWEAECTGDGFSIAVTHKGRKDDHSEFGCDEESWSLRCQGHRYTAHYNNQSTDIFWFTEDEIRIGVYLDCQSGTLSFYNISSDTQRYALIYTFQSCKFTGPLYAGFGIRGSDTSLCLVDSVDKEDEENLFFFFLSTGLDDIESYRGFV	9	1	1	11

	ly-6/neurotoxin-like protein 1	A0A6J2WIV0	MNKFLLAAVVVVASLLAAEALTCKKCTVGVLGRCIIGSTETCTGNDNNCYTGKAEFNITGALSFYSQGCIASSSCNGTSGTVLGVGYTVTRTCCNTDQCNGASSIQLPVTAALGAALLASVWGSYL	1	0	0	1
	toxin MIT1-like	A0A6J2VNJ3	MKSSFLILCCVLMLSSGSSAVITGACERDSQCGGAMCCAVSLWIRSLRMCTPMGQEGEECHPMSHKVPFFGKRLHHTCPCLPNLACIATGEGKYKCLSPYKFQDYYL	6	3	0	9
Hydrophis lapemoides	Short neurotoxin 1	P01437	MTCCNQQSSQPKTTTNCAESSCYKKTWRDFRGTRIERGCGCPQVKPGIKLECCHTNECNN	5	4	1	10
Plotosus lineatus	PL-toxin II	F2ZAL6	MSYLAGILVIGGRGGSSFRFNGLGNGSTVRKIWVWAGGWQIKSIKVWLTDGRVEQFGSPSGSYSEFEFQDGECMTSLSLWGNGAGTRLGAIKFKTNRSREFFAHMTDWGLKTEYPIDVGSGICVGVLGRAASDIDCFGFMFINTIKSTKLRNIEYPSLHSEIPEVAVEELKSMTYNNTTSETQEYKIETSKKITKTSSWSVSNNLESTLSIEVSAGIPEVADVTTGFAFTVSTESTRSLENSEERTETLSFPVKVAPGKSVDVEITIGRAAFDLPYKGTIEITCYNGSVLRFPTSGTYKGVTYTEAETSLVEKDL	6	3	0	9
	PL-toxin I	F2ZAL5	MSYLAGIHVIGGQGGSNFDFNGTNNGSTVRKIWVWAAGWQIKSMKIWLTDGREQQFGNPAGNHSEFEFQDGECMTSLSLWGNGAGTRLGAIKFKTNRSREFFAHMTDWGLKTEYPIDVGSGICVGVTGSAGSDIDCLGFMFINTVKSTKLRNVEYPTLHSEIPKVAVEELKSMTYHNNTSETQEYKIETSKKITKTSSWSVTNKLEFTFSFEVSAGIPEVVDVKTGFAFTVGTESTRSLANSEERTEVLSFPIKVAPGKTVDVDITIGRAAFDLPYKGTVEITCYNGSVLTFPTSGTYKGVTYTEAKTTVNESKKSL	7	3	0	10
Scorpaenopsis oxycephala	Tx alpha-subunit	A0A068BD77	MSSDIMIMAGLGRPFTLGMLYDARREKLIPGFSLFGDETLQQYATSTPQRSSEFQIVASDSVESKSNLMDIEASLGVSFLGGLVEVGGSAKYLNNTKKYQNQSRVTLKYKVTTTYQQFKAPPGKVNVQQTAITDKDVATHVVTAILFGANAFFVFDSGKLEDSNLQDIQGKMEAVIKKIPTVSIEGFGSVQLTDEEKSLASNLSCKFHGDFLLESLPTTFEDAVMTYQKLPELLGEEATDAVPMKVWLVPLKKFYSQADLLVRDISQGIVRKVHSILEDLHEQRRRCNEAMDDRLVKQLPLIYDRVSNFQQIFQDYILTVQQTIAKKLPLIREGTEEESSLEEIIEDRANSPFSNENVNIWLDNIEREIGSLKACAGILEKTQTKFVSNQTELDREVLAENVKHALCFVFTYMERNDPYLRVLSQYLESPDTEITGDVAPSVENNSRMVIKMKQKALAFSEFAKKLEKSRNVHFLAAAITNGKYQGASIYHYKEGSLVTEDFTFPPLAPVKTIKDRSDLLWYACDLTFDPNTAHQQLALSDDNKYVESGKHQKYSKHPERFDNYRQVLCKEPLRGRHYWELEWNGTVIAGVTYLSIGRKNIDIQSVIGFNETSWTFISFPKNGYFIKGTGIELLRVGLPDPDFTRLGVYLDCHASTLSFYKVSSNVLHHFHTIEIKPKLSEPVYAIIRIGDVDRPYHGRARLL	19	1	3	23
	Tx beta-subunit	A0A068BD90	MSSDILVLAALGRPFTLGTLYDARKDKLIPGFTLWEDEVLQENTVESAQPSSVFEITASDSIEDKSSLMDIEASLKASFLGGLVEVGGSAKYLNDTKKFKNQSRVTLQYKATTSFKQLMTNLETKHVEYSEYFQNIEATHVVIGILYGANAFFVFDSDKVDSSKVQDIQGSMEAVIKKIPSVEISGQGAVKLTSEESDITNSFSCKFHGDFHLTSNPTTFEDAVKTYQQLPQMMGKETAVPMTVWLVPMTNFYSEAPQLMADSSTPILRKVRNTLEAMRQLDMRCNDSLEDDTVKLFPLLEKKLKTFQKHYRDYMTNLRRTISQKLQSIRKGDEDESAMLQLFEDKLRSPFNIDSLNMWMECAEREINVLRSCIDIIEEAKHKVVLSQTKLDRELDDSEVKHAVCYIFTYVTDYDPYLNALSDFSKSIKPKKFSPSKKDYWYTSDDVPEMMREKAHHFYNLAKDMENRCVRFLVASIVNPKEEGAAIHYYREGIQIINDFSNPRIPPVETIQDRRELLWYDCELTLDPETAHPALHLSEGNKKAMSGKPQPSDNNPERFDHYQQVLCNEGLSKRHYWEVE	20	8	6	34

			WCGYVRVGITYKAIQRKTSDSVNAVGQNLASWAFDYDPKSGYCHIYGNEKTPVKVASPGFDRLGVYLDWPAGTLSFYMVTSTWVTHLHTFSAKFYEAVYPAFLIGDAQQKTNGQIKLL				
Pardachirus marmoratus	Pardaxin-1 (Fragment)	P81863	GFFALIPGIE	0	0	0	0
	Pardaxin P-4	P81861	GFFALIPKIISSPLFKTLLSAVGSALSSSGGQE	2	1	0	3
	Pardaxin P-5	P81862	GFFALIPKIISSPLFKTLLSAVGSALSSSGDQE	2	1	0	3
Epinephelus coioides	Neoverrucotoxin subunit-like protein (Fragment)	F6KMK9	TNEEPWYFSEEIYSNMRNKAKAFRDFAKAQKNNNRLRILIATIANEKYKGATIYHYKEGILVSEDYTKLDPLGVETITDREVLMGYATDLTLDPNTANCYLTLSEGDKKATHGEPQSYPAHPERFDSQPQVLCKESLTGRHYWEVEWSAGIYGFLQVAVAYKGIERKTESLDSQFGYNTMSWAVGQLYVLPVIYSHYVPTVTAYHNGKQWDTAFPSYGCDKVGVYLDWPAGTLSFYRVSSNTLSHLYTFRTKFTEAVYPGFCAGGPLANVYLSPVQ	10	3	3	16
	Ly-6/neurotoxin-like protein 1	G8G909	MQLFGPLIFLVTLSAAFGLRCHSCSGKSCTGPMTCPQGTDRCAIAESNGAVAKSCMLSALCVSPIKCCETDLCNSATPTGSSVLLLLVSSGIITLFL	0	0	0	0
	Neurotoxin/C59/Ly-6-like protein (Fragment)	D6Q1Q4	ARGTFTHDYSYFQSCYDARTCRELVNSSGYSGRCCSSDLCN	0	0	0	0
Conus betulinus	Kappa-conotoxin BtX	Q9U3Z3	MMFRVTSVGCLLLVIVFLNLVVPTSACRAEGTYCENDSQCCLNECCWGGCGHPCRHPGKRSKLQEFFRQR	2	1	0	3
	Neuropeptide Y1-like conopeptide	P0CJ22	TVSDPPARPAVFHSREELMNYVRELNRYFAIVGRPRF	1	0	0	1
	Neuropeptide Y2-like conopeptide	P0CJ23	TVSDPPARPAVFHSREELMNYVRELNLYFAIVGRPRY	0	0	0	0
	Conotoxin Bt9.2	P81727	GCGGVCAYGESCPSSCNTCYSAQCTAQ	0	0	0	0
	Conotoxin BtIIIA	P58623	CCKQSCTTCMPCCW	0	0	0	0
	BtIIIB	P58624	CCELPCHGCVPCCWP	0	0	0	0
	Conotoxin Bt1.8 (Fragment)	A0A068B6Q6	PDGRNAAAKAFDLITPTVRKGCCSNPACILNNPNQCG	0	0	0	0
	Conotoxin Bt6.5	Q3YEG7	MKLTCVLIIAVLFLTACQLATAKTYSTGRQKHRALRSTDKNIKLSRRCNDPGGSCTRHYHCCQLYCNKQESVCLENEPAF	8	10	9	27

	Delta-conotoxin-like Bt6.4	M9PQ91	MKLTCMVIVAVLFLTAWTSVMADGSINRPDIAEGWQKFFSKARDEMKNRAASELNKRCAGIGSFCGLPGLVDCCSGRCFIVCLP	4	1	1	6
	Conotoxin Bt6.6	Q3YEF9	MEKLTILLLVAAVLMSTQALIQSDGEKRQQAKINFLSXRKSTAESWWEGECKGWSVYCSWDWECCSGECTRYYCELW	2	1	0	3
	Conotoxin Bt15a	B0KZ78	MEKLTILVLVATVLLAIQVLVQSDGEKPLKRRVKQYAAKRLSALMRGPRQCTPRNQRCEGDAECCPNLVCKCFTRPDCQSGYKCDTS	17	13	12	42
	Conotoxin Bt12.1	P58625	RCAHGTYYSNDSQQCLLNCCWWGGGDHCCR	0	0	0	0
	Conotoxin Bt5.1	Q3YEH6	MRGLPVFVILLLLIASEPSVDARPKTKADVPLTSLNDNAKRTLQILRNKRACCPYEPSCCI	10	8	3	21
	Conotoxin Bt11.1	P0C251	MKLCVAFLLVLVILPSVIGGKPSERTLSGATRRGDRRMCLSLGQRCERHSNCCGYLCCFYDKCVVTAIGCGHY	9	6	5	20
	Conotoxin Bt11.4	P0C609	MCLSLGQRCGRHSNCCGYLCCFYDKCVVTAIGCGHY	1	1	0	2
	Conotoxin	A0A142C1B1	MMSKLGVLLTVCLLLFPLTAVPMDGDQPADLPALRMQDFAPEHSPWFDPVRRCCSRDCRVCIPCCPN	5	1	0	6
	Conotoxin (Fragment)	A0A142C1R0	AFLVVTLLLFTATATTGAANRVNAAANGKASDSISLAVRDDCCPDPACRQNHPELCSSG	0	0	0	0
	Conotoxin	A0A142C187	MGMRMMFTVFLLVVLATTVVSFAADRESNHRNRRASNQIPRGLWAECCEDPPCRVNNMEHCPAN	8	6	1	15
	Conotoxin	A0A142C1D4	MMFTVFLLVVLATTVVSFTSGRASGGRNAAAKASNRIARAISSGACCAYPPCFEAYPERCL	5	1	0	6
	Conotoxin	A0A142C1D5	MMFTVFLLVVLATTVVSFTSDRAFRGRNPAANDKRSDLAALSVRRGCCSHPACSVNHPELCGRRR	6	4	1	11
	Alpha-conopeptide Bt1.5	A0A075IR52	MFTVFLLVVLATTVVSFTSGRASGGRNAAAKASNRIALIVRNAECCYYPPCYEAYPEICL	2	0	0	2
	A superfamily conotoxin Bt1.7a	S4UKL3	MGMRMMFIVFLLVVLATTVVSFTSDRASDGGNAAAKASDLIAQTIRGGCCSYPACSVEHQDLCDGRR	0	0	0	0
	M superfamily MMSK group conopeptide Bt3-IP01	H2BKR1	MMSKLGVLLTVCLLLFPLTAVPMDGDQPADLPALRMQDFAPEHSPWFDPVRRCCSQDCRVCIPCCPH	1	0	0	1
	Alpha-conopeptide Bt1.4	A0A075IP10	MFTVFLLVVLATTVVSFTSDRAFRGRNSAANDKRSDLAALSVRRGCCSHPACSVNHPELCGRRR	4	2	0	6

	Alpha-conotoxin Bt1.91	F5BDH1	MGMRMMFTVFLLVVLATTVVSLTLDRASGGRRSGADNMIALLIIRKCCSNPACNRYNPAICD	4	1	1	6
	Alpha-conotoxin Bt1.61	F5BDH0	MGMRMMFTVFLLVVLATTVVSFTSDRASDGRNAAGNEMPDWVIRAIKLTCCSELTCAGNYPNICG	1	0	0	1
	Alpha-conopeptide Bt1.1	A0A075ILT2	MFSVFLLVVLATTVVSSTSGGASGGRKAAAKASNRIALTVRSATCCNYPPCYETYPESL	5	3	1	9
	Conotoxin	A0A142C1P8	MRCLPVFVILLLLIGSAPSVDAQPMAIDNVPVASFDDNAKRTLQILLNKRPCCPRDTWCCGFPR	6	10	2	18
	Conotoxin	A0A142C1C7	MRCLPVFIILLLLVASAPSADVQPKTKNSMTLASLRDFAKKGRKNLWRRSECCIRNFLC	12	12	9	33
	Conotoxin	A0A142C1Q1	MLCLPVFIILLLLVSPAATLPVTSKLHGLLTRRSLKNFWKRNLYLRDEDWVDCCQMGDCCDEK	14	9	6	29
	Conotoxin	A0A142C1D2	MRGLPVFVILLLLIASAPSVDARPKTKADVPLTSLNDNAKRTLQILRNKRACCPYEPSCI	10	8	3	21
	Conotoxin	A0A142C1C8	MRCLPIFIILLLLIPSAASVAQPKTKDDVALASFYDNAKRALQRYWAKSLCCPEDRWCC	6	3	3	12
	Conotoxin	A0A142C1C0	MRCLPVFVILLLLIASAPSVDAQPKTKYDVPLTSLNDNAEGSLQIRWCCPNGPRMCCP	1	1	0	2
	Conotoxin	A0A142C1P9	MLCLPVFIILLLLVSPAATLPVKSKLHGFLTRRSLKDFWKRHFYLRDEDWVDCCQMGDCCDEK	12	8	6	26
	Conotoxin	A0A142C1J1	MRCLPVFVILLLLIGSAPTVDAQPKTNDVSMASFDDNAKRTLQMLRGVQICCPYILWCCLIP	2	0	0	2
	Conotoxin	S4UJY9	MLCLPVFIILLLLVSPAATLPVTSKLHGLLTRRSLKNFWKRNLYLRDEDWVDCCHMPRCCVED	14	9	6	29
	Conotoxin	A0A1P7ZCP5	MRCLPVFVILLLLIASAPSVDAQPKTKYDVPLTSLNDNAEGSLQIRWCCPNGPRFCCP	1	1	0	2
	Conotoxin	S4UKA5	MLCLPVFIILPVLSSAAGFPVESELQRDLTQESPKDLGMRNEHQLLKKVADDCCVGKVGTCCR	2	0	0	2
	Conotoxin	S4UJR6	MLCLPVFIILLLLVSPAATLLVKSKLHGFLTRRSLKDFWKRHFYLRDEDWVDCCQMGDCCDEK	12	8	8	28
	Conotoxin	A0A142C1N1	MEKLTILVLVAAVLLSTQLMVQGDGDQPTDRDAVPRHIYPGGVSGKLMHILQSQSGCPWKPWCG	0	0	0	0

	Conotoxin	A0A142C1N2	MEKLTILVLVAAVLLSTQLMVQGDGDQPTDRDAVPRHIYPIGASGKLMYILQSQSGC PWKPWCG	0	0	0	0
	Conotoxin (Fragment)	A0A142C1Q4	LVVLATTVVSFTSDRASDGGNAAAKASDLIAQTIRGGCCSYPACSVEHQDLCDGRR	0	0	0	0
	Conotoxin (Fragment)	A0A142C1Q5	LVVLATTVVSFTSGHASGGRKAAAKASNRIALTVRSATCCNYPPCYETYPESCL	5	2	1	8
	Conotoxin Bt14.5 (Fragment)	A0A068B6R0	SDGRDATVISTESDVIALDDCRLCMHPDCRFNRGRCRPRA	5	3	2	10
	Conotoxin Bt14.15 (Fragment)	A0A068B6S1	SDGRNAAANVKASDLMARVLEKGCPPVPVPGMHKCVCLKTCR	1	0	0	1
	Conotoxin Bt14.2 (Fragment)	A0A068B2V1	SDGRDAAANDKASEVIARDDCVPCMRPACGIHFGECR	0	0	0	0
	Conotoxin Bt14.17 (Fragment)	A0A068B0Y6	SDGRDAAANDKASEVIARDDCVPCMRPACGIKFGECR	0	0	0	0
	Conotoxin Bt1.11 (Fragment)	A0A068B0X3	PDGRNAAATDGWNAAAKAFGLITPTVRDGCCSNPACILNNPNQCG	0	0	0	0
	Conotoxin Bt1.10 (Fragment)	A0A068B3Z0	PDGRNAAATDGRNAAAKAFGLITPTVRDGCCSNPACILNNPNLCG	0	0	0	0
	Conotoxin Bt1.7 (Fragment)	A0A068B2G2	SDNRNAAADNKAYDLLAMTVGGGCCSFPPCIANNPFCAGRR	0	0	0	0
	Conotoxin Bt14.8 (Fragment)	A0A068B2V6	SDGRDAAANDEASEVIARNECDNCMRSFCSMIYEKCRLKG	0	0	0	0
	Conotoxin Bt14.11 (Fragment)	A0A068B403	SHGRNAAANVKASDLMARVLGIRCPHSVPGMHPCKCNNTCR	0	0	0	0
	Conotoxin Bt14.12 (Fragment)	A0A068B0Y2	SDGRDAAVIYTESDVIARGDCKPCMHPDCRFNPGRCRQRE	3	2	2	7
	Conotoxin Bt14.16 (Fragment)	A0A068B409	SDGRDAAVIYTESDVIARGDCKPCMHPDCRFNPGRCRPRE	4	3	2	9
	Conotoxin Bt14.4 (Fragment)	A0A068B2G8	SDGRNAAVISTESDVIVTCKPCVNPACGINRGKCR	1	0	0	1
	Conotoxin (Fragment)	A0A142C1V7	KYDVPLTSLNDNAKRTLQIHWNKRCCPEKIWCCIIPVA	6	4	2	12

	Conotoxin	A0A142C1G6	MQDFAPEHSPWFDPVRRCCSRDCRVCIPCCPH	6	4	0	10
Conus ferrugineus	Conopeptide Y-Fe1	B3SVF1	MSKLGVVLFVFLLLLPLAAPQPVGDQPADQPADRNAEARGTYLYPFSYYRLWRYFTR FLHKQPYYYVHI	4	1	0	5
Conus caracteristicus	Conotoxin Ca6.2	V5V9Y7	MHTLEMLLLVLLLVPLAPGEGDGQAVGGDRNPSEARRAYKRLLQRPARRMDRGGC TPCGPNLCCSEEFRCGTSTHHQTYGEPACLSY	16	17	14	47
Conus flavidus	Conotoxin Fla16d	V5V893	MHTLEMLLLLLLLLPLALGEGDGQAVAGDRNPSEARSTHEHFLQRLIRLIHGSDCQPC GQYVCCPPWKYAEYRRFT	5	1	0	6
	Alpha-conotoxin-like	U3L010	MGMRMMFTMLLLVVLATTVVSFTLDHAFDGRNIAANNKATDLMALPVRGCCSDPP CRHKHQDLCG	1	0	0	1
	Alpha-conotoxin-like	U3L074	MLLLVVLATTVVSFTLDHAFDGRNTAANNKATDLMALPVRGCCSDPPCRHKHQDLC G	1	0	0	1
	Alpha-conotoxin-like	U3L0H7	MGMRMMFTMLLLVVLATTVVSFTLDHAFDGRNTAANNKATDLMALPVRGCCSDP PCRHKHQDLCG	1	0	0	1
	Alpha-conotoxin-like (Fragment)	A0A1S5TAF9	AANKKASALIAQIADRDCCNFPACAASNPGLCT	0	0	0	0
	A-conotoxin peptide Fla1.6	U3L0F2	MGMRMMFTVFLLVALATTVASFTLDHASNGRNAAADDNMSDWIAQALKRCCGN PPCAHVNCRGRR	3	1	0	4
	A-conotoxin peptide Fla6.2 (Fragment)	U3KZW1	MVLTMFLLVVLATTVVSFTLDHASDSRGTAANDKATDQMALAVRLDWCDDADFLV DHPELCGWDVCCAYPPCRHKHQDCNQGR	1	0	0	1
	A-conotoxin peptide Fla1.1	U3L069	MGMRMIFTVFLLVALATTVASFTLDRASNGRNAAADNKPSDWIALAIKQCCRNPKC SQQGCP	2	1	0	3
	A-conotoxin peptide Fla6.1 (Fragment)	U3L0E9	MGKRMVLTMFLLVVLATTVVSFTLDHASDSRGTAANDKATDQMALAVRLDWCDD ADFLVDHPELCGWDVCCAYPPCRHKHQDCNQGR	1	0	0	1
	A-conotoxin peptide Fla1.7a (Fragment)	U3KZV0	MGMRMMFTVFLLVVLSTTVVSLPVDHASNGRDAAADSNAADQIAQTARDPCCSNP SCAQTHPEICRRMLQNPLNHDMSPSA	1	0	0	1
	A-conotoxin peptide Fla1.8	U3L011	MGMRMMLTVFLLVVLATTVVSDHASDGRNTAAKVKYSKTQEECCPNPPCFAVNSA LCGARR	0	0	0	0
	A-conotoxin peptide Fla1.2	U3KZU9	MGMRMMFTMFVLVVLATTVVSFPLDHASNGRDAAAYDKATPLIARDMEECCSHPA CQGINPDYCD	0	0	0	0
	Conotoxin	U3L0G6	MRCVPVFIILLLLSPSAPSVDAHPKTKDDVPLASFHDDAKRTLQRLWIKALCCYGYAFC CRLGR	4	5	0	9

Conus monile	Kappa-conotoxin Mo1659	P84713	FHGGSWYRFPWGY	1	0	0	1
	Conopressin/conophysin, isoform 3 (Fragment)	A0A4Y5X1A7	ACFIRNCPKGGKRNVDEGPTKPCMFCSFGQCVAPHTCCGEKGCEMGTVDANMCQEENESPIPCHVFGKRCLLNHPGNSHGNCVTYGICCSHDTCTVHLACM	3	0	0	3
	Conophysin-conopressin, isoform 1 (Fragment)	A0A291NVT7	MQMGRPTLLPCLLLLLVLSTQACFIRNCPEGGKRDVHMIQPTKPCMNCSFGQCVGPRVCCGAGRCEIGSTEADRCEEENEDPVPCKVLGQHCVLNNPGNVNGNCVDGGIGICCVDDTCAIHRRCD	3	0	0	3
	Conotoxin Mo1274	P0C2F2	GNWCCSARVCC	0	0	0	0
	Delta/kappa-conotoxin Mo3964	A0A0R4I952	DGECGDKDEPCCGRPDGAKVCNDPWVCILTSSRCENP	0	0	0	0
	Conopressin/conophysin, isoform 2 (Fragment)	A0A4Y5X186	MKCSVLQMSRLSWAMCLMLLMLLLLGTAQGCFIRNCPRGGKRAVDALQPTRQCMSCGPDGVGQCVGPSVCCGLGLGCLMGTPETEVCQKENESSVPCAISGRHCGMDNTGNCVADGICCVEDACSFNSLCRVDTDQEDSVSARQELLTLIRRLLVNRQYD	9	5	2	16
	Contryphan-Mo (Fragment)	A0A1P8NVU1	QGDRDQPADRNAVPRDVNPGRARRKLMKVLRESECPWKPWCG	9	8	9	26
	Conotoxin (Fragment)	A0A0P0C7W0	MLCLPIFILLLLLVSPAAALPAESEVRKYLTQEFRKDFERRAGNPRGNWCCSARVCC	2	2	1	5
Conus striatus	Mu-conotoxin SIIIA	Q86DU6	MMSKLGVLLTVCPLLFPLTALPPDGDQPADRPAERMQDDISSDEHPLFDKRQNCCNGGCSSKWCRDHARCCGR	4	0	0	4
	Con-ikot-ikot	P0CB20	MAMNMSMTLCMFVMVVVAATVIDSTQLQEPDLSRMRRSGPADCCRMKECCTDRVNECLQRYSGREDKFVSFCYQEATVTCGSFNEIVGCCYGYQMCMIRVVKPNSLSGAHEACKTVSCGNPCA	6	4	0	10
	Kappa-conotoxin SIVA	P0C828	MGMRMMFTVFLLVVLATTVVSTPSDRASDGRNAAVHERQKSLVPSVITTCCGYDPGTMCPPCRCTNSCG	0	0	0	0
	Mu-conotoxin SIIIC	P0C349	RHGCCKGPKGCSSRECRPQHCC	4	0	0	4
	Omega-conotoxin SVIA	P28880	MKLTCVMIVAVLLLTACQLITAEDSRGTQKHRTLRSTARRSKSESTTRCRSSGSPCGVTSICCGRCYRGKCT	12	12	9	33
	Omega-conotoxin SO-3	Q9XZK2	MKLTCMVIVAVLLLTACQLITADDSRGTQKHRTLRSKTKLSMSTRCKAAGKPCSRIAYNCCTGSCRSGKCG	14	13	12	39
	Omega-conotoxin SVIB	P28881	MKLTCVVIVAVLLLTACQLITADDSRGTQKHRALRSDTKLPMSTRCKLKGQSCRKTSYDCCSGSCGRSGKCG	12	8	5	25

	Delta-conotoxin SVIE	Q9XZK5	MKLTCVMIVAVLFLTTWTFVTADDSRYGLKNLFPKARHEMKNPEASKLNKRDGCSSG GTFCGIHPGLCCSEFCFLWCITFID	4	1	2	7
	Alpha-conotoxin S2	P28879	MGMRMMFTVFLLVVLATTVVSFPSDRASDGRDDEAKDERSDMHESDRNGRGCCC NPACGPNYGCGTSCSRTL	0	0	0	0
	Alpha-conotoxin S1	P15471	MGMRMMFTVFLLVVLATTVVSFPSDRASDGRDDEAKDERSDMHESDRKEICCNPA CGPKYSCGR	0	0	0	0
	Kunitz-type conkunitzin-S1	P0C1X2	MEGRRFAAVLILTICMLAPGTGTLLPKDRPSLCDLPADSGSGTKAEKRIYYNSARKQCL RFDYTGQGGNENNFRRTYDCQRTCLYT	4	3	1	8
	Contryphan-S	P0C837	MEKLTILVLVAAVLLSTQVMVQGDADQPADRDAVPRDDNAGGTDGKFMNVQRRS GCPWEPWCG	1	0	0	1
	Conopressin-S	P05487	CIIRNCPRG	0	0	0	0
	Mu-conotoxin SIIIB	P0CE77	QNCCNGGCSSKWCKGHARCC	2	0	0	2
	Kappa-conotoxin-like SIVB	P0C829	MGMRMMFTVFLSVVLATTVVSTPSDRASDGRNAAVHERQKELVPSVITTCCGYDPG TMCP<br>PCRCTNSCPTKPKKPGRRND	9	6	0	15
	Alpha-conotoxin SIA	P28878	YCCHPACGKNFDC	0	0	0	0
	Omega-conotoxin-like SVIA mutant 1	Q9XZL4	MKLTCVVIVAVLLLTACQLITAEDSRGAQKHRTLRSTARRSKSELTTRCRPSGSPCGVT SICCGRCYRGKCT	14	13	11	38
	Omega-conotoxin-like SO-4	Q9XZK3	MKLTCMVIVAVLLLTACQLITADDSRGTQKHRSLRSTTKVSKATDCIEAGNYCGPTVM KICCGFCSPYSKICMNYPKN	7	7	2	16
	Omega-conotoxin-like SO-5	Q9XZK4	MKLTCVMIVAVLLLTACQLITADDSRGTQKHRSLRSTTKVSKSTSCMEAGSYCGSTTRI CCGYCAYFGKKCIDYPSN	7	7	1	15
	Delta-conotoxin-like S6.8	B2KJ30	MKLTCMMIVAVLFLTAWTFVTADDSRNGLKNLFPKARHEMKNPDASKLNKRDGCS NAGGFCGIHPGLCCSEICLVWCT	4	1	2	7
	Omega-conotoxin-like 6	Q5K0D3	MKLTCVVIIAVLLLTACQLITADDSRGVQKHRSLRSTTKVSKSTSCMEAGSYCGSTTRIC CGYCAYFGKKCIDYPSN	6	6	0	12
	Omega-conotoxin-like 9	Q5K0D2	MKLTCMMIAAVLFLTTWTFVTADDSRYGLKNLFPKARHEMKNPEASKLNKREGCSS GGTFCGIHPGLCCSEFCFLWCITFID	5	1	2	8
	Omega-conotoxin-like 2	Q5K0D8	MKLTCVVIVAVLLLTACQLITADDSRGTQKHRSLRSTTKVSKAADCIEAGNYCGPTVM KLCCGFCSPYSKICMNYPKN	7	7	2	16

	Omega-conotoxin-like 15	Q5K0D5	MKLTCVVIVAVLLLTACQLITAEDSRGTQKHRTLRSTARRSKSELTTRCRPSGSPCGVTSICCGRCSRGKCT	14	14	10	38
	Conotoxin 3	Q5K0D7	MKLTCVVIVAVLLLTACQLITADDSRGTQEHRALRSDTKLSMLTLRCESYGKPCGIYNDCCNACDPAKKTCT	2	1	0	3
	Kappa-conotoxin-like S4.3	P0C830	QKELVPSKTTTCCGYSPGTMCPSCMCTNTCPPQK	0	0	0	0
	Conotoxin S6.11	P0C834	MEKLTILLLVAAVLMSTQALIQGGLDERQKAKSNFFSKRKSNAESWWEGECRTWNAPCSFTSQCCFGKCAHHRCIAW	6	3	3	12
	Conotoxin S6.10	P0C833	MKLTCVLIIAVLFLTACQLATAKTYSKGRQKHRALRSTDKNIKLTRRCTPDDGACAEPVQCCSTFCNPVTNMCIDWLGIGLSRSVL	14	14	12	40
	Iota-conotoxin-like S11.2 (Fragment)	P0C259	GCKKDRKPCSYQADCCNCCPIGTCAPSTNWILPGCSTGPFMAR	2	0	0	2
	conotoxin S11.3	P69499	MMFRLTSVSCFLLVIVCLNLFQVVLTRRCVPPSRYCTRHRPCCRGTCCSGLCRPMCNLWYGKRATFQE	12	10	9	31
	Omega-conotoxin-like S6.7 (Fragment)	P0C832	KSTSCMEAGSYCGSTTRICCGYCAYSASKNVCDYPSN	0	0	0	0
	Alpha-conotoxin-like	S4UJF7	MGMRMMFTVFLLVVLATTVVSFPSDRASDGRDDEAKDERSDMHESDRNGRGCCCNPACGPNYGCGTSCSRTI	0	0	0	0
	Alpha-conotoxin-like	D4HPH2	MFTVFLLVVLATTVVSFTSDRASDGRDDEAKDERSDMHESDRKGRAYCCHPVCGKNFDCGR	0	0	0	0
	Alpha-conotoxin-like	D4HPH1	MFTVFLLVVLATTVVSFTSDRASDGRDDEAKDERSDMHESDRKGRAYCCHPACGPNYSCGTSCSRTL	0	0	0	0
	Alpha-conotoxin-like (Fragment)	D4HPG8	SDGRDDEAKDERSDMHESDRNGRGCCCNPACGPKYSCGR	0	0	0	0
	Alpha-conotoxin-like (Fragment)	D4HPH0	SDGRDDEAKDERSDMHESDRNGRGCCCNPACGPNYGCGTSCSRPSEPRR	1	0	0	1
	Alpha-conotoxin-like	S4UKB4	MGMRMMFIVFLLVVLATTVVSFPSDRASDGRDDEAKDVDMHESDRKGRAYCCHPACGKNFDCGR	0	0	0	0
	Alpha-conotoxin-like (Fragment)	D4HPG6	SDGRDDEAKDERSDMHESDRKGRAYCCHPACGKNFDCGR	0	0	0	0
	Conotoxin (Fragment)	Q45RU7	GDCSCEGQICKCGYRVSPGKSGCACTCRNAK	1	0	0	1

	Conotoxin S5.1 (Fragment)	Q45RU8	HNDLIRAGLTVCLSENRKRLTCSGLLNMAGSVCCKVDTSCCSSQ	2	0	0	2
	Superfamily conotoxin (Fragment)	D4HPG9	SDGRDDEAKDKRSDMYGSDRSGRCCHPACGRKYNCGR	2	1	0	3
	A superfamily conotoxin SIVAa	S4UKL2	MGMRMMFTVFLLVVLATTVVSTPSDRASDGRNAAVHERQKSLVPSVITTCCGYDPG TMCPPCRCTNSCG	0	0	0	0
	Superfamily conotoxin	D4HPH4	MFTVFLLVVLATNVVSTPSDRASDGRNAAVHERQKSLVPSVITTCCGYDPGTMCPPC RCTNSCG	0	0	0	0
	Superfamily conotoxin	D4HPH3	MFTVFLSVVLATTVVSTPSDRASDGRNAAVHERQKELVPSVITTCCGYDPGTMCPPC RCTNSCPTKPKKPGRRND	6	1	0	7
	A superfamily conotoxin S1.1a	S4UJZ7	MFTVFLLVVLAITVVSFPLDRESDGANAEARTHDHEKHALDRNGCCRNPACESHRCG	2	2	1	5
	A superfamily conotoxin S4.4	S4UJD9	MGMRMMFTVFLSVVLATTVVSTPSDRASDGRNAAVHERQKGLVPSVITTCCGYDP GTMCPPCRCTNSCPKKPKKPGRRND	10	6	0	16
	Conotoxin	Q45RU9	MLCLPVFIMLLLLASPAAPNPLETRTQSDLIRAALEDADAKPDERFLGGIFGGISLLSALI NALSS	0	0	0	0
	Conotoxin	S4UJE7	MRCLPVFVILLLLIASAPSVDAQLKTKDDVPLSSFRGHAKSTLRRLQDKQTCCGYRMCI PCG	6	6	1	13
	Conotoxin	S4UKA0	MRCLPVFIILLLLIASAPCFDARTKTDDDVPLSPLRDNLKRTIRTRLNIRECCEDGWCCT AAPLTGR	9	9	1	19
	Conotoxin	S4UJX8	MRCFPVFVILLLLIASAPSVDARPKTKDGMPLASFHDNATRNLQILCCKHTPACCTGK	2	0	0	2
	Conotoxin	S4UJQ7	MRCLPVFVILLLLIASAPSVDAQPKTKDDVPLPHLHDNIQNTLQTLRKKVCCRPMQDC CSGK	9	3	0	12
Conus kinoshitai	Mu-conotoxin KIIIB (Fragment)	P0C195	KRNGCCNCSSKWCRDHSRCCGR	4	0	0	4
Conus geographus	Conophysin-conopressin	P05486	MTRSAMQMGRLTLVLCLLLLLLLTTQACFIRNCPKGGKRDVDERYLFKACMSCSFGQ CVGPRICCGPRGCEMGTAEANRCIEEDEDPIPCQVVGQHCDLNNPGNIHGNCVANG ICCVDDTCTIHTGCL	4	3	1	8
Conus textile	Omega-conotoxin-like TxO4	Q9XZL1	MKLTCVVIVAVLFLTAWTFVTAVPHSSNALENLYLKARHEMENPEASKLNTRYDCEPP GNFCGMIKIGPPCCSGWCFFACA	1	0	0	1
	Alpha-conotoxin-like Tx1	Q9XZK6	MGMRMMFVVFLLVVLASTVVSSTSGRRAFHGRNAAAKASGLVSLTDRRPECCSDPR CNSSHPELCGGRR	6	4	0	10

	Omega-conotoxin-like TxO6	Q9XZL3	MKLTCVVIVAVLFLTAWTLVMADDSNNGLANLFSKSRDEMEDPEAAKLEKNYCQEK WDYCPVPFLGSRYCCDGLFCTLFFCA	1	0	0	1
	Omega-conotoxin-like TxO1	Q9XZK8	MKLTCVVIVAVLFLTVWTFATADDSGNGLEKLFSNAHHEMKNPEASKLNERCLDAGE VCDIFFPTCCGYCILLFCA	0	0	0	0
	Alpha-conotoxin-like Tx2	Q9XZK7	MGMRMMFTVFLLVVLATTVVSFTSGRRTFHGRNAAAKASGLVSLTDRRPECCSHPA CNVDHPEICR	5	2	0	7
Conus tessulatus	Alpha-conotoxin-like ts14a	P86362	DGCPPHPVPGMHPCMCTNTC	0	0	0	0
	Mu-conotoxin TsIIIA	H2BKS9	MMSKLGVLLTICLLLFPLTAVPLDGDQPADQPAERKQNEQHPLFDQKRGCCRWPCP SRCGMARCCSS	7	4	3	14
	Conotoxin TsMMSK-021	Q9BPJ9	MMSKLGVLLTICLLLFPLTAVPLDGDQHADRPADRMQDISSEQHPLFDPVKRCCDW PCTIGCVPCCLP	1	0	0	1
	Conotoxin TsMMSK-B022	Q9BPI5	MMSKLGVLLTICLLLFPLTAVSLDGDQPADLPELRAQDFAPERSPWFDPVRRCCSQD CRVCIPCCPY	0	0	0	0
	Delta-conotoxin TsVIA	P0DL66	CAAFGSFCGLPGLVDCCSGRCFIVCLL	0	0	0	0
	Conotoxin TsMSGL-2	Q9BP73	MSGLGIMVLTLLLLVFMATSHQDAGEKQATQRDAVNVRRRRSIAGRTTTEECDEYCE DLNKNCCGLSNGEPVCATACLG	10	8	1	19
	Conotoxin TsMSGL-13	Q9BP75	MSGLGIMVLTLLLFMFMATSHQDAGEKQATQRDAINVRRRRSITRRGDEECNEHCE DRNKECCGRTNGHPRCANVCFG	12	11	5	28
	Conotoxin TsMMSK-B022	Q9BPI8	MMSKLGVLLTICLLLFPLTAVQLDGDQPADLPELRAQDFAPERSPWFDPVRRCCSQD CRVCIPCCPY	1	0	0	1
	Conotoxin TsMLKM-011	Q9BH51	MLKMGVVLFVFLVLFPLATLQLDADQPVERYAENKQLVSPYERRQIILHALGQRQCC DWQWCDGACDCCA	0	0	0	0
	Conotoxin TsMEKL-03 (Fragment)	Q9BPC0	VILLMSTQALIQSGVEKRSNKIKALSKRKTTAESWWEGECYGWWTSCSSPEQCCSLN CENIYCRAW	5	4	2	11
	Conotoxin TsMEKL-011	Q9BPB9	MEKLTILLLVAAVLMSTQALIQRGGAKRRKVNFFSIREPGAEDWREGNCTPWLGGCT SPEECCPGNCETYCRAWR	10	7	1	18
	Conotoxin TsMEKL-P012	Q9BPC1	MEKLTILLLLAAVLVLAQALIKKGGGEKRQKEKINFLSKRKTTAESWWEGECSGWSVY CTSDPECCSGECSSYYCELW	8	5	4	17
	Conotoxin Ts-011	Q9BH75	MHCLPVLVILLLLIASTPSVDARPKTKDDVPLASFHGADNANRILRTLWNLRGCCEDKT CCFIG	5	2	0	7

	Conotoxin TsMLKM-012	Q9BPH9	MLKMGVVLFVFLVLFPLATLQLDADQPVERYAENKQLVSPYERRQIILHALGQRDCCVMPWCDGACDCCVSS	0	0	0	0
	Conotoxin TsMLCL-04	Q9BPD9	MLCLPVFIILLLLASPAAPNPLETRIQRDLIRAALEDADMKTNERFLEGVISTIKDFAGKVCCSVSVNFCCPTA	3	0	0	3
	Conotoxin TsMLCL-03	Q9BPE3	MLCLPVFIILLLLASPAAPNPLERRIQSDLIRTALEDADMKTPKGVLSGIMSNLGTVGNMVGGFCCTVYSGCCSEK	2	0	0	2
	Conotoxin TsMLKM-02	Q9BPH4	MLKMGVVLFTFLVLFPLATLQLDADQPVERYAENKQGLNPDESREIILSALRQRDCCEQGWCDGGCDCCQ	1	0	0	1
	Conotoxin TsMRCL-05	Q9BPE5	MHCLPVLVILLLLIASTPSVDARPNPKDDVPLASFHGAVNAKRYLRTLWNSRDCCDPKEPCCFIG	7	4	2	13
	Conotoxin TsMRCL-04	Q9BPF5	MRCLPVFIILLLLIPSAASAAQPETKDDAALASFYDNAKRTLQRHWAKSLCCPEDAWCCSHDEGK	6	2	1	9
	Conotoxin Ts5.5 (Fragment)	Q9BP46	PNPLERRIQSDLIRAALEDADMKTDERSVEGVISTIKDFAVKVCCSVSLKFCCPTA	1	0	0	1
	Conotoxin Ts-03	Q9BPF6	MRCLPVFIILLLLIPSAASVAQPKTKDDVALASFYDNAKRTLQRHWAKSLCCPEDAWCC	6	2	3	11
	Conotoxin TsMLCL-02	Q9BPD7	MLCLPVFIILLLLASPAAPNPLERRIQSDLIRAALEDADMKTEKGILSSIMGTLGKIVGLAP	2	0	0	2
	Conotoxin TsMEKL-02 (Fragment)	Q9BP49	NNPPCCSGYVCEGVYCAVDV	0	0	0	0
	Conotoxin	S4UJR4	MLCLPVFIILLLLASPAAPNPFETKLQSDSTRADADXETDKNLDAVRKAICCDVFPGTDCCMG	1	0	0	1
	Conotoxin	Q9BPD8	MLCLPVFIILLLLASPAAPNPLERRIQSDLIRAVLEDADMKTEKGILSSIMGALGGIVG	2	0	0	2
Conus eburneus	Delta-conotoxin-like ErVIA (Fragment)	A0A0F6QDA5	LNKRCAGIGSFCGLPGLVDCCSGRCFIVCLP	0	0	0	0
Conus abbreviatus	Conotoxin AbVIA (Fragment)	Q9TVY1	VLIIAVLFLTACQLTTAVTSSRGEQKHRALRSTDKKFKVALLCSPPGSYCFGPAACCSNFCSTLSDVCQESWSG	7	8	4	19
	Conotoxin AbVII (Fragment)	Q9TVR4	VLIIAVLFLTACQLTTAETSSRGKQKHRALRSTDKNSRMTKRCTPAGDACDATTECCILFCNLATKKCQVPTFP	13	13	7	33
	Conotoxin AbVID (Fragment)	Q9UA91	VLIIAVLFLTACQLTTAETSSRGEQEHLALRSTDKNSRMTKRCTPRHGVCFYSYFCCSKACNPSSKRCL	9	8	4	21

	Conotoxin AbVIH (Fragment)	Q9UA95	VLIIAVLFLTACQLTTAETSSRGKQKHRALRSTDKDSRMTKRCTPAGDACDATTKCCIPFCNLATKKCQVPTFP	12	10	7	29
	Conotoxin AbVIO (Fragment)	Q9TVQ7	VIIIAVLFLTACQLIATASYARSERKHPDLRLSSRNSKLSKRCLGSRELCVRDTSCCSMSCTNNICF	10	8	6	24
	Conotoxin AbVIG (Fragment)	Q9TVX4	VLIIAVLFLTACQLTTAETSSRGKQKHRALRSTDKNSRMTKRCTAPGGACYAAYTCCSNACNLNTKKCVLS	12	11	8	31
	Conotoxin AbVIN (Fragment)	Q9TVQ6	VIIIAVLFLTACQLIATASYARSERKHPDLRLSSRNSKLSKRCLGSREQCVRDTSCCSMSCTNNICF	10	8	6	24
	Conotoxin AbVIE (Fragment)	Q9UA85	VLIIAVLFLTACQLTTAETSSRGKQKHRALRSTDKYSRMTKHCTPPEVGCLFAYECCSKICWRPRCYPS	13	10	10	33
	Conotoxin AbVIM (Fragment)	Q9UA72	VLIIAVLFLTACQLIATASYARSERKHPDLRLSSRNSKLSKRCLGSGELCVRDTSCCSMSCTNNICF	10	6	5	21
	Conotoxin AbVIB (Fragment)	Q9UA83	VIIIAVLFLTACQLTTAETSSRGKQKHRALRSTDKNSKLTRGCTPPGGACGGHAHCCSQSCDILASTCNA	10	9	8	27
	Conotoxin AbVIF (Fragment)	Q9TVK3	VLIIAVLFLTACQLTTAETSSRGKQKHRALRSTDKNSRMSKRCTPPGGYCYHPDPCCSQVCNFPRKHCL	15	12	10	37
	Conotoxin AbVIC (Fragment)	Q9TVU1	VLIIAVLFLTACQLTTAETSSRGKQKHRALRSTDKNSKLTRGCTPPGGACGGHAHCCSQSCNILASTCNA	10	9	8	28
	Conotoxin AbVIK (Fragment)	Q9UA71	VLIIAVLFLTACQLTTAETSSRGKQKHRALRSTDKNSRMTKRCTPAGGACDATTECCILFCNLATKKCQVPTFP	13	12	7	32
	Conotoxin AbVIJ (Fragment)	Q9UA93	VLIIAVLFLAACQLTTAETSSRGKQKHRALRSTDKNSRMTKRCTPAGDACDATTECCILFCNLATKECQVPAFP	13	13	7	33
	Conotoxin AbVIL (Fragment)	Q9UA86	VLIIAVLFLTACQLTTAETSSRGEQKHRAPRSTDKNSRMTKRCTPGGEACDATTNCCFLTCNLATNKCRSPNFP	12	11	5	28
	Four-loop conotoxin ABVIA (Fragment)	Q9UA79	VLIIAVLFLTACQLTTAETSSRGEQKHRALRSTDKKFKVALLCSPPGSYCFGPAACCSNFCSTLSDVCQESWSG	7	8	3	18
	Four-loop conotoxin (Fragment)	B3FTN1	HCTYHRHCCDLYCNKTTNVCLET	0	0	0	0
Conus pennaceus	Alpha-conotoxin PnIB	P50985	MGMRMMFTVFLLVVLATTVVSFTSDRASDDGNAAASDLIALTIKGCCSLPPCALSNPDYCG	0	0	0	0
	Gamma-conotoxin PnVIIA	P56711	MEKLTILLLVAAVLMSTQAQNQEQRQQAKINFLSKRKPSAERWRRDCTSWFGRCTVNSECCSNSCDQTYCELYAFPSFGA	13	10	9	32

	Omega-conotoxin PnVIA	P56712	MKLTCMMIIAVLFLTAWTFVMADDPRDEPEARDEMNPAASKLNERGCLEVDYFCGIPFANNGLCCSGNCVFVCTPQGK	0	0	0	0
	Mu-conotoxin PnIVB	P58927	MMSKLGVLLIICLLLCPLTAVPQDGDQPADQPAERMQDDISSEHHPFFDPVKRCCKYGWTCWLGCSPCGC	1	0	0	1
	Omega-conotoxin PnVIB	P56713	MKLTCMMIVAVLFLTAWTVVTAEPHSSNVLENLYLKAHHEMENPEASKLNTRDDDCEPPGNFCGMIKIGPPCCSGWCFFACA	1	0	0	1
	Mu-conotoxin pn4c (Fragment)	Q9BP55	DQPAERMQDDISSEHHPFFDPVKRCCKYGWTCVLGCSPCGC	1	0	0	1
	Alpha-conotoxin Pn1.2	P0DM23	MFTVFLLVVLATTVVSFTSDRASDGGNAAMSDLIALTIKGCCSHPPCFLNNPDYCG	0	0	0	0
	Alpha-conotoxin-like PnMGMR-02	Q9BP56	MGMRMMFTVFLLVVLATTVVSFTSDRASDGGNAAASDLIALTIKGCCSRPPCALSNPDYCG	1	0	0	1
	Mu-conotoxin-like PnMKLT1-014	Q9U657	MNLTCMMIVAVLFLTAWTFVMADDSNNGLANLFSKSRYEMEDPEPSKLEKRKTCQRRWDFCPGALVGVITCCGGLICLGVMCI	9	3	1	13
	Conotoxin PnMSGL-03	Q9BP63	MSRLGIMVLTLLLLVFIVTSHQDAGEKQATQRDAINFRWRRSLIRRTATEECEEYCEDEEKTCCGLEDGEPVCATTCLG	10	10	5	25
	Conotoxin PnMSGL-01	Q9BP64	MSGLGIMVLTLLLLVFMETSHQDAGEKQAMQRDAINVRRRRSITRRVSEACEESCEDEEKHCCHENNGVYTCLRYCWG	11	11	6	28
	Conotoxin PnMLKM-011	Q9BPI1	MGVVLFIFLVLFPLATLQLDPDQPVERYAENKQLLNPDERRGIILHALGQRVCCPPESCTDRCLCCLG	0	0	0	0
	Conotoxin PnMKLT1-0121	Q9U660	MKLTCMMIVAVLFLTAWTFATADDPRNRLENFFSKTQHEMKNPEASKLNKRCIAESEPCNIITQNCCDGKCLFFCIQIPE	2	0	0	2
	Conotoxin PnMKLT1-1111	Q9U662	MKLTCMMIVAVLFLTAWTVVTAVPHSNKRLANLYLKARHEMKNPEASNVDKRCFESWVACESPKRCCSHVCLFVCA	3	2	1	6
	Conotoxin PnMEKL-04	Q9BPA6	MEKLTILLLVAAVLMSTQALPQGGGENRLKENIKFLLKRKTAADRGMWGDCDDWLAACTTPSQCCTEVCDGFCRLWE	7	5	3	15
	Conotoxin Pn-014	Q9BH86	MRCLPVFVILLLLIASAPSVDARPKTKDDIPLVSFQDHAKRILQTFESRYDCCKTFECCHWG	2	0	0	2
	Conotoxin PnMRCL-0111	Q9BPF8	MHCLSVFVILLLLTASAPSVDAQPKTEDDVPLSSFHDDLQRTVRTLLDIRMCCLGTSGCCPWG	1	0	0	1
	Chi-conotoxin-like PnMRCL-013	Q9BPE9	MRCLPVFVILLLLTASGPSVDAKVHLKTKGDGPLSSFRDNAKSTLQRLQDKSTCCGYRMCVPCG	1	0	0	1

	Conotoxin PnMLKM-013	Q9BPH5	MLKMGVLLFTFLVLFPLATLQLDADQPVEQYAGNKQDLNPDEKREMILPALRQWQCCTMAWCDSGCLCCE	1	0	0	1
	Conotoxin PnMRCL-0111	Q9BPF4	MRCLPVFIVLLLLIVSAPGFDARPKTEDDVPLSSFHDDLQRTVRTLLDIRMCCLGTSGCCPWG	1	0	0	1
	Conotoxin Pn-B02	Q9BPG8	MRCLPVFIILLLLIASAPSFDALPKTEDNVPLSSFHDNLKRTRRIHLNIRECCSDGWCCPAG	8	7	3	18
	Conotoxin Pn-B01121	Q9BPF3	MCCLPVFVILLLLIASAPSVDALPKTKDDMSLASFHDNAKRTLQILSNKRYCCVYDYSCCLSWG	2	0	0	2
	Conotoxin PnMLCL-01	Q9BPE2	MLCLPVFIILLLLASPAASNPLEKRIQSDLIRAALEDADTKNDPRLLDYVTGACCAGLNFVCCGKK	2	0	0	2
	Conotoxin Pn-B01411	Q9BPE7	MRCFPVFIILLLLMASAPSFDARPKTEDDVPLSSFRDNLKRTLRTLLDPRRCCYETPGCCVIG	12	9	7	28
	Conotoxin PnMRCL-022	Q9BPH2	MRCLPVFVILLLLIASTPSVNARPKTKDLASFHDNAKRTQHIFWSKRNCCIYENWCCEWI	7	2	1	10
	Conotoxin Pn-B0151	Q9BPG0	MRCLPVFVILLLLIASTPSVDALQKTKDDMPLASFHDNVKRILQTLSNKRSCCPTILSCCFV	2	1	1	4
	Conotoxin Pn-B01122	Q9BPF1	MRCLPVFVILLLLIASAPSVDARPKTKDDIPLVSFQDNAKRALQILSNKRYCCYFDYSCCLYLR	3	0	0	3
	Conotoxin	Q9BPF0	MRCLPVFVILLLLIASGPSIDARPKTKDDVPLSSFHARPKTKDDVPLSSFRGNAERTQQRAMEPWTRVV	7	1	1	9
	Conotoxin	Q9BH99	MHCLSVFVILLLLTASAPSVDAQPKTEDGVLLSSFRDNAERTLQKLWNHARDWCDPCPWG	1	0	0	1
Conus coronatus	Alpha-conotoxin-like	S4UKH7	MGMRMMFTVFLLVVLATAAVSFTSDRAYDGGNDAAKAFDWIALTARHECCSYPACAYTHPELCGGRR	0	0	0	0
	A superfamily conotoxin Co1.1	S4UJD2	MGMRMMSTVFLLVVLATAVVSFTLDRASDGGDAAAKAFDLMALTARLDCCDIHACWSAHPELCGGRR	0	0	0	0
	A superfamily conotoxin Co1.3	S4UJN8	MGMRMMFTVFLLVVLATSVVSFNSDRASHGRNAAAKVFKRIALIATRECCANPQCWSKNCR	3	3	0	6
	A superfamily conotoxin Co1.2	S4UKH6	MGMRMMFTVFLLVVLATAVVSSTSDRASDGRNTASKVFDLIALTAREDCCFIDDCWASHPEACGGGMELLRQDPLNRDMPPSA	0	0	0	0
	A superfamily conotoxin Co1.6	S4UJD3	MGMRMMFTMFLLVVLATAVVSYTSDRGPTSDGGNAAAKAFDLMALIARDDCCLVASCWQRYPERCGGGR	2	0	0	2

	Four-loop conotoxin (Fragment)	A9P3W5	VLIIAVLLLTACQLTTAETSSRGKQKHRALRSTDKNSRMTKRCTPAGKACDATATCCVLFCNLVTNKCQVPRFP	14	13	9	36
	Four-loop conotoxin (Fragment)	A9P3W2	VMIVAVLFLTACQLTTAETSSRGKQKHRALRSTDKNSRMTKRCTSPGGACYSASTCCSKACNLTTKRCVLS	14	11	7	32
	Four-loop conotoxin (Fragment)	A9P3W9	VLIVAVLFLTACQLTTAETSSRGKQKHRALRSTDKNSKMTRCTDPGGACGNPGHCCSKFCITTSSTCN	8	8	6	22
Conus frigidus	Contryphan-Fr1	A0A1P8NVT3	MGKLTILVLVAAVLLSTQVMVQGDRDQPADRDAVPRDDKPGGTSGKFMNVLRRSGCPWDPWCG	2	0	0	2
	Alpha-conotoxin-like (Fragment)	A0A1S5TAG0	AASKKASALVAQIADRDCCNDPACAVNNPGLCT	0	0	0	0
