# Supplementary material for: Polypharmacological Cell-Penetrating Peptides from Venomous Marine Animals Based on Immunomodulating, Antimicrobial, and Anticancer Properties
Source: Mar Drugs. 2022 Dec 4;20(12):763. doi: 10.3390/md20120763 (PMC9787916; doi:10.3390/md20120763)
Supplement: Supplementary file 1 [file marinedrugs-20-00763-s001.zip › Table S2.pdf]

Table S2 The sequence of 3505 cell-penetrating peptides with a length of 10, 15, and 20 amino acids in each toxin

Species	Toxin name	CPP (10)		CPP (15)		CPP (20)	
Stichodactyla haddoni	KappaPI-stichotoxin-Shd2a<br>B1B5I8	CPP10-1<br>CPP10-2	KLYFLLCLAL<br>PKCRGYFPRY				
	Delta-stichotoxin-Shd3a<br>B1B5I9	CPP10-3<br>CPP10-4<br>CPP10-5<br>CPP10-6	SVVKRAACKC<br>TAVASCCRKK<br>AVASCCRKKK<br>VASCCRKKKG			CPP20-1	GWEKCTAVYTAVAS<br>CCRKKK
	Kappa-stichotoxin-Shd5a<br>E2S062	CPP10-7<br>CPP10-8<br>CPP10-9<br>CPP10-10<br>CPP10-11<br>CPP10-12<br>CPP10-13	ENGFQKRRSC<br>NGFQKRRSCI<br>FQCKHSMKYR<br>KHSMKYRLSF<br>MKYRLSFCRK<br>KYRLSFCRKT<br>RLSFCRKTCG	CPP15-1<br>CPP15-2<br>CPP15-3<br>CPP15-4<br>CPP15-5	NGFQKRRSCIDTIPQ<br>QCKHSMKYRLSFCRK<br>CKHSMKYRLSFCRKT<br>KHSMKYRLSFCRKTC<br>HSMKYRLSFCRKTCG	CPP20-2	RCTAFQCKHSMKYR<br>LSFCRK
Chironex fleckeri	Toxin CfTX-1<br>A7L035	CPP10-14<br>CPP10-15<br>CPP10-16<br>CPP10-17<br>CPP10-18<br>CPP10-19<br>CPP10-20<br>CPP10-21	KIDAKMPSGK<br>VQRIKYIKPK<br>QRIKYIKPKT<br>RIKYIKPKTE<br>FLSYLYPREK<br>KTTQHDPKIC<br>GNYILFTHKR<br>KREIYLLSSK	CPP15-6<br>CPP15-7	FIAMVVQRIKYIKPK<br>QRIKYIKPKTESEIK	CPP20-3	YQGVRFIAMVVQRI<br>KYIKPK
	Toxin CfTX-2<br>A7L036	CPP10-22<br>CPP10-23<br>CPP10-24<br>CPP10-25<br>CPP10-26<br>CPP10-27<br>CPP10-28<br>CPP10-29<br>CPP10-30<br>CPP10-31<br>CPP10-32<br>CPP10-33	SLKAKIDIKK<br>KAKIDIKKPT<br>KIDIKKPTGK<br>KQLFDKVKSM<br>VQRIKYIKPK<br>QRIKYIKPKT<br>RIKYIKPKTE<br>FLSYLYPKEK<br>DLRVGLKLEK<br>KTYGRDPRTC<br>KGQKAYLLST<br>QKAYLLSTKK	CPP15-8<br>CPP15-9<br>CPP15-10<br>CPP15-11<br>CPP15-12<br>CPP15-13<br>CPP15-14<br>CPP15-15<br>CPP15-16	SSGLASLKAKIDIKK<br>GLASLKAKIDIKKPT<br>ASLKAKIDIKKPTGK<br>KIDIKKPTGKQLFDK<br>KKPTGKQLFDKVKSM<br>FIAMVVQRIKYIKPK<br>QRIKYIKPKTESEIK<br>KGQKAYLLSTKKWPH<br>GQKAYLLSTKKWPHN	CPP20-4<br>CPP20-5	LKAKIDIKKPTGKQLF<br>DKVK<br>YQGVRFIAMVVQRI<br>KYIKPK

		CPP10-34<br>CPP10-35	KAYLLSTKKW<br>YLLSTKKWPH				
Acanthaster planci	Plancitoxin-1<br>Q75WF2	CPP10-36	WNRQLNLQSK	CPP15-17	SPPWNRQLNLQSKSG		
Pterois volitans	Pvtoxin-b<br>F2ZAF1	CPP10-37<br>CPP10-38<br>CPP10-39<br>CPP10-40<br>CPP10-41<br>CPP10-42<br>CPP10-43<br>CPP10-44<br>CPP10-45<br>CPP10-46<br>CPP10-47<br>CPP10-48<br>CPP10-49<br>CPP10-50<br>CPP10-51<br>CPP10-52<br>CPP10-53<br>CPP10-54<br>CPP10-55<br>CPP10-56<br>CPP10-57<br>CPP10-58	DARKVRLIPG<br>RKVRLIPGFT<br>KYLDDRKKFK<br>DRKKFKNQSR<br>RKKFKNQSRV<br>KKFKNQSRVT<br>SSTPILRKVR<br>STPILRKVRN<br>TPILRKVRNT<br>LDSPTSKPKK<br>DSPTSKPKKL<br>SPTSKPKKLR<br>PTSKPKKLRP<br>TSKPKKLRPS<br>SKPKKLRPST<br>KPKKLRPSTK<br>KKLRPSTKDY<br>KLRPSTKDYW<br>MMRAKAHHFR<br>RAKAHHFRNL<br>NHIFDAKKTK<br>HIFDAKKTKV	CPP15-18<br>CPP15-19<br>CPP15-20<br>CPP15-21<br>CPP15-22<br>CPP15-23<br>CPP15-24<br>CPP15-25<br>CPP15-26<br>CPP15-27<br>CPP15-28<br>CPP15-29<br>CPP15-30<br>CPP15-31	GSAKYLDDRKKFKNQ<br>KYLDDRKKFKNQSRV<br>RKKFKNQSRVTLQYK<br>KLMADSSTPILRKVR<br>MLNLRRTIAEKLQSI<br>SDFLDSPTSKPKKLR<br>DFLDSPTSKPKKLRP<br>FLDSPTSKPKKLRPS<br>LDSPTSKPKKLRPST<br>DSPTSKPKKLRPSTK<br>SPTSKPKKLRPSTKD<br>KPKKLRPSTKDYWYI<br>KAHHFRNLALDMDNR<br>KKTKVNVSSPGFKKL	CPP20-6	KYLDDRKKFKNQSR<br>VTLQYK

	Pvtoxin-a<br>F2ZAF0	CPP10-59<br>CPP10-60<br>CPP10-61<br>CPP10-62<br>CPP10-63<br>CPP10-64<br>CPP10-65<br>CPP10-66<br>CPP10-67<br>CPP10-68<br>CPP10-69<br>CPP10-70<br>CPP10-71<br>CPP10-72<br>CPP10-73<br>CPP10-74<br>CPP10-75<br>CPP10-76<br>CPP10-77<br>CPP10-78<br>CPP10-79<br>CPP10-80<br>CPP10-81<br>CPP10-82<br>CPP10-83<br>CPP10-84<br>CPP10-85<br>CPP10-86<br>CPP10-87	DSRKDRLIPG<br>RKDRLIPGFT<br>KYLDDRKKFK<br>DRKKFKNQSR<br>RKKFKNQSRV<br>KKFKNQSRVT<br>SAITDRFSCK<br>SSISILRRVR<br>SISILRRVRN<br>ISILRRVRNT<br>SILRRVRNTL<br>LDSPDSRPKR<br>DSPDSRPKRT<br>SPDSRPKRTR<br>PDSRPKRTRP<br>DSRPKRTRPP<br>SRPKRTRPPA<br>RPKRTRPPAT<br>PKRTRPPATE<br>DNRRVRFLVA<br>RRVRFLVASI<br>KKAVSGKPKP<br>KAVSGKPKPS<br>VLCKKQLIRR<br>CKKQLIRRHY<br>KKQLIRRHYW<br>KQLIRRHYWE<br>QLIRRHYWEI<br>LIRRHYWEIK	CPP15-32<br>CPP15-33<br>CPP15-34<br>CPP15-35<br>CPP15-36<br>CPP15-37<br>CPP15-38<br>CPP15-39<br>CPP15-40<br>CPP15-41<br>CPP15-42<br>CPP15-43<br>CPP15-44<br>CPP15-45	GSAKYLDDRKKFKNQ<br>KYLDDRKKFKNQSRV<br>RKKFKNQSRVTLQYK<br>KLMADSSISILRRVR<br>SSISILRRVRNTLEA<br>DYLDSPDSRPKRTRP<br>YLDSPDSRPKRTRPP<br>LDSPDSRPKRTRPPA<br>DSPDSRPKRTRPPAT<br>SPDSRPKRTRPPATE<br>DSRPKRTRPPATEYW<br>RPKRTRPPATEYWYA<br>KKQLIRRHYWEIKWS<br>KQLIRRHYWEIKWSG	CPP20-7	KYLDDRKKFKNQSR<br>VTLQYK
Scorpaena<br>plumieri	Cytolytic toxin-<br>alpha<br>A0A2P1BRQ0	CPP10-88<br>CPP10-89<br>CPP10-90<br>CPP10-91<br>CPP10-92<br>CPP10-93<br>CPP10-94<br>CPP10-95<br>CPP10-96<br>CPP10-97<br>CPP10-98<br>CPP10-99<br>CPP10-100	YDARREKLIP<br>KVTTTFKQFK<br>LEDLHKLKRR<br>DLHKLKRRAN<br>HKLKRRANDS<br>KLKRRANDSL<br>KKLKNFQKNY<br>MTTFRRTISQ<br>FRRTISQKLQ<br>LKSPNSRPAR<br>KSPNSRPARV<br>SPNSRPARVR<br>PNSRPARVRP	CPP15-46<br>CPP15-47<br>CPP15-48<br>CPP15-49<br>CPP15-50<br>CPP15-51<br>CPP15-52<br>CPP15-53<br>CPP15-54<br>CPP15-55<br>CPP15-56	LQYKVTTTFKQFKAP<br>KVHSILEDLHKLKRR<br>YMTTFRRTISQKLQS<br>MTTFRRTISQKLQSI<br>FRRTISQKLQSIRKG<br>ALRDYLKSPNSRPAR<br>DYLKSPNSRPARVRP<br>YLKSPNSRPARVRPV<br>LKSPNSRPARVRPVV<br>KSPNSRPARVRPVVT<br>KAHLFRSLAKDMENR	CPP20-8<br>CPP20-9<br>CPP20-10<br>CPP20-11<br>CPP20-12<br>CPP20-13<br>CPP20-14	MAGLGRPFTLGFLY<br>DARREK<br>GRPFTLGFLYDARRE<br>KLIPG<br>QGLVRKVHSILEDLH<br>KLKRR<br>GLVRKVHSILEDLHK<br>LKRRA<br>KKLKNFQKNYSDYM<br>TTFRRT<br>ALRDYLKSPNSRPAR<br>VRPVV<br>KGARRSVSYYFISDLA

		CPP10-101<br>CPP10-102<br>CPP10-103<br>CPP10-104<br>CPP10-105	NSRPARVRPV<br>SRPARVRPVV<br>REKAHLFRSL<br>KAHLFRSLAK<br>TSWTLMFIPK				FPPL
	Cytolytic toxin-beta A0A2P1BRP3	CPP10-106<br>CPP10-107<br>CPP10-108<br>CPP10-109<br>CPP10-110<br>CPP10-111<br>CPP10-112<br>CPP10-113<br>CPP10-114<br>CPP10-115<br>CPP10-116<br>CPP10-117<br>CPP10-118<br>CPP10-119<br>CPP10-120<br>CPP10-121<br>CPP10-122<br>CPP10-123<br>CPP10-124<br>CPP10-125<br>CPP10-126<br>CPP10-127<br>CPP10-128<br>CPP10-129<br>CPP10-130<br>CPP10-131<br>CPP10-132<br>CPP10-133<br>CPP10-134<br>CPP10-135	GALYDARKDK<br>KYLNDTKKFK<br>KKFKNQSRVT<br>SSTPILRKVR<br>STPILRKVRN<br>TPILRKVRNT<br>LKKKLKTFQK<br>KKKLKTFQKH<br>KKLKTFQKHY<br>RLHVNPFRKN<br>GKGTKMKLQC<br>KGTKMKLQCL<br>MKLQCLPTFR<br>CLPTFRNKLR<br>LPTFRNKLRS<br>PTFRNKLRSP<br>FRNKLRSPSN<br>RNKLRSPSNI<br>DFSKSIKPKK<br>FSKSIKPKKY<br>SKSIKPKKYS<br>KSIKPKKYSP<br>IKPKKYSPSK<br>KPKKYSPSKK<br>KKYSPSKKDY<br>KKAMSGKPQP<br>GITYKGIQRK<br>HHIYNNKKVR<br>KKVRVKVASP<br>KNANGQIKLK	CPP15-57<br>CPP15-58<br>CPP15-59<br>CPP15-60<br>CPP15-61<br>CPP15-62<br>CPP15-63<br>CPP15-64<br>CPP15-65<br>CPP15-66<br>CPP15-67<br>CPP15-68	GSAKYLNDTKKFKNQ<br>LKKKLKTFQKHYERL<br>GKGTKMKLQCLPTFR<br>MKLQCLPTFRNKLRS<br>KLQCLPTFRNKLRSP<br>CLPTFRNKLRSPSNI<br>LNALSDFSKSIKPKK<br>DFSKSIKPKKYSPSK<br>FSKSIKPKKYSPSKK<br>SKSIKPKKYSPSKKD<br>KSIKPKKYSPSKKDY<br>KPKKYSPSKKDYWYT	CPP20-15<br>CPP20-16<br>CPP20-17	KYLNDTKKFKNQSRV<br>TLQYK<br>KLKTFQKHYERLHVN<br>PFRKN<br>KGTKMKLQCLPTFR<br>NKLRSP

Synanceia horrida	Stonustoxin subunit alpha<br>Q98989	CPP10-136<br>CPP10-137<br>CPP10-138<br>CPP10-139<br>CPP10-140<br>CPP10-141<br>CPP10-142<br>CPP10-143<br>CPP10-144<br>CPP10-145<br>CPP10-146<br>CPP10-147<br>CPP10-148<br>CPP10-149<br>CPP10-150<br>CPP10-151<br>CPP10-152<br>CPP10-153<br>CPP10-154<br>CPP10-155<br>CPP10-156<br>CPP10-157<br>CPP10-158<br>CPP10-159<br>CPP10-160<br>CPP10-161<br>CPP10-162<br>CPP10-163<br>CPP10-164<br>CPP10-165	MLYDARREKL<br>YDARREKLIP<br>NQSRVTLKYK<br>QSRVTLKYKA<br>SRVTLKYKAT<br>KMEAAIKKIP<br>PMKVWLAPLK<br>MKVWLAPLKS<br>SKVRRIHTTL<br>LEELHKLKRR<br>EELHKLKRRA<br>ELHKLKRRAN<br>HKLKRRANEA<br>KKIAEKLPLV<br>KIAEKLPLVR<br>KIIDDRAQSP<br>KMQQRAQTFC<br>RMPFVQGYKK<br>KKRSDLLWYA<br>HGKRQNYPKH<br>GKRQNYPKHP<br>RQNYPKHPER<br>KIGYIERHKK<br>IGYIERHKKR<br>GYIERHKKRE<br>YIERHKKREY<br>RHKKREYNVR<br>KKREYNVRAP<br>RAPNPGFKRL<br>KRLGLFLDWR	CPP15-69<br>CPP15-70<br>CPP15-71<br>CPP15-72<br>CPP15-73<br>CPP15-74<br>CPP15-75<br>CPP15-76<br>CPP15-77<br>CPP15-78	TKKYQNQSRVTLKYK<br>KKYQNQSRVTLKYKA<br>RIHTTLEELHKLKRR<br>IHTTLEELHKLKRRA<br>KIIDDRAQSPFSNEK<br>SEHGKRQNYPKHPER<br>KIGYIERHKKREYNV<br>IGYIERHKKREYNVR<br>GYIERHKKREYNVRA<br>RHKKREYNVRAPNPG	CPP20-18<br>CPP20-19<br>CPP20-20<br>CPP20-21<br>CPP20-22<br>CPP20-23<br>CPP20-24<br>CPP20-25	MPALGRPFTLGMLY<br>DARREK<br>KYLNNTKKYQNQSR<br>VTLKYK<br>VSKVRRIHTTLEELHK<br>LKRR<br>SKVRRIHTTLEELHKL<br>KRRA<br>KVRRIHTTLEELHKLK<br>RRAN<br>KIGYIERHKKREYNV<br>RAPNP<br>ERHKKREYNVRAPN<br>PGFKRL<br>RHKKREYNVRAPNP<br>GFKRLG
	Stonustoxin subunit beta<br>Q91453	CPP10-166<br>CPP10-167<br>CPP10-168<br>CPP10-169<br>CPP10-170<br>CPP10-171<br>CPP10-172<br>CPP10-173<br>CPP10-174<br>CPP10-175<br>CPP10-176<br>CPP10-177	KYLNNQKKFK<br>LNNQKKFKNQ<br>NQKKFKNQSR<br>KKFKNQSRVT<br>SSTPILRKVR<br>STPILRKVRN<br>TPILRKVRNT<br>KLQATIAKKL<br>MDILTKAKPK<br>DILTKAKPKV<br>LDSPQSRPKK<br>DSPQSRPKKL	CPP15-79<br>CPP15-80<br>CPP15-81<br>CPP15-82<br>CPP15-83<br>CPP15-84<br>CPP15-85<br>CPP15-86<br>CPP15-87<br>CPP15-88<br>CPP15-89<br>CPP15-90	GSAKYLNNQKKFKNQ<br>QKKFKNQSRVTLQYK<br>KLQATIAKKLFAIRS<br>LRSCMDILTKAKPKV<br>NEFLDSPQSRPKKLR<br>EFLDSPQSRPKKLRP<br>FLDSPQSRPKKLRPS<br>LDSPQSRPKKLRPSP<br>DSPQSRPKKLRPSPK<br>SPQSRPKKLRPSPKD<br>PQSRPKKLRPSPKDY<br>SRPKKLRPSPKDYWY	CPP20-26<br>CPP20-27<br>CPP20-28<br>CPP20-29	KYLNNQKKFKNQSR<br>VTLQYK<br>QKICDDHMSKLQATI<br>AKKLF<br>REINVLRSCMDILTK<br>AKPKV<br>NEFLDSPQSRPKKLR<br>PSPKD

		CPP10-178<br>CPP10-179<br>CPP10-180<br>CPP10-181<br>CPP10-182<br>CPP10-183<br>CPP10-184<br>CPP10-185<br>CPP10-186<br>CPP10-187	SPQSRPKKLR<br>PQSRPKKLRP<br>QSRPKKLRPS<br>SRPKKLRPSP<br>RPKKLRPSPK<br>PKKLRPSPKD<br>KKLRPSPKDY<br>KLRPSPKDYW<br>KAYLFRNLAK<br>QQIHNSKKTR	CPP15-91<br>CPP15-92	RPKKLRPSPKDYWYS<br>KAYLFRNLAKEMNNR		
Synanceia verrucosa	Neoverrucotoxin subunit alpha A0ZSK3	CPP10-188<br>CPP10-189<br>CPP10-190<br>CPP10-191<br>CPP10-192<br>CPP10-193<br>CPP10-194<br>CPP10-195<br>CPP10-196<br>CPP10-197<br>CPP10-198<br>CPP10-199<br>CPP10-200<br>CPP10-201<br>CPP10-202<br>CPP10-203<br>CPP10-204<br>CPP10-205<br>CPP10-206<br>CPP10-207<br>CPP10-208<br>CPP10-209<br>CPP10-210<br>CPP10-211<br>CPP10-212	MLYDTRREKL<br>YDTRREKLIP<br>NQSRVTLKYK<br>QSRVTLKYKA<br>SRVTLKYKAT<br>PMKVWLVPLK<br>MKVWLVPLKK<br>KVWLVPLKKF<br>PLKKFFSKAK<br>KKFFSKAKLL<br>KFFSKAKLLT<br>SKVRRIHTTL<br>ELYKLKRRAN<br>YKLKRRANEA<br>KKIAEKLPLV<br>KIAEKLPLVR<br>KIIDDRAKSP<br>TRVVLKMKQR<br>KMKQRAQTFC<br>KQRAQTFCDH<br>RMPFVQGYKK<br>KKRSDLLWYA<br>HGKRQNYPKH<br>GKRQNYPKHP<br>RQNYPKHPER	CPP15-93<br>CPP15-94<br>CPP15-95<br>CPP15-96<br>CPP15-97<br>CPP15-98<br>CPP15-99<br>CPP15-100<br>CPP15-101<br>CPP15-102<br>CPP15-103	TKKYQNQSRVTLKYK<br>KKYQNQSRVTLKYKA<br>MKVWLVPLKKFFSKA<br>KVWLVPLKKFFSKAK<br>WLVPLKKFFSKAKLL<br>LVPLKKFFSKAKLLT<br>IHTTLEELYKLKRRA<br>KWCFSTRVVLKMKQR<br>WCFSTRVVLKMKQRA<br>SEHGKRQNYPKHPER<br>GKRQNYPKHPERFLC	CPP20-30<br>CPP20-31<br>CPP20-32	KYLNNTKKYQNQSR<br>VTLKYK<br>SKVRRIHTTLEELYKL<br>KRRA<br>KVRRIHTTLEELYKLK<br>RRAN
	Neoverrucotoxin subunit beta A0ZSK4	CPP10-213<br>CPP10-214<br>CPP10-215<br>CPP10-216<br>CPP10-217<br>CPP10-218<br>CPP10-219	KYLNNQKKFK<br>LNNQKKFKNQ<br>NQKKFKNQSR<br>KKFKNQSRVT<br>SSTPILRKVR<br>STPILRKVRN<br>TPILRKVRNT	CPP15-104<br>CPP15-105<br>CPP15-106<br>CPP15-107<br>CPP15-108<br>CPP15-109<br>CPP15-110	GSAKYLNNQKKFKNQ<br>QKKFKNQSRVTLQYK<br>KLQATIAKKLFAIRS<br>LKSCMDILTKAKPKV<br>NDFLDSPQSRPKKLR<br>DFLDSPQSRPKKLRP<br>FLDSPQSRPKKLRPS	CPP20-33<br>CPP20-34<br>CPP20-35<br>CPP20-36<br>CPP20-37	KYLNNQKKFKNQSR<br>VTLQYK<br>KKFKNQSRVTLQYKA<br>TTNFK<br>QIICDDHMSKLQATI<br>AKKLF<br>REINVLKSCMDILTK

		CPP10-220<br>CPP10-221<br>CPP10-222<br>CPP10-223<br>CPP10-224<br>CPP10-225<br>CPP10-226<br>CPP10-227<br>CPP10-228<br>CPP10-229<br>CPP10-230<br>CPP10-231<br>CPP10-232<br>CPP10-233<br>CPP10-234<br>CPP10-235<br>CPP10-236<br>CPP10-237	KLQATIAKKL<br>KSCMDILTKA<br>MDILTKAKPK<br>DILTKAKPKV<br>LDSPQSRPKK<br>DSPQSRPKKL<br>SPQSRPKKLR<br>PQSRPKKLRP<br>QSRPKKLRPS<br>SRPKKLRPSP<br>RPKKLRPSPK<br>PKKLRPSPKD<br>KKLRPSPKDY<br>KLRPSPKDYW<br>MMREKAHLFR<br>REKAHLFRNL<br>KAHLFRNLAK<br>KSWVFEYTKK	CPP15-111<br>CPP15-112<br>CPP15-113<br>CPP15-114<br>CPP15-115<br>CPP15-116<br>CPP15-117	LDSPQSRPKKLRPSP<br>DSPQSRPKKLRPSPK<br>SPQSRPKKLRPSPKD<br>PQSRPKKLRPSPKDY<br>SRPKKLRPSPKDYWY<br>RPKKLRPSPKDYWYS<br>KAHLFRNLAKEMNNR		AKPKV<br>NDFLDSPQSRPKKLR<br>PSPKD
	Verrucotoxin<br>subunit beta<br>Q98993	CPP10-238<br>CPP10-239<br>CPP10-240<br>CPP10-241<br>CPP10-242<br>CPP10-243<br>CPP10-244<br>CPP10-245<br>CPP10-246<br>CPP10-247<br>CPP10-248<br>CPP10-249<br>CPP10-250<br>CPP10-251<br>CPP10-252<br>CPP10-253<br>CPP10-254<br>CPP10-255<br>CPP10-256<br>CPP10-257<br>CPP10-258<br>CPP10-259<br>CPP10-260<br>CPP10-261	KYLNNQKKFK<br>LNNQKKFKNQ<br>NQKKFKNQSR<br>KKFKNQSRVT<br>SSTPILRKVR<br>STPILRKVRN<br>TPILRKVRNT<br>KLQATIAKKL<br>KSCMDILTKA<br>MDILTKAKPK<br>DILTKAKPKV<br>LDSPQSRPKK<br>DSPQSRPKKL<br>SPQSRPKKLR<br>PQSRPKKLRP<br>QSRPKKLRPS<br>SRPKKLRPSP<br>RPKKLRPSPK<br>PKKLRPSPKD<br>KKLRPSPKDY<br>KLRPSPKDYW<br>MMREKAHLFR<br>REKAHLFRNL<br>KAHLFRNLAK	CPP15-118<br>CPP15-119<br>CPP15-120<br>CPP15-121<br>CPP15-122<br>CPP15-123<br>CPP15-124<br>CPP15-125<br>CPP15-126<br>CPP15-127<br>CPP15-128<br>CPP15-129<br>CPP15-130<br>CPP15-131<br>CPP15-132<br>CPP15-133<br>CPP15-134<br>CPP15-135<br>CPP15-136<br>CPP15-137<br>CPP15-138<br>CPP15-139	GSAKYLNNQKKFKNQ<br>QKKFKNQSRVTLQYK<br>KLQATIAKKLFAIRS<br>LKSCMDILTKAKPKV<br>NDFLDSPQSRPKKLR<br>DFLDSPQSRPKKLRP<br>FLDSPQSRPKKLRPS<br>LDSPQSRPKKLRPSP<br>DSPQSRPKKLRPSPK<br>SPQSRPKKLRPSPKD<br>PQSRPKKLRPSPKDY<br>SRPKKLRPSPKDYWY<br>RPKKLRPSPKDYWYS<br>KAHLFRNLAKEMNNR<br>GNKRQCRGVRVTRRS<br>NKRQCRGVRVTRRSL<br>KRQCRGVRVTRRSLR<br>RQCRGVRVTRRSLRE<br>QCRGVRVTRRSLREF<br>RGVRVTRRSLREFSH<br>GVRVTRRSLREFSHF<br>VRVTRRSLREFSHFQ	CPP20-38<br>CPP20-39<br>CPP20-40<br>CPP20-41<br>CPP20-42<br>CPP20-43<br>CPP20-44<br>CPP20-45	KYLNNQKKFKNQSR<br>VTLQYK<br>KKFKNQSRVTLQYKA<br>TTNFK<br>QIICDDHMSKLQATI<br>AKKLF<br>REINVLKSCMDILTK<br>AKPKV<br>NDFLDSPQSRPKKLR<br>PSPKD<br>EGNKRQCRGVRVTR<br>RSLREF<br>NKRQCRGVRVTRRS<br>LREFSH<br>KRQCRGVRVTRRSL<br>REFSHF

		CPP10-262<br>CPP10-263<br>CPP10-264<br>CPP10-265<br>CPP10-266<br>CPP10-267<br>CPP10-268<br>CPP10-269<br>CPP10-270<br>CPP10-271<br>CPP10-272<br>CPP10-273<br>CPP10-274	GNKRQCRGVR<br>NKRQCRGVRV<br>KRQCRGVRVT<br>RQCRGVRVTR<br>QCRGVRVTRR<br>CRGVRVTRRS<br>RGVRVTRRSL<br>GVRVTRRSLR<br>VRVTRRSLRE<br>RVTRRSLREF<br>TRRSLREFSH<br>RRSLREFSHF<br>KSWVFEYTKK				
Dendrochirus zebra	Tx alpha-subunit (Fragment) A0A068BGX9	CPP10-275<br>CPP10-276<br>CPP10-277<br>CPP10-278<br>CPP10-279<br>CPP10-280<br>CPP10-281<br>CPP10-282<br>CPP10-283<br>CPP10-284<br>CPP10-285<br>CPP10-286<br>CPP10-287<br>CPP10-288<br>CPP10-289<br>CPP10-290	GMLYDARKEK<br>KMEAAIRKIP<br>PMKVWLVPLK<br>MKVWLVPLKK<br>KVWLVPLKKF<br>RDISAGRVKK<br>DISAGRVKKI<br>ELFVLRRRCN<br>QTIATKLPLI<br>TIATKLPLIR<br>KIAKDRAKSP<br>GVILKMKQKA<br>DTVKNRRYVR<br>VKNRRYVRFL<br>KNRRYVRFLA<br>NRRYVRFLAA	CPP15-140<br>CPP15-141	LFVLRRRCNEALDDK<br>KIAKDRAKSPFSNEK	CPP20-46<br>CPP20-47	MPALGRPFTLGMLY<br>DARKEK<br>GRVKKIHSIVEELFVL<br>RRRC
	Tx beta-subunit (Fragment) A0A068BD83	CPP10-291<br>CPP10-292<br>CPP10-293<br>CPP10-294<br>CPP10-295<br>CPP10-296<br>CPP10-297<br>CPP10-298<br>CPP10-299<br>CPP10-300<br>CPP10-301<br>CPP10-302<br>CPP10-303	KYLNDSKKFK<br>KKFKNQSRVT<br>RKVLNNFKKI<br>KKICNDYKLN<br>KLNLQRTIAK<br>NLQRTIAKKL<br>LQRTIAKKLL<br>RTIAKKLLSI<br>TIAKKLLSIR<br>KMTVNIKKLQ<br>VNIKKLQPPP<br>NIKKLQPPPN<br>KAHHFRNLAK	CPP15-142<br>CPP15-143<br>CPP15-144<br>CPP15-145	GSAKYLNDSKKFKNQ<br>SKKFKNQSRVTLQYK<br>KLNLQRTIAKKLLSI<br>KAHHFRNLAKDMNNR	CPP20-48<br>CPP20-49	KKICNDYKLNLQRTI<br>AKKLL<br>KDFSESPKMTVNIKK<br>LQPPP

		CPP10-304<br>CPP10-305<br>CPP10-306	DRRALLWYDC<br>KKAVSGKPKP<br>KAVSGKPKPF				
	Tx gamma-subunit (Fragment)<br>A0A068BFX2	CPP10-307<br>CPP10-308<br>CPP10-309<br>CPP10-310<br>CPP10-311<br>CPP10-312<br>CPP10-313<br>CPP10-314<br>CPP10-315<br>CPP10-316<br>CPP10-317<br>CPP10-318<br>CPP10-319<br>CPP10-320<br>CPP10-321<br>CPP10-322<br>CPP10-323<br>CPP10-324<br>CPP10-325<br>CPP10-326<br>CPP10-327<br>CPP10-328<br>CPP10-329<br>CPP10-330<br>CPP10-331<br>CPP10-332	DARKDRLIPG<br>RKDRLIPGFT<br>KYLDDRKKFK<br>DRKKFKNQSR<br>RKKFKNQSRV<br>KKFKNQSRVT<br>SAITDRFSCK<br>SSLPILRRVR<br>SLPILRRVRN<br>LPILRRVRNT<br>PILRRVRNTL<br>LDSPTSKPKK<br>DSPTSKPKKL<br>SPTSKPKKLR<br>PTSKPKKLRP<br>TSKPKKLRPS<br>SKPKKLRPST<br>KPKKLRPSTK<br>KKLRPSTKDY<br>KLRPSTKDYW<br>MMREKAHHFR<br>FDNQQRRFER<br>DNQQRRFERY<br>NQQRRFERYQ<br>QQRRFERYQQ<br>GLSRRHYWEL	CPP15-146<br>CPP15-147<br>CPP15-148<br>CPP15-149<br>CPP15-150<br>CPP15-151<br>CPP15-152<br>CPP15-153<br>CPP15-154<br>CPP15-155<br>CPP15-156<br>CPP15-157<br>CPP15-158<br>CPP15-159<br>CPP15-160<br>CPP15-161	GSAKYLDDRKKFKNQ<br>KYLDDRKKFKNQSRV<br>RKKFKNQSRVTLQYK<br>KLMADSSLPILRRVR<br>SLPILRRVRNTLEAM<br>LPILRRVRNTLEAMR<br>SDFLDSPTSKPKKLR<br>DFLDSPTSKPKKLRP<br>FLDSPTSKPKKLRPS<br>LDSPTSKPKKLRPST<br>DSPTSKPKKLRPSTK<br>SPTSKPKKLRPSTKD<br>KPKKLRPSTKDYWYI<br>KAHHFRNLALDMNNR<br>AKPFDNQQRRFERYQ<br>KPFDNQQRRFERYQQ	CPP20-50<br>CPP20-51	KYLDDRKKFKNQSR<br>VTLQYK<br>RRFERYQQVLCNKG<br>LSRRHY
Hydrophis hardwickii	Short neurotoxin 1<br>P68416	CPP10-333<br>CPP10-334	KTWSDHRGTR<br>HRGTRIERGC	CPP15-162	KTWSDHRGTRIERGC		
	Cysteine-rich venom protein 1<br>Q8UW25	CPP10-335<br>CPP10-336<br>CPP10-337<br>CPP10-338<br>CPP10-339<br>CPP10-340<br>CPP10-341<br>CPP10-342	KHNALRRSVK<br>HNALRRSVKP<br>NALRRSVKPT<br>ALRRSVKPTA<br>LRRSVKPTAR<br>RAAQNAKRSA<br>AQNAKRSADR<br>KRSADRCTFA	CPP15-163<br>CPP15-164<br>CPP15-165<br>CPP15-166<br>CPP15-167<br>CPP15-168<br>CPP15-169<br>CPP15-170	REIVDKHNALRRSVK<br>IVDKHNALRRSVKPT<br>DKHNALRRSVKPTAR<br>KHNALRRSVKPTARN<br>HNALRRSVKPTARNM<br>NSRAAQNAKRSADRC<br>EDTFSNCKALAKKTK<br>TFSNCKALAKKTKCK	CPP20-52<br>CPP20-53<br>CPP20-54<br>CPP20-55<br>CPP20-56<br>CPP20-57<br>CPP20-58<br>CPP20-59	KKDYRREIVDKHNAL<br>RRSVK<br>KDYRREIVDKHNALR<br>RSVKP<br>KHNALRRSVKPTAR<br>NMLQMK<br>CEYEDTFSNCKALAK<br>KTKCK

		CPP10-343<br>CPP10-344<br>CPP10-345<br>CPP10-346<br>CPP10-347<br>CPP10-348<br>CPP10-349<br>CPP10-350<br>CPP10-351<br>CPP10-352	HTRTVGKFRC<br>FSNCKALAKK<br>SNCKALAKKT<br>NCKALAKKTK<br>CKALAKKTKC<br>KALAKKTKCK<br>ALAKKTKCKT<br>KKTKCKTEWI<br>KCKTEWIKSK<br>CKTEWIKSKC	CPP15-171<br>CPP15-172<br>CPP15-173<br>CPP15-174<br>CPP15-175	FSNCKALAKKTKCKT<br>CKALAKKTKCKTEWI<br>KALAKKTKCKTEWIK<br>KKTKCKTEWIKSKCP<br>KCKTEWIKSKCPATC		SNCKALAKKTKCKTE<br>WIKSK<br>CKALAKKTKCKTEWI<br>KSKCP<br>KALAKKTKCKTEWIK<br>SKCPA<br>ALAKKTKCKTEWIKS<br>KCPAT
	Cysteine-rich venom protein 2<br>Q8UW11	CPP10-353<br>CPP10-354<br>CPP10-355<br>CPP10-356<br>CPP10-357<br>CPP10-358<br>CPP10-359<br>CPP10-360<br>CPP10-361	KHNALRRSVK<br>HNALRRSVKP<br>NALRRSVKPT<br>ALRRSVKPTA<br>LRRSVKPTAR<br>HAAQNAKRSA<br>AQNAKRSADR<br>KRSADRCTFA<br>HTRTVGKFRC	CPP15-176<br>CPP15-177<br>CPP15-178<br>CPP15-179<br>CPP15-180	REIVDKHNALRRSVK<br>IVDKHNALRRSVKPT<br>DKHNALRRSVKPTAR<br>KHNALRRSVKPTARN<br>HNALRRSVKPTARNM	CPP20-60<br>CPP20-61	KKDYQREIVDKHNAL<br>RRSVK<br>KHNALRRSVKPTAR<br>NMLQMK
	Long neurotoxin 1<br>Q8UW29	CPP10-362<br>CPP10-363<br>CPP10-364<br>CPP10-365<br>CPP10-366	YTRTCFRTPY<br>TRTCFRTPYK<br>RTCFRTPYKP<br>GQNLCYKKSW<br>GCTAKCPTVK	CPP15-181<br>CPP15-182<br>CPP15-183	DLAYTRTCFRTPYKP<br>LAYTRTCFRTPYKPE<br>TRTCFRTPYKPETCP		
	Long neurotoxin 2<br>A3FM53	CPP10-367<br>CPP10-368<br>CPP10-369<br>CPP10-370<br>CPP10-371	YTRTCYRTHP<br>RTCYRTHPYK<br>GQNLCYKKSW<br>GCTAKCPTVK<br>KCPTVKHGKD	CPP15-184<br>CPP15-185<br>CPP15-186	DLAYTRTCYRTHPYK<br>LAYTRTCYRTHPYKP<br>YTRTCYRTHPYKPET	CPP20-62	HPYKPETCPPGQNL<br>CYKKSW
Hydrophis cyanocinctus	Short neurotoxin 2 (Fragment)<br>P62376	CPP10-372<br>CPP10-373	KTWSDHRGTR<br>HRGTRIERGC	CPP15-187	KTWSDHRGTRIERGC		
	Short neurotoxin 1<br>P25494	CPP10-374<br>CPP10-375<br>CPP10-376	KTWSDHRGTR<br>HRGTRIERGC<br>GCPQVKKGIK	CPP15-188	KTWSDHRGTRIERGC		
Hydrophis schistosus	Short neurotoxin 1<br>P68415	CPP10-377<br>CPP10-378	KTWSDHRGTR<br>HRGTRIERGC	CPP15-189	KTWSDHRGTRIERGC	CPP20-63	KKTWSDHRGTRIER<br>GCGCPQ

Hydrophis platurus	Pelamitoxin a<br>P62388	CPP10-379<br>CPP10-380	KTWSDHRGTR<br>HRGTRIERGC	CPP15-190	KTWSDHRGTRIERGC		
Conus textile	Conotoxin tx3c<br>P58846	CPP10-381	KRCCRTCFGC				
	Epsilon-conotoxin TxVA<br>P81755	CPP10-382<br>CPP10-383<br>CPP10-384<br>CPP10-385<br>CPP10-386<br>CPP10-387<br>CPP10-388<br>CPP10-389<br>CPP10-390	LRDNLKRTIR<br>RDNLKRTIRT<br>DNLKRTIRTR<br>NLKRTIRTRL<br>LKRTIRTRLN<br>KRTIRTRLNI<br>RTIRTRLNIR<br>TIRTRLNIRE<br>RTRLNIRECC	CPP15-191<br>CPP15-192<br>CPP15-193<br>CPP15-194<br>CPP15-195<br>CPP15-196<br>CPP15-197<br>CPP15-198<br>CPP15-199	PLSSLRDNLKRTIRT<br>SSLRDNLKRTIRTRL<br>SLRDNLKRTIRTRLN<br>LRDNLKRTIRTRLNI<br>RDNLKRTIRTRLNIR<br>DNLKRTIRTRLNIRE<br>NLKRTIRTRLNIREC<br>LKRTIRTRLNIRECC<br>RTIRTRLNIRECCED	CPP20-64<br>CPP20-65	PLSSLRDNLKRTIRTR<br>LNIR<br>SLRDNLKRTIRTRLNI<br>RECC
	Delta-conotoxin TxVIA<br>Q9U655	CPP10-391<br>CPP10-392<br>CPP10-393<br>CPP10-394<br>CPP10-395	KNPEASKLNK<br>EASKLNKRWC<br>ASKLNKRWCK<br>SKLNKRWCKQ<br>KLNKRWCKQS	CPP15-200	KNPEASKLNKRWCKQ	CPP20-66	SNAHHQMKNPEAS<br>KLNKRWC
	Alpha-conotoxin TxIA<br>P0DM21	CPP10-396	KGCCSRPPCI				
Conus textile	Conotoxin Tx3.5-a<br>P0C1N7	CPP10-397<br>CPP10-398<br>CPP10-399<br>CPP10-400<br>CPP10-401<br>CPP10-402<br>CPP10-403<br>CPP10-404	HPLFDQKRRC<br>PLFDQKRRCC<br>LFDQKRRCCK<br>FDQKRRCCKF<br>DQKRRCCKFP<br>QKRRCCKFPC<br>KRRCCKFPCP<br>RRCCKFPCPD	CPP15-201<br>CPP15-202<br>CPP15-203<br>CPP15-204<br>CPP15-205<br>CPP15-206	QHPLFDQKRRCCKFP<br>HPLFDQKRRCCKFPC<br>PLFDQKRRCCKFPCP<br>LFDQKRRCCKFPCPD<br>FDQKRRCCKFPCPDS<br>KRRCCKFPCPDSCRY	CPP20-67	RMQAEQHPLFDQK<br>RRCCKFP
	Conotoxin tx9a<br>Q9GU58	CPP10-405<br>CPP10-406<br>CPP10-407<br>CPP10-408<br>CPP10-409<br>CPP10-410	TDNRRNLQSK<br>DNRRNLQSKW<br>NRRNLQSKWK<br>RRNLQSKWKP<br>RNLQSKWKPV<br>KPVSLYMSRR	CPP15-207<br>CPP15-208<br>CPP15-209	GQLTDNRRNLQSKWK<br>QLTDNRRNLQSKWKP<br>DNRRNLQSKWKPVSL	CPP20-68	DNRRNLQSKWKPVS<br>LYMSRR
	Gamma-conotoxin-like TxVIIA<br>P24160	CPP10-411<br>CPP10-412<br>CPP10-413<br>CPP10-414	KLTILLLVAA<br>SETRKLARNK<br>ETRKLARNKQ<br>TRKLARNKQK	CPP15-210<br>CPP15-211<br>CPP15-212<br>CPP15-213	KSRKAEINFSETRKL<br>NFSETRKLARNKQKR<br>FSETRKLARNKQKRC<br>SETRKLARNKQKRCG	CPP20-69<br>CPP20-70<br>CPP20-71	KSRKAEINFSETRKLA<br>RNKQ<br>RKAEINFSETRKLAR<br>NKQKR

	CPP10-415<br>CPP10-416<br>CPP10-417<br>CPP10-418	RKLARNKQKR<br>KLARNKQKRC<br>LARNKQKRCG<br>RNKQKRCGGY	CPP15-214<br>CPP15-215<br>CPP15-216	ETRKLARNKQKRCGG<br>TRKLARNKQKRCGGY<br>KLARNKQKRCGGYST		KAEINFSETRKLARN<br>KQKRC
Conotoxin tx3h<br>P0C1N6	CPP10-419<br>CPP10-420<br>CPP10-421<br>CPP10-422	RREILLPALR<br>REILLPALRK<br>ILLPALRKFC<br>LLPALRKFCC	CPP15-217	ERREILLPALRKFCC	CPP20-72	NKQLLNPDERREILL<br>PALRK
TxMEKL-P2<br>Q9BPA9	CPP10-423<br>CPP10-424<br>CPP10-425	KLTILLLVAA<br>KINFLSRSDR<br>FLSRSDRDCR				
TxMMSK-03<br>Q9BPJ7	CPP10-426<br>CPP10-427<br>CPP10-428	LIICLLLFPL<br>DQPADRPAER<br>RRCCNAGFCR				
Alpha-conotoxin-like Tx1.2<br>P0DPL9	CPP10-429<br>CPP10-430<br>CPP10-431<br>CPP10-432<br>CPP10-433<br>CPP10-434<br>CPP10-435	TSGRSTFRGR<br>SGRSTFRGRN<br>GRSTFRGRNA<br>RSTFRGRNAA<br>VSLTDRRPQC<br>SLTDRRPQCC<br>RRPQCCSHPA	CPP15-218<br>CPP15-219<br>CPP15-220<br>CPP15-221<br>CPP15-222	SFTSGRSTFRGRNAA<br>GRSTFRGRNAAAKAS<br>RSTFRGRNAAAKASG<br>VSLTDRRPQCCSHPA<br>SLTDRRPQCCSHPAC		
Alpha-conotoxin TxIB (Fragment)<br>K4RNX9	CPP10-436	RGCCSDPPCR				
Conotoxin tx3b<br>P0C1N8	CPP10-437<br>CPP10-438	KLGALLTICL<br>DQPAQRLQDR				
Cysteine-rich venom protein<br>Q7YT83	CPP10-439<br>CPP10-440<br>CPP10-441<br>CPP10-442<br>CPP10-443<br>CPP10-444<br>CPP10-445<br>CPP10-446<br>CPP10-447<br>CPP10-448<br>CPP10-449	VLVAGRKRHH<br>LVAGRKRHHC<br>AGRKRHHCDS<br>GRKRHHCDSK<br>RKRHHCDSKY<br>SGRRGEPDLP<br>MCRYPDALRR<br>CRYPDALRRP<br>RYPDALRRPQ<br>YPDALRRPQH<br>RRPQHWCQYD	CPP15-223<br>CPP15-224<br>CPP15-225<br>CPP15-226	IVLVAGRKRHHCDSK<br>VLVAGRKRHHCDSKY<br>MCRYPDALRRPQHWC<br>YPDALRRPQHWCQYD		

Conotoxin Gla(1)-TxVI<br>P86259	CPP10-450<br>CPP10-451<br>CPP10-452<br>CPP10-453<br>CPP10-454<br>CPP10-455<br>CPP10-456<br>CPP10-457<br>CPP10-458<br>CPP10-459<br>CPP10-460	KLTILLLVAA<br>KENINFLLKR<br>ENINFLLKRK<br>NINFLLKRKR<br>INFLLKRKRA<br>NFLLKRKRAA<br>FLLKRKRAAD<br>LLKRKRAADR<br>LKRKRAADRG<br>KRKRAADRGM<br>RKRAADRGMW	CPP15-227<br>CPP15-228<br>CPP15-229<br>CPP15-230<br>CPP15-231<br>CPP15-232<br>CPP15-233<br>CPP15-234	NHSKENINFLLKRKR<br>HSKENINFLLKRKRA<br>SKENINFLLKRKRAA<br>KENINFLLKRKRAAD<br>ENINFLLKRKRAADR<br>NINFLLKRKRAADRG<br>INFLLKRKRAADRGM<br>FLLKRKRAADRGMWG	CPP20-73<br>CPP20-74<br>CPP20-75	ERAGENHSKENINFL<br>LKRKR<br>RAGENHSKENINFLL<br>KRKRA<br>FLLKRKRAADRGM<br>WGECKDG
Omega-conotoxin TxVII<br>P56714	CPP10-461<br>CPP10-462<br>CPP10-463<br>CPP10-464<br>CPP10-465	KNPEASKLNK<br>EASKLNKRCK<br>ASKLNKRCKQ<br>SKLNKRCKQA<br>KLNKRCKQAD	CPP15-235	KNPEASKLNKRCKQA	CPP20-76	PKAHHEMKNPEASK<br>LNKRCK
Conotoxin Gla(2)-TxVI/A<br>P58922	CPP10-466<br>CPP10-467<br>CPP10-468<br>CPP10-469<br>CPP10-470<br>CPP10-471<br>CPP10-472<br>CPP10-473	KLIILLLVAA<br>ALFQEKRPMK<br>LFQEKRPMKK<br>FQEKRPMKKI<br>KRPMKKIDFL<br>KKIDFLSKGK<br>KIDFLSKGKT<br>KTDAEKQQKR	CPP15-236<br>CPP15-237<br>CPP15-238<br>CPP15-239	MSTQALFQEKRPMKK<br>FQEKRPMKKIDFLSK<br>KRPMKKIDFLSKGKT<br>GKTDAEKQQKRSCSD	CPP20-77	ALFQEKRPMKKIDFL<br>SKGKT
Conotoxin 3<br>Q3YEH7	CPP10-474<br>CPP10-475<br>CPP10-476<br>CPP10-477<br>CPP10-478<br>CPP10-479<br>CPP10-480<br>CPP10-481	FNGNARRTPR<br>NGNARRTPRM<br>GNARRTPRML<br>NARRTPRMLS<br>ARRTPRMLSN<br>RRTPRMLSNK<br>RTPRMLSNKR<br>RMLSNKRICC	CPP15-240<br>CPP15-241<br>CPP15-242<br>CPP15-243<br>CPP15-244<br>CPP15-245	PLPSFNGNARRTPRM<br>FNGNARRTPRMLSNK<br>NGNARRTPRMLSNKR<br>GNARRTPRMLSNKRI<br>NARRTPRMLSNKRIC<br>ARRTPRMLSNKRICC	CPP20-78<br>CPP20-79<br>CPP20-80<br>CPP20-81	KDDMPLPSFNGNAR<br>RTPRML<br>PLPSFNGNARRTPR<br>MLSNKR<br>GNARRTPRMLSNKR<br>ICCYPN<br>ARRTPRMLSNKRICC<br>YPNVW
Conotoxin Gla(2)-TxVI/B<br>Q9BPB1	CPP10-482<br>CPP10-483<br>CPP10-484<br>CPP10-485<br>CPP10-486<br>CPP10-487<br>CPP10-488<br>CPP10-489<br>CPP10-490<br>CPP10-491	KLIILLLVAA<br>ALFQEKRTMK<br>LFQEKRTMKK<br>FQEKRTMKKI<br>KKIDFLSKGK<br>KIDFLSKGKA<br>KGKADAEKQR<br>GKADAEKQRK<br>KADAEKQRKR<br>DAEKQRKRNC	CPP15-246<br>CPP15-247<br>CPP15-248<br>CPP15-249	MSTQALFQEKRTMKK<br>KRTMKKIDFLSKGKA<br>GKADAEKQRKRNCSD<br>KADAEKQRKRNCSDD	CPP20-82<br>CPP20-83<br>CPP20-84<br>CPP20-85	ALFQEKRTMKKIDFL<br>SKGKA<br>MKKIDFLSKGKADAE<br>KQRKR<br>KKIDFLSKGKADAEK<br>QRKRN<br>KIDFLSKGKADAEKQ<br>RKRNC

	CPP10-492	KQRKRNCSDD				
Omega-conotoxin-like TxO4<br>Q9XZL1	CPP10-493	GMIKIGPPCC				
Conotoxin TeAr151<br>Q3YEE9	CPP10-494<br>CPP10-495<br>CPP10-496<br>CPP10-497<br>CPP10-498<br>CPP10-499<br>CPP10-500<br>CPP10-501<br>CPP10-502	IQNTLQTLRK<br>QNTLQTLRKK<br>NTLQTLRKKV<br>TLQTLRKKVC<br>LQTLRKKVCC<br>QTLRKKVCCR<br>TLRKKVCCRP<br>RKKVCCRPMQ<br>KKVCCRPMQD	CPP15-250<br>CPP15-251<br>CPP15-252	DNIQNTLQTLRKKVC<br>IQNTLQTLRKKVCCR<br>TLQTLRKKVCCRPMQ		
Conotoxin Gla(3)-TxVI<br>Q9BPB4	CPP10-503<br>CPP10-504<br>CPP10-505<br>CPP10-506<br>CPP10-507<br>CPP10-508<br>CPP10-509<br>CPP10-510<br>CPP10-511<br>CPP10-512<br>CPP10-513<br>CPP10-514<br>CPP10-515	KLIILLLVAA<br>QAVLQEKRPK<br>VLQEKRPKEK<br>EKRPKEKIKF<br>KRPKEKIKFL<br>PKEKIKFLSK<br>KEKIKFLSKR<br>EKIKFLSKRK<br>KIKFLSKRKT<br>KFLSKRKTDA<br>RKTDAEKQQK<br>KTDAEKQQKR<br>KQQKRLCPDY	CPP15-253<br>CPP15-254<br>CPP15-255<br>CPP15-256<br>CPP15-257<br>CPP15-258<br>CPP15-259<br>CPP15-260<br>CPP15-261<br>CPP15-262<br>CPP15-263	QAVLQEKRPKEKIKF<br>LQEKRPKEKIKFLSK<br>QEKRPKEKIKFLSKR<br>EKRPKEKIKFLSKRK<br>KRPKEKIKFLSKRKT<br>PKEKIKFLSKRKTDA<br>EKIKFLSKRKTDAEK<br>KFLSKRKTDAEKQQK<br>FLSKRKTDAEKQQKR<br>RKTDAEKQQKRLCPD<br>KTDAEKQQKRLCPDY	CPP20-86<br>CPP20-87<br>CPP20-88<br>CPP20-89<br>CPP20-90<br>CPP20-91<br>CPP20-92<br>CPP20-93<br>CPP20-94<br>CPP20-95<br>CPP20-96<br>CPP20-97	SAQAVLQEKRPKEKIKFLSK<br>QAVLQEKRPKEKIKFLSKRK<br>AVLQEKRPKEKIKFLSKRKT<br>VLQEKRPKEKIKFLSKRKTD<br>LQEKRPKEKIKFLSKRKTDA<br>KRPKEKIKFLSKRKTDAEKQ<br>PKEKIKFLSKRKTDAEKQQK<br>KEKIKFLSKRKTDAEKQQKR<br>EKIKFLSKRKTDAEKQQKRL<br>KIKFLSKRKTDAEKQQKRLC<br>KFLSKRKTDAEKQQKRLCPD<br>FLSKRKTDAEKQQKRLCPDY
Conotoxin TeAr154	CPP10-516<br>CPP10-517	GLSVDARPKT<br>TKSTLQRLLK	CPP15-264<br>CPP15-265	SFRDNTKSTLQRLLK<br>FRDNTKSTLQRLLKR	CPP20-98	SSFRDNTKSTLQRLLKRVNC

Q3YEE8	CPP10-518<br>CPP10-519<br>CPP10-520<br>CPP10-521	KSTLQRLLKR<br>STLQRLLKRV<br>TLQRLLKRVN<br>LQRLLKRVNC	CPP15-266<br>CPP15-267<br>CPP15-268<br>CPP15-269<br>CPP15-270	RDNTKSTLQRLLKRV<br>DNTKSTLQRLLKRVN<br>NTKSTLQRLLKRVNC<br>TKSTLQRLLKRVNCC<br>KSTLQRLLKRVNCCP		
Omega-conotoxin P28880	CPP10-522<br>CPP10-523<br>CPP10-524<br>CPP10-525<br>CPP10-526<br>CPP10-527<br>CPP10-528<br>CPP10-529<br>CPP10-530<br>CPP10-531<br>CPP10-532<br>CPP10-533	RGTQKHRTLR<br>GTQKHRTLRS<br>TQKHRTLRST<br>KHRTLRSTAR<br>HRTLRSTARR<br>RTLRSTARRS<br>TLRSTARRSK<br>LRSTARRSKS<br>RSTARRSKSE<br>RRSKSESTTR<br>CCGRCYRGKC<br>CGRCYRGKCT	CPP15-271<br>CPP15-272<br>CPP15-273<br>CPP15-274<br>CPP15-275<br>CPP15-276<br>CPP15-277<br>CPP15-278<br>CPP15-279<br>CPP15-280<br>CPP15-281<br>CPP15-282	TAEDSRGTQKHRTLR<br>SRGTQKHRTLRSTAR<br>RGTQKHRTLRSTARR<br>GTQKHRTLRSTARRS<br>TQKHRTLRSTARRSK<br>QKHRTLRSTARRSKS<br>KHRTLRSTARRSKSE<br>HRTLRSTARRSKSES<br>TLRSTARRSKSESTT<br>LRSTARRSKSESTTR<br>RSTARRSKSESTTRC<br>STARRSKSESTTRCR	CPP20-99<br>CPP20-100<br>CPP20-101<br>CPP20-102<br>CPP20-103<br>CPP20-104<br>CPP20-105<br>CPP20-106<br>CPP20-107	DSRGTQKHRTLRSTA<br>RRSKS<br>SRGTQKHRTLRSTAR<br>RSKSE<br>RGTQKHRTLRSTARR<br>SKSES<br>GTQKHRTLRSTARRS<br>KSEST<br>QKHRTLRSTARRSKS<br>ESTTR<br>KHRTLRSTARRSKSE<br>STTRC<br>HRTLRSTARRSKSES<br>TTRCR<br>TLRSTARRSKSESTTR<br>CRSS<br>RSTARRSKSESTTRC<br>RSSGS
Omega-conotoxin Q9XZK2	CPP10-534<br>CPP10-535<br>CPP10-536<br>CPP10-537<br>CPP10-538<br>CPP10-539<br>CPP10-540<br>CPP10-541<br>CPP10-542<br>CPP10-543<br>CPP10-544<br>CPP10-545<br>CPP10-546	RGTQKHRTLR<br>GTQKHRTLRS<br>TQKHRTLRSK<br>QKHRTLRSKT<br>KHRTLRSKTK<br>HRTLRSKTKL<br>RTLRSKTKLS<br>TLRSKTKLSM<br>RSKTKLSMST<br>KLSMSTRCKA<br>STRCKAAGKP<br>TRCKAAGKPC<br>CKAAGKPCSR	CPP15-283<br>CPP15-284<br>CPP15-285<br>CPP15-286<br>CPP15-287<br>CPP15-288<br>CPP15-289<br>CPP15-290<br>CPP15-291<br>CPP15-292<br>CPP15-293<br>CPP15-294<br>CPP15-295	TADDSRGTQKHRTLR<br>DDSRGTQKHRTLRSK<br>DSRGTQKHRTLRSKT<br>SRGTQKHRTLRSKTK<br>RGTQKHRTLRSKTKL<br>GTQKHRTLRSKTKLS<br>TQKHRTLRSKTKLSM<br>QKHRTLRSKTKLSMS<br>KHRTLRSKTKLSMST<br>HRTLRSKTKLSMSTR<br>TLRSKTKLSMSTRCK<br>KLSMSTRCKAAGKPC<br>CKAAGKPCSRIAYNC	CPP20-108<br>CPP20-109<br>CPP20-110<br>CPP20-111<br>CPP20-112<br>CPP20-113<br>CPP20-114<br>CPP20-115<br>CPP20-116<br>CPP20-117<br>CPP20-118<br>CPP20-119	QLITADDSRGTQKH<br>RTLRSK<br>ITADDSRGTQKHRTL<br>RSKTK<br>SRGTQKHRTLRSKTK<br>LSMST<br>RGTQKHRTLRSKTKL<br>SMSTR<br>GTQKHRTLRSKTKLS<br>MSTRC<br>TQKHRTLRSKTKLSM<br>STRCK<br>QKHRTLRSKTKLSMS<br>TRCKA<br>KHRTLRSKTKLSMST<br>RCKAA<br>HRTLRSKTKLSMSTR<br>CKAAG<br>TLRSKTKLSMSTRCK

						AAGKP<br>RSKTKLSMSTRCKAA<br>GKPCS<br>SKTKLSMSTRCKAAG<br>KPCSR
Delta-conotoxin<br>Q9XZK5	CPP10-547<br>CPP10-548<br>CPP10-549<br>CPP10-550	YGLKNLFPKA<br>GLKNLFPKAR<br>PKARHEMKNP<br>KNPEASKLNK	CPP15-296	LKNLFPKARHEMKNP	CPP20-120<br>CPP20-121	DSRYGLKNLFPKARH<br>EMKNP<br>LFPKARHEMKNPEA<br>SKLNKR
Omega-conotoxin<br>P28881	CPP10-551<br>CPP10-552<br>CPP10-553<br>CPP10-554<br>CPP10-555<br>CPP10-556<br>CPP10-557<br>CPP10-558<br>CPP10-559<br>CPP10-560<br>CPP10-561<br>CPP10-562	RGTQKHRALR<br>GTQKHRALRS<br>TQKHRALRSD<br>KHRALRSDTK<br>RALRSDTKLP<br>RSDTKLPMST<br>KLPMSTRCKL<br>LPMSTRCKLK<br>PMSTRCKLKG<br>MSTRCKLKGQ<br>CKLKGQSCRK<br>KGQSCRKTSY	CPP15-297<br>CPP15-298<br>CPP15-299<br>CPP15-300<br>CPP15-301<br>CPP15-302<br>CPP15-303<br>CPP15-304	SRGTQKHRALRSDTK<br>RGTQKHRALRSDTKL<br>GTQKHRALRSDTKLP<br>KHRALRSDTKLPMST<br>RSDTKLPMSTRCKLK<br>SDTKLPMSTRCKLKG<br>KLPMSTRCKLKGQSC<br>MSTRCKLKGQSCRKT	CPP20-122<br>CPP20-123<br>CPP20-124<br>CPP20-125<br>CPP20-126	QKHRALRSDTKLPM<br>STRCKL<br>KHRALRSDTKLPMST<br>RCKLK<br>HRALRSDTKLPMSTR<br>CKLKG<br>RALRSDTKLPMSTRC<br>KLKGQ<br>KLPMSTRCKLKGQS<br>CRKTSY
Conotoxin Gla-TxX<br>Q5I4E6	CPP10-563<br>CPP10-564<br>CPP10-565<br>CPP10-566<br>CPP10-567<br>CPP10-568<br>CPP10-569<br>CPP10-570	CCHWARRDQC<br>SAQKGNGRRR<br>AQKGNGRRRL<br>QKGNGRRRLI<br>KGNGRRRLIH<br>GNGRRRLIHM<br>NGRRRLIHMQ<br>GRRRLIHMQK	CPP15-305<br>CPP15-306<br>CPP15-307	RCISAQKGNGRRRLI<br>SAQKGNGRRRLIHMQ<br>AQKGNGRRRLIHMQK	CPP20-127<br>CPP20-128	KPQRCISAQKGNGR<br>RRLIHM<br>QRCISAQKGNGRRR<br>LIHMQK
Contryphan-Tx<br>Q9NDA7	CPP10-571<br>CPP10-572<br>CPP10-573	FLNALQRRGC<br>ALQRRGCPWQ<br>LQRRGCPWQP				
Conotoxin King-Kong 2<br>P18513	CPP10-574	KRCAPFLHPC	CPP15-308	ASNLNKRCAPFLHPC		
Conotoxin King-Kong 1<br>P18512	CPP10-575	KNPEASKLNK	CPP15-309	KNPEASKLNKRCIEQ		

Leu-contryphan-Tx<br>Q9NDA6	CPP10-576<br>CPP10-577<br>CPP10-578	DQPADRKAVP<br>KLMDVLRPKK<br>LMDVLRPKKC	CPP15-310	KLMDVLRPKKCVLYP		
Conorfamide-Tx2<br>P0DM27	CPP10-579<br>CPP10-580<br>CPP10-581<br>CPP10-582<br>CPP10-583<br>CPP10-584<br>CPP10-585<br>CPP10-586	WSGPRNRFVR<br>SGPRNRFVRI<br>PRNRFVRIGR<br>RNRFVRIGRR<br>NRFVRIGRRD<br>RFVRIGRRDM<br>RIGRRDMQSP<br>PLLSERLRFR	CPP15-311<br>CPP15-312<br>CPP15-313<br>CPP15-314<br>CPP15-315<br>CPP15-316	LAWSGPRNRFVRIGR<br>AWSGPRNRFVRIGRR<br>WSGPRNRFVRIGRRD<br>SGPRNRFVRIGRRDM<br>GPRNRFVRIGRRDMQ<br>RNRFVRIGRRDMQSP	CPP20-129<br>CPP20-130<br>CPP20-131<br>CPP20-132<br>CPP20-133<br>CPP20-134<br>CPP20-135<br>CPP20-136	KKHSGILLAWSGPR<br>NRFVRI<br>HSGILLAWSGPRNRF<br>VRIGR<br>SGILLAWSGPRNRFV<br>RIGRR<br>GILLAWSGPRNRFV<br>RIGRRD<br>LLAWSGPRNRFVRI<br>GRRDMQ<br>WSGPRNRFVRIGRR<br>DMQSPL<br>RNRFVRIGRRDMQS<br>PLLSER<br>VRIGRRDMQSPLLSE<br>RLRFR
Alpha-conotoxin-like Tx1<br>Q9XZK6	CPP10-587<br>CPP10-588<br>CPP10-589<br>CPP10-590<br>CPP10-591<br>CPP10-592	TSGRRAFHGR<br>SGRRAFHGRN<br>GRRAFHGRNA<br>RRAFHGRNAA<br>VSLTDRRPEC<br>RRPECCSDPR	CPP15-317<br>CPP15-318<br>CPP15-319<br>CPP15-320	SSTSGRRAFHGRNAA<br>GRRAFHGRNAAAKAS<br>VSLTDRRPECCSDPR<br>SLTDRRPECCSDPRC		
Omega-conotoxin-like TxO6<br>Q9XZL3	CPP10-593	GLANLFSKSR				
Con-Ins Tx1<br>A0A0B5A7N8	CPP10-594<br>CPP10-595<br>CPP10-596<br>CPP10-597<br>CPP10-598<br>CPP10-599<br>CPP10-600<br>CPP10-601<br>CPP10-602<br>CPP10-603<br>CPP10-604<br>CPP10-605	TGRTSSLMKR<br>GRTSSLMKRR<br>RTSSLMKRRG<br>SSLMKRRGFL<br>MKRRGFLSLL<br>KRRGFLSLLK<br>RRGFLSLLKK<br>RGFLSLLKKR<br>GFLSLLKKRG<br>FLSLLKKRGK<br>LSLLKKRGKR<br>SLLKKRGKRD	CPP15-321<br>CPP15-322<br>CPP15-323<br>CPP15-324<br>CPP15-325<br>CPP15-326<br>CPP15-327<br>CPP15-328<br>CPP15-329<br>CPP15-330<br>CPP15-331<br>CPP15-332	NARANTGRTSSLMKR<br>ARANTGRTSSLMKRR<br>RANTGRTSSLMKRRG<br>GRTSSLMKRRGFLSL<br>SSLMKRRGFLSLLKK<br>SLMKRRGFLSLLKKR<br>LMKRRGFLSLLKKRG<br>MKRRGFLSLLKKRGK<br>KRRGFLSLLKKRGKR<br>RRGFLSLLKKRGKRD<br>RGFLSLLKKRGKRDE<br>SLLKKRGKRDEGSPL	CPP20-137<br>CPP20-138<br>CPP20-139<br>CPP20-140<br>CPP20-141<br>CPP20-142<br>CPP20-143<br>CPP20-144<br>CPP20-145<br>CPP20-146<br>CPP20-147<br>CPP20-148	GANNARANTGRTSS<br>LMKRRG<br>NTGRTSSLMKRRGF<br>LSLLKK<br>TGRTSSLMKRRGFLS<br>LLKKR<br>GRTSSLMKRRGFLSL<br>LKKRG<br>RTSSLMKRRGFLSLL<br>KKRGK<br>TSSLMKRRGFLSLLK<br>KRGKR

	CPP10-606<br>CPP10-607	LLKKRGKRDE<br>KRGKRDEGSP			CPP20-149	SSLMKRRGFLSLLKK<br>RGKRD<br>SLMKRRGFLSLLKKR<br>GKRDE<br>LMKRRGFLSLLKKRG<br>KRDEG<br>KRRGFLSLLKKRGKR<br>DEGSP<br>RRGFLSLLKKRGKRD<br>EGSPL<br>SLLKKRGKRDEGSPL<br>QRSGR<br>KRGKRDEGSPLQRS<br>GRGIVC
Omega-conotoxin-like TxO5<br>Q9XZL2	CPP10-608<br>CPP10-609	KNPEASKLNK<br>KLNKRCVPYE				
Alpha-conotoxin-like Tx2<br>Q9XZK7	CPP10-610<br>CPP10-611<br>CPP10-612<br>CPP10-613<br>CPP10-614	TSGRRTFHGR<br>SGRRTFHGRN<br>GRRTFHGRNA<br>RRTFHGRNAA<br>VSLTDRRPEC	CPP15-333<br>CPP15-334	SFTSGRRTFHGRNAA<br>GRRTFHGRNAAAKAS		
Conorfamide-Tx1<br>P0DM26	CPP10-615<br>CPP10-616<br>CPP10-617	DSIFRYGRRD<br>SIFRYGRRDM<br>RYGRRDMQSP				
Omega-conotoxin-like TxO5<br>Q9U654	CPP10-618<br>CPP10-619	KNPEASKLNK<br>KLNKRCVPYE	CPP15-335	KNPEASKLNKRCVPY		
Gamma-conotoxin-like TxMEKL-0511<br>Q9BHA0	CPP10-620<br>CPP10-621<br>CPP10-622<br>CPP10-623<br>CPP10-624<br>CPP10-625<br>CPP10-626<br>CPP10-627<br>CPP10-628<br>CPP10-629<br>CPP10-630<br>CPP10-631	KLTILLLVAA<br>EKHQRAKINL<br>QRAKINLLSK<br>RAKINLLSKR<br>AKINLLSKRK<br>KINLLSKRKP<br>INLLSKRKPP<br>NLLSKRKPPA<br>LLSKRKPPAE<br>SKRKPPAERW<br>KRKPPAERWW<br>RKPPAERWWR	CPP15-336<br>CPP15-337<br>CPP15-338<br>CPP15-339<br>CPP15-340<br>CPP15-341<br>CPP15-342<br>CPP15-343<br>CPP15-344<br>CPP15-345<br>CPP15-346<br>CPP15-347	EKHQRAKINLLSKRK<br>KHQRAKINLLSKRKP<br>HQRAKINLLSKRKPP<br>QRAKINLLSKRKPPA<br>RAKINLLSKRKPPAE<br>AKINLLSKRKPPAER<br>KINLLSKRKPPAERW<br>NLLSKRKPPAERWWR<br>LLSKRKPPAERWWRW<br>SKRKPPAERWWRWGG<br>KRKPPAERWWRWGGC<br>RKPPAERWWRWGGCM	CPP20-150<br>CPP20-151<br>CPP20-152<br>CPP20-153<br>CPP20-154<br>CPP20-155<br>CPP20-156<br>CPP20-157<br>CPP20-158	QALNQEKHQRAKIN<br>LLSKRK<br>ALNQEKHQRAKINLL<br>SKRKP<br>LNQEKHQRAKINLLS<br>KRKPP<br>NQEKHQRAKINLLSK<br>RKPPA<br>HQRAKINLLSKRKPP<br>AERWW<br>QRAKINLLSKRKPPA<br>ERWWR

	CPP10-632<br>CPP10-633<br>CPP10-634	KPPAERWWRW<br>AERWWRWGGC<br>RWWRWGGCMA	CPP15-348	KPPAERWWRWGGCMA		RAKINLLSKRKPPAERWWRW<br>KINLLSKRKPPAERWWRWGG<br>LLSKRKPPAERWWRWGGCMA
Contryphan-R/Tx<br>Q9NDA5	CPP10-635	MNVQRRSGCP				
Conotoxin Gla-TxXI<br>Q5I4E5	CPP10-636<br>CPP10-637	KSCCRWTCNQ<br>NQPCLIPGKR	CPP15-349	CRWTCNQPCLIPGKR	CPP20-159	CHKSCCRWTCNQPCLIPGKR
Gamma-conotoxin-like TeA53<br>Q3YEG0	CPP10-638<br>CPP10-639<br>CPP10-640<br>CPP10-641<br>CPP10-642<br>CPP10-643<br>CPP10-644<br>CPP10-645<br>CPP10-646<br>CPP10-647<br>CPP10-648<br>CPP10-649<br>CPP10-650<br>CPP10-651<br>CPP10-652<br>CPP10-653	KLTILLLVAA<br>EQHQRAKINL<br>QRAKINLLSK<br>RAKINLLSKR<br>AKINLLSKRK<br>KINLLSKRKP<br>INLLSKRKPP<br>NLLSKRKPPA<br>LLSKRKPPAE<br>SKRKPPAERW<br>KRKPPAERWW<br>RKPPAERWWR<br>KPPAERWWRW<br>AERWWRWGGC<br>RWWRWGGCML<br>MLWFGRCTKD	CPP15-350<br>CPP15-351<br>CPP15-352<br>CPP15-353<br>CPP15-354<br>CPP15-355<br>CPP15-356<br>CPP15-357<br>CPP15-358<br>CPP15-359<br>CPP15-360<br>CPP15-361<br>CPP15-362<br>CPP15-363	EQHQRAKINLLSKRK<br>QHQRAKINLLSKRKP<br>HQRAKINLLSKRKPP<br>QRAKINLLSKRKPPA<br>RAKINLLSKRKPPAE<br>AKINLLSKRKPPAER<br>KINLLSKRKPPAERW<br>NLLSKRKPPAERWWR<br>LLSKRKPPAERWWRW<br>SKRKPPAERWWRWGG<br>KRKPPAERWWRWGGC<br>RKPPAERWWRWGGCM<br>KPPAERWWRWGGCML<br>RWWRWGGCMLWFGRC	CPP20-160<br>CPP20-161<br>CPP20-162<br>CPP20-163<br>CPP20-164<br>CPP20-165<br>CPP20-166<br>CPP20-167<br>CPP20-168	ALNQEQHQRAKINLLSKRKP<br>NQEQHQRAKINLLSKRKPPA<br>HQRAKINLLSKRKPPAERWW<br>QRAKINLLSKRKPPAERWWR<br>RAKINLLSKRKPPAERWWRW<br>KINLLSKRKPPAERWWRWGG<br>LLSKRKPPAERWWRWGGCML<br>KPPAERWWRWGGCMLWFGRC<br>AERWWRWGGCMLWFGRCTKD
Omega-conotoxin-like TxMKLT1-0223<br>Q9U645	CPP10-654	GMIKIGPPCC				
Delta-conotoxin-like TxMKLT1-0111<br>Q9U656	CPP10-655<br>CPP10-656<br>CPP10-657<br>CPP10-658<br>CPP10-659	KNPEASKLNK<br>EASKLNKRWC<br>ASKLNKRWCK<br>SKLNKRWCKQ<br>KLNKRWCKQS	CPP15-364	KNPEASKLNKRWCKQ	CPP20-169	SNAHHQMKNPEASKLNKRWC

Gamma-conotoxin-like TxMEKL-053 Q9BPB0	CPP10-660<br>CPP10-661<br>CPP10-662<br>CPP10-663<br>CPP10-664<br>CPP10-665<br>CPP10-666<br>CPP10-667<br>CPP10-668<br>CPP10-669<br>CPP10-670<br>CPP10-671<br>CPP10-672<br>CPP10-673	KLTILLLVAA<br>EQHQRAKINL<br>QRAKINLLSK<br>RAKINLLSKR<br>AKINLLSKRK<br>KINLLSKRKP<br>INLLSKRKPP<br>NLLSKRKPPA<br>LLSKRKPPAE<br>SKRKPPAERW<br>KRKPPAERWW<br>RKPPAERWWE<br>CGIWFSRCTK<br>GIWFSRCTKD	CPP15-365<br>CPP15-366<br>CPP15-367<br>CPP15-368<br>CPP15-369<br>CPP15-370<br>CPP15-371<br>CPP15-372<br>CPP15-373	EQHQRAKINLLSKRK<br>QHQRAKINLLSKRKP<br>HQRAKINLLSKRKPP<br>QRAKINLLSKRKPPA<br>RAKINLLSKRKPPAE<br>AKINLLSKRKPPAER<br>KINLLSKRKPPAERW<br>NLLSKRKPPAERWWE<br>LLSKRKPPAERWWEC	CPP20-170<br>CPP20-171<br>CPP20-172<br>CPP20-173<br>CPP20-174	ALNQEQHQRAKINL<br>LSKRKP<br>NQEQHQRAKINLLS<br>KRKPPA<br>HQRAKINLLSKRKPP<br>AERWW<br>QRAKINLLSKRKPPA<br>ERWWE<br>KPPAERWWECGIW<br>FSRCTKD
Omega-conotoxin-like TxMKLT1-031 Q9U644	CPP10-674	GLANLFSKSR				
Conotoxin TxMEKL-021 Q9BHB7	CPP10-675<br>CPP10-676<br>CPP10-677<br>CPP10-678<br>CPP10-679<br>CPP10-680<br>CPP10-681<br>CPP10-682<br>CPP10-683<br>CPP10-684	PQGGGEKRPR<br>KRPRENIRFL<br>PRENIRFLSK<br>RENIRFLSKR<br>ENIRFLSKRK<br>NIRFLSKRKS<br>IRFLSKRKSN<br>RFLSKRKSNA<br>KRKSNAERWR<br>RKSNAERWRE	CPP15-374<br>CPP15-375<br>CPP15-376<br>CPP15-377<br>CPP15-378<br>CPP15-379<br>CPP15-380<br>CPP15-381	GGEKRPRENIRFLSK<br>GEKRPRENIRFLSKR<br>EKRPRENIRFLSKRK<br>KRPRENIRFLSKRKS<br>RPRENIRFLSKRKSN<br>PRENIRFLSKRKSNA<br>IRFLSKRKSNAERWR<br>RFLSKRKSNAERWRE	CPP20-175<br>CPP20-176<br>CPP20-177<br>CPP20-178<br>CPP20-179<br>CPP20-180<br>CPP20-181	PQGGGEKRPRENIRF<br>LSKRK<br>QGGGEKRPRENIRFL<br>SKRKS<br>GGGEKRPRENIRFLS<br>KRKSN<br>GGEKRPRENIRFLSK<br>RKSNA<br>RPRENIRFLSKRKSN<br>AERWR<br>PRENIRFLSKRKSNAE<br>RWRE<br>NIRFLSKRKSNAERW<br>REGSC
Conotoxin TxMMSK-02 Q9BPJ6	CPP10-685<br>CPP10-686	KLGALLTICL<br>DQPAQRLQDR				
Conotoxin TxMMSK-01 Q9BPJ1	CPP10-687<br>CPP10-688	PVKRCCRLLC<br>VKRCCRLLCL				

Conotoxin<br>TxMMSK-06<br>Q9BPJ4	CPP10-689<br>CPP10-690<br>CPP10-691<br>CPP10-692<br>CPP10-693<br>CPP10-694	HHPMLNSIRR<br>MLNSIRRREQ<br>LNSIRRREQN<br>NSIRRREQNQ<br>SIRRREQNQF<br>KLRDSRGERC	CPP15-382<br>CPP15-383	HPMLNSIRRREQNQF<br>KLRDSRGERCCGPTA		
Conotoxin<br>TxMMSK-04<br>Q9BPJ5	CPP10-695<br>CPP10-696	DQPADRPAER<br>HPFFDSVKKK				
Conotoxin<br>TeAr193<br>Q3YEH2	CPP10-697<br>CPP10-698<br>CPP10-699<br>CPP10-700	LESKRNCCRR<br>SKRNCCRRQI<br>KRNCCRRQIC<br>RNCCRRQICC	CPP15-384<br>CPP15-385	RYLQVLESKRNCCRR<br>VLESKRNCCRRQICC	CPP20-182<br>CPP20-183	DNAKRYLQVLESKR<br>NCCRRQ<br>KRYLQVLESKRNCCR<br>RQICC
Conotoxin<br>TxMEKL-011<br>Q9BPB7	CPP10-701<br>CPP10-702<br>CPP10-703<br>CPP10-704<br>CPP10-705<br>CPP10-706<br>CPP10-707<br>CPP10-708<br>CPP10-709<br>CPP10-710<br>CPP10-711	KLTILLLVAA<br>KENIKFLLKR<br>ENIKFLLKRK<br>NIKFLLKRKR<br>IKFLLKRKRA<br>KFLLKRKRAA<br>FLLKRKRAAD<br>LLKRKRAADR<br>LKRKRAADRG<br>KRKRAADRGM<br>RKRAADRGMW	CPP15-386<br>CPP15-387<br>CPP15-388<br>CPP15-389<br>CPP15-390<br>CPP15-391<br>CPP15-392<br>CPP15-393<br>CPP15-394<br>CPP15-395<br>CPP15-396<br>CPP15-397<br>CPP15-398	ENRSKENIKFLLKRK<br>NRSKENIKFLLKRKR<br>RSKENIKFLLKRKRA<br>SKENIKFLLKRKRAA<br>KENIKFLLKRKRAAD<br>ENIKFLLKRKRAADR<br>NIKFLLKRKRAADRG<br>IKFLLKRKRAADRGM<br>KFLLKRKRAADRGMW<br>FLLKRKRAADRGMWG<br>LKRKRAADRGMWGKC<br>RKRAADRGMWGKCKD<br>KRAADRGMWGKCKDG	CPP20-184<br>CPP20-185<br>CPP20-186<br>CPP20-187<br>CPP20-188<br>CPP20-189<br>CPP20-190<br>CPP20-191<br>CPP20-192	VERAGENRSKENIKF<br>LLKRK<br>ERAGENRSKENIKFL<br>LKRKR<br>RAGENRSKENIKFLL<br>KRKRA<br>AGENRSKENIKFLLK<br>RKRAA<br>KENIKFLLKRKRAAD<br>RGMWG<br>ENIKFLLKRKRAADR<br>GMWGK<br>NIKFLLKRKRAADRG<br>MWGKC<br>KFLLKRKRAADRGM<br>WGKCKD<br>FLLKRKRAADRGM<br>WGKCKDG
Conotoxin Tx-<br>D021<br>Q9BH21	CPP10-712	KRILQILQDR				
Conotoxin Tx-<br>D0111<br>Q9BPG7	CPP10-713<br>CPP10-714<br>CPP10-715<br>CPP10-716	HGNARRTLQM<br>GNARRTLQML<br>ARRTLQMLSK<br>RRTLQMLSKK	CPP15-399<br>CPP15-400<br>CPP15-401	FHGNARRTLQMLSKK<br>HGNARRTLQMLSKKQ<br>GNARRTLQMLSKKQC	CPP20-193	MPQASFHGNARRTL<br>QMLSKK

	Omega-conotoxin-like SVIA mutant 2<br>Q9XZL5	CPP10-717<br>CPP10-718<br>CPP10-719<br>CPP10-720<br>CPP10-721<br>CPP10-722<br>CPP10-723<br>CPP10-724<br>CPP10-725<br>CPP10-726<br>CPP10-727<br>CPP10-728<br>CPP10-729<br>CPP10-730	RGTQKHRTLR<br>GTQKHRTLRS<br>TQKHRTLRST<br>KHRTLRSTAR<br>HRTLRSTARR<br>RTLRSTARRS<br>TLRSTARRSK<br>LRSTARRSKS<br>RSTARRSKSE<br>RRSKSELTTR<br>KSELTTRCRP<br>ELTTRCRPSG<br>CCGRCSRGKC<br>CGRCSRGKCT	CPP15-402<br>CPP15-403<br>CPP15-404<br>CPP15-405<br>CPP15-406<br>CPP15-407<br>CPP15-408<br>CPP15-409<br>CPP15-410<br>CPP15-411<br>CPP15-412<br>CPP15-413<br>CPP15-414<br>CPP15-415	TAEDSRGTQKHRTLR<br>SRGTQKHRTLRSTAR<br>RGTQKHRTLRSTARR<br>GTQKHRTLRSTARRS<br>TQKHRTLRSTARRSK<br>QKHRTLRSTARRSKS<br>KHRTLRSTARRSKSE<br>HRTLRSTARRSKSEL<br>TLRSTARRSKSELTT<br>LRSTARRSKSELTTR<br>RSTARRSKSELTTRC<br>STARRSKSELTTRCR<br>TARRSKSELTTRCRP<br>RRSKSELTTRCRPSG	CPP20-194<br>CPP20-195<br>CPP20-196<br>CPP20-197<br>CPP20-198<br>CPP20-199<br>CPP20-200<br>CPP20-201<br>CPP20-202<br>CPP20-203	DSRGTQKHRTLRSTA<br>RRSKS<br>SRGTQKHRTLRSTAR<br>RSKSE<br>RGTQKHRTLRSTARR<br>SKSEL<br>QKHRTLRSTARRSKS<br>ELTTR<br>KHRTLRSTARRSKSE<br>LTTRC<br>HRTLRSTARRSKSELT<br>TRCR<br>RTLRSTARRSKSELTT<br>RCRP<br>TLRSTARRSKSELTTR<br>CRPS<br>LRSTARRSKSELTTRC<br>RPSG<br>RSTARRSKSELTTRC<br>RPSGS
	Conotoxin Tx8.1 (Fragment)<br>B2CI27	CPP10-731<br>CPP10-732<br>CPP10-733<br>CPP10-734<br>CPP10-735<br>CPP10-736<br>CPP10-737	GDIQARKTHL<br>DIQARKTHLK<br>QARKTHLKSD<br>KSDFYRTLPR<br>FYRTLPRFAR<br>YRTLPRFARG<br>RTLPRFARGC	CPP15-416<br>CPP15-417<br>CPP15-418<br>CPP15-419	SHREGDIQARKTHLK<br>GDIQARKTHLKSDFY<br>KSDFYRTLPRFARGC<br>FYRTLPRFARGCTIS	CPP20-204<br>CPP20-205<br>CPP20-206<br>CPP20-207	GDIQARKTHLKSDFY<br>RTLPR<br>DIQARKTHLKSDFYR<br>TLPRF<br>QARKTHLKSDFYRTL<br>PRFAR<br>RKTHLKSDFYRTLPR<br>FARGC
Chanos chanos	toxin MIT1-like<br>A0A6J2VNJ3	CPP10-738<br>CPP10-739<br>CPP10-740<br>CPP10-741<br>CPP10-742<br>CPP10-743	SLWIRSLRMC<br>LWIRSLRMCT<br>WIRSLRMCTP<br>KVPFFGKRLH<br>GKRLHHTCPC<br>GKYKCLSPYK	CPP15-420<br>CPP15-421<br>CPP15-422<br>CPP15-423	MKSSFLILCCVLMLS<br>AVSLWIRSLRMCTPM<br>SHKVPFFGKRLHHTC<br>VPFFGKRLHHTCPCL		
	stonustoxin subunit beta-like<br>A0A6J2VWL8	CPP10-744<br>CPP10-745<br>CPP10-746<br>CPP10-747<br>CPP10-748<br>CPP10-749<br>CPP10-750<br>CPP10-751	KEKVKHFSCK<br>PLKVVWLYPLK<br>KLAELLPTIR<br>PHVTDIPFRK<br>RGDTQRYRDN<br>TQRYRDNSKR<br>RYRDNSKRFQ<br>KGITRSDTKI				

	stonustoxin subunit beta-like<br>A0A6J2VWA1	CPP10-752<br>CPP10-753<br>CPP10-754<br>CPP10-755<br>CPP10-756<br>CPP10-757<br>CPP10-758<br>CPP10-759<br>CPP10-760	PLKVWLYPLK<br>KLTELLPTIR<br>KITKTTEDLR<br>RRGINIRLPD<br>RGINIRLPDQ<br>VRWYRNGTCR<br>RWYRNGTCRD<br>NKKVTRLGPP<br>KKVTRLGPPS	CPP15-424	GASVRWYRNGTCRDP	CPP20-208	KITKTTEDLRRGINIR<br>LPDQ
	ly-6/neurotoxin-like protein 1<br>A0A6J2WIV0	CPP10-761	AAEALTCKKC				
	toxin MIT1-like<br>A0A6J2VNJ3	CPP10-762<br>CPP10-763<br>CPP10-764<br>CPP10-765<br>CPP10-766<br>CPP10-767	SLWIRSLRMC<br>LWIRSLRMCT<br>WIRSLRMCTP<br>KVPFFGKRLH<br>GKRLHHTCPC<br>GKYKCLSPYK	CPP15-425<br>CPP15-426<br>CPP15-427	CAVSLWIRSLRMCTP<br>MSHKVPFFGKRLHHT<br>KVPFFGKRLHHTCPC		
Hydrophis lapemoides	Short neurotoxin 1<br>P01437	CPP10-768<br>CPP10-769<br>CPP10-770<br>CPP10-771<br>CPP10-772	CYKKTWRDFR<br>KKTWRDFRGT<br>KTWRDFRGTR<br>RDFRGTRIER<br>FRGTRIERGC	CPP15-428<br>CPP15-429<br>CPP15-430<br>CPP15-431	CYKKTWRDFRGTRIE<br>YKKTWRDFRGTRIER<br>KTWRDFRGTRIERGC<br>TWRDFRGTRIERGCG	CPP20-209	KKTWRDFRGTRIER<br>GCGCPQ
Plotosus lineatus	PL-toxin II<br>F2ZAL6	CPP10-773<br>CPP10-774<br>CPP10-775<br>CPP10-776<br>CPP10-777<br>CPP10-778	LGAIKFKTNR<br>GAIKFKTNRS<br>AIKFKTNRSR<br>KIETSKKITK<br>KKITKTSSWS<br>GRAAFDLPYK	CPP15-432<br>CPP15-433<br>CPP15-434	GAGTRLGAIKFKTNR<br>GTRLGAIKFKTNRSR<br>KIETSKKITKTSSWS		
	PL-toxin I<br>F2ZAL5	CPP10-779<br>CPP10-780<br>CPP10-781<br>CPP10-782<br>CPP10-783<br>CPP10-784<br>CPP10-785	WQIKSMKIWL<br>LGAIKFKTNR<br>GAIKFKTNRS<br>AIKFKTNRSR<br>KIETSKKITK<br>KKITKTSSWS<br>GRAAFDLPYK	CPP15-435<br>CPP15-436<br>CPP15-437	GAGTRLGAIKFKTNR<br>GTRLGAIKFKTNRSR<br>KIETSKKITKTSSWS		
Scorpaenopsis oxycephala	Tx alpha-subunit<br>A0A068BD77	CPP10-786<br>CPP10-787<br>CPP10-788<br>CPP10-789<br>CPP10-790	MLYDARREKL<br>YDARREKLIP<br>NQSRVTLKYK<br>SRVTLKYKVT<br>PMKVWLVPLK	CPP15-438	TKKYQNQSRVTLKYK	CPP20-210<br>CPP20-211<br>CPP20-212	KYLNNTKKYQNQSR<br>VTLKYK<br>MKQKALAFSEFAKKL<br>EKSRN<br>SKHPERFDNYRQVL

		CPP10-791<br>CPP10-792<br>CPP10-793<br>CPP10-794<br>CPP10-795<br>CPP10-796<br>CPP10-797<br>CPP10-798<br>CPP10-799<br>CPP10-800<br>CPP10-801<br>CPP10-802<br>CPP10-803<br>CPP10-804	MKVWLVPLKK<br>KVWLVPLKKF<br>LHEQRRRCNE<br>VQQTIAKKLP<br>QTIAKKLPLI<br>TIAKKLPLIR<br>SRMVIKMKQK<br>RMVIKMKQKA<br>FAKKLEKSRN<br>KTIKDRSDLL<br>GKHQKYSKHP<br>HQKYSKHPER<br>RQVLCKEPLR<br>KEPLRGRHYW				CKEPLR
	Tx beta-subunit<br>A0A068BD90	CPP10-805<br>CPP10-806<br>CPP10-807<br>CPP10-808<br>CPP10-809<br>CPP10-810<br>CPP10-811<br>CPP10-812<br>CPP10-813<br>CPP10-814<br>CPP10-815<br>CPP10-816<br>CPP10-817<br>CPP10-818<br>CPP10-819<br>CPP10-820<br>CPP10-821<br>CPP10-822<br>CPP10-823<br>CPP10-824	KYLNDTKKFK<br>KKFKNQSRVT<br>SSTPILRKVR<br>STPILRKVRN<br>TPILRKVRNT<br>KKLKTFQKHY<br>KLKTFQKHYR<br>MTNLRRTISQ<br>NLRRTISQKL<br>DFSKSIKPKK<br>FSKSIKPKKF<br>SKSIKPKKFS<br>KSIKPKKFSP<br>SIKPKKFSPS<br>IKPKKFSPSK<br>KPKKFSPSKK<br>KKFSPSKKDY<br>KKAMSGKPQP<br>GITYKAIQRK<br>YKAIQRKTSD	CPP15-439<br>CPP15-440<br>CPP15-441<br>CPP15-442<br>CPP15-443<br>CPP15-444<br>CPP15-445<br>CPP15-446	GSAKYLNDTKKFKNQ<br>MTNLRRTISQKLQSI<br>NLRRTISQKLQSIRK<br>DFSKSIKPKKFSPSK<br>FSKSIKPKKFSPSKK<br>SKSIKPKKFSPSKKD<br>KSIKPKKFSPSKKDY<br>KPKKFSPSKKDYWYT	CPP20-213<br>CPP20-214<br>CPP20-215<br>CPP20-216<br>CPP20-217<br>CPP20-218	LAALGRPFTLGTLYD<br>ARKDK<br>KYLNDTKKFKNQSRV<br>TLQYK<br>KKLKTFQKHYRDYM<br>TNLRRT<br>KLKTFQKHYRDYMT<br>NLRRTI<br>RDYMTNLRRTISQKL<br>QSIRK<br>KSIKPKKFSPSKKDY<br>WYTSD
Pardachirus marmoratus	Pardaxin P-4<br>P81861	CPP10-825<br>CPP10-826	PKIISSPLFK<br>KIISSPLFKT	CPP15-447	KIISSPLFKTLLSAV		
	Pardaxin P-5<br>P81862	CPP10-827<br>CPP10-828	PKIISSPLFK<br>KIISSPLFKT	CPP15-448	KIISSPLFKTLLSAV		

Epinephelus coioides	Neoverrucotoxin subunit-like protein (Fragment) F6KMK9	CPP10-829<br>CPP10-830<br>CPP10-831<br>CPP10-832<br>CPP10-833<br>CPP10-834<br>CPP10-835<br>CPP10-836<br>CPP10-837<br>CPP10-838	YSNMRNKAKA<br>NMRNKAKAFR<br>RNKAKAFRDF<br>KAKAFRDFAK<br>KAFRDFAKAQ<br>KAQKNNNRLR<br>KNNNRLRILI<br>NNRLRILIAT<br>RLRILIATIA<br>HLYTFRTKFT	CPP15-449<br>CPP15-450<br>CPP15-451	MRNKAKAFRDFAKAQ<br>KAKAFRDFAKAQKNN<br>KAQKNNNRLRILIAT	CPP20-219<br>CPP20-220<br>CPP20-221	KAKAFRDFAKAQKN<br>NNRLRI<br>KAFRDFAKAQKNNN<br>RLRILI<br>KNNNRLRILIATIANE<br>KYKG
Conus betulinus	Kappa-conotoxin BtX Q9U3Z3	CPP10-839<br>CPP10-840	CGHPCRHPGK<br>GHPCRHPGKR	CPP15-452	CWGGCGHPCRHPGKR		
	Neuropeptide Y1-like conopeptide P0CJ22	CPP10-841	RYFAIVGRPR				
	Conotoxin Bt6.5 Q3YEG7	CPP10-842<br>CPP10-843<br>CPP10-844<br>CPP10-845<br>CPP10-846<br>CPP10-847<br>CPP10-848<br>CPP10-849	KTYSTGRQKH<br>YSTGRQKHRA<br>TGRQKHRALR<br>GRQKHRALRS<br>RQKHRALRST<br>KHRALRSTDK<br>DKNIKLSRRC<br>KNIKLSRRCN	CPP15-453<br>CPP15-454<br>CPP15-455<br>CPP15-456<br>CPP15-457<br>CPP15-458<br>CPP15-459<br>CPP15-460<br>CPP15-461<br>CPP15-462	AKTYSTGRQKHRALR<br>KTYSTGRQKHRALRS<br>TYSTGRQKHRALRST<br>STGRQKHRALRSTDK<br>TGRQKHRALRSTDKN<br>GRQKHRALRSTDKNI<br>RQKHRALRSTDKNIK<br>QKHRALRSTDKNIKL<br>KHRALRSTDKNIKLS<br>ALRSTDKNIKLSRRC	CPP20-222<br>CPP20-223<br>CPP20-224<br>CPP20-225<br>CPP20-226<br>CPP20-227<br>CPP20-228<br>CPP20-229<br>CPP20-230	LATAKTYSTGRQKHR<br>ALRST<br>TAKTYSTGRQKHRAL<br>RSTDK<br>KTYSTGRQKHRALRS<br>TDKNI<br>TGRQKHRALRSTDK<br>NIKLSR<br>GRQKHRALRSTDKNI<br>KLSRR<br>RQKHRALRSTDKNIK<br>LSRRC<br>QKHRALRSTDKNIKL<br>SRRCN<br>KHRALRSTDKNIKLS<br>RRCND<br>HRALRSTDKNIKLSR<br>RCNDP
	Delta-conotoxin-like Bt6.4 M9PQ91	CPP10-850<br>CPP10-851<br>CPP10-852<br>CPP10-853	GWQKFFSKAR<br>SKARDEMKNR<br>KNRAASELNK<br>NRAASELNKR	CPP15-463	GWQKFFSKARDEMKN	CPP20-231	SKARDEMKNRAASE<br>LNKRCA
	Conotoxin Bt6.6 Q3YEF9	CPP10-854<br>CPP10-855	KLTILLLVAA<br>KINFLSRKST	CPP15-464	KRQQAKINFLSRKST		

Conotoxin Bt15a<br>B0KZ78	CPP10-856<br>CPP10-857<br>CPP10-858<br>CPP10-859<br>CPP10-860<br>CPP10-861<br>CPP10-862<br>CPP10-863<br>CPP10-864<br>CPP10-865<br>CPP10-866<br>CPP10-867<br>CPP10-868<br>CPP10-869<br>CPP10-870<br>CPP10-871<br>CPP10-872	DGEKPLKRRV<br>GEKPLKRRVK<br>EKPLKRRVKQ<br>KPLKRRVKQY<br>PLKRRVKQYA<br>KRRVKQYAAK<br>RRVKQYAAKR<br>RVKQYAAKRL<br>KQYAAKRLSA<br>AAKRLSALMR<br>KRLSALMRGP<br>RLSALMRGPR<br>MRGPRQCTPR<br>RGPRQCTPRN<br>GPRQCTPRNQ<br>PRQCTPRNQR<br>RQCTPRNQRC	CPP15-465<br>CPP15-466<br>CPP15-467<br>CPP15-468<br>CPP15-469<br>CPP15-470<br>CPP15-471<br>CPP15-472<br>CPP15-473<br>CPP15-474<br>CPP15-475<br>CPP15-476<br>CPP15-477	GEKPLKRRVKQYAAK<br>EKPLKRRVKQYAAKR<br>KPLKRRVKQYAAKRL<br>PLKRRVKQYAAKRLS<br>LKRRVKQYAAKRLSA<br>KRRVKQYAAKRLSAL<br>KQYAAKRLSALMRGP<br>QYAAKRLSALMRGPR<br>YAAKRLSALMRGPRQ<br>RLSALMRGPRQCTPR<br>LSALMRGPRQCTPRN<br>ALMRGPRQCTPRNQR<br>LMRGPRQCTPRNQRC	CPP20-232<br>CPP20-233<br>CPP20-234<br>CPP20-235<br>CPP20-236<br>CPP20-237<br>CPP20-238<br>CPP20-239<br>CPP20-240<br>CPP20-241<br>CPP20-242<br>CPP20-243	LAIQVLVQSDGEKPLKRRVK<br>QSDGEKPLKRRVKQYAAKRL<br>DGEKPLKRRVKQYAAKRLSA<br>KPLKRRVKQYAAKRLSALMR<br>PLKRRVKQYAAKRLSALMRG<br>LKRRVKQYAAKRLSALMRGP<br>KRRVKQYAAKRLSALMRGPR<br>RRVKQYAAKRLSALMRGPRQ<br>QYAAKRLSALMRGPRQCTPR<br>YAAKRLSALMRGPRQCTPRN<br>AKRLSALMRGPRQCTPRNQR<br>KRLSALMRGPRQCTPRNQRC
Conotoxin Bt5.1<br>Q3YEH6	CPP10-873<br>CPP10-874<br>CPP10-875<br>CPP10-876<br>CPP10-877<br>CPP10-878<br>CPP10-879<br>CPP10-880<br>CPP10-881<br>CPP10-882	PSVDARPKTK<br>SVDARPKTKA<br>NAKRTLQILR<br>AKRTLQILRN<br>KRTLQILRNK<br>RTLQILRNKR<br>TLQILRNKRA<br>LQILRNKRAC<br>QILRNKRACC<br>LRNKRACCPY	CPP15-478<br>CPP15-479<br>CPP15-480<br>CPP15-481<br>CPP15-482<br>CPP15-483<br>CPP15-484<br>CPP15-485	NDNAKRTLQILRNKR<br>DNAKRTLQILRNKRA<br>NAKRTLQILRNKRAC<br>AKRTLQILRNKRACC<br>KRTLQILRNKRACCP<br>RTLQILRNKRACCPY<br>TLQILRNKRACCPYE<br>LQILRNKRACCPYEP	CPP20-244<br>CPP20-245<br>CPP20-246	PLTSLNDNAKRTLQILRNKR<br>DNAKRTLQILRNKRACCPYE<br>KRTLQILRNKRACCPYEPSC
Conotoxin Bt11.1<br>P0C251	CPP10-883<br>CPP10-884<br>CPP10-885<br>CPP10-886<br>CPP10-887<br>CPP10-888<br>CPP10-889<br>CPP10-890	LSGATRRGDR<br>SGATRRGDRR<br>GATRRGDRRM<br>ATRRGDRRMC<br>TRRGDRRMCL<br>RRGDRRMCLS<br>RGDRRMCLSL<br>RRMCLSLGQR	CPP15-486<br>CPP15-487<br>CPP15-488<br>CPP15-489<br>CPP15-490<br>CPP15-491	GKPSERTLSGATRRG<br>SERTLSGATRRGDRR<br>SGATRRGDRRMCLSL<br>TRRGDRRMCLSLGQR<br>RRGDRRMCLSLGQRC<br>GQRCERHSNCCGYLC	CPP20-247<br>CPP20-248<br>CPP20-249<br>CPP20-250<br>CPP20-251	GKPSERTLSGATRRGDRRMC<br>SERTLSGATRRGDRRMCLSL<br>LSGATRRGDRRMCLSLGQRC<br>GATRRGDRRMCLSLGQRCER

	CPP10-891	GQRCERHSNC				RRGDRRMCLSLGQR CERHSN
Conotoxin Bt11.4 P0C609	CPP10-892	GQRCGRHSNC	CPP15-492	GQRCGRHSNCCGYLC		
Conotoxin A0A142C1B1	CPP10-893<br>CPP10-894<br>CPP10-895<br>CPP10-896<br>CPP10-897	HSPWFDPVRR<br>FDPVRRCCSR<br>PVRRCCSRDC<br>VRRCCSRDCR<br>RRCCSRDCRV	CPP15-493	SPWFDPVRRCCSRDC		
Conotoxin A0A142C187	CPP10-898<br>CPP10-899<br>CPP10-900<br>CPP10-901<br>CPP10-902<br>CPP10-903<br>CPP10-904<br>CPP10-905	DRESNHRNRR<br>RESNHRNRRA<br>SNHRNRRASN<br>NHRNRRASNQ<br>HRNRRASNQI<br>RNRRASNQIP<br>NRRASNQIPR<br>RRASNQIPRG	CPP15-494<br>CPP15-495<br>CPP15-496<br>CPP15-497<br>CPP15-498<br>CPP15-499	SFAADRESNHRNRRA<br>ADRESNHRNRRASNQ<br>DRESNHRNRRASNQI<br>ESNHRNRRASNQIPR<br>SNHRNRRASNQIPRG<br>NHRNRRASNQIPRGL	CPP20-252	FAADRESNHRNRRA SNQIPR
Conotoxin A0A142C1D4	CPP10-906<br>CPP10-907<br>CPP10-908<br>CPP10-909<br>CPP10-910	RNAAAKASNR<br>AAKASNRIAR<br>KASNRIARAI<br>SNRIARAISS<br>NRIARAISSG	CPP15-500	RNAAAKASNRIARAI		
Conotoxin A0A142C1D5	CPP10-911<br>CPP10-912<br>CPP10-913<br>CPP10-914<br>CPP10-915<br>CPP10-916	FTSDRAFRGR<br>SDRAFRGRNP<br>DRAFRGRNPA<br>RAFRGRNPAA<br>RRGCCSHPAC<br>NHPELCGRRR	CPP15-501<br>CPP15-502<br>CPP15-503<br>CPP15-504	SFTSDRAFRGRNPAA<br>SDRAFRGRNPAANDK<br>DRAFRGRNPAANDKR<br>RAFRGRNPAANDKRS	CPP20-253	VSFTSDRAFRGRNPA ANDKR
Alpha-conopeptide Bt1.5 A0A075IR52	CPP10-917<br>CPP10-918	RNAAAKASNR<br>NRIALIVRNA				
M superfamily MMSK group conopeptide Bt3-IP01 H2BKR1	CPP10-919	HSPWFDPVRR				
Alpha-conopeptide Bt1.4 A0A075IP10	CPP10-920<br>CPP10-921<br>CPP10-922<br>CPP10-923	FTSDRAFRGR<br>SDRAFRGRNS<br>DRAFRGRNSA<br>RRGCCSHPAC	CPP15-505<br>CPP15-506	SDRAFRGRNSAANDK<br>DRAFRGRNSAANDKR		

Alpha-conotoxin Bt1.91 F5BDH1	CPP10-924<br>CPP10-925<br>CPP10-926<br>CPP10-927	NMIALLIIRK<br>MIALLIIRKC<br>LLIIRKCCSN<br>LIIRKCCSNP	CPP15-507	NMIALLIIRKCCSNP	CPP20-254	ASGGRRSGADNMIA LLIIRK
Alpha-conotoxin Bt1.61 F5BDH0	CPP10-928	VIRAIKLTCC				
Alpha-conopeptide Bt1.1 A0A075ILT2	CPP10-929<br>CPP10-930<br>CPP10-931<br>CPP10-932<br>CPP10-933	GGRKAAAKAS<br>GRKAAAKASN<br>RKAAAKASNR<br>KAAAKASNRI<br>NRIALTVRSA	CPP15-508<br>CPP15-509<br>CPP15-510	GASGGRKAAAKASNR<br>ASGGRKAAAKASNRI<br>KAAAKASNRIALTVR	CPP20-255	GGRKAAAKASNRIAL TVRSA
Conotoxin A0A142C1P8	CPP10-934<br>CPP10-935<br>CPP10-936<br>CPP10-937<br>CPP10-938<br>CPP10-939	KRTLQILLNK<br>RTLQILLNKR<br>LQILLNKRPC<br>QILLNKRPCC<br>LLNKRPCCPR<br>NKRPCCPRDT	CPP15-511<br>CPP15-512<br>CPP15-513<br>CPP15-514<br>CPP15-515<br>CPP15-516<br>CPP15-517<br>CPP15-518<br>CPP15-519<br>CPP15-520	DDNAKRTLQILLNKR<br>DNAKRTLQILLNKRP<br>NAKRTLQILLNKRPC<br>AKRTLQILLNKRPCC<br>KRTLQILLNKRPCCP<br>RTLQILLNKRPCCPR<br>TLQILLNKRPCCPRD<br>LQILLNKRPCCPRDT<br>LLNKRPCCPRDTWCC<br>KRPCCPRDTWCCGFP	CPP20-256<br>CPP20-257	DNAKRTLQILLNKRP CCPRD<br>KRTLQILLNKRPCCPR DTWC
Conotoxin A0A142C1C7	CPP10-940<br>CPP10-941<br>CPP10-942<br>CPP10-943<br>CPP10-944<br>CPP10-945<br>CPP10-946<br>CPP10-947<br>CPP10-948<br>CPP10-949<br>CPP10-950<br>CPP10-951	SLRDFAKKGR<br>LRDFAKKGRK<br>RDFAKKGRKN<br>DFAKKGRKNL<br>FAKKGRKNLW<br>AKKGRKNLWR<br>KKGRKNLWRR<br>KGRKNLWRRS<br>GRKNLWRRSE<br>RKNLWRRSEC<br>KNLWRRSECC<br>LWRRSECCIR	CPP15-521<br>CPP15-522<br>CPP15-523<br>CPP15-524<br>CPP15-525<br>CPP15-526<br>CPP15-527<br>CPP15-528<br>CPP15-529<br>CPP15-530<br>CPP15-531<br>CPP15-532	TLASLRDFAKKGRKN<br>LASLRDFAKKGRKNL<br>SLRDFAKKGRKNLWR<br>LRDFAKKGRKNLWRR<br>RDFAKKGRKNLWRRS<br>DFAKKGRKNLWRRSE<br>FAKKGRKNLWRRSEC<br>AKKGRKNLWRRSECC<br>KKGRKNLWRRSECCI<br>KGRKNLWRRSECCIR<br>GRKNLWRRSECCIRN<br>RKNLWRRSECCIRNF	CPP20-258<br>CPP20-259<br>CPP20-260<br>CPP20-261<br>CPP20-262<br>CPP20-263<br>CPP20-264<br>CPP20-265<br>CPP20-266	PKTKNSMTLASLRDF AKKGR<br>TKNSMTLASLRDFAK KGRKN<br>KNSMTLASLRDFAKK GRKNL<br>MTLASLRDFAKKGR KNLWRR<br>TLASLRDFAKKGRKN LWRRS<br>LASLRDFAKKGRKNL WRRSE<br>SLRDFAKKGRKNLW RRSECC<br>FAKKGRKNLWRRSE CCIRNF<br>KGRKNLWRRSECCIR NFLCC

Conotoxin A0A142C1Q1	CPP10-952<br>CPP10-953<br>CPP10-954<br>CPP10-955<br>CPP10-956<br>CPP10-957<br>CPP10-958<br>CPP10-959<br>CPP10-960<br>CPP10-961<br>CPP10-962<br>CPP10-963<br>CPP10-964<br>CPP10-965	FIILLLLVSP<br>SKLHGLLTRR<br>KLHGLLTRRS<br>HGLLTRRSLK<br>GLLTRRSLKN<br>LLTRRSLKNF<br>TRRSLKNFWK<br>RRSLKNFWKR<br>RSLKNFWKRN<br>SLKNFWKRNL<br>LKNFWKRNLY<br>KNFWKRNLYL<br>NFWKRNLYLR<br>FWKRNLYLRD	CPP15-533<br>CPP15-534<br>CPP15-535<br>CPP15-536<br>CPP15-537<br>CPP15-538<br>CPP15-539<br>CPP15-540<br>CPP15-541	KLHGLLTRRSLKNFW<br>LHGLLTRRSLKNFWK<br>HGLLTRRSLKNFWKR<br>GLLTRRSLKNFWKRN<br>LLTRRSLKNFWKRNL<br>LTRRSLKNFWKRNLY<br>RRSLKNFWKRNLYLR<br>RSLKNFWKRNLYLRD<br>SLKNFWKRNLYLRDE	CPP20-267<br>CPP20-268<br>CPP20-269<br>CPP20-270<br>CPP20-271<br>CPP20-272	VTSKLHGLLTRRSLKNFWKR<br>KLHGLLTRRSLKNFWKRNLY<br>LHGLLTRRSLKNFWKRNLYL<br>HGLLTRRSLKNFWKRNLYLR<br>GLLTRRSLKNFWKRNLYLRD<br>LLTRRSLKNFWKRNLYLRDE
Conotoxin A0A142C1D2	CPP10-966<br>CPP10-967<br>CPP10-968<br>CPP10-969<br>CPP10-970<br>CPP10-971<br>CPP10-972<br>CPP10-973<br>CPP10-974<br>CPP10-975	PSVDARPKTK<br>SVDARPKTKA<br>NAKRTLQILR<br>AKRTLQILRN<br>KRTLQILRNK<br>RTLQILRNKR<br>TLQILRNKRA<br>LQILRNKRAC<br>QILRNKRACC<br>LRNKRACCPY	CPP15-542<br>CPP15-543<br>CPP15-544<br>CPP15-545<br>CPP15-546<br>CPP15-547<br>CPP15-548<br>CPP15-549	NDNAKRTLQILRNKR<br>DNAKRTLQILRNKRA<br>NAKRTLQILRNKRAC<br>AKRTLQILRNKRACC<br>KRTLQILRNKRACCP<br>RTLQILRNKRACCPY<br>TLQILRNKRACCPYE<br>LQILRNKRACCPYEP	CPP20-273<br>CPP20-274<br>CPP20-275	PLTSLNDNAKRTLQILRNKR<br>DNAKRTLQILRNKRACCPYE<br>KRTLQILRNKRACCPYEPSC
Conotoxin A0A142C1C8	CPP10-976<br>CPP10-977<br>CPP10-978<br>CPP10-979<br>CPP10-980<br>CPP10-981	FIILLLLIPS<br>DNAKRALQRY<br>AKRALQRYWA<br>KRALQRYWAK<br>RALQRYWAKS<br>LQRYWAKSLC	CPP15-550<br>CPP15-551<br>CPP15-552	DNAKRALQRYWAKSL<br>NAKRALQRYWAKSLC<br>KRALQRYWAKSLCCP	CPP20-276<br>CPP20-277<br>CPP20-278	FIILLLLIPSAASVAQPKTK<br>DNAKRALQRYWAKSLCCPED<br>KRALQRYWAKSLCCPEDRWC
Conotoxin A0A142C1C0	CPP10-982	RWCCPNGPRM	CPP15-553	GSLQIRWCCPNGPRM		
Conotoxin A0A142C1P9	CPP10-983<br>CPP10-984<br>CPP10-985<br>CPP10-986<br>CPP10-987<br>CPP10-988<br>CPP10-989<br>CPP10-990<br>CPP10-991	FIILLLLVSP<br>PAATLPVKSK<br>LHGFLTRRSL<br>HGFLTRRSLK<br>GFLTRRSLKD<br>FLTRRSLKDF<br>TRRSLKDFWK<br>RRSLKDFWKR<br>RSLKDFWKRH	CPP15-554<br>CPP15-555<br>CPP15-556<br>CPP15-557<br>CPP15-558<br>CPP15-559<br>CPP15-560<br>CPP15-561	KSKLHGFLTRRSLKD<br>KLHGFLTRRSLKDFW<br>LHGFLTRRSLKDFWK<br>HGFLTRRSLKDFWKR<br>GFLTRRSLKDFWKRH<br>LTRRSLKDFWKRHFY<br>RRSLKDFWKRHFYLR<br>RSLKDFWKRHFYLRD	CPP20-279<br>CPP20-280<br>CPP20-281<br>CPP20-282<br>CPP20-283<br>CPP20-284	VKSKLHGFLTRRSLKDFWKR<br>KSKLHGFLTRRSLKDFWKRH<br>KLHGFLTRRSLKDFWKRHFY<br>HGFLTRRSLKDFWKRHFYLR<br>GFLTRRSLKDFWKR

	CPP10-992<br>CPP10-993<br>CPP10-994	KDFWKRHFYL<br>DFWKRHFYLR<br>FWKRHFYLRD				HFYLRD<br>FLTRRSLKDFWKRHF<br>YLRDE
Conotoxin<br>A0A142C1J1	CPP10-995<br>CPP10-996	NAKRTLQMLR<br>AKRTLQMLRG				
Conotoxin<br>S4UJY9	CPP10-997<br>CPP10-998<br>CPP10-999<br>CPP10-1000<br>CPP10-1001<br>CPP10-1002<br>CPP10-1003<br>CPP10-1004<br>CPP10-1005<br>CPP10-1006<br>CPP10-1007<br>CPP10-1008<br>CPP10-1009<br>CPP10-1010	FIILLLLVSP<br>SKLHGLLTRR<br>KLHGLLTRRS<br>HGLLTRRSLK<br>GLLTRRSLKN<br>LLTRRSLKNF<br>TRRSLKNFWK<br>RRSLKNFWKR<br>RSLKNFWKRN<br>SLKNFWKRNL<br>LKNFWKRNLY<br>KNFWKRNLYL<br>NFWKRNLYLR<br>FWKRNLYLRD	CPP15-562<br>CPP15-563<br>CPP15-564<br>CPP15-565<br>CPP15-566<br>CPP15-567<br>CPP15-568<br>CPP15-569<br>CPP15-570	KLHGLLTRRSLKNFW<br>LHGLLTRRSLKNFWK<br>HGLLTRRSLKNFWKR<br>GLLTRRSLKNFWKRN<br>LLTRRSLKNFWKRNL<br>LTRRSLKNFWKRNLY<br>RRSLKNFWKRNLYLR<br>RSLKNFWKRNLYLRD<br>SLKNFWKRNLYLRDE	CPP20-285<br>CPP20-286<br>CPP20-287<br>CPP20-288<br>CPP20-289<br>CPP20-290	VTSKLHGLLTRRSLK<br>NFWKR<br>KLHGLLTRRSLKNFW<br>KRNLY<br>LHGLLTRRSLKNFWK<br>RNLYL<br>HGLLTRRSLKNFWK<br>RNLYLR<br>GLLTRRSLKNFWKR<br>NLYLRD<br>LLTRRSLKNFWKRNL<br>YLRDE
Conotoxin<br>A0A1P7ZCP5	CPP10-1011	RWCCPNGPRF	CPP15-571	GSLQIRWCCPNGPRF		
Conotoxin<br>S4UKA5	CPP10-1012<br>CPP10-1013	MRNEHQLLKK<br>RNEHQLLKKV				
Conotoxin<br>S4UJR6	CPP10-1014<br>CPP10-1015<br>CPP10-1016<br>CPP10-1017<br>CPP10-1018<br>CPP10-1019<br>CPP10-1020<br>CPP10-1021<br>CPP10-1022<br>CPP10-1023<br>CPP10-1024<br>CPP10-1025	FIILLLLVSP<br>PAATLLVKSK<br>LHGFLTRRSL<br>HGFLTRRSLK<br>GFLTRRSLKD<br>FLTRRSLKDF<br>TRRSLKDFWK<br>RRSLKDFWKR<br>RSLKDFWKRH<br>KDFWKRHFYL<br>DFWKRHFYLR<br>FWKRHFYLRD	CPP15-572<br>CPP15-573<br>CPP15-574<br>CPP15-575<br>CPP15-576<br>CPP15-577<br>CPP15-578<br>CPP15-579	KSKLHGFLTRRSLKD<br>KLHGFLTRRSLKDFW<br>LHGFLTRRSLKDFWK<br>HGFLTRRSLKDFWKR<br>GFLTRRSLKDFWKRH<br>LTRRSLKDFWKRHFY<br>RRSLKDFWKRHFYLR<br>RSLKDFWKRHFYLRD	CPP20-291<br>CPP20-292<br>CPP20-293<br>CPP20-294<br>CPP20-295<br>CPP20-296<br>CPP20-297<br>CPP20-298	VFIILLLLVSPAATLLV<br>KSK<br>FIILLLLVSPAATLLVK<br>SKL<br>VKSKLHGFLTRRSLK<br>DFWKR<br>KSKLHGFLTRRSLKD<br>FWKRH<br>KLHGFLTRRSLKDFW<br>KRHFY<br>HGFLTRRSLKDFWK<br>RHFYLR<br>GFLTRRSLKDFWKR<br>HFYLRD<br>FLTRRSLKDFWKRHF

							YLRDE
	Conotoxin<br>(Fragment)<br>A0A142C1Q5	CPP10-1026<br>CPP10-1027<br>CPP10-1028<br>CPP10-1029<br>CPP10-1030	GGRKAAAKAS<br>GRKAAAKASN<br>RKAAAKASNR<br>KAAAKASNRI<br>NRIALTVRSA	CPP15-580<br>CPP15-581	ASGGRKAAAKASNRI<br>KAAAKASNRIALTVR	CPP20-299	GGRKAAAKASNRIAL<br>TVRSA
	Conotoxin Bt14.5<br>(Fragment)<br>A0A068B6R0	CPP10-1031<br>CPP10-1032<br>CPP10-1033<br>CPP10-1034<br>CPP10-1035	PDCRFNRGRC<br>DCRFNRGRCR<br>CRFNRGRCRP<br>RFNRGRCRPR<br>FNRGRCRPRA	CPP15-582<br>CPP15-583<br>CPP15-584	CMHPDCRFNRGRCRP<br>MHPDCRFNRGRCRPR<br>HPDCRFNRGRCRPRA	CPP20-300<br>CPP20-301	DCRLCMHPDCRFNR<br>GRCRPR<br>CRLCMHPDCRFNRG<br>RCRPRA
	Conotoxin Bt14.15<br>(Fragment)<br>A0A068B6S1	CPP10-1036	HKCVCLKTCR				
	Conotoxin Bt14.12<br>(Fragment)<br>A0A068B0Y2	CPP10-1037<br>CPP10-1038<br>CPP10-1039	DCRFNPGRCR<br>CRFNPGRCRQ<br>RFNPGRCRQR	CPP15-585<br>CPP15-586	MHPDCRFNPGRCRQR<br>HPDCRFNPGRCRQRE	CPP20-302<br>CPP20-303	DCKPCMHPDCRFNP<br>GRCRQR<br>CKPCMHPDCRFNPG<br>RCRQRE
	Conotoxin Bt14.16<br>(Fragment)<br>A0A068B409	CPP10-1040<br>CPP10-1041<br>CPP10-1042<br>CPP10-1043	DCRFNPGRCR<br>CRFNPGRCRP<br>RFNPGRCRPR<br>FNPGRCRPRE	CPP15-587<br>CPP15-588<br>CPP15-589	CMHPDCRFNPGRCRP<br>MHPDCRFNPGRCRPR<br>HPDCRFNPGRCRPRE	CPP20-304<br>CPP20-305	DCKPCMHPDCRFNP<br>GRCRPR<br>CKPCMHPDCRFNPG<br>RCRPRE
	Conotoxin Bt14.4<br>(Fragment)<br>A0A068B2G8	CPP10-1044	ACGINRGKCR				
	Conotoxin<br>(Fragment)<br>A0A142C1V7	CPP10-1045<br>CPP10-1046<br>CPP10-1047<br>CPP10-1048<br>CPP10-1049<br>CPP10-1050	KRTLQIHWNK<br>RTLQIHWNKR<br>LQIHWNKRCC<br>QIHWNKRCCP<br>HWNKRCCPEK<br>KRCCPEKIWC	CPP15-590<br>CPP15-591<br>CPP15-592<br>CPP15-593	DNAKRTLQIHWNKRC<br>KRTLQIHWNKRCCPE<br>RTLQIHWNKRCCPEK<br>QIHWNKRCCPEKIWC	CPP20-306<br>CPP20-307	DNAKRTLQIHWNKR<br>CCPEKI<br>KRTLQIHWNKRCCP<br>EKIWCC
	Conotoxin<br>A0A142C1G6	CPP10-1051<br>CPP10-1052<br>CPP10-1053<br>CPP10-1054<br>CPP10-1055<br>CPP10-1056	HSPWFDPVRR<br>FDPVRRCCSR<br>PVRRCCSRDC<br>VRRCCSRDCR<br>RRCCSRDCRV<br>RCCSRDCRVC	CPP15-594<br>CPP15-595<br>CPP15-596<br>CPP15-597	SPWFDPVRRCCSRDC<br>FDPVRRCCSRDCRVC<br>VRRCCSRDCRVCIPC<br>RRCCSRDCRVCIPCC		

Conus ferrugineus	Conopeptide Y-Fe1<br>B3SVF1	CPP10-1057<br>CPP10-1058<br>CPP10-1059<br>CPP10-1060	YYRLWRYFTR<br>YRLWRYFTRF<br>RLWRYFTRFL<br>LWRYFTRFLH	CPP15-598	YPFSYYRLWRYFTRF		
Conus caracteristicus	Conotoxin Ca6.2<br>V5V9Y7	CPP10-1061<br>CPP10-1062<br>CPP10-1063<br>CPP10-1064<br>CPP10-1065<br>CPP10-1066<br>CPP10-1067<br>CPP10-1068<br>CPP10-1069<br>CPP10-1070<br>CPP10-1071<br>CPP10-1072<br>CPP10-1073<br>CPP10-1074<br>CPP10-1075<br>CPP10-1076	RNPSEARRAY<br>PSEARRAYKR<br>SEARRAYKRL<br>EARRAYKRLL<br>ARRAYKRLLQ<br>RRAYKRLLQR<br>RAYKRLLQRP<br>AYKRLLQRPA<br>YKRLLQRPAR<br>KRLLQRPARR<br>RLLQRPARRM<br>LLQRPARRMD<br>LQRPARRMDR<br>QRPARRMDRG<br>RPARRMDRGG<br>PARRMDRGGC	CPP15-599<br>CPP15-600<br>CPP15-601<br>CPP15-602<br>CPP15-603<br>CPP15-604<br>CPP15-605<br>CPP15-606<br>CPP15-607<br>CPP15-608<br>CPP15-609<br>CPP15-610<br>CPP15-611<br>CPP15-612<br>CPP15-613<br>CPP15-614<br>CPP15-615	GGDRNPSEARRAYKR<br>GDRNPSEARRAYKRL<br>DRNPSEARRAYKRLL<br>RNPSEARRAYKRLLQ<br>NPSEARRAYKRLLQR<br>PSEARRAYKRLLQRP<br>SEARRAYKRLLQRPA<br>EARRAYKRLLQRPAR<br>ARRAYKRLLQRPARR<br>RRAYKRLLQRPARRM<br>RAYKRLLQRPARRMD<br>AYKRLLQRPARRMDR<br>YKRLLQRPARRMDRG<br>KRLLQRPARRMDRGG<br>RLLQRPARRMDRGGC<br>LQRPARRMDRGGCTP<br>QRPARRMDRGGCTPC	CPP20-308<br>CPP20-309<br>CPP20-310<br>CPP20-311<br>CPP20-312<br>CPP20-313<br>CPP20-314<br>CPP20-315<br>CPP20-316<br>CPP20-317<br>CPP20-318<br>CPP20-319<br>CPP20-320<br>CPP20-321	GQAVGGDRNPSEARRAYKRL<br>VGGDRNPSEARRAYKRLLQR<br>GGDRNPSEARRAYKRLLQRP<br>GDRNPSEARRAYKRLLQRPA<br>DRNPSEARRAYKRLLQRPAR<br>RNPSEARRAYKRLLQRPARR<br>NPSEARRAYKRLLQRPARRM<br>PSEARRAYKRLLQRPARRMD<br>SEARRAYKRLLQRPARRMDR<br>EARRAYKRLLQRPARRMDRG<br>ARRAYKRLLQRPARRMDRGG<br>RRAYKRLLQRPARRMDRGGC<br>RAYKRLLQRPARRMDRGGCT<br>YKRLLQRPARRMDRGGCTPC
Conus flavidus	Conotoxin Fla16d<br>V5V893	CPP10-1077<br>CPP10-1078<br>CPP10-1079<br>CPP10-1080<br>CPP10-1081	MLLLLLLLLP<br>LLLLLLLLPL<br>LLLLLLLPLA<br>FLQRLIRLIH<br>RLIRLIHGSD	CPP15-616	TLEMLLLLLLLLPLA		
	Alpha-conotoxin-like<br>U3L010	CPP10-1082	RGCCSDPPCR				

	A-conotoxin peptide Fla1.6 U3L0F2	CPP10-1083<br>CPP10-1084<br>CPP10-1085	DWIAQALKRC<br>WIAQALKRCC<br>CAHVNCRGRR	CPP15-617	WIAQALKRCCGNPPC		
	A-conotoxin peptide Fla6.2 (Fragment) U3KZW1	CPP10-1086	CAYPPCRHKH				
	A-conotoxin peptide Fla1.1 U3L069	CPP10-1087<br>CPP10-1088	AIKQCCRNPK<br>KQCCRNPKCS	CPP15-618	WIALAIKQCCRNPKC		
	A-conotoxin peptide Fla6.1 (Fragment) U3L0E9	CPP10-1089	CAYPPCRHKH				
	A-conotoxin peptide Fla1.7a (Fragment) U3KZV0	CPP10-1090	EICRRMLQNP				
	Conotoxin U3L0G6	CPP10-1091<br>CPP10-1092<br>CPP10-1093<br>CPP10-1094	AKRTLQRLWI<br>KRTLQRLWIK<br>RTLQRLWIKA<br>LQRLWIKALC	CPP15-619<br>CPP15-620<br>CPP15-621<br>CPP15-622<br>CPP15-623	FHDDAKRTLQRLWIK<br>HDDAKRTLQRLWIKA<br>DDAKRTLQRLWIKAL<br>AKRTLQRLWIKALCC<br>KRTLQRLWIKALCCY		
Conus monile	Kappa-conotoxin Mo1659 P84713	CPP10-1095	GSWYRFPWGY				
	Conopressin/cono physin, isoform 3 (Fragment) A0A4Y5X1A7	CPP10-1096<br>CPP10-1097<br>CPP10-1098	FIRNCPKGGK<br>IRNCPKGGKR<br>RNCPKGGKRN				
	Conophysin-conopressin, isoform 1 (Fragment) A0A291NVT7	CPP10-1099<br>CPP10-1100<br>CPP10-1101	GRPTLLPCLL<br>LLPCLLLLLV<br>HMIQPTKPCM				
	Conopressin/cono physin, isoform 2 (Fragment) A0A4Y5X186	CPP10-1102<br>CPP10-1103<br>CPP10-1104<br>CPP10-1105<br>CPP10-1106	GCFIRNCPRG<br>FIRNCPRGGK<br>IRNCPRGGKR<br>RNCPRGGKRA<br>LQPTRQCMSC	CPP15-624<br>CPP15-625<br>CPP15-626<br>CPP15-627<br>CPP15-628	QGCFIRNCPRGGKRA<br>GCFIRNCPRGGKRAV<br>DSVSARQELLTLIRR<br>RQELLTLIRRLLVNR<br>LLTLIRRLLVNRQYD	CPP20-322<br>CPP20-323	LLGTAQGCFIRNCPR GGKRA<br>FIRNCPRGGKRAVD ALQPTR

		CPP10-1107<br>CPP10-1108<br>CPP10-1109<br>CPP10-1110	RQELLTLIRR<br>ELLTLIRRLL<br>TLIRRLLVNR<br>LIRRLLVNRQ				
	Contryphan-Mo (Fragment)<br>A0A1P8NVU1	CPP10-1111<br>CPP10-1112<br>CPP10-1113<br>CPP10-1114<br>CPP10-1115<br>CPP10-1116<br>CPP10-1117<br>CPP10-1118<br>CPP10-1119	RDVNPGRARR<br>DVNPGRARRK<br>VNPGRARRKL<br>NPGRARRKLM<br>PGRARRKLMK<br>GRARRKLMKV<br>RARRKLMKVL<br>ARRKLMKVLR<br>RRKLMKVLRE	CPP15-629<br>CPP15-630<br>CPP15-631<br>CPP15-632<br>CPP15-633<br>CPP15-634<br>CPP15-635<br>CPP15-636	AVPRDVNPGRARRKL<br>PRDVNPGRARRKLMK<br>RDVNPGRARRKLMKV<br>DVNPGRARRKLMKVL<br>VNPGRARRKLMKVLR<br>NPGRARRKLMKVLRE<br>PGRARRKLMKVLRES<br>GRARRKLMKVLRESE	CPP20-324<br>CPP20-325<br>CPP20-326<br>CPP20-327<br>CPP20-328<br>CPP20-329<br>CPP20-330<br>CPP20-331<br>CPP20-332	QPADRNAVPRDVNPGRARRK<br>DRNAVPRDVNPGRARRKLMK<br>RNAVPRDVNPGRARRKLMKV<br>AVPRDVNPGRARRKLMKVLR<br>VPRDVNPGRARRKLMKVLRE<br>PGRARRKLMKVLRESECPWK<br>GRARRKLMKVLRESECPWKP<br>RARRKLMKVLRESECPWKPW<br>ARRKLMKVLRESECPWKPWC
	Conotoxin (Fragment)<br>A0A0P0C7W0	CPP10-1120<br>CPP10-1121	PIFILLLLLV<br>RKYLTQEFRK	CPP15-637<br>CPP15-638	RKYLTQEFRKDFERR<br>RRAGNPRGNWCCSAR	CPP20-333	RKYLTQEFRKDFERRAGNPR
Conus striatus	Mu-conotoxin SIIIA<br>Q86DU6	CPP10-1122<br>CPP10-1123<br>CPP10-1124<br>CPP10-1125	DQPADRPAER<br>SKWCRDHARC<br>KWCRDHARCC<br>CRDHARCCGR				
	Con-ikot-ikot<br>P0CB20	CPP10-1126<br>CPP10-1127<br>CPP10-1128<br>CPP10-1129<br>CPP10-1130<br>CPP10-1131	QEPDLSRMRR<br>PDLSRMRRSG<br>DLSRMRRSGP<br>LSRMRRSGPA<br>SRMRRSGPAD<br>RMRRSGPADC	CPP15-639<br>CPP15-640<br>CPP15-641<br>CPP15-642	QEPDLSRMRRSGPAD<br>DLSRMRRSGPADCCR<br>LSRMRRSGPADCCRM<br>SRMRRSGPADCCRMK		
	Mu-conotoxin SIIIC<br>P0C349	CPP10-1132<br>CPP10-1133<br>CPP10-1134<br>CPP10-1135	RHGCCKGPKG<br>KGCSSRECRP<br>GCSSRECRPQ<br>SSRECRPQHC				

Omega-conotoxin SVIA<br>P28880	CPP10-1136<br>CPP10-1137<br>CPP10-1138<br>CPP10-1139<br>CPP10-1140<br>CPP10-1141<br>CPP10-1142<br>CPP10-1143<br>CPP10-1144<br>CPP10-1145<br>CPP10-1146<br>CPP10-1147	RGTQKHRTLR<br>GTQKHRTLRS<br>TQKHRTLRST<br>KHRTLRSTAR<br>HRTLRSTARR<br>RTLRSTARRS<br>TLRSTARRSK<br>LRSTARRSKS<br>RSTARRSKSE<br>RRSKSESTTR<br>CCGRCYRGKC<br>CGRCYRGKCT	CPP15-643<br>CPP15-644<br>CPP15-645<br>CPP15-646<br>CPP15-647<br>CPP15-648<br>CPP15-649<br>CPP15-650<br>CPP15-651<br>CPP15-652<br>CPP15-653<br>CPP15-654	TAEDSRGTQKHRTLR<br>SRGTQKHRTLRSTAR<br>RGTQKHRTLRSTARR<br>GTQKHRTLRSTARRS<br>TQKHRTLRSTARRSK<br>QKHRTLRSTARRSKS<br>KHRTLRSTARRSKSE<br>HRTLRSTARRSKSES<br>TLRSTARRSKSESTT<br>LRSTARRSKSESTTR<br>RSTARRSKSESTTRC<br>STARRSKSESTTRCR	CPP20-334<br>CPP20-335<br>CPP20-336<br>CPP20-337<br>CPP20-338<br>CPP20-339<br>CPP20-340<br>CPP20-341<br>CPP20-342	DSRGTQKHRTLRSTA<br>RRSKS<br>SRGTQKHRTLRSTAR<br>RSKSE<br>RGTQKHRTLRSTARR<br>SKSES<br>GTQKHRTLRSTARRS<br>KSEST<br>QKHRTLRSTARRSKS<br>ESTTR<br>KHRTLRSTARRSKSE<br>STTRC<br>HRTLRSTARRSKSES<br>TTRCR<br>TLRSTARRSKSESTTR<br>CRSS<br>RSTARRSKSESTTRC<br>RSSGS
Omega-conotoxin SO-3<br>Q9XZK2	CPP10-1148<br>CPP10-1149<br>CPP10-1150<br>CPP10-1151<br>CPP10-1152<br>CPP10-1153<br>CPP10-1154<br>CPP10-1155<br>CPP10-1156<br>CPP10-1157<br>CPP10-1158<br>CPP10-1159<br>CPP10-1160	RGTQKHRTLR<br>GTQKHRTLRS<br>TQKHRTLRSK<br>QKHRTLRSKT<br>KHRTLRSKTK<br>HRTLRSKTKL<br>RTLRSKTKLS<br>TLRSKTKLSM<br>RSKTKLSMST<br>KLSMSTRCKA<br>STRCKAAGKP<br>TRCKAAGKPC<br>CKAAGKPCSR	CPP15-655<br>CPP15-656<br>CPP15-657<br>CPP15-658<br>CPP15-659<br>CPP15-660<br>CPP15-661<br>CPP15-662<br>CPP15-663<br>CPP15-664<br>CPP15-665<br>CPP15-666<br>CPP15-667	TADDSRGTQKHRTLR<br>DDSRGTQKHRTLRSK<br>DSRGTQKHRTLRSKT<br>SRGTQKHRTLRSKTK<br>RGTQKHRTLRSKTKL<br>GTQKHRTLRSKTKLS<br>TQKHRTLRSKTKLSM<br>QKHRTLRSKTKLSMS<br>KHRTLRSKTKLSMST<br>HRTLRSKTKLSMSTR<br>TLRSKTKLSMSTRCK<br>KLSMSTRCKAAGKPC<br>CKAAGKPCSRIAYNC	CPP20-343<br>CPP20-344<br>CPP20-345<br>CPP20-346<br>CPP20-347<br>CPP20-348<br>CPP20-349<br>CPP20-350<br>CPP20-351<br>CPP20-352<br>CPP20-353<br>CPP20-354	QLITADDSRGTQKH<br>RTLRSK<br>ITADDSRGTQKHRTL<br>RSKTK<br>SRGTQKHRTLRSKTK<br>LSMST<br>RGTQKHRTLRSKTKL<br>SMSTR<br>GTQKHRTLRSKTKLS<br>MSTRC<br>TQKHRTLRSKTKLSM<br>STRCK<br>QKHRTLRSKTKLSMS<br>TRCKA<br>KHRTLRSKTKLSMST<br>RCKAA<br>HRTLRSKTKLSMSTR<br>CKAAG<br>TLRSKTKLSMSTRCK<br>AAGKP<br>RSKTKLSMSTRCKAA<br>GKPCS<br>SKTKLSMSTRCKAAG<br>KPCSR

Omega-conotoxin<br>SVIB<br>P28881	CPP10-1161<br>CPP10-1162<br>CPP10-1163<br>CPP10-1164<br>CPP10-1165<br>CPP10-1166<br>CPP10-1167<br>CPP10-1168<br>CPP10-1169<br>CPP10-1170<br>CPP10-1171<br>CPP10-1172	RGTQKHRALR<br>GTQKHRALRS<br>TQKHRALRSD<br>KHRALRSDTK<br>RALRSDTKLP<br>RSDTKLPMST<br>KLPMSTRCKL<br>LPMSTRCKLK<br>PMSTRCKLKG<br>MSTRCKLKGQ<br>CKLKGQSCRK<br>KGQSCRKTSY	CPP15-668<br>CPP15-669<br>CPP15-670<br>CPP15-671<br>CPP15-672<br>CPP15-673<br>CPP15-674<br>CPP15-675	SRGTQKHRALRSDTK<br>RGTQKHRALRSDTKL<br>GTQKHRALRSDTKLP<br>KHRALRSDTKLPMST<br>RSDTKLPMSTRCKLK<br>SDTKLPMSTRCKLKG<br>KLPMSTRCKLKGQSC<br>MSTRCKLKGQSCRKT	CPP20-355<br>CPP20-356<br>CPP20-357<br>CPP20-358<br>CPP20-359	QKHRALRSDTKLPM<br>STRCKL<br>KHRALRSDTKLPMST<br>RCKLK<br>HRALRSDTKLPMSTR<br>CKLKG<br>RALRSDTKLPMSTRC<br>KLKGQ<br>KLPMSTRCKLKGQS<br>CRKTSY
Delta-conotoxin<br>SVIE<br>Q9XZK5	CPP10-1173<br>CPP10-1174<br>CPP10-1175<br>CPP10-1176	YGLKNLFPKA<br>GLKNLFPKAR<br>PKARHEMKNP<br>KNPEASKLNK	CPP15-676	LKNLFPKARHEMKNP	CPP20-360<br>CPP20-361	DSRYGLKNLFPKARH<br>EMKNP<br>LFPKARHEMKNPEA<br>SKLNKR
Kunitz-type<br>conkunitzin-S1<br>P0C1X2	CPP10-1177<br>CPP10-1178<br>CPP10-1179<br>CPP10-1180	GTGTLLPKDR<br>LLPKDRPSLC<br>KRIYYNSARK<br>YNSARKQCLR	CPP15-677<br>CPP15-678<br>CPP15-679	LLPKDRPSLCDLPAD<br>KAEKRIYYNSARKQC<br>EKRIYYNSARKQCLR	CPP20-362	SGTKAEKRIYYNSAR<br>KQCLR
Contryphan-S<br>P0C837	CPP10-1181	MNVQRRSGCP				
Mu-conotoxin<br>SIIIB<br>P0CE77	CPP10-1182<br>CPP10-1183	SSKWCKGHAR<br>KWCKGHARCC				
Kappa-conotoxin-<br>like SIVB<br>P0C829	CPP10-1184<br>CPP10-1185<br>CPP10-1186<br>CPP10-1187<br>CPP10-1188<br>CPP10-1189<br>CPP10-1190<br>CPP10-1191<br>CPP10-1192	CRCTNSCPTK<br>RCTNSCPTKP<br>TNSCPTKPKK<br>NSCPTKPKKP<br>SCPTKPKKPG<br>CPTKPKKPGR<br>PTKPKKPGRR<br>TKPKKPGRRN<br>KPKKPGRRND	CPP15-680<br>CPP15-681<br>CPP15-682<br>CPP15-683<br>CPP15-684<br>CPP15-685	PCRCTNSCPTKPKKP<br>CRCTNSCPTKPKKPG<br>RCTNSCPTKPKKPGR<br>CTNSCPTKPKKPGRR<br>TNSCPTKPKKPGRRN<br>NSCPTKPKKPGRRND		
Omega-conotoxin-<br>like SVIA mutant 1<br>Q9XZL4	CPP10-1193<br>CPP10-1194<br>CPP10-1195<br>CPP10-1196<br>CPP10-1197<br>CPP10-1198<br>CPP10-1199	RGAQKHRTLR<br>GAQKHRTLRS<br>AQKHRTLRST<br>KHRTLRSTAR<br>HRTLRSTARR<br>RTLRSTARRS<br>TLRSTARRSK	CPP15-686<br>CPP15-687<br>CPP15-688<br>CPP15-689<br>CPP15-690<br>CPP15-691<br>CPP15-692	SRGAQKHRTLRSTAR<br>RGAQKHRTLRSTARR<br>GAQKHRTLRSTARRS<br>AQKHRTLRSTARRSK<br>QKHRTLRSTARRSKS<br>KHRTLRSTARRSKSE<br>HRTLRSTARRSKSEL	CPP20-363<br>CPP20-364<br>CPP20-365<br>CPP20-366<br>CPP20-367<br>CPP20-368<br>CPP20-369	DSRGAQKHRTLRST<br>ARRSKS<br>SRGAQKHRTLRSTA<br>RRSKSE<br>RGAQKHRTLRSTAR<br>RSKSEL<br>GAQKHRTLRSTARR

	CPP10-1200<br>CPP10-1201<br>CPP10-1202<br>CPP10-1203<br>CPP10-1204<br>CPP10-1205<br>CPP10-1206	LRSTARRSKS<br>RSTARRSKSE<br>RRSKSELTTR<br>KSELTTRCRP<br>ELTTRCRPSG<br>CCGRCYRGKC<br>CGRCYRGKCT	CPP15-693<br>CPP15-694<br>CPP15-695<br>CPP15-696<br>CPP15-697<br>CPP15-698	TLRSTARRSKSELTT<br>LRSTARRSKSELTTR<br>RSTARRSKSELTTRC<br>STARRSKSELTTRCR<br>TARRSKSELTTRCRP<br>RRSKSELTTRCRPSG	CPP20-370<br>CPP20-371<br>CPP20-372<br>CPP20-373	SKSELT<br>QKHRTLRSTARRSKS<br>ELTTR<br>KHRTLRSTARRSKSE<br>LTTRC<br>HRTLRSTARRSKSELT<br>TRCR<br>RTLRSTARRSKSELTT<br>RCRP<br>TLRSTARRSKSELTTR<br>CRPS<br>LRSTARRSKSELTTRC<br>RPSG<br>RSTARRSKSELTTRC<br>RPSGS
Omega-conotoxin-like SO-4<br>Q9XZK3	CPP10-1207<br>CPP10-1208<br>CPP10-1209<br>CPP10-1210<br>CPP10-1211<br>CPP10-1212<br>CPP10-1213	SRGTQKHRSL<br>RGTQKHRSLR<br>GTQKHRSLRS<br>TQKHRSLRST<br>KHRSLRSTTK<br>HRSLRSTTKV<br>SLRSTTKVSK	CPP15-699<br>CPP15-700<br>CPP15-701<br>CPP15-702<br>CPP15-703<br>CPP15-704<br>CPP15-705	TADDSRGTQKHRSLR<br>SRGTQKHRSLRSTTK<br>RGTQKHRSLRSTTKV<br>GTQKHRSLRSTTKVS<br>TQKHRSLRSTTKVSK<br>QKHRSLRSTTKVSKA<br>KHRSLRSTTKVSKAT	CPP20-374<br>CPP20-375	DSRGTQKHRSLRSTT<br>KVSKA<br>SRGTQKHRSLRSTTK<br>VSKAT
Omega-conotoxin-like SO-5<br>Q9XZK4	CPP10-1214<br>CPP10-1215<br>CPP10-1216<br>CPP10-1217<br>CPP10-1218<br>CPP10-1219<br>CPP10-1220	SRGTQKHRSL<br>RGTQKHRSLR<br>GTQKHRSLRS<br>TQKHRSLRST<br>KHRSLRSTTK<br>HRSLRSTTKV<br>SLRSTTKVSK	CPP15-706<br>CPP15-707<br>CPP15-708<br>CPP15-709<br>CPP15-710<br>CPP15-711<br>CPP15-712	TADDSRGTQKHRSLR<br>SRGTQKHRSLRSTTK<br>RGTQKHRSLRSTTKV<br>GTQKHRSLRSTTKVS<br>TQKHRSLRSTTKVSK<br>QKHRSLRSTTKVSKS<br>KHRSLRSTTKVSKST	CPP20-376	SRGTQKHRSLRSTTK<br>VSKST
Delta-conotoxin-like S6.8<br>B2KJ30	CPP10-1221<br>CPP10-1222<br>CPP10-1223<br>CPP10-1224	RNGLKNLFPK<br>GLKNLFPKAR<br>PKARHEMKNP<br>KNPDASKLNK	CPP15-713	LKNLFPKARHEMKNP	CPP20-377<br>CPP20-378	DSRNGLKNLFPKAR<br>HEMKNP<br>LFPKARHEMKNPDA<br>SKLNKRl
Omega-conotoxin-like 6<br>Q5K0D3	CPP10-1225<br>CPP10-1226<br>CPP10-1227<br>CPP10-1228<br>CPP10-1229<br>CPP10-1230	RGVQKHRSLR<br>GVQKHRSLRS<br>VQKHRSLRST<br>KHRSLRSTTK<br>HRSLRSTTKV<br>SLRSTTKVSK	CPP15-714<br>CPP15-715<br>CPP15-716<br>CPP15-717<br>CPP15-718<br>CPP15-719	SRGVQKHRSLRSTTK<br>RGVQKHRSLRSTTKV<br>GVQKHRSLRSTTKVS<br>VQKHRSLRSTTKVSK<br>QKHRSLRSTTKVSKS<br>KHRSLRSTTKVSKST		
Omega-conotoxin-like 9<br>Q5K0D2	CPP10-1231<br>CPP10-1232<br>CPP10-1233	YGLKNLFPKA<br>GLKNLFPKAR<br>PKARHEMKNP	CPP15-720	LKNLFPKARHEMKNP	CPP20-379<br>CPP20-380	DSRYGLKNLFPKARH<br>EMKNP<br>LFPKARHEMKNPEA

	CPP10-1234<br>CPP10-1235	KNPEASKLNK<br>KLNKREGCSS				SKLNKR
Omega-conotoxin-like 2<br>Q5K0D8	CPP10-1236<br>CPP10-1237<br>CPP10-1238<br>CPP10-1239<br>CPP10-1240<br>CPP10-1241<br>CPP10-1242	SRGTQKHRSL<br>RGTQKHRSLR<br>GTQKHRSLRS<br>TQKHRSLRST<br>KHRSLRSTTK<br>HRSLRSTTKV<br>SLRSTTKVSK	CPP15-721<br>CPP15-722<br>CPP15-723<br>CPP15-724<br>CPP15-725<br>CPP15-726<br>CPP15-727	TADDSRGTQKHRSLR<br>SRGTQKHRSLRSTTK<br>RGTQKHRSLRSTTKV<br>GTQKHRSLRSTTKVS<br>TQKHRSLRSTTKVSK<br>QKHRSLRSTTKVSKA<br>KHRSLRSTTKVSKAA	CPP20-381<br>CPP20-382	DSRGTQKHRSLRSTT<br>KVSKA<br>SRGTQKHRSLRSTTK<br>VSKAA
Omega-conotoxin-like 15<br>Q5K0D5	CPP10-1243<br>CPP10-1244<br>CPP10-1245<br>CPP10-1246<br>CPP10-1247<br>CPP10-1248<br>CPP10-1249<br>CPP10-1250<br>CPP10-1251<br>CPP10-1252<br>CPP10-1253<br>CPP10-1254<br>CPP10-1255<br>CPP10-1256	RGTQKHRTLR<br>GTQKHRTLRS<br>TQKHRTLRST<br>KHRTLRSTAR<br>HRTLRSTARR<br>RTLRSTARRS<br>TLRSTARRSK<br>LRSTARRSKS<br>RSTARRSKSE<br>RRSKSELTTR<br>KSELTTRCRP<br>ELTTRCRPSG<br>CCGRCSRGKC<br>CGRCSRGKCT	CPP15-728<br>CPP15-729<br>CPP15-730<br>CPP15-731<br>CPP15-732<br>CPP15-733<br>CPP15-734<br>CPP15-735<br>CPP15-736<br>CPP15-737<br>CPP15-738<br>CPP15-739<br>CPP15-740<br>CPP15-741	TAEDSRGTQKHRTLR<br>SRGTQKHRTLRSTAR<br>RGTQKHRTLRSTARR<br>GTQKHRTLRSTARRS<br>TQKHRTLRSTARRSK<br>QKHRTLRSTARRSKS<br>KHRTLRSTARRSKSE<br>HRTLRSTARRSKSEL<br>TLRSTARRSKSELTT<br>LRSTARRSKSELTTR<br>RSTARRSKSELTTRC<br>STARRSKSELTTRCR<br>TARRSKSELTTRCRP<br>RRSKSELTTRCRPSG	CPP20-383<br>CPP20-384<br>CPP20-385<br>CPP20-386<br>CPP20-387<br>CPP20-388<br>CPP20-389<br>CPP20-390<br>CPP20-391<br>CPP20-392	DSRGTQKHRTLRSTA<br>RRSKS<br>SRGTQKHRTLRSTAR<br>RSKSE<br>RGTQKHRTLRSTARR<br>SKSEL<br>QKHRTLRSTARRSKS<br>ELTTR<br>KHRTLRSTARRSKSE<br>LTTRC<br>HRTLRSTARRSKSELT<br>TRCR<br>RTLRSTARRSKSELTT<br>RCRP<br>TLRSTARRSKSELTTR<br>CRPS<br>LRSTARRSKSELTTRC<br>RPSG<br>RSTARRSKSELTTRC<br>RPSGS
Conotoxin 3<br>Q5K0D7	CPP10-1257<br>CPP10-1258	RGTQEHRALR<br>EHRALRSDTK	CPP15-742	SRGTQEHRALRSDTK		
Conotoxin S6.11<br>P0C834	CPP10-1259<br>CPP10-1260<br>CPP10-1261<br>CPP10-1262<br>CPP10-1263<br>CPP10-1264	KLTILLLVAA<br>KAKSNFFSKR<br>AKSNFFSKRK<br>KSNFFSKRKS<br>SNFFSKRKSN<br>KCAHHRCIAW	CPP15-743<br>CPP15-744<br>CPP15-745	DERQKAKSNFFSKRK<br>QKAKSNFFSKRKSNA<br>KAKSNFFSKRKSNAE	CPP20-393<br>CPP20-394<br>CPP20-395	QGGLDERQKAKSNF<br>FSKRKS<br>GGLDERQKAKSNFF<br>SKRKSN<br>GLDERQKAKSNFFSK<br>RKSNA

Conotoxin S6.10<br>P0C833	CPP10-1265<br>CPP10-1266<br>CPP10-1267<br>CPP10-1268<br>CPP10-1269<br>CPP10-1270<br>CPP10-1271<br>CPP10-1272<br>CPP10-1273<br>CPP10-1274<br>CPP10-1275<br>CPP10-1276<br>CPP10-1277<br>CPP10-1278	AKTYSKGRQK<br>KTYSKGRQKH<br>TYSKGRQKHR<br>YSKGRQKHRA<br>SKGRQKHRAL<br>KGRQKHRALR<br>GRQKHRALRS<br>RQKHRALRST<br>KHRALRSTDK<br>DKNIKLTRRC<br>KNIKLTRRCT<br>NIKLTRRCTP<br>IKLTRRCTPD<br>KLTRRCTPDD	CPP15-746<br>CPP15-747<br>CPP15-748<br>CPP15-749<br>CPP15-750<br>CPP15-751<br>CPP15-752<br>CPP15-753<br>CPP15-754<br>CPP15-755<br>CPP15-756<br>CPP15-757<br>CPP15-758<br>CPP15-759<br>CPP15-760	QLATAKTYSKGRQKH<br>ATAKTYSKGRQKHRA<br>AKTYSKGRQKHRALR<br>KTYSKGRQKHRALRS<br>TYSKGRQKHRALRST<br>SKGRQKHRALRSTDK<br>KGRQKHRALRSTDKN<br>GRQKHRALRSTDKNI<br>RQKHRALRSTDKNIK<br>QKHRALRSTDKNIKL<br>KHRALRSTDKNIKLT<br>ALRSTDKNIKLTRRC<br>LRSTDKNIKLTRRCT<br>STDKNIKLTRRCTPD<br>KNIKLTRRCTPDDGA	CPP20-396<br>CPP20-397<br>CPP20-398<br>CPP20-399<br>CPP20-400<br>CPP20-401<br>CPP20-402<br>CPP20-403<br>CPP20-404<br>CPP20-405<br>CPP20-406<br>CPP20-407	CQLATAKTYSKGRQ<br>KHRALR<br>QLATAKTYSKGRQK<br>HRALRS<br>LATAKTYSKGRQKHR<br>ALRST<br>TAKTYSKGRQKHRAL<br>RSTDK<br>KTYSKGRQKHRALRS<br>TDKNI<br>TYSKGRQKHRALRST<br>DKNIK<br>KGRQKHRALRSTDK<br>NIKLTR<br>GRQKHRALRSTDKNI<br>KLTRR<br>RQKHRALRSTDKNIK<br>LTRRC<br>QKHRALRSTDKNIKL<br>TRRCT<br>KHRALRSTDKNIKLT<br>RRCTP<br>HRALRSTDKNIKLTR<br>RCTPD
Iota-conotoxin-<br>like S11.2<br>(Fragment)<br>P0C259	CPP10-1279<br>CPP10-1280	GCKKDRKPCS<br>CKKDRKPCSY				
conotoxin S11.3<br>P69499	CPP10-1281<br>CPP10-1282<br>CPP10-1283<br>CPP10-1284<br>CPP10-1285<br>CPP10-1286<br>CPP10-1287<br>CPP10-1288<br>CPP10-1289<br>CPP10-1290<br>CPP10-1291<br>CPP10-1292	MMFRLTSVSC<br>LTRRCVPPSR<br>TRRCVPPSRY<br>RCVPPSRYCT<br>PSRYCTRHRP<br>SRYCTRHRPC<br>RYCTRHRPCC<br>YCTRHRPCCR<br>CTRHRPCCRG<br>TRHRPCCRGT<br>HRPCCRGTCC<br>RPCCRGTCCS	CPP15-761<br>CPP15-762<br>CPP15-763<br>CPP15-764<br>CPP15-765<br>CPP15-766<br>CPP15-767<br>CPP15-768<br>CPP15-769<br>CPP15-770	QVVLTRRCVPPSRYC<br>LTRRCVPPSRYCTRH<br>TRRCVPPSRYCTRHR<br>RRCVPPSRYCTRHRP<br>RCVPPSRYCTRHRPC<br>CVPPSRYCTRHRPCC<br>VPPSRYCTRHRPCCR<br>PSRYCTRHRPCCRGT<br>SRYCTRHRPCCRGTC<br>RYCTRHRPCCRGTCC	CPP20-408<br>CPP20-409<br>CPP20-410<br>CPP20-411<br>CPP20-412<br>CPP20-413<br>CPP20-414<br>CPP20-415<br>CPP20-416	FQVVLTRRCVPPSRY<br>CTRHR<br>QVVLTRRCVPPSRYC<br>TRHRP<br>VVLTRRCVPPSRYCT<br>RHRPC<br>VLTRRCVPPSRYCTR<br>HRPCC<br>LTRRCVPPSRYCTRH<br>RPCCR<br>TRRCVPPSRYCTRHR<br>PCCRG<br>RRCVPPSRYCTRHRP<br>CCRGT

						RYCTRHRPCCRGTCC SGLCR<br>YCTRHRPCCRGTCCS GLCRP
Alpha-conotoxin-like (Fragment) D4HPH0	CPP10-1293	SCSRPSEPRR				
Conotoxin (Fragment) Q45RU7	CPP10-1294	KCGYRVSPGK				
Conotoxin S5.1 (Fragment) Q45RU8	CPP10-1295<br>CPP10-1296	CLSENRKRLT<br>ENRKRLTCSG				
Superfamily conotoxin (Fragment) D4HPG9	CPP10-1297<br>CPP10-1298	RSGRCCHPAC<br>RCCHPACGRK	CPP15-771	SDRSGRCCHPACGRK		
Superfamily conotoxin D4HPH3	CPP10-1299<br>CPP10-1300<br>CPP10-1301<br>CPP10-1302<br>CPP10-1303<br>CPP10-1304	NSCPTKPKKP<br>SCPTKPKKPG<br>CPTKPKKPGR<br>PTKPKKPGRR<br>TKPKKPGRRN<br>KPKKPGRRND	CPP15-772	NSCPTKPKKPGRRND		
A superfamily conotoxin S1.1a S4UJZ7	CPP10-1305<br>CPP10-1306	RNGCCRNPAC<br>RNPACESHRC	CPP15-773<br>CPP15-774	KHALDRNGCCRNPAC<br>RNGCCRNPACESHRC	CPP20-417	KHALDRNGCCRNPA CESHRC
A superfamily conotoxin S4.4 S4UJD9	CPP10-1307<br>CPP10-1308<br>CPP10-1309<br>CPP10-1310<br>CPP10-1311<br>CPP10-1312<br>CPP10-1313<br>CPP10-1314<br>CPP10-1315<br>CPP10-1316	CRCTNSCPKK<br>RCTNSCPKKP<br>CTNSCPKKPK<br>TNSCPKKPKK<br>NSCPKKPKKP<br>SCPKKPKKPG<br>CPKKPKKPGR<br>PKKPKKPGRR<br>KKPKKPGRRN<br>KPKKPGRRND	CPP15-775<br>CPP15-776<br>CPP15-777<br>CPP15-778<br>CPP15-779<br>CPP15-780	PCRCTNSCPKKPKKP<br>CRCTNSCPKKPKKPG<br>RCTNSCPKKPKKPGR<br>CTNSCPKKPKKPGRR<br>TNSCPKKPKKPGRRN<br>NSCPKKPKKPGRRND		
Conotoxin S4UJE7	CPP10-1317<br>CPP10-1318<br>CPP10-1319<br>CPP10-1320<br>CPP10-1321	PLSSFRGHAK<br>RGHAKSTLRR<br>GHAKSTLRRL<br>HAKSTLRRLQ<br>AKSTLRRLQD	CPP15-781<br>CPP15-782<br>CPP15-783<br>CPP15-784<br>CPP15-785	PLSSFRGHAKSTLRR<br>FRGHAKSTLRRLQDK<br>RGHAKSTLRRLQDKQ<br>GHAKSTLRRLQDKQT<br>AKSTLRRLQDKQTCC	CPP20-418	KSTLRRLQDKQTCCG YRMCI

		CPP10-1322	KSTLRRLQDK	CPP15-786	KSTLRRLQDKQTCCG		
	Conotoxin<br>S4UKA0	CPP10-1323<br>CPP10-1324<br>CPP10-1325<br>CPP10-1326<br>CPP10-1327<br>CPP10-1328<br>CPP10-1329<br>CPP10-1330<br>CPP10-1331	LRDNLKRTIR<br>RDNLKRTIRT<br>DNLKRTIRTR<br>NLKRTIRTRL<br>LKRTIRTRLN<br>KRTIRTRLNI<br>RTIRTRLNIR<br>TIRTRLNIRE<br>RTRLNIRECC	CPP15-787<br>CPP15-788<br>CPP15-789<br>CPP15-790<br>CPP15-791<br>CPP15-792<br>CPP15-793<br>CPP15-794<br>CPP15-795	LSPLRDNLKRTIRTR<br>SPLRDNLKRTIRTRL<br>PLRDNLKRTIRTRLN<br>LRDNLKRTIRTRLNI<br>RDNLKRTIRTRLNIR<br>DNLKRTIRTRLNIRE<br>NLKRTIRTRLNIREC<br>LKRTIRTRLNIRECC<br>RTIRTRLNIRECCED	CPP20-419	PLSPLRDNLKRTIRTR<br>LNIR
	Conotoxin<br>S4UJX8	CPP10-1332<br>CPP10-1333	PSVDARPKTK<br>RNLQILCCKH				
	Conotoxin<br>S4UJQ7	CPP10-1334<br>CPP10-1335<br>CPP10-1336<br>CPP10-1337<br>CPP10-1338<br>CPP10-1339<br>CPP10-1340<br>CPP10-1341<br>CPP10-1342	IQNTLQTLRK<br>QNTLQTLRKK<br>NTLQTLRKKV<br>TLQTLRKKVC<br>LQTLRKKVCC<br>QTLRKKVCCR<br>TLRKKVCCRP<br>RKKVCCRPMQ<br>KKVCCRPMQD	CPP15-796<br>CPP15-797<br>CPP15-798	DNIQNTLQTLRKKVC<br>IQNTLQTLRKKVCCR<br>TLQTLRKKVCCRPMQ		
Conus<br>kinoshitai	Mu-conotoxin<br>KIIIB (Fragment)<br>P0C195	CPP10-1343<br>CPP10-1344<br>CPP10-1345<br>CPP10-1346	SSKWCRDHSR<br>SKWCRDHSRC<br>KWCRDHSRCC<br>CRDHSRCCGR				
Conus<br>geographus	Conophysin-<br>conopressin<br>P05486	CPP10-1347<br>CPP10-1348<br>CPP10-1349<br>CPP10-1350	FIRNCPKGGK<br>IRNCPKGGKR<br>RNCPKGGKRD<br>PRICCGPRGC	CPP15-799<br>CPP15-800<br>CPP15-801	TQACFIRNCPKGGKR<br>QACFIRNCPKGGKRD<br>RDVDERYLFKACMSC	CPP20-420	LLLTTQACFIRNCPK<br>GGKRD
Conus textile	Omega-conotoxin-<br>like TxO4<br>Q9XZL1	CPP10-1351	GMIKIGPPCC				
	Alpha-conotoxin-<br>like Tx1<br>Q9XZK6	CPP10-1352<br>CPP10-1353<br>CPP10-1354<br>CPP10-1355<br>CPP10-1356<br>CPP10-1357	TSGRRAFHGR<br>SGRRAFHGRN<br>GRRAFHGRNA<br>RRAFHGRNAA<br>VSLTDRRPEC<br>RRPECCSDPR	CPP15-802<br>CPP15-803<br>CPP15-804<br>CPP15-805	SSTSGRRAFHGRNAA<br>GRRAFHGRNAAAKAS<br>VSLTDRRPECCSDPR<br>SLTDRRPECCSDPRC		

	Omega-conotoxin-like TxO6<br>Q9XZL3	CPP10-1358	GLANLFSKSR				
	Alpha-conotoxin-like Tx2<br>Q9XZK7	CPP10-1359<br>CPP10-1360<br>CPP10-1361<br>CPP10-1362<br>CPP10-1363	TSGRRTFHGR<br>SGRRTFHGRN<br>GRRTFHGRNA<br>RRTFHGRNAA<br>VSLTDRRPEC	CPP15-806<br>CPP15-807	SFTSGRRTFHGRNAA<br>GRRTFHGRNAAAKAS		
Conus tessulatus	Mu-conotoxin TsIIIA<br>H2BKS9	CPP10-1364<br>CPP10-1365<br>CPP10-1366<br>CPP10-1367<br>CPP10-1368<br>CPP10-1369<br>CPP10-1370	DQKRGCCRWP<br>QKRGCCRWPC<br>KRGCCRWPCP<br>RGCCRWPCPS<br>GCCRWPCPSR<br>CCRWPCPSRC<br>RWPCPSRCGM	CPP15-808<br>CPP15-809<br>CPP15-810<br>CPP15-811	FDQKRGCCRWPCPSR<br>QKRGCCRWPCPSRCG<br>KRGCCRWPCPSRCGM<br>GCCRWPCPSRCGMAR	CPP20-421<br>CPP20-422<br>CPP20-423	FDQKRGCCRWPCPS<br>RCGMAR<br>QKRGCCRWPCPSRC<br>GMARCC<br>KRGCCRWPCPSRCG<br>MARCCS
	Conotoxin TsMMSK-021<br>Q9BPJ9	CPP10-1371	DQHADRPADR				
	Conotoxin TsMSGL-2<br>Q9BP73	CPP10-1372<br>CPP10-1373<br>CPP10-1374<br>CPP10-1375<br>CPP10-1376<br>CPP10-1377<br>CPP10-1378<br>CPP10-1379<br>CPP10-1380<br>CPP10-1381	QRDAVNVRRR<br>RDAVNVRRRR<br>DAVNVRRRRS<br>AVNVRRRRSI<br>VN79VRRRRSIA<br>NVRRRRSIAG<br>VRRRRSIAGR<br>RRRRSIAGRT<br>RRRSIAGRTT<br>RRSIAGRTTT	CPP15-812<br>CPP15-813<br>CPP15-814<br>CPP15-815<br>CPP15-816<br>CPP15-817<br>CPP15-818<br>CPP15-819	KQATQRDAVNVRRRR<br>TQRDAVNVRRRRSIA<br>QRDAVNVRRRRSIAG<br>RDAVNVRRRRSIAGR<br>DAVNVRRRRSIAGRT<br>AVNVRRRRSIAGRTT<br>VNVRRRRSIAGRTTT<br>NVRRRRSIAGRTTTE	CPP20-424	KQATQRDAVNVRRR<br>RSIAGR
	Conotoxin TsMSGL-13<br>Q9BP75	CPP10-1382<br>CPP10-1383<br>CPP10-1384<br>CPP10-1385<br>CPP10-1386<br>CPP10-1387<br>CPP10-1388<br>CPP10-1389<br>CPP10-1390<br>CPP10-1391<br>CPP10-1392<br>CPP10-1393	QRDAINVRRR<br>RDAINVRRRR<br>DAINVRRRRS<br>AINVRRRRSI<br>INVRRRRSIT<br>NVRRRRSITR<br>VRRRRSITRR<br>RRRRSITRRG<br>RRRSITRRGD<br>RRSITRRGDE<br>RSITRRGDEE<br>CGRTNGHPRC	CPP15-820<br>CPP15-821<br>CPP15-822<br>CPP15-823<br>CPP15-824<br>CPP15-825<br>CPP15-826<br>CPP15-827<br>CPP15-828<br>CPP15-829<br>CPP15-830	KQATQRDAINVRRRR<br>QATQRDAINVRRRRS<br>ATQRDAINVRRRRSI<br>TQRDAINVRRRRSIT<br>QRDAINVRRRRSITR<br>RDAINVRRRRSITRR<br>DAINVRRRRSITRRG<br>AINVRRRRSITRRGD<br>INVRRRRSITRRGDE<br>NVRRRRSITRRGDEE<br>VRRRRSITRRGDEEC	CPP20-425<br>CPP20-426<br>CPP20-427<br>CPP20-428<br>CPP20-429	EKQATQRDAINVRR<br>RRSITR<br>KQATQRDAINVRRR<br>RSITRR<br>QATQRDAINVRRRR<br>SITRRG<br>ATQRDAINVRRRRSI<br>TRRGD<br>TQRDAINVRRRRSIT<br>RRGDE

	Conotoxin<br>TsMMSK-B022<br>Q9BPI8	CPP10-1394	RSPWFDPVRR				
	Conotoxin<br>TsMEKL-03<br>(Fragment)<br>Q9BPC0	CPP10-1395<br>CPP10-1396<br>CPP10-1397<br>CPP10-1398<br>CPP10-1399	RSNKIKALSK<br>SNKIKALSKR<br>NKIKALSKRK<br>KIKALSKRKT<br>KALSKRKTTA	CPP15-831<br>CPP15-832<br>CPP15-833<br>CPP15-834	GVEKRSNKIKALSKR<br>EKRSNKIKALSKRKT<br>KRSNKIKALSKRKTT<br>RSNKIKALSKRKTTA	CPP20-430<br>CPP20-431	ALIQSGVEKRSNKIK<br>ALSKR<br>IQSGVEKRSNKIKALS<br>KRKT
	Conotoxin<br>TsMEKL-011<br>Q9BPB9	CPP10-1400<br>CPP10-1401<br>CPP10-1402<br>CPP10-1403<br>CPP10-1404<br>CPP10-1405<br>CPP10-1406<br>CPP10-1407<br>CPP10-1408<br>CPP10-1409	KLTILLLVAA<br>ALIQRGGAKR<br>LIQRGGAKRR<br>IQRGGAKRRK<br>QRGGAKRRKV<br>RGGAKRRKVN<br>GGAKRRKVNF<br>GAKRRKVNFF<br>KRRKVNFFSI<br>RRKVNFFSIR	CPP15-835<br>CPP15-836<br>CPP15-837<br>CPP15-838<br>CPP15-839<br>CPP15-840<br>CPP15-841	MSTQALIQRGGAKRR<br>STQALIQRGGAKRRK<br>QALIQRGGAKRRKVN<br>ALIQRGGAKRRKVNF<br>RGGAKRRKVNFFSIR<br>GGAKRRKVNFFSIRE<br>KRRKVNFFSIREPGA	CPP20-432	AAVLMSTQALIQRG<br>GAKRRK
	Conotoxin<br>TsMEKL-P012<br>Q9BPC1	CPP10-1410<br>CPP10-1411<br>CPP10-1412<br>CPP10-1413<br>CPP10-1414<br>CPP10-1415<br>CPP10-1416<br>CPP10-1417	KLTILLLLAA<br>KKGGGEKRQK<br>GGGEKRQKEK<br>GGEKRQKEKI<br>EKRQKEKINF<br>KEKINFLSKR<br>EKINFLSKRK<br>KINFLSKRKT	CPP15-842<br>CPP15-843<br>CPP15-844<br>CPP15-845<br>CPP15-846	ALIKKGGGEKRQKEK<br>KKGGGEKRQKEKINF<br>EKRQKEKINFLSKRK<br>KRQKEKINFLSKRKT<br>QKEKINFLSKRKTTA	CPP20-433<br>CPP20-434<br>CPP20-435<br>CPP20-436	KKGGGEKRQKEKINF<br>LSKRK<br>KGGGEKRQKEKINFL<br>SKRKT<br>GGGEKRQKEKINFLS<br>KRKTT<br>GGEKRQKEKINFLSK<br>RKTTA
	Conotoxin Ts-011<br>Q9BH75	CPP10-1418<br>CPP10-1419<br>CPP10-1420<br>CPP10-1421<br>CPP10-1422	PSVDARPKTK<br>ANRILRTLWN<br>NRILRTLWNL<br>RILRTLWNLR<br>RTLWNLRGCC	CPP15-847<br>CPP15-848	ADNANRILRTLWNLR<br>ANRILRTLWNLRGCC		
	Conotoxin<br>TsMLCL-04<br>Q9BPD9	CPP10-1423<br>CPP10-1424<br>CPP10-1425	FIILLLLASP<br>TRIQRDLIRA<br>RIQRDLIRAA				
	Conotoxin<br>TsMLCL-03<br>Q9BPE3	CPP10-1426<br>CPP10-1427	FIILLLLASP<br>RRIQSDLIRT				
	Conotoxin<br>TsMLKM-02<br>Q9BPH4	CPP10-1428	ILSALRQRDC				

	Conotoxin<br>TsMRCL-05<br>Q9BPE5	CPP10-1429<br>CPP10-1430<br>CPP10-1431<br>CPP10-1432<br>CPP10-1433<br>CPP10-1434<br>CPP10-1435	PSVDARPNPK<br>GAVNAKRYLR<br>VNAKRYLRTL<br>NAKRYLRTLW<br>AKRYLRTLWN<br>KRYLRTLWNS<br>RYLRTLWNSR	CPP15-849<br>CPP15-850<br>CPP15-851<br>CPP15-852	GAVNAKRYLRTLWNS<br>AVNAKRYLRTLWNSR<br>AKRYLRTLWNSRDCC<br>YLRTLWNSRDCCDPK	CPP20-437<br>CPP20-438	VNAKRYLRTLWNSR<br>DCCDPK<br>KRYLRTLWNSRDCC<br>DPKEPC
	Conotoxin<br>TsMRCL-04<br>Q9BPF5	CPP10-1436<br>CPP10-1437<br>CPP10-1438<br>CPP10-1439<br>CPP10-1440<br>CPP10-1441	FIILLLLIPS<br>DNAKRTLQRH<br>AKRTLQRHWA<br>KRTLQRHWAK<br>LQRHWAKSLC<br>RHWAKSLCCP	CPP15-853<br>CPP15-854	DNAKRTLQRHWAKSL<br>KRTLQRHWAKSLCCP	CPP20-439	DNAKRTLQRHWAK<br>SLCCPED
	Conotoxin Ts5.5<br>(Fragment)<br>Q9BP46	CPP10-1442	RRIQSDLIRA				
	Conotoxin Ts-03<br>Q9BPF6	CPP10-1443<br>CPP10-1444<br>CPP10-1445<br>CPP10-1446<br>CPP10-1447<br>CPP10-1448	FIILLLLIPS<br>DNAKRTLQRH<br>AKRTLQRHWA<br>KRTLQRHWAK<br>LQRHWAKSLC<br>RHWAKSLCCP	CPP15-855<br>CPP15-856	DNAKRTLQRHWAKSL<br>KRTLQRHWAKSLCCP	CPP20-440<br>CPP20-441<br>CPP20-442	MRCLPVFIILLLLIPSA<br>ASV<br>IILLLLIPSAASVAQPK<br>TKD<br>NAKRTLQRHWAKSL<br>CCPEDA
	Conotoxin<br>TsMLCL-02<br>Q9BPD7	CPP10-1449<br>CPP10-1450	FIILLLLASP<br>RRIQSDLIRA				
	Conotoxin<br>S4UJR4	CPP10-1451	FIILLLLASP				
	Conotoxin<br>Q9BPD8	CPP10-1452<br>CPP10-1453	FIILLLLASP<br>RRIQSDLIRA				
Conus<br>abbreviatus	Conotoxin AbVIA<br>(Fragment)<br>Q9TVY1	CPP10-1454<br>CPP10-1455<br>CPP10-1456<br>CPP10-1457<br>CPP10-1458<br>CPP10-1459<br>CPP10-1460	RGEQKHRALR<br>EQKHRALRST<br>KHRALRSTDK<br>HRALRSTDKK<br>ALRSTDKKFK<br>LRSTDKKFKV<br>RSTDKKFKVA	CPP15-857<br>CPP15-858<br>CPP15-859<br>CPP15-860<br>CPP15-861<br>CPP15-862<br>CPP15-863<br>CPP15-864	TSSRGEQKHRALRST<br>SRGEQKHRALRSTDK<br>RGEQKHRALRSTDKK<br>GEQKHRALRSTDKKF<br>EQKHRALRSTDKKFK<br>QKHRALRSTDKKFKV<br>KHRALRSTDKKFKVA<br>HRALRSTDKKFKVAL	CPP20-443<br>CPP20-444<br>CPP20-445	TSSRGEQKHRALRST<br>DKKFK<br>KHRALRSTDKKFKVA<br>LLCSP<br>HRALRSTDKKFKVAL<br>LCSPP

Conotoxin AbVII (Fragment)<br>Q9TVR4	CPP10-1461<br>CPP10-1462<br>CPP10-1463<br>CPP10-1464<br>CPP10-1465<br>CPP10-1466<br>CPP10-1467<br>CPP10-1468<br>CPP10-1469<br>CPP10-1470<br>CPP10-1471<br>CPP10-1472<br>CPP10-1473	TSSRGKQKHR<br>SSRGKQKHRA<br>SRGKQKHRAL<br>RGKQKHRALR<br>GKQKHRALRS<br>KQKHRALRST<br>KHRALRSTDK<br>ALRSTDKNSR<br>DKNSRMTKRC<br>KNSRMTKRCT<br>NSRMTKRCTP<br>SRMTKRCTPA<br>RMTKRCTPAG	CPP15-865<br>CPP15-866<br>CPP15-867<br>CPP15-868<br>CPP15-869<br>CPP15-870<br>CPP15-871<br>CPP15-872<br>CPP15-873<br>CPP15-874<br>CPP15-875<br>CPP15-876<br>CPP15-877	QLTTAETSSRGKQKH<br>AETSSRGKQKHRALR<br>TSSRGKQKHRALRST<br>SRGKQKHRALRSTDK<br>RGKQKHRALRSTDKN<br>GKQKHRALRSTDKNS<br>KQKHRALRSTDKNSR<br>KHRALRSTDKNSRMT<br>ALRSTDKNSRMTKRC<br>LRSTDKNSRMTKRCT<br>RSTDKNSRMTKRCTP<br>STDKNSRMTKRCTPA<br>NSRMTKRCTPAGDAC	CPP20-446<br>CPP20-447<br>CPP20-448<br>CPP20-449<br>CPP20-450<br>CPP20-451<br>CPP20-452	QLTTAETSSRGKQKHRALRS<br>TAETSSRGKQKHRALRSTDK<br>GKQKHRALRSTDKNSRMTKR<br>KQKHRALRSTDKNSRMTKRC<br>QKHRALRSTDKNSRMTKRCT<br>KHRALRSTDKNSRMTKRCTP<br>HRALRSTDKNSRMTKRCTPA
Conotoxin AbVID (Fragment)<br>Q9UA91	CPP10-1474<br>CPP10-1475<br>CPP10-1476<br>CPP10-1477<br>CPP10-1478<br>CPP10-1479<br>CPP10-1480<br>CPP10-1481<br>CPP10-1482	ALRSTDKNSR<br>DKNSRMTKRC<br>KNSRMTKRCT<br>NSRMTKRCTP<br>SRMTKRCTPR<br>RMTKRCTPRH<br>MTKRCTPRHG<br>TKRCTPRHGV<br>KRCTPRHGVC	CPP15-878<br>CPP15-879<br>CPP15-880<br>CPP15-881<br>CPP15-882<br>CPP15-883<br>CPP15-884<br>CPP15-885	ALRSTDKNSRMTKRC<br>LRSTDKNSRMTKRCT<br>RSTDKNSRMTKRCTP<br>STDKNSRMTKRCTPR<br>TDKNSRMTKRCTPRH<br>DKNSRMTKRCTPRHG<br>SRMTKRCTPRHGVCF<br>RMTKRCTPRHGVCFY	CPP20-453<br>CPP20-454<br>CPP20-455<br>CPP20-456	QEHLALRSTDKNSRMTKRCT<br>HLALRSTDKNSRMTKRCTPR<br>ALRSTDKNSRMTKRCTPRHG<br>KRCTPRHGVCFYSYFCCSKA
Conotoxin AbVIH (Fragment)<br>Q9UA95	CPP10-1483<br>CPP10-1484<br>CPP10-1485<br>CPP10-1486<br>CPP10-1487<br>CPP10-1488<br>CPP10-1489<br>CPP10-1490<br>CPP10-1491<br>CPP10-1492<br>CPP10-1493<br>CPP10-1494	TSSRGKQKHR<br>SSRGKQKHRA<br>SRGKQKHRAL<br>RGKQKHRALR<br>GKQKHRALRS<br>KQKHRALRST<br>KHRALRSTDK<br>ALRSTDKDSR<br>KDSRMTKRCT<br>DSRMTKRCTP<br>SRMTKRCTPA<br>RMTKRCTPAG	CPP15-886<br>CPP15-887<br>CPP15-888<br>CPP15-889<br>CPP15-890<br>CPP15-891<br>CPP15-892<br>CPP15-893<br>CPP15-894<br>CPP15-895	QLTTAETSSRGKQKH<br>AETSSRGKQKHRALR<br>TSSRGKQKHRALRST<br>SRGKQKHRALRSTDK<br>RGKQKHRALRSTDKD<br>GKQKHRALRSTDKDS<br>KQKHRALRSTDKDSR<br>KHRALRSTDKDSRMT<br>ALRSTDKDSRMTKRC<br>STDKDSRMTKRCTPA	CPP20-457<br>CPP20-458<br>CPP20-459<br>CPP20-460<br>CPP20-461<br>CPP20-462<br>CPP20-463	QLTTAETSSRGKQKHRALRS<br>TAETSSRGKQKHRALRSTDK<br>GKQKHRALRSTDKDSRMTKR<br>KQKHRALRSTDKDSRMTKRC<br>QKHRALRSTDKDSRMTKRCT<br>KHRALRSTDKDSRMTKRCTP<br>HRALRSTDKDSRMTKRCTPA
Conotoxin AbVIO (Fragment)<br>Q9TVQ7	CPP10-1495<br>CPP10-1496<br>CPP10-1497<br>CPP10-1498<br>CPP10-1499	ASYARSERKH<br>SYARSERKHP<br>YARSERKHPD<br>RSERKHPDLR<br>KHPDLRLSSR	CPP15-896<br>CPP15-897<br>CPP15-898<br>CPP15-899<br>CPP15-900	LIATASYARSERKHP<br>TASYARSERKHPDLR<br>KHPDLRLSSRNSKLS<br>DLRLSSRNSKLSKRC<br>LRLSSRNSKLSKRCL	CPP20-464<br>CPP20-465<br>CPP20-466<br>CPP20-467<br>CPP20-468	RSERKHPDLRLSSRNSKLSK<br>SERKHPDLRLSSRNSKLSKR<br>ERKHPDLRLSSRNSK

	CPP10-1500<br>CPP10-1501<br>CPP10-1502<br>CPP10-1503<br>CPP10-1504	DLRLSSRNSK<br>SSRNSKLSKR<br>SRNSKLSKRC<br>RNSKLSKRCL<br>KLSKRCLGSR	CPP15-901<br>CPP15-902<br>CPP15-903	RLSSRNSKLSKRCLG<br>SSRNSKLSKRCLGSR<br>KLSKRCLGSRELCVR	CPP20-469	LSKRC<br>RKHPDLRLSSRNSKL<br>SKRCL<br>KHPDLRLSSRNSKLS<br>KRCLG<br>KLSKRCLGSRELCVR<br>DTSCC
Conotoxin AbVIG (Fragment) Q9TVX4	CPP10-1505<br>CPP10-1506<br>CPP10-1507<br>CPP10-1508<br>CPP10-1509<br>CPP10-1510<br>CPP10-1511<br>CPP10-1512<br>CPP10-1513<br>CPP10-1514<br>CPP10-1515<br>CPP10-1516	TSSRGKQKHR<br>SSRGKQKHRA<br>SRGKQKHRAL<br>RGKQKHRALR<br>GKQKHRALRS<br>KQKHRALRST<br>KHRALRSTDK<br>ALRSTDKNSR<br>DKNSRMTKRC<br>KNSRMTKRCT<br>NSRMTKRCTA<br>SRMTKRCTAP	CPP15-904<br>CPP15-905<br>CPP15-906<br>CPP15-907<br>CPP15-908<br>CPP15-909<br>CPP15-910<br>CPP15-911<br>CPP15-912<br>CPP15-913<br>CPP15-914	QLTTAETSSRGKQKH<br>AETSSRGKQKHRALR<br>TSSRGKQKHRALRST<br>SRGKQKHRALRSTDK<br>RGKQKHRALRSTDKN<br>GKQKHRALRSTDKNS<br>KQKHRALRSTDKNSR<br>KHRALRSTDKNSRMT<br>ALRSTDKNSRMTKRC<br>LRSTDKNSRMTKRCT<br>STDKNSRMTKRCTAP	CPP20-470<br>CPP20-471<br>CPP20-472<br>CPP20-473<br>CPP20-474<br>CPP20-475<br>CPP20-476<br>CPP20-477	QLTTAETSSRGKQKH<br>RALRS<br>TAETSSRGKQKHRAL<br>RSTDK<br>GKQKHRALRSTDKN<br>SRMTKR<br>KQKHRALRSTDKNS<br>RMTKRC<br>QKHRALRSTDKNSR<br>MTKRCT<br>KHRALRSTDKNSRM<br>TKRCTA<br>HRALRSTDKNSRMT<br>KRCTAP<br>ALRSTDKNSRMTKR<br>CTAPGG
Conotoxin AbVIN (Fragment) Q9TVQ6	CPP10-1517<br>CPP10-1518<br>CPP10-1519<br>CPP10-1520<br>CPP10-1521<br>CPP10-1522<br>CPP10-1523<br>CPP10-1524<br>CPP10-1525<br>CPP10-1526	ASYARSERKH<br>SYARSERKHP<br>YARSERKHPD<br>RSERKHPDLR<br>KHPDLRLSSR<br>DLRLSSRNSK<br>SSRNSKLSKR<br>SRNSKLSKRC<br>RNSKLSKRCL<br>KLSKRCLGSR	CPP15-915<br>CPP15-916<br>CPP15-917<br>CPP15-918<br>CPP15-919<br>CPP15-920<br>CPP15-921<br>CPP15-922	LIATASYARSERKHP<br>TASYARSERKHPDLR<br>KHPDLRLSSRNSKLS<br>DLRLSSRNSKLSKRC<br>LRLSSRNSKLSKRCL<br>RLSSRNSKLSKRCLG<br>SSRNSKLSKRCLGSR<br>KLSKRCLGSREQCVR	CPP20-478<br>CPP20-479<br>CPP20-480<br>CPP20-481<br>CPP20-482<br>CPP20-483	RSERKHPDLRLSSRN<br>SKLSK<br>SERKHPDLRLSSRNS<br>KLSKR<br>ERKHPDLRLSSRNSK<br>LSKRC<br>RKHPDLRLSSRNSKL<br>SKRCL<br>KHPDLRLSSRNSKLS<br>KRCLG<br>KLSKRCLGSREQCVR<br>DTSCC
Conotoxin AbVIE (Fragment) Q9UA85	CPP10-1527<br>CPP10-1528<br>CPP10-1529<br>CPP10-1530<br>CPP10-1531<br>CPP10-1532<br>CPP10-1533	TSSRGKQKHR<br>SSRGKQKHRA<br>SRGKQKHRAL<br>RGKQKHRALR<br>GKQKHRALRS<br>KQKHRALRST<br>KHRALRSTDK	CPP15-923<br>CPP15-924<br>CPP15-925<br>CPP15-926<br>CPP15-927<br>CPP15-928<br>CPP15-929	QLTTAETSSRGKQKH<br>AETSSRGKQKHRALR<br>TSSRGKQKHRALRST<br>SRGKQKHRALRSTDK<br>RGKQKHRALRSTDKY<br>GKQKHRALRSTDKYS<br>KQKHRALRSTDKYSR	CPP20-484<br>CPP20-485<br>CPP20-486<br>CPP20-487<br>CPP20-488<br>CPP20-489<br>CPP20-490	QLTTAETSSRGKQKH<br>RALRS<br>TAETSSRGKQKHRAL<br>RSTDK<br>SSRGKQKHRALRSTD<br>KYSRM<br>SRGKQKHRALRSTD

	CPP10-1534<br>CPP10-1535<br>CPP10-1536<br>CPP10-1537<br>CPP10-1538<br>CPP10-1539	ALRSTDKYSR<br>SRMTKHCTPP<br>CCSKICWRPR<br>CSKICWRPRC<br>SKICWRPRCY<br>KICWRPRCYP	CPP15-930<br>CPP15-931<br>CPP15-932	QKHRALRSTDKYSRM<br>KHRALRSTDKYSRMT<br>ALRSTDKYSRMTKHC	CPP20-491<br>CPP20-492<br>CPP20-493	KYSRMT<br>RGKQKHRALRSTDK<br>YSRMTK<br>GKQKHRALRSTDKYS<br>RMTKH<br>KQKHRALRSTDKYSR<br>MTKHC<br>QKHRALRSTDKYSR<br>MTKHCT<br>KHRALRSTDKYSRM<br>TKHCTP<br>HRALRSTDKYSRMT<br>KHCTPP
Conotoxin AbVIM<br>(Fragment)<br>Q9UA72	CPP10-1540<br>CPP10-1541<br>CPP10-1542<br>CPP10-1543<br>CPP10-1544<br>CPP10-1545<br>CPP10-1546<br>CPP10-1547<br>CPP10-1548<br>CPP10-1549	ASYARSERKH<br>SYARSERKHP<br>YARSERKHPD<br>RSERKHPDLR<br>KHPDLRLSSR<br>DLRLSSRNSK<br>SSRNSKLSKR<br>SRNSKLSKRC<br>RNSKLSKRCL<br>KLSKRCLGSG	CPP15-933<br>CPP15-934<br>CPP15-935<br>CPP15-936<br>CPP15-937<br>CPP15-938	LIATASYARSERKHP<br>TASYARSERKHPDLR<br>KHPDLRLSSRNSKLS<br>DLRLSSRNSKLSKRC<br>LRLSSRNSKLSKRCL<br>RLSSRNSKLSKRCLG	CPP20-494<br>CPP20-495<br>CPP20-496<br>CPP20-497<br>CPP20-498	RSERKHPDLRLSSRN<br>SKLSK<br>SERKHPDLRLSSRNS<br>KLSKR<br>ERKHPDLRLSSRNSK<br>LSKRC<br>RKHPDLRLSSRNSKL<br>SKRCL<br>KHPDLRLSSRNSKLS<br>KRCLG
Conotoxin AbVIB<br>(Fragment)<br>Q9UA83	CPP10-1550<br>CPP10-1551<br>CPP10-1552<br>CPP10-1553<br>CPP10-1554<br>CPP10-1555<br>CPP10-1556<br>CPP10-1557<br>CPP10-1558<br>CPP10-1559	TSSRGKQKHR<br>SSRGKQKHRA<br>SRGKQKHRAL<br>RGKQKHRALR<br>GKQKHRALRS<br>KQKHRALRST<br>KHRALRSTDK<br>ALRSTDKNSK<br>KNSKLTRGCT<br>KLTRGCTPPG	CPP15-939<br>CPP15-940<br>CPP15-941<br>CPP15-942<br>CPP15-943<br>CPP15-944<br>CPP15-945<br>CPP15-946<br>CPP15-947	QLTTAETSSRGKQKH<br>AETSSRGKQKHRALR<br>TSSRGKQKHRALRST<br>SRGKQKHRALRSTDK<br>RGKQKHRALRSTDKN<br>GKQKHRALRSTDKNS<br>KQKHRALRSTDKNSK<br>KHRALRSTDKNSKLT<br>ALRSTDKNSKLTRGC	CPP20-499<br>CPP20-500<br>CPP20-501<br>CPP20-502<br>CPP20-503<br>CPP20-504<br>CPP20-505<br>CPP20-506	QLTTAETSSRGKQKH<br>RALRS<br>TAETSSRGKQKHRAL<br>RSTDK<br>SSRGKQKHRALRSTD<br>KNSKL<br>GKQKHRALRSTDKN<br>SKLTRG<br>KQKHRALRSTDKNS<br>KLTRGC<br>QKHRALRSTDKNSKL<br>TRGCT<br>KHRALRSTDKNSKLT<br>RGCTP<br>HRALRSTDKNSKLTR<br>GCTPP
Conotoxin AbVIF<br>(Fragment)<br>Q9TVK3	CPP10-1560<br>CPP10-1561<br>CPP10-1562	TSSRGKQKHR<br>SSRGKQKHRA<br>SRGKQKHRAL	CPP15-948<br>CPP15-949<br>CPP15-950	QLTTAETSSRGKQKH<br>AETSSRGKQKHRALR<br>TSSRGKQKHRALRST	CPP20-507<br>CPP20-508<br>CPP20-509	QLTTAETSSRGKQKH<br>RALRS<br>TAETSSRGKQKHRAL

	CPP10-1563<br>CPP10-1564<br>CPP10-1565<br>CPP10-1566<br>CPP10-1567<br>CPP10-1568<br>CPP10-1569<br>CPP10-1570<br>CPP10-1571<br>CPP10-1572<br>CPP10-1573<br>CPP10-1574	RGKQKHRALR<br>GKQKHRALRS<br>KQKHRALRST<br>KHRALRSTDK<br>ALRSTDKNSR<br>DKNSRMSKRC<br>KNSRMSKRCT<br>NSRMSKRCTP<br>SRMSKRCTPP<br>RMSKRCTPPG<br>CSQVCNFPRK<br>SQVCNFPRKH	CPP15-951<br>CPP15-952<br>CPP15-953<br>CPP15-954<br>CPP15-955<br>CPP15-956<br>CPP15-957<br>CPP15-958<br>CPP15-959	SRGKQKHRALRSTDK<br>RGKQKHRALRSTDKN<br>GKQKHRALRSTDKNS<br>KQKHRALRSTDKNSR<br>KHRALRSTDKNSRMS<br>ALRSTDKNSRMSKRC<br>LRSTDKNSRMSKRCT<br>STDKNSRMSKRCTPP<br>TDKNSRMSKRCTPPG	CPP20-510<br>CPP20-511<br>CPP20-512<br>CPP20-513<br>CPP20-514<br>CPP20-515<br>CPP20-516	RSTDK<br>RGKQKHRALRSTDK<br>NSRMSK<br>GKQKHRALRSTDKN<br>SRMSKR<br>KQKHRALRSTDKNS<br>RMSKRC<br>QKHRALRSTDKNSR<br>MSKRCT<br>KHRALRSTDKNSRM<br>SKRCTP<br>HRALRSTDKNSRMS<br>KRCTPP<br>RALRSTDKNSRMSK<br>RCTPPG<br>ALRSTDKNSRMSKR<br>CTPPGG
Conotoxin AbVIC<br>(Fragment)<br>Q9TVU1	CPP10-1575<br>CPP10-1576<br>CPP10-1577<br>CPP10-1578<br>CPP10-1579<br>CPP10-1580<br>CPP10-1581<br>CPP10-1582<br>CPP10-1583<br>CPP10-1584	TSSRGKQKHR<br>SSRGKQKHRA<br>SRGKQKHRAL<br>RGKQKHRALR<br>GKQKHRALRS<br>KQKHRALRST<br>KHRALRSTDK<br>ALRSTDKNSK<br>KNSKLTRGCT<br>KLTRGCTPPG	CPP15-960<br>CPP15-961<br>CPP15-962<br>CPP15-963<br>CPP15-964<br>CPP15-965<br>CPP15-966<br>CPP15-967<br>CPP15-968	QLTTAETSSRGKQKH<br>AETSSRGKQKHRALR<br>TSSRGKQKHRALRST<br>SRGKQKHRALRSTDK<br>RGKQKHRALRSTDKN<br>GKQKHRALRSTDKNS<br>KQKHRALRSTDKNSK<br>KHRALRSTDKNSKLT<br>ALRSTDKNSKLTRGC	CPP20-517<br>CPP20-518<br>CPP20-519<br>CPP20-520<br>CPP20-521<br>CPP20-522<br>CPP20-523<br>CPP20-524	QLTTAETSSRGKQKH<br>RALRS<br>TAETSSRGKQKHRAL<br>RSTDK<br>SSRGKQKHRALRSTD<br>KNSKL<br>GKQKHRALRSTDKN<br>SKLTRG<br>KQKHRALRSTDKNS<br>KLTRGC<br>QKHRALRSTDKNSKL<br>TRGCT<br>KHRALRSTDKNSKLT<br>RGCTP<br>HRALRSTDKNSKLTR<br>GCTPP
Conotoxin AbVIK<br>(Fragment)<br>Q9UA71	CPP10-1585<br>CPP10-1586<br>CPP10-1587<br>CPP10-1588<br>CPP10-1589<br>CPP10-1590<br>CPP10-1591<br>CPP10-1592<br>CPP10-1593	TSSRGKQKHR<br>SSRGKQKHRA<br>SRGKQKHRAL<br>RGKQKHRALR<br>GKQKHRALRS<br>KQKHRALRST<br>KHRALRSTDK<br>ALRSTDKNSR<br>DKNSRMTKRC	CPP15-969<br>CPP15-970<br>CPP15-971<br>CPP15-972<br>CPP15-973<br>CPP15-974<br>CPP15-975<br>CPP15-976<br>CPP15-977	QLTTAETSSRGKQKH<br>AETSSRGKQKHRALR<br>TSSRGKQKHRALRST<br>SRGKQKHRALRSTDK<br>RGKQKHRALRSTDKN<br>GKQKHRALRSTDKNS<br>KQKHRALRSTDKNSR<br>KHRALRSTDKNSRMT<br>ALRSTDKNSRMTKRC	CPP20-525<br>CPP20-526<br>CPP20-527<br>CPP20-528<br>CPP20-529<br>CPP20-530<br>CPP20-531	QLTTAETSSRGKQKH<br>RALRS<br>TAETSSRGKQKHRAL<br>RSTDK<br>GKQKHRALRSTDKN<br>SRMTKR<br>KQKHRALRSTDKNS<br>RMTKRC<br>QKHRALRSTDKNSR

		CPP10-1594<br>CPP10-1595<br>CPP10-1596<br>CPP10-1597	KNSRMTKRCT<br>NSRMTKRCTP<br>SRMTKRCTPA<br>RMTKRCTPAG	CPP15-978<br>CPP15-979<br>CPP15-980	LRSTDKNSRMTKRCT<br>RSTDKNSRMTKRCTP<br>STDKNSRMTKRCTPA		MTKRCT<br>KHRALRSTDKNSRMTKRCTP<br>HRALRSTDKNSRMTKRCTPA
	Conotoxin AbVIJ (Fragment) Q9UA93	CPP10-1598<br>CPP10-1599<br>CPP10-1600<br>CPP10-1601<br>CPP10-1602<br>CPP10-1603<br>CPP10-1604<br>CPP10-1605<br>CPP10-1606<br>CPP10-1607<br>CPP10-1608<br>CPP10-1609<br>CPP10-1610	TSSRGKQKHR<br>SSRGKQKHRA<br>SRGKQKHRAL<br>RGKQKHRALR<br>GKQKHRALRS<br>KQKHRALRST<br>KHRALRSTDK<br>ALRSTDKNSR<br>DKNSRMTKRC<br>KNSRMTKRCT<br>NSRMTKRCTP<br>SRMTKRCTPA<br>RMTKRCTPAG	CPP15-981<br>CPP15-982<br>CPP15-983<br>CPP15-984<br>CPP15-985<br>CPP15-986<br>CPP15-987<br>CPP15-988<br>CPP15-989<br>CPP15-990<br>CPP15-991<br>CPP15-992<br>CPP15-993	QLTTAETSSRGKQKH<br>AETSSRGKQKHRALR<br>TSSRGKQKHRALRST<br>SRGKQKHRALRSTDK<br>RGKQKHRALRSTDKN<br>GKQKHRALRSTDKNS<br>KQKHRALRSTDKNSR<br>KHRALRSTDKNSRMT<br>ALRSTDKNSRMTKRC<br>LRSTDKNSRMTKRCT<br>RSTDKNSRMTKRCTP<br>STDKNSRMTKRCTPA<br>NSRMTKRCTPAGDAC	CPP20-532<br>CPP20-533<br>CPP20-534<br>CPP20-535<br>CPP20-536<br>CPP20-537<br>CPP20-538	QLTTAETSSRGKQKHRALRS<br>TAETSSRGKQKHRALRSTDK<br>GKQKHRALRSTDKNSRMTKR<br>KQKHRALRSTDKNSRMTKRC<br>QKHRALRSTDKNSRMTKRCT<br>KHRALRSTDKNSRMTKRCTP<br>HRALRSTDKNSRMTKRCTPA
	Conotoxin AbVIL (Fragment) Q9UA86	CPP10-1611<br>CPP10-1612<br>CPP10-1613<br>CPP10-1614<br>CPP10-1615<br>CPP10-1616<br>CPP10-1617<br>CPP10-1618<br>CPP10-1619<br>CPP10-1620<br>CPP10-1621<br>CPP10-1622	RGEQKHRAPR<br>GEQKHRAPRS<br>EQKHRAPRST<br>KHRAPRSTDK<br>HRAPRSTDKN<br>APRSTDKNSR<br>DKNSRMTKRC<br>KNSRMTKRCT<br>NSRMTKRCTP<br>SRMTKRCTPG<br>RMTKRCTPGG<br>NLATNKCRSP	CPP15-994<br>CPP15-995<br>CPP15-996<br>CPP15-997<br>CPP15-998<br>CPP15-999<br>CPP15-1000<br>CPP15-1001<br>CPP15-1002<br>CPP15-1003<br>CPP15-1004	TSSRGEQKHRAPRST<br>SRGEQKHRAPRSTDK<br>RGEQKHRAPRSTDKN<br>GEQKHRAPRSTDKNS<br>EQKHRAPRSTDKNSR<br>QKHRAPRSTDKNSRM<br>KHRAPRSTDKNSRMT<br>APRSTDKNSRMTKRC<br>PRSTDKNSRMTKRCT<br>RSTDKNSRMTKRCTP<br>STDKNSRMTKRCTPG	CPP20-539<br>CPP20-540<br>CPP20-541<br>CPP20-542<br>CPP20-543	QLTTAETSSRGEQKHRAPRS<br>EQKHRAPRSTDKNSRMTKRC<br>QKHRAPRSTDKNSRMTKRCT<br>KHRAPRSTDKNSRMTKRCTP<br>HRAPRSTDKNSRMTKRCTPG
	Four-loop conotoxin ABVIA (Fragment) Q9UA79	CPP10-1623<br>CPP10-1624<br>CPP10-1625<br>CPP10-1626<br>CPP10-1627<br>CPP10-1628<br>CPP10-1629	RGEQKHRALR<br>EQKHRALRST<br>KHRALRSTDK<br>HRALRSTDKK<br>ALRSTDKKFK<br>LRSTDKKFKV<br>RSTDKKFKVA	CPP15-1005<br>CPP15-1006<br>CPP15-1007<br>CPP15-1008<br>CPP15-1009<br>CPP15-1010<br>CPP15-1011<br>CPP15-1012	TSSRGEQKHRALRST<br>SRGEQKHRALRSTDK<br>RGEQKHRALRSTDKK<br>GEQKHRALRSTDKKF<br>EQKHRALRSTDKKFK<br>QKHRALRSTDKKFKV<br>KHRALRSTDKKFKVA<br>HRALRSTDKKFKVAL	CPP20-544<br>CPP20-545<br>CPP20-546	TSSRGEQKHRALRSTDKKFK<br>KHRALRSTDKKFKVALLCSP<br>HRALRSTDKKFKVALLCSPP
Conus pennaceus	Gamma-conotoxin PnVIIA P56711	CPP10-1630<br>CPP10-1631<br>CPP10-1632	KLTILLLVAA<br>AKINFLSKRK<br>KINFLSKRKP	CPP15-1013<br>CPP15-1014<br>CPP15-1015	EQRQQAKINFLSKRK<br>QRQQAKINFLSKRKP<br>QQAKINFLSKRKPSA	CPP20-547<br>CPP20-548<br>CPP20-549	AQNQEQRQQAKINFLSKRKP<br>NQEQRQQAKINFLS

		CPP10-1633<br>CPP10-1634<br>CPP10-1635<br>CPP10-1636<br>CPP10-1637<br>CPP10-1638<br>CPP10-1639<br>CPP10-1640<br>CPP10-1641<br>CPP10-1642	NFLSKRKPSA<br>FLSKRKPSAE<br>KRKPSAERWR<br>RKPSAERWRR<br>KPSAERWRRD<br>PSAERWRRDC<br>SAERWRRDCT<br>ERWRRDCTSW<br>RWRRDCTSWF<br>RRDCTSWFGR	CPP15-1016<br>CPP15-1017<br>CPP15-1018<br>CPP15-1019<br>CPP15-1020<br>CPP15-1021<br>CPP15-1022	KINFLSKRKPSAERW<br>INFLSKRKPSAERWR<br>NFLSKRKPSAERWRR<br>FLSKRKPSAERWRRD<br>SKRKPSAERWRRDCT<br>RKPSAERWRRDCTSW<br>KPSAERWRRDCTSWF	CPP20-550<br>CPP20-551<br>CPP20-552<br>CPP20-553<br>CPP20-554<br>CPP20-555	KRKPSA<br>RQQAKINFLSKRKPS<br>AERWR<br>QQAKINFLSKRKPSA<br>ERWRR<br>QAKINFLSKRKPSAE<br>RWRRD<br>AKINFLSKRKPSAER<br>WRRDC<br>KINFLSKRKPSAERW<br>RRDCT<br>FLSKRKPSAERWRR<br>DCTSWF<br>RKPSAERWRRDCTS<br>WFGRCT
	Mu-conotoxin<br>PnIVB<br>P58927	CPP10-1643	LIICLLLCPL				
	Omega-conotoxin<br>PnVIB<br>P56713	CPP10-1644	GMIKIGPPCC				
	Mu-conotoxin<br>pn4c (Fragment)<br>Q9BP55	CPP10-1645	KRCCKYGWTC				
	Alpha-conotoxin-<br>like PnMGMR-02<br>Q9BP56	CPP10-1646	KGCCSRPPCA				
	Mu-conotoxin-like<br>PnMKLT1-014<br>Q9U657	CPP10-1647<br>CPP10-1648<br>CPP10-1649<br>CPP10-1650<br>CPP10-1651<br>CPP10-1652<br>CPP10-1653<br>CPP10-1654<br>CPP10-1655	GLANLFSKSR<br>PEPSKLEKRK<br>EPSKLEKRKT<br>SKLEKRKTCQ<br>KLEKRKTCQR<br>LEKRKTCQRR<br>EKRKTCQRRW<br>KRKTCQRRWD<br>RKTCQRRWDF	CPP15-1023<br>CPP15-1024<br>CPP15-1025	EPSKLEKRKTCQRRW<br>PSKLEKRKTCQRRWD<br>SKLEKRKTCQRRWDF	CPP20-556	FSKSRYEMEDPEPSK<br>LEKRK
	Conotoxin<br>PnMSGL-03<br>Q9BP63	CPP10-1656<br>CPP10-1657<br>CPP10-1658<br>CPP10-1659<br>CPP10-1660	QRDAINFRWR<br>RDAINFRWRR<br>DAINFRWRRS<br>AINFRWRRSL<br>INFRWRRSLI	CPP15-1026<br>CPP15-1027<br>CPP15-1028<br>CPP15-1029<br>CPP15-1030	KQATQRDAINFRWRR<br>TQRDAINFRWRRSLI<br>QRDAINFRWRRSLIR<br>RDAINFRWRRSLIRR<br>DAINFRWRRSLIRRT	CPP20-557<br>CPP20-558<br>CPP20-559<br>CPP20-560<br>CPP20-561	KQATQRDAINFRWR<br>RSLIRR<br>QATQRDAINFRWRR<br>SLIRRT<br>ATQRDAINFRWRRS

	CPP10-1661<br>CPP10-1662<br>CPP10-1663<br>CPP10-1664<br>CPP10-1665	NFRWRRSLIR<br>FRWRRSLIRR<br>RWRRSLIRRT<br>WRRSLIRRTA<br>RRSLIRRTAT	CPP15-1031<br>CPP15-1032<br>CPP15-1033<br>CPP15-1034<br>CPP15-1035	AINFRWRRSLIRRTA<br>INFRWRRSLIRRTAT<br>NFRWRRSLIRRTATE<br>FRWRRSLIRRTATEE<br>RWRRSLIRRTATEEC		LIRRTA<br>TQRDAINFRWRRSLIRRTAT<br>RDAINFRWRRSLIRRTATEE
Conotoxin PnMSGL-01 Q9BP64	CPP10-1666<br>CPP10-1667<br>CPP10-1668<br>CPP10-1669<br>CPP10-1670<br>CPP10-1671<br>CPP10-1672<br>CPP10-1673<br>CPP10-1674<br>CPP10-1675<br>CPP10-1676	QRDAINVRRR<br>RDAINVRRRR<br>DAINVRRRRS<br>AINVRRRRSI<br>INVRRRRSIT<br>NVRRRRSITR<br>VRRRRSITRR<br>RRRRSITRRV<br>RRRSITRRVS<br>RRSITRRVSE<br>RSITRRVSEA	CPP15-1036<br>CPP15-1037<br>CPP15-1038<br>CPP15-1039<br>CPP15-1040<br>CPP15-1041<br>CPP15-1042<br>CPP15-1043<br>CPP15-1044<br>CPP15-1045<br>CPP15-1046	KQAMQRDAINVRRRR<br>QAMQRDAINVRRRRS<br>AMQRDAINVRRRRSI<br>MQRDAINVRRRRSIT<br>QRDAINVRRRRSITR<br>RDAINVRRRRSITRR<br>DAINVRRRRSITRRV<br>AINVRRRRSITRRVS<br>INVRRRRSITRRVSE<br>NVRRRRSITRRVSEA<br>VRRRRSITRRVSEAC	CPP20-562<br>CPP20-563<br>CPP20-564<br>CPP20-565<br>CPP20-566<br>CPP20-567	EKQAMQRDAINVRRRRSITR<br>KQAMQRDAINVRRRRSITRR<br>QAMQRDAINVRRRRSITRRV<br>MQRDAINVRRRRSITRRVSE<br>QRDAINVRRRRSITRRVSEA<br>RDAINVRRRRSITRRVSEAC
Conotoxin PnMKLT1-0121 Q9U660	CPP10-1677<br>CPP10-1678	FATADDPRNR<br>KNPEASKLNK				
Conotoxin PnMKLT1-1111 Q9U662	CPP10-1679<br>CPP10-1680<br>CPP10-1681	KRLANLYLKA<br>RLANLYLKAR<br>SWVACESPKR	CPP15-1047<br>CPP15-1048	VPHSNKRLANLYLKA<br>PHSNKRLANLYLKAR	CPP20-568	HSNKRLANLYLKARHEMKNP
Conotoxin PnMEKL-04 Q9BPA6	CPP10-1682<br>CPP10-1683<br>CPP10-1684<br>CPP10-1685<br>CPP10-1686<br>CPP10-1687<br>CPP10-1688	KLTILLLVAA<br>KENIKFLLKR<br>ENIKFLLKRK<br>NIKFLLKRKT<br>IKFLLKRKTA<br>KFLLKRKTAA<br>KRKTAADRGM	CPP15-1049<br>CPP15-1050<br>CPP15-1051<br>CPP15-1052<br>CPP15-1053	ENRLKENIKFLLKRK<br>NRLKENIKFLLKRKT<br>RLKENIKFLLKRKTA<br>LKENIKFLLKRKTAA<br>ENIKFLLKRKTAADR	CPP20-569<br>CPP20-570<br>CPP20-571	QGGGENRLKENIKFLLKRKT<br>GGGENRLKENIKFLLKRKTA<br>GGENRLKENIKFLLKRKTAA
Conotoxin Pn-014 Q9BH86	CPP10-1689<br>CPP10-1690	PSVDARPKTK<br>KRILQTFESR				
Conotoxin PnMRCL-0111 Q9BPF8	CPP10-1691	RTVRTLLDIR				
Chi-conotoxin-like PnMRCL-013 Q9BPE9	CPP10-1692	KSTLQRLQDK				

Conotoxin PnMLKM-013 Q9BPH5	CPP10-1693	KREMILPALR				
Conotoxin PnMRCL-0111 Q9BPF4	CPP10-1694	RTVRTLLDIR				
Conotoxin Pn-B02 Q9BPG8	CPP10-1695<br>CPP10-1696<br>CPP10-1697<br>CPP10-1698<br>CPP10-1699<br>CPP10-1700<br>CPP10-1701<br>CPP10-1702	FHDNLKRTRR<br>HDNLKRTRRI<br>DNLKRTRRIH<br>NLKRTRRIHL<br>LKRTRRIHLN<br>KRTRRIHLNI<br>RTRRIHLNIR<br>RRIHLNIREC	CPP15-1054<br>CPP15-1055<br>CPP15-1056<br>CPP15-1057<br>CPP15-1058<br>CPP15-1059<br>CPP15-1060	SSFHDNLKRTRRIHL<br>SFHDNLKRTRRIHLN<br>FHDNLKRTRRIHLNI<br>HDNLKRTRRIHLNIR<br>DNLKRTRRIHLNIRE<br>NLKRTRRIHLNIREC<br>LKRTRRIHLNIRECC	CPP20-573<br>CPP20-574<br>CPP20-575	FIILLLLIASAPSFDALP<br>KT<br>KTEDNVPLSSFHDNL<br>KRTRR<br>KRTRRIHLNIRECCSD<br>GWCC
Conotoxin Pn-B01121 Q9BPF3	CPP10-1703<br>CPP10-1704	KRTLQILSNK<br>RTLQILSNKR				
Conotoxin PnMLCL-01 Q9BPE2	CPP10-1705<br>CPP10-1706	FIILLLLASP<br>KRIQSDLIRA				
Conotoxin Pn-B01411 Q9BPE7	CPP10-1707<br>CPP10-1708<br>CPP10-1709<br>CPP10-1710<br>CPP10-1711<br>CPP10-1712<br>CPP10-1713<br>CPP10-1714<br>CPP10-1715<br>CPP10-1716<br>CPP10-1717<br>CPP10-1718	SSFRDNLKRT<br>FRDNLKRTLR<br>RDNLKRTLRT<br>DNLKRTLRTL<br>NLKRTLRTLL<br>LKRTLRTLLD<br>KRTLRTLLDP<br>RTLRTLLDPR<br>TLRTLLDPRR<br>LRTLLDPRRC<br>RTLLDPRRCC<br>RRCCYETPGC	CPP15-1061<br>CPP15-1062<br>CPP15-1063<br>CPP15-1064<br>CPP15-1065<br>CPP15-1066<br>CPP15-1067<br>CPP15-1068<br>CPP15-1069	LSSFRDNLKRTLRTL<br>SSFRDNLKRTLRTLL<br>SFRDNLKRTLRTLLD<br>FRDNLKRTLRTLLDP<br>RDNLKRTLRTLLDPR<br>DNLKRTLRTLLDPRR<br>NLKRTLRTLLDPRRC<br>LKRTLRTLLDPRRCC<br>TLLDPRRCCYETPGC	CPP20-576<br>CPP20-577<br>CPP20-578<br>CPP20-579<br>CPP20-580<br>CPP20-581<br>CPP20-582	FIILLLLMASAPSFDA<br>RPKT<br>PLSSFRDNLKRTLRTL<br>LDPR<br>LSSFRDNLKRTLRTLL<br>DPRR<br>SSFRDNLKRTLRTLLD<br>PRRC<br>SFRDNLKRTLRTLLD<br>PRRCC<br>LKRTLRTLLDPRRCCY<br>ETPG<br>KRTLRTLLDPRRCCY<br>ETPGC
Conotoxin PnMRCL-022 Q9BPH2	CPP10-1719<br>CPP10-1720<br>CPP10-1721<br>CPP10-1722<br>CPP10-1723<br>CPP10-1724<br>CPP10-1725	PSVNARPKTK<br>SVNARPKTKD<br>NARPKTKDLA<br>KRTQHIFWSK<br>RTQHIFWSKR<br>QHIFWSKRNC<br>HIFWSKRNCC	CPP15-1070<br>CPP15-1071	FHDNAKRTQHIFWSK<br>KRTQHIFWSKRNCCI	CPP20-583	KRTQHIFWSKRNCCI<br>YENWC

	Conotoxin Pn-B0151<br>Q9BPG0	CPP10-1726<br>CPP10-1727	KRILQTLSNK<br>RILQTLSNKR	CPP15-1072	KRILQTLSNKRSCCP	CPP20-584	KRILQTLSNKRSCCPT<br>ILSC
	Conotoxin Pn-B01122<br>Q9BPF1	CPP10-1728<br>CPP10-1729<br>CPP10-1730	PSVDARPKTK<br>KRALQILSNK<br>RALQILSNKR				
	Conotoxin<br>Q9BPF0	CPP10-1731<br>CPP10-1732<br>CPP10-1733<br>CPP10-1734<br>CPP10-1735<br>CPP10-1736<br>CPP10-1737	PSIDARPKTK<br>SIDARPKTKD<br>LSSFHARPKT<br>SSFHARPKTK<br>SFHARPKTKD<br>HARPKTKDDV<br>QQRAMEPWTR	CPP15-1073	KDDVPLSSFHARPKT	CPP20-585	ARPKTKDDVPLSSFH<br>ARPKT
	Conotoxin<br>Q9BH99	CPP10-1738	KLWNHARDWC				
Conus coronatus	A superfamily conotoxin Co1.3<br>S4UJN8	CPP10-1739<br>CPP10-1740<br>CPP10-1741	GRNAAAKVFK<br>RNAAAKVFKR<br>KRIALIATRE	CPP15-1074<br>CPP15-1075<br>CPP15-1076	RASHGRNAAAKVFKR<br>ASHGRNAAAKVFKRI<br>RNAAAKVFKRIALIA		
	A superfamily conotoxin Co1.6<br>S4UJD3	CPP10-1742<br>CPP10-1743	ASCWQRYPER<br>SCWQRYPERC				
	Four-loop conotoxin (Fragment)<br>A9P3W5	CPP10-1744<br>CPP10-1745<br>CPP10-1746<br>CPP10-1747<br>CPP10-1748<br>CPP10-1749<br>CPP10-1750<br>CPP10-1751<br>CPP10-1752<br>CPP10-1753<br>CPP10-1754<br>CPP10-1755<br>CPP10-1756<br>CPP10-1757	TSSRGKQKHR<br>SSRGKQKHRA<br>SRGKQKHRAL<br>RGKQKHRALR<br>GKQKHRALRS<br>KQKHRALRST<br>KHRALRSTDK<br>ALRSTDKNSR<br>DKNSRMTKRC<br>KNSRMTKRCT<br>NSRMTKRCTP<br>SRMTKRCTPA<br>RMTKRCTPAG<br>KRCTPAGKAC	CPP15-1077<br>CPP15-1078<br>CPP15-1079<br>CPP15-1080<br>CPP15-1081<br>CPP15-1082<br>CPP15-1083<br>CPP15-1084<br>CPP15-1085<br>CPP15-1086<br>CPP15-1087<br>CPP15-1088<br>CPP15-1089	QLTTAETSSRGKQKH<br>AETSSRGKQKHRALR<br>TSSRGKQKHRALRST<br>SRGKQKHRALRSTDK<br>RGKQKHRALRSTDKN<br>GKQKHRALRSTDKNS<br>KQKHRALRSTDKNSR<br>KHRALRSTDKNSRMT<br>ALRSTDKNSRMTKRC<br>LRSTDKNSRMTKRCT<br>RSTDKNSRMTKRCTP<br>STDKNSRMTKRCTPA<br>NSRMTKRCTPAGKAC	CPP20-586<br>CPP20-587<br>CPP20-588<br>CPP20-589<br>CPP20-590<br>CPP20-591<br>CPP20-592<br>CPP20-593<br>CPP20-594	QLTTAETSSRGKQKH<br>RALRS<br>TAETSSRGKQKHRAL<br>RSTDK<br>GKQKHRALRSTDKN<br>SRMTKR<br>KQKHRALRSTDKNS<br>RMTKRC<br>QKHRALRSTDKNSR<br>MTKRCT<br>KHRALRSTDKNSRM<br>TKRCTP<br>HRALRSTDKNSRMT<br>KRCTPA<br>ALRSTDKNSRMTKR<br>CTPAGK<br>RSTDKNSRMTKRCT<br>PAGKAC

	Four-loop<br>conotoxin<br>(Fragment)<br>A9P3W2	CPP10-1758<br>CPP10-1759<br>CPP10-1760<br>CPP10-1761<br>CPP10-1762<br>CPP10-1763<br>CPP10-1764<br>CPP10-1765<br>CPP10-1766<br>CPP10-1767<br>CPP10-1768<br>CPP10-1769<br>CPP10-1770<br>CPP10-1771	TSSRGKQKHR<br>SSRGKQKHRA<br>SRGKQKHRAL<br>RGKQKHRALR<br>GKQKHRALRS<br>KQKHRALRST<br>KHRALRSTDK<br>ALRSTDKNSR<br>DKNSRMTKRC<br>KNSRMTKRCT<br>SRMTKRCTSP<br>RMTKRCTSPG<br>SKACNLTTKR<br>KACNLTTKRC	CPP15-1090<br>CPP15-1100<br>CPP15-1101<br>CPP15-1102<br>CPP15-1103<br>CPP15-1104<br>CPP15-1105<br>CPP15-1106<br>CPP15-1107<br>CPP15-1108<br>CPP15-1109	QLTTAETSSRGKQKH<br>AETSSRGKQKHRALR<br>TSSRGKQKHRALRST<br>SRGKQKHRALRSTDK<br>RGKQKHRALRSTDKN<br>GKQKHRALRSTDKNS<br>KQKHRALRSTDKNSR<br>KHRALRSTDKNSRMT<br>ALRSTDKNSRMTKRC<br>LRSTDKNSRMTKRCT<br>STDKNSRMTKRCTSP	CPP20-595<br>CPP20-596<br>CPP20-597<br>CPP20-598<br>CPP20-599<br>CPP20-600<br>CPP20-601	QLTTAETSSRGKQKH<br>RALRS<br>TAETSSRGKQKHRAL<br>RSTDK<br>GKQKHRALRSTDKN<br>SRMTKR<br>KQKHRALRSTDKNS<br>RMTKRC<br>QKHRALRSTDKNSR<br>MTKRCT<br>KHRALRSTDKNSRM<br>TKRCTS<br>HRALRSTDKNSRMT<br>KRCTSP
	Four-loop<br>conotoxin<br>(Fragment)<br>A9P3W9	CPP10-1772<br>CPP10-1773<br>CPP10-1774<br>CPP10-1775<br>CPP10-1776<br>CPP10-1777<br>CPP10-1778<br>CPP10-1779	TSSRGKQKHR<br>SSRGKQKHRA<br>SRGKQKHRAL<br>RGKQKHRALR<br>GKQKHRALRS<br>KQKHRALRST<br>KHRALRSTDK<br>ALRSTDKNSK	CPP15-1110<br>CPP15-1111<br>CPP15-1112<br>CPP15-1113<br>CPP15-1114<br>CPP15-1115<br>CPP15-1116<br>CPP15-1117	QLTTAETSSRGKQKH<br>AETSSRGKQKHRALR<br>TSSRGKQKHRALRST<br>SRGKQKHRALRSTDK<br>RGKQKHRALRSTDKN<br>GKQKHRALRSTDKNS<br>KQKHRALRSTDKNSK<br>KHRALRSTDKNSKMT	CPP20-602<br>CPP20-603<br>CPP20-604<br>CPP20-605<br>CPP20-606<br>CPP20-607	QLTTAETSSRGKQKH<br>RALRS<br>TAETSSRGKQKHRAL<br>RSTDK<br>SSRGKQKHRALRSTD<br>KNSKM<br>GKQKHRALRSTDKN<br>SKMTRC<br>KQKHRALRSTDKNS<br>KMTRCT<br>KHRALRSTDKNSKM<br>TRCTDP
Conus frigidus	Contryphan-Fr1<br>A0A1P8NVT3	CPP10-1780<br>CPP10-1781	FMNVLRRSGC<br>MNVLRRSGCP				
